# Quantitative proteomic and phenotypic responses of urinary pathogens to CuO/Cu₂O nanoparticles

**DOI:** 10.1080/17435889.2025.2579616

**Published:** 2025-10-29

**Authors:** Kidon Sung, Miseon Park, Ohgew Kweon, Alena Savenka, Angel Paredes, Saeed Khan, Seonggi Min, Steven Foley

**Affiliations:** a Division of Microbiology, National Center for Toxicological Research, U.S. Food and Drug Administration, Jefferson, AR, USA; b Nanotechnology Core Facility, Office of Scientific Coordination, National Center for Toxicological Research, U.S. Food and Drug Administration, Jefferson, AR, USA; c Division of Biochemical Toxicology, National Center for Toxicological Research, U.S. Food and Drug Administration, Jefferson, AR, USA

**Keywords:** Copper nanoparticles, urinary pathogens, proteome, *Enterococcus faecalis*, *Proteus mirabilis*

## Abstract

**Aims:**

To evaluate the antibacterial efficacy of pulsed laser ablation-generated copper oxide (CuO/Cu₂O) nanoparticles (NPs) against urinary pathogens and to elucidate molecular stress responses through proteomic profiling.

**Methods:**

*Enterococcus faecalis*, *Proteus mirabilis*, *Escherichia coli*, and *Pseudomonas aeruginosa* were exposed to CuO/Cu₂O NPs. Viability was assessed by colony-forming unit counts, while morphological alterations were examined using field emission scanning electron microscopy (FESEM). Quantitative proteomic analysis with COG and KEGG bioinformatics was performed for *E. faecalis* and *P. mirabilis* at early exposure times (10–60 min).

**Results:**

CuO/Cu₂O NPs significantly reduced viability, with *E. coli* and *P. aeruginosa* fully inhibited after 30 min, whereas *P. mirabilis* showed relative resistance. FESEM revealed nanoparticle-induced membrane rupture and cell deformation. Proteomic analysis identified conserved and species-specific stress responses. Shared adaptations included upregulation of energy metabolism, transcription, and transport pathways. *E. faecalis* uniquely increased carbohydrate metabolism and cell wall biogenesis, while *P. mirabilis* emphasized ribosome biogenesis, ion transport, and nucleotide metabolism. Virulence-associated proteins were differentially expressed, linking stress adaptation to pathogenicity.

**Conclusions:**

CuO/Cu₂O NPs exert rapid antibacterial activity via oxidative stress, membrane disruption, and metabolic reprogramming. Distinct proteomic adaptations explain species differences in susceptibility and highlight copper nanoparticles as promising antimicrobial candidates.

## 1. Introduction

Antibiotics are valuable tools for combating infectious diseases, but their indiscriminate use in human medicine, agriculture, and aquaculture is driving the alarming rise of antibiotic resistance [1]. In 2019, 4.95 million individuals succumbed to antibiotic-resistant infections [2]. Biofilm-embedded pathogens exhibit significantly higher antibiotic resistance in comparison to their free-floating counterparts, necessitating innovative therapeutic approaches beyond traditional antibiotics [3]. Consequently, enhancing the effectiveness of existing treatments for infectious diseases has become a critical public health imperative.

Urinary tract infections (UTIs) are a significant global health issue, impacting more than 404 million individuals each year [4]. They are a major cause of secondary bloodstream infections, contributing to approximately 17% of hospital-acquired bacteremia [5]. Uropathogens infect the urinary tract by colonizing catheters or uroepithelial cells, evading host defenses, and causing tissue damage [6]. UTIs are primarily caused by Gram-negative bacteria, with *Escherichia coli* being the most prevalent pathogen [7]. Other bacteria, such as *Staphylococcus, Enterococcus, Klebsiella, Proteus*, and *Pseudomonas*, also frequently contribute to UTIs.

Nanotechnology has enabled the development of a diverse array of nanomaterials with promising applications in medicine, biotechnology, and physics [8]. Nanoparticles (NPs) exhibit exceptional properties due to their diminutive size, resulting in an extraordinarily high surface area to volume ratio and elevated surface energy [9]. Fabricated from a diverse range of materials including gold, titanium, zinc, selenium, silver, and copper, NPs have found broad applications across the biomedical field [9].

Copper, the third most abundant essential trace element in the human body, is a critical cofactor in various physiological processes, including the maintenance of cellular and genetic integrity [10]. Copper NPs have been extensively studied for their potent antibacterial properties, driven by their nanoscale dimensions and high surface reactivity [11]. They effectively minimize resistance development by simultaneously targeting multiple bacterial pathways [12], and their ability to disrupt and penetrate biofilms makes them potent against chronic and device-associated infections [13]. Additionally, copper NPs often enhance therapeutic efficacy through synergistic effects when combined with antimicrobial agents [14]. Their low cytotoxicity toward human cells further highlights their potential for safe and effective biomedical applications [15]. These combined attributes underscore the significant potential of copper NPs as a next-generation antimicrobial agent, addressing the growing challenge of antibiotic resistance and persistent infections.

Copper oxide nanoparticles (CuO/Cu₂O NPs) exert antibacterial effects through multi target pathways, including Cu(I/II) ion release and reactive oxygen species (ROS) generation, leading to lipid peroxidation, protein and nucleic acid damage, and membrane perturbation; they also show activity within biofilms and may synergize with conventional antibiotics [16,17]. Dissolution kinetics, valence state, and surface chemistry jointly determine biological responses, with nanoparticle-specific contributions beyond dissolved copper [13]. These properties motivate time resolved studies that link phenotypic killing to molecular adaptations, particularly for urinary pathogens.

Pulsed laser ablation in liquid (PLAL) is a versatile technique for generating particles spanning the micro- to nanoscale directly from bulk materials [18]. This process involves focusing pulsed laser radiation on a submerged target, resulting in the formation of NPs within the liquid medium. PLAL stands out as a rapid, cost-effective, and environmentally friendly approach that eliminates the need for harmful chemicals. By producing ligand-free NPs in a single step, PLAL offers significant advantages over traditional chemical synthesis methods, particularly for biomedical applications requiring pristine NPs [19].

Despite numerous reports demonstrating the antibacterial activity of copper NPs, the time-resolved molecular responses of clinically relevant urinary pathogens remain poorly defined. In this study, we address this critical knowledge gap by integrating rapid bactericidal assays and field emission scanning electron microscopy (FESEM) with quantitative proteomic profiling to capture time-dependent, species-specific adaptations to PLAL-generated CuO/Cu₂O NPs. Using a panel of major urinary pathogens—*E. coli, P. aeruginosa, E. faecalis*, and *P. mirabilis*—we identified both conserved stress programs and distinct, species-specific proteomic signatures. This combined antimicrobial and proteomic approach provides a unique systems-level perspective on how urinary pathogens mount defense strategies against copper NP stress, advancing mechanistic insights beyond prior studies that primarily focused on antimicrobial endpoints alone.

## 2. Materials and methods

### 2.1. Synthesis of CuO/Cu_2_O NPs

CuO/Cu₂O NPs used in this study were synthesized by pulsed laser ablation in liquid (PLAL) in Professor Gregory Guisbiers’ laboratory (School of Physical Sciences, University of Arkansas at Little Rock, Arkansas) and provided to our team for testing. Copper (Cu, 99.99%, ~2 mm diameter) beads (MilliporeSigma, Burlington, MA) served as target materials. Spherical NPs were synthesized using a Nd:YAG laser (Electro Scientific Industries, Portland, OR) emitting a 1,064 nm infrared beam deflected at a 45° angle. The Cu beads were fully submerged in deionized water, with water depths of approximately 10 mm. The laser repetition rate varied from 1 to 15 kHz, with pulse energies of 5.5 mJ at 1 kHz. Successful preparation of CuO/Cu₂O NPs was verified for the experimental batch. Transmission and scanning electron microscopy (TEM/SEM) were used to assess morphology and primary particle size. Dynamic light scattering (DLS) and zeta potential measurements determined hydrodynamic size distribution and colloidal stability under the assay buffer conditions. UV–visible spectroscopy verified optical features consistent with copper oxide nanostructures. Powder X-ray diffraction (XRD) confirmed the presence of CuO/Cu₂O crystalline phases, and X-ray photoelectron spectroscopy (XPS) resolved Cu(I)/Cu(II) oxidation states. The results can be found in our prior detailed characterization of PLAL generated CuO/Cu₂O NPs [20,21].

### 2.2. Antibacterial activity

This study utilized three Gram-negative uropathogenic bacteria—*Escherichia coli* ATCC 700,928, *Proteus mirabilis* ATCC 7002, and *Pseudomonas aeruginosa* PA14—as well as one Gram-positive uropathogen, *Enterococcus faecalis* ATCC 29,212. *E. coli* ATCC 700,928, *P. mirabilis* ATCC 7002, and *E. faecalis* ATCC 29,212 were purchased from ATCC (Manassas, VA), while *P. aeruginosa* PA14 was generously provided by Professor Vincent Lee, Department of Cell Biology and Molecular Genetics, University of Maryland, College Park. All strains were recovered from −80°C stocks and cultured on tryptic soy agar (Thermo Fisher Scientific, Waltham, MA). A single colony was inoculated into 10 mL of tryptic soy broth (Thermo Fisher Scientific) and incubated overnight at 37°C with vigorous shaking. Bacterial cells were harvested by centrifugation at 14,000 rpm for 10 min at 4°C, washed with phosphate-buffered saline (PBS, Thermo Fisher Scientific). Bacterial suspensions were adjusted to an optical density of 0.01 at 600 nm with 3 ppm CuO/Cu_2_O NPs. The mixtures were incubated with continuous mixing for 10, 30, or 60 min. A positive control (testing medium with bacteria) and a negative control (medium only) were included. Bacterial viability was quantified by colony-forming unit counts after serial dilution and plating on tryptic soy agar. All experiments were performed in triplicate. For accurate interpretation of antibacterial activity, the starting inoculum corresponding to an optical density (OD₆₀₀) of 0.1 was determined for each bacterial species. The approximate baseline values were as follows: *P. mirabilis* ATCC 7002, ~2.0 × 10^7^ CFU/mL; *E. coli* CFT073, ~3.02 × 10^7^ CFU/mL; *E. faecalis* ATCC 29,212, ~4.66 × 10^6^ CFU/mL; and *P. aeruginosa* PA14, ~2.9 × 10^7^ CFU/mL. These baseline values provide a quantitative reference point for the reductions in viable counts observed after CuO/Cu₂O NP treatment. Statistical analyses, including one-way Analysis of Variance and post-hoc pairwise t-tests, were performed to determine the significance of bacterial colony-forming unit reductions over time.

### 2.3. Field emission scanning electron microscope (FESEM)

Bacterial cells were washed thrice with PBS, followed by overnight fixation in 2.5% (v/v) glutaraldehyde at 4°C. Dehydration was achieved through a graded ethanol series (15%, 50%, 90%, 95%, 100%). Next, cells were treated with various hexamethyldisilazane (MilliporeSigma) and ethanol mixtures (ratios: 1:2, 1:1, 2:1), as well as with pure hexamethyldisilazane. Finally, samples were sputter-coated with gold/palladium (Denton Vacuum, Moorestown, NJ), and imaged using a Zeiss-Merlin Gemini2 FESEM (Carl Zeiss Microscopy, White Plains, NY).

### 2.4. Protein extraction

We selected *E. faecalis* ATCC 29,212 (10, 30, and 60 min) and *P. mirabilis* ATCC 7002 (10 and 30 min) for proteome analysis, as *E. coli* ATCC 700,928 became undetectable after 10 min and *P. aeruginosa* PA14 exhibited complete inhibition throughout. Bacterial cells were centrifuged at 14,000 rpm and 4°C for 1 min. After removing the supernatant, the pellets were washed with PBS and resuspended in the BugBuster Plus Lysonase kit (MilliporeSigma), which contains BugBuster Protein Extraction Reagent, rLysozyme, and Benzonase Nuclease. To lyse the bacterial cells, the suspension was transferred to Lysing Matrix tubes (MP Biomedicals, Santa Ana, CA) and lysed using FastPrep-24 (MP Biomedicals). To further disrupt the bacterial cells, the lysate was boiled and vortexed. Finally, the protein extract was obtained by centrifugation at 14,000 rpm for 30 min at 4°C.

### 2.5. Protein sample preparation

Bacterial lysates were initially precipitated using trichloroacetic acid (MilliporeSigma) to remove contaminants and concentrate the protein content. After precipitation and washing, the protein pellet was solubilized in a buffer consisting of 8 M urea and 50 mM Tris-HCl at pH 8.0, supplemented with 1X Roche Complete Protease Inhibitor (MilliporeSigma). The samples underwent reduction for 1 h at room temperature with 12 mM dithiothreitol (MilliporeSigma), followed by alkylation for an additional hour at room temperature using 15 mM iodoacetamide (MilliporeSigma). Subsequently, trypsin (MilliporeSigma) was added at an enzyme-to-substrate ratio of 1:20. Each sample was then acidified with 0.3% trifluoroacetic acid (TFA, MilliporeSigma) and subjected to solid-phase extraction using μHLB (Waters, Milford, MA). The solid-phase extraction procedure included matrix activation with four additions of 70% acetonitrile (MilliporeSigma), equilibration with four additions of 0.3% TFA, washing with three additions of 0.3% TFA, and elution with 60% acetonitrile in 0.3% TFA, followed by a second elution with 60% acetonitrile in 0.3% TFA. The resulting eluates were frozen at −80°C and lyophilized overnight. Finally, the peptides were reconstituted in 0.1% TFA for subsequent analysis.

### 2.6. Mass spectrometry analysis

#### 2.6.1. Data-independent acquisition (DIA) chromatogram library generation

One microgram of the peptide pool was analyzed by nano liquid chromatography-tandem mass spectrometry (LC-MS) using an M-class high performance liquid chromatography system (Waters) coupled to an Orbitrap Exploris 480 Mass Spectrometer (Thermo Fisher Scientific). After packing a trapping column with XSelect CSH C18 resin (5 μm particle size, Waters), peptides were separated on a 75 μm analytical column packed with the same resin (2.4 μm particle size). Both columns were maintained at 55°C. Peptides were eluted with a 30-min linear gradient at a flow rate of 350 nL/minute. The mass spectrometer was operated in data-independent acquisition (DIA) mode. Six gas-phase fractionation injections were acquired across six mass ranges (396–502, 496–602, 596–702, 696–802, 796–902, and 896–1002 m/z). Each injection consisted of a full MS scan at 60,000 resolution followed by 26 MS/MS scans with 4 m/z isolation windows, interspersed with another full MS scan and an additional 26 MS/MS scans with staggered isolation windows. MS/MS spectra were acquired at 30,000 resolution. Automatic gain control target and maximum ion injection time were set to 1e6 and 50 ms, respectively, for full MS scans. For MS/MS scans, dynamic ion injection time was used with a target of nine data points across the peak, and normalized collision energy (NCE) was set to 30.

#### 2.6.2. Sample analysis

One microgram per sample was analyzed using the LC-MS conditions described for library generation; however, a different DIA acquisition method was used. Full MS scans were acquired from m/z 385–1015 at 60,000 resolution, followed by 61 tandem mass spectrometry (MS/MS) scans with 10 m/z isolation windows. MS/MS spectra were acquired at 15,000 resolution. Other mass spectrometry parameters were identical to those used for library generation.

#### 2.6.3. Data processing and bioinformatic analysis

The DIA data were analyzed using Scaffold DIA version 3.2.1 (Proteome Software, Portland, OR), which facilitated several key functions. Initially, raw files were converted to mzML format (ProteoWizard version 3.0.19254, Proteome Software), including the deconvolution of staggered windows, and subsequently converted to DIA format. Retention times were aligned for consistency. The data were searched using the Prosit library (DLIB) and the chromatogram/reference library to create a custom ELIB with the search parameters set to a precursor mass tolerance of 10 ppm, a fragment mass tolerance of 10 ppm, and a library fragment tolerance of 10 ppm. Peptide identification results were filtered at a 1% false discovery rate using Percolator, a semi-supervised learning for peptide identification from shotgun proteomics datasets [22]. Peak areas for detected peptides were calculated using Encyclopedia version 1.12.31 (Proteome Software), with the five highest-quality fragment ions selected for quantitation. Finally, data normalization was performed based on the total protein intensity per sample. Proteins were considered differentially expressed if their expression levels changed significantly compared to those of the control group. Specifically, proteins were upregulated if their fold changes were 2.0 or greater, and downregulated if their fold changes were 0.5 or less.

The functional annotation of proteins was conducted using the Clusters of Orthologous Groups (COG) database [23]. Additionally, Kyoto Encyclopedia of Genes and Genomes (KEGG) pathway analysis was used to link these differentially expressed proteins to specific biological processes [24]. To cluster protein expression (EPN) data of treatment time from our proteomic experiments, we employed a custom Python script [25], which computed fold ratios for the proteins and utilized these ratios to both cluster and annotate expression patterns. This approach enabled the effective identification of significant temporal expression patterns and highlighted proteins exhibiting these trends.

## 3. Results

### 3.1. Antibacterial activity of CuO/Cu_2_O NPs

**Figure 1.**
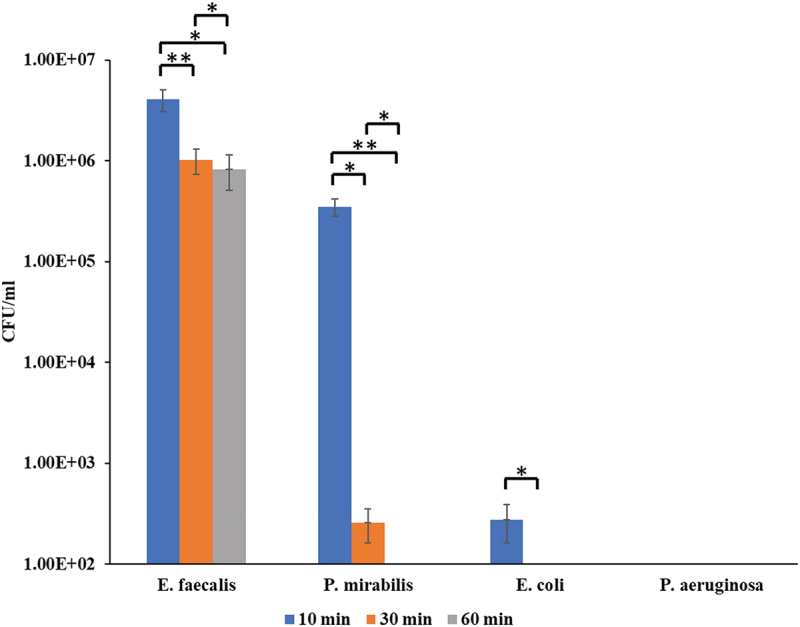
Bactericidal activities of CuO/Cu₂O NPs. A single asterisk (*) indicates statistically significant differences between groups at *p* < 0.05, while two asterisks (**) denote a more stringent significance level at *p* < 0.01.

The antibacterial activity of NPs was evaluated against four urinary pathogens (*E. faecalis* ATCC 29,212, *P. mirabilis* ATCC 7002, *E. coli* ATCC 700,928, and *P. aeruginosa* PA14) at three time points: 10, 30, and 60 min (Figure 1). The cell count of *E. faecalis* decreased significantly from 4.08 × 10^6^ at 10 min to 1.02 × 10^6^ at 30 min (*p* = 0.004) and 8.25 × 10^5^ at 60 min (*p* = 0.015). *P. mirabilis* cell count dropped from 3.5 × 10^5^ at 10 min to 258.33 at 30 min (p = 0.021) and was undetectable at 60 min (p = 0.003). For *E. coli*, cell numbers declined from 275 at 10 min to undetectable levels at 30 and 60 min (p = 0.018). No viable cells were detected for *P. aeruginosa* at any time point, indicating complete inhibition. In all cases, positive control samples showed significantly higher CFU counts than CuO/Cu₂O NP-treated samples.

### 3.2. Microscopic examination of bacterial cells following CuO/Cu_2_O NP treatment

**Figure 2.**
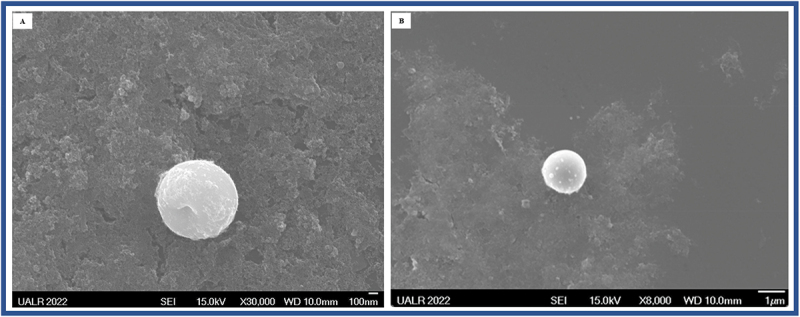
Representative sem image of the CuO/Cu₂O NPs. (A) Lower magnification views (scale bar: 100 nm)μm), (B) Higher magnification views (scale bar: 1 µm). SEM was a JSM—7000F from JEOL that employs a Schottky-type field emission gun for the elec-tron source, and was operating at 15 kV.

**Figure 3.**
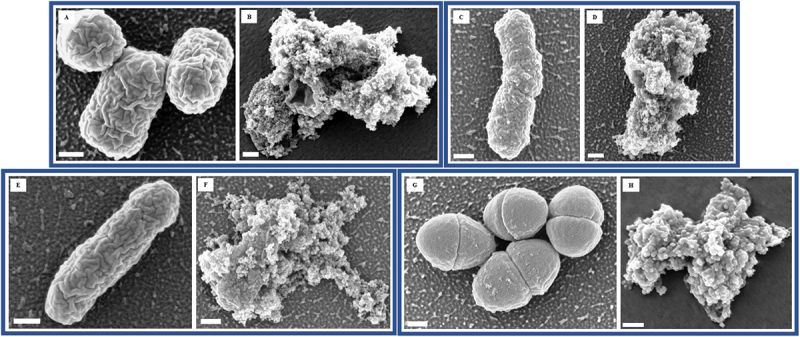
FESEM images of *E. coli* ATCC 700,928 (A, B), *P. mirabilis* ATCC 7002 (C, D), *P. aeruginosa* PA14 (E, F), and *E. faecalis* ATCC 29,212 (G, H). The scale bar in the images corresponds to 200 nm. A, C, E, and G are untreated (controls) and B, D, F, and H are CuO/Cu_2_O NP-treated for 10 min.

The shape of the CuO/Cu₂O NPs was confirmed to be spherical by SEM (Figure 2(A,B)). FESEM images revealed severe morphological damage to bacterial cells treated with CuO/Cu₂O NPs (Figure 3). The untreated control images (A, C, E, G) showed intact uropathogens with smooth and well-defined cellular structures. In contrast, images B, D, F, and H showed that CuO/Cu₂O NPs caused severe disruption to the uropathogens’ cellular structures. The cells exhibited pronounced surface roughness, deformation, membrane disruption, and loss of integrity.

### 3.3. Proteome expression profile of *E. faecalis* ATCC 29,212

**Figure 4.**
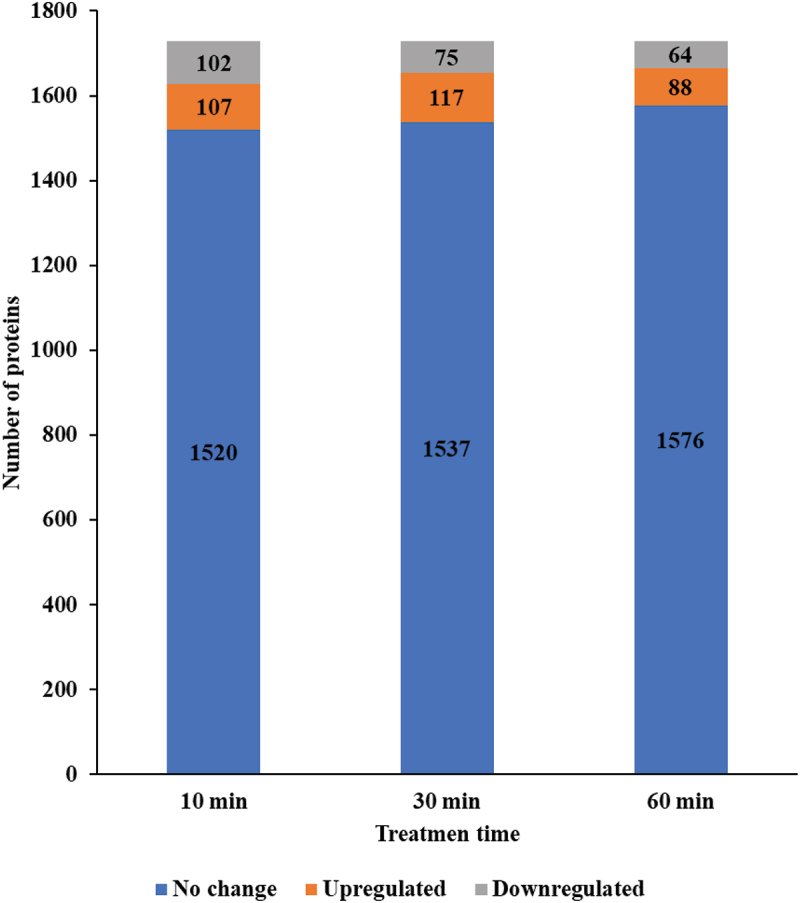
Number of differentially expressed proteins in *E. faecalis* ATCC 29,212 following CuO/Cu₂O NP treatment.

CuO/Cu₂O NP treatment induced time-dependent variations in the proteome of *E. faecalis* (Figure 4, Table S1). The number of upregulated proteins peaked at 30 min, with 117 proteins (4.11% of the total proteome) showing increased expression, followed by a decline to 88 proteins (3.09%) at 60 min. In contrast, the number of downregulated proteins showed a slight decrease over time, reaching 64 proteins (2.25%) by 60 min.

**Figure 5.**
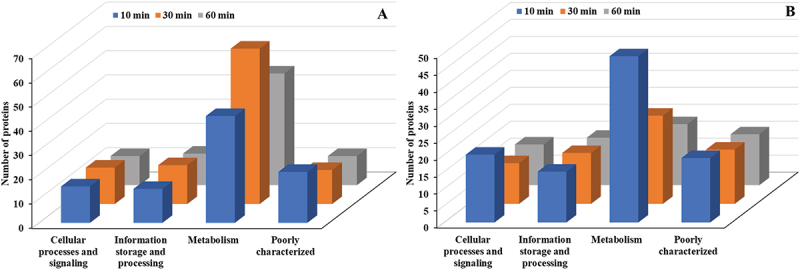
COG functional classification (major categories) of differentially expressed proteins in *E. faecalis* ATCC 29,212 after CuO/Cu₂O NP treatment. (A) Upregulated proteins. (B) Downregulated proteins.

Among the four primary COG categories – Information Storage and Processing, Cellular Processes and Signaling, Metabolism, and Poorly Characterized – Metabolism exhibited the most significant upregulation, reaching its peak activity at 30 min with 64 proteins (Figure 5(A)). Other categories, including Cellular processes and signaling, Information storage and processing, and Poorly characterized proteins, showed smaller changes. Downregulated proteins followed a similar trend, with Metabolism showing the largest reduction (49 proteins) at 10 min, while other categories displayed minimal variation over time (Figure 5(B)). Metabolism consistently exhibited the most significant changes in both upregulated and downregulated proteins.

**Figure 6.**
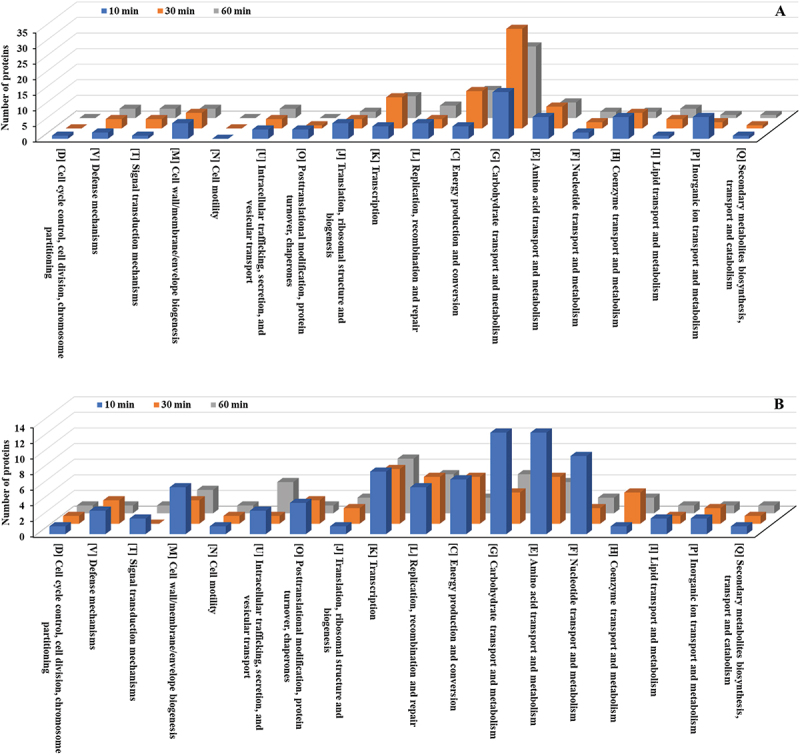
COG functional classification (detailed categories) of differentially expressed proteins in *E. faecalis* ATCC 29,212 after CuO/Cu₂O NP treatment. (A) Upregulated proteins. (B) Downregulated proteins. COG functional categories: J, translation, ribosomal structure, and biogenesis; K, transcription; L, replication, recombination, and repair; D, cell cycle control, cell division, chromosome partitioning; V, defense mechanisms; T, signal transduction mechanisms; M, cell wall/membrane/envelope biogenesis; N, cell motility; U, intracellular trafficking, secretion, and vesicular transport; O, post-translational modification, protein turnover, chaperones; C, energy production and conversion; G, carbohydrate transport and metabolism; E, amino acid transport and metabolism; F, nucleotide transport and metabolism; H, coenzyme transportand metabolism; I, lipid transport and metabolism; P, inorganic ion transport and metabolism; Q, secondary metabolite biosynthesis, transport, and catabolism. Poorly characterized (s) was excluded.

The Carbohydrate transport and metabolism (G) category had the highest number of upregulated proteins, followed by Energy production and conversion (C) and Transcription (K) (Figure 6(A), Table S2). Additional categories with significant upregulation included Amino acid transport and metabolism (E), Cell wall/membrane/envelope biogenesis (M), and Coenzyme transport and metabolism (H). Among downregulated proteins, Amino acid transport and metabolism (E) had the highest count, followed by Transcription (K) and Carbohydrate transport and metabolism (G) (Figure 6(B), Table S2). Notable reductions were also observed in Energy production and conversion (C) and Replication, recombination, and repair (L).

**Figure 7.**
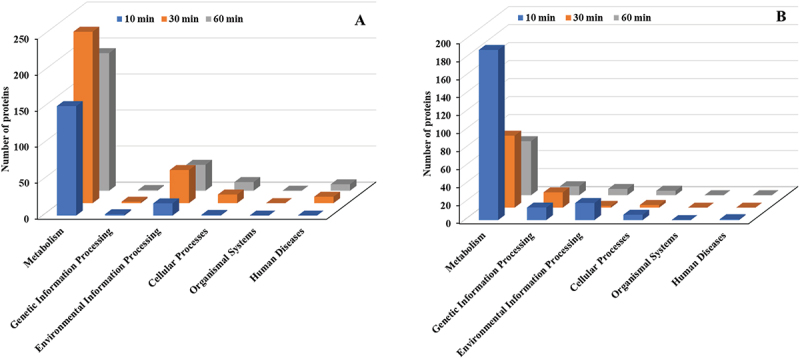
KEGG pathway classification (major groups) of differentially expressed proteins in *E. faecalis* ATCC 29,212 after CuO/Cu₂O NP treatment. (A) Upregulated proteins. (B) Downregulated proteins.

Among the six main KEGG categories (Metabolism, Cellular Processes, Environmental Information Processing, Genetic Information Processing, Organismal Systems, and Human Diseases), the Metabolism category showed the most extensive increase in upregulated proteins, highlighting a pronounced enhancement in metabolic processes (Figure 7(A)). Environmental Information Processing was the second most affected category, followed by Cellular Processes and Human Diseases. Similarly, for downregulated proteins, the Metabolism category exhibited the largest number of changes, indicating significant suppression of certain metabolic activities (Figure 7(B)). Genetic Information Processing was the second most affected category, followed by Environmental Information Processing.

**Figure 8.**
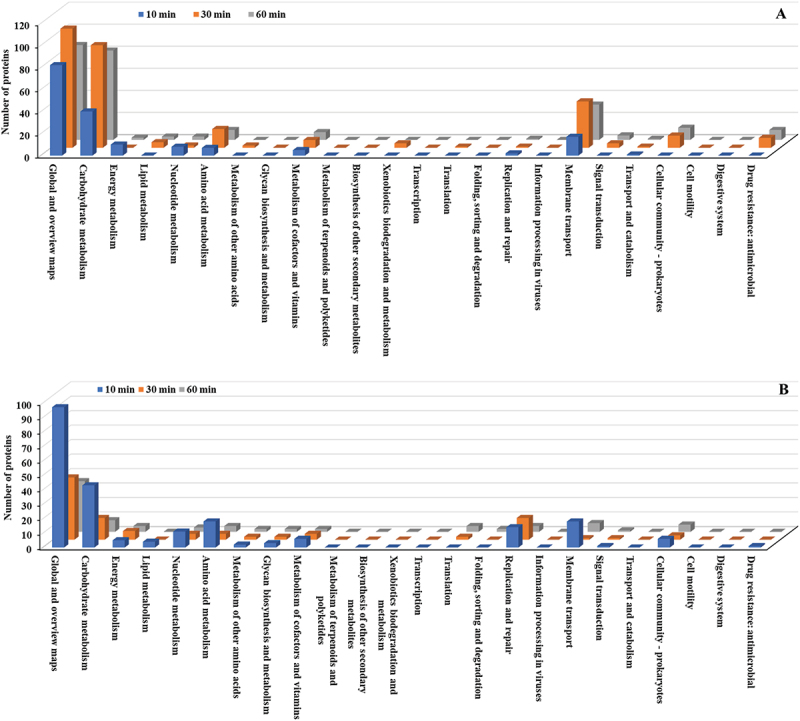
KEGG pathway classification (detailed pathways) of differentially expressed proteins in *E. faecalis* ATCC 29,212 after CuO/Cu₂O NP treatment. (A) Upregulated proteins. (B) downregulated proteins.

KEGG pathway analysis of *E. faecalis* treated with copper NPs revealed significant temporal shifts in protein expression across functional categories. Notably, Global and overview maps and Carbohydrate metabolism exhibited the highest activity among upregulated proteins, peaking at 30 min with 108 and 93 proteins, respectively (Figure 8(A), Table S3). Within the Membrane transport category, a substantial increase was observed in proteins associated with adenosine triphosphate (ATP)-binding cassette (ABC) transporters and the phosphotransferase system (PTS). Conversely, Global and overview maps and Carbohydrate metabolism displayed the most pronounced suppression among downregulated proteins (Figure 8(B), Table S4). Replication and repair also showed a marked reduction in protein abundance.

**Figure 9.**
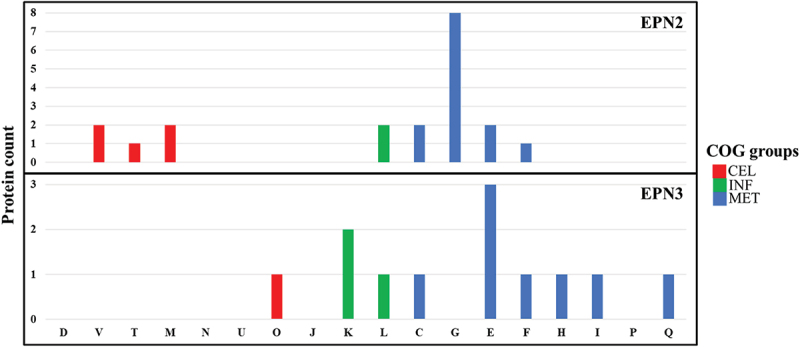
Protein expression patterns and functional distribution in *E. faecalis* ATCC 29,212 after CuO/Cu₂O NP treatment. cel, cellular processes and signaling; inf, Information storage and processing; met, metabolism. COG categories are as defined in Figure 5.

To identify proteins with similar EPNs, we utilized an in-house Python script. A total of 23 significant protein expression patterns were clustered (Table S1). Among these, the EPN2 dataset, comprising 225 proteins, showed consistent upregulation throughout the treatment (Figure 9, Table S6). Notably, proteins involved in Carbohydrate transport and metabolism, such as ArbF3, MalE, MapA, NplT, PtsG, SgcB, and YpcG, represented the most significantly upregulated group. Conversely, the EPN3 dataset included 15 proteins that were consistently downregulated (Figure 9, Table S7). Among these, proteins associated with Amino acid transport and metabolism, including Apc3 and TyrA, exhibited the most pronounced decrease in expression.

Seven differentially expressed proteins associated with virulence in *E. faecalis* were identified following CuO/Cu₂O NP treatment (Table 1). These proteins were involved in various functions, including adherence (YvcC), exotoxin production (CylA, CylL-S), immune modulation (GalE), nutrient acquisition (AllC, AllD), and stress survival (SodA). Among these, GalE and SodA were consistently upregulated throughout the treatment duration.

**Table 1.** Differentially expressed proteins associated with virulence in *E. faecalis* ATCC 29,212 following CuO/Cu₂O NP treatment.

				Fold ratio		
Protein ID	Protein description	Gene name	COG	10 min	30 min	60 min
AIL05596.1	Superoxide dismutase Mn	sodA	C	1.60	1.13	1.25
AIL05719.1	UDP-glucose 4-epimerase	galE	M	1.75	1.56	1.36
AIL03183.1	ClyA protein	cylA	O	−1.00	−1.21	−1.31
AIL04885.1	Allantoate amidohydrolase	allC	E	3.34	3.44	0.03
AIL03465.1	von Willebrand factor type A domain protein	yvcC	M	0.18	1.48	0.45
AIL04215.1	Ureidoglycolate dehydrogenase	allD	C	0.68	3.62	0.17
AIL03190.1	Lanthipeptide cytolysin subunit	cylL-S	S	0.10	−1.54	−1.31

### 3.4. Proteome expression profile of *P. mirabilis* ATCC 7002

**Figure 10.**
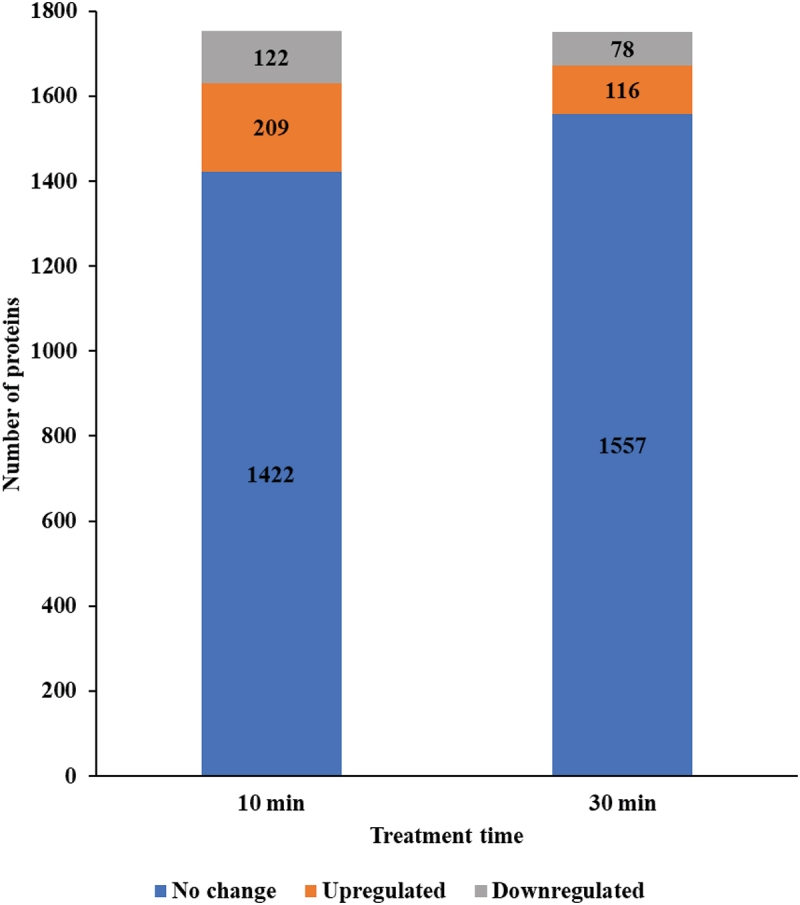
Number of differentially expressed proteins in *P. mirabilis* ATCC 7002 following CuO/Cu₂O NP treatment.

In *P. mirabilis* treated with CuO/Cu₂O NPs, 209 proteins (5.76% of total proteins) were upregulated, and 122 proteins (3.36%) were downregulated at 10 min (Figure 10, Table S8). By 30 min, the number of upregulated proteins decreased to 116 (3.19%), while that of downregulated proteins decreased to 78 (2.15%).

**Figure 11.**
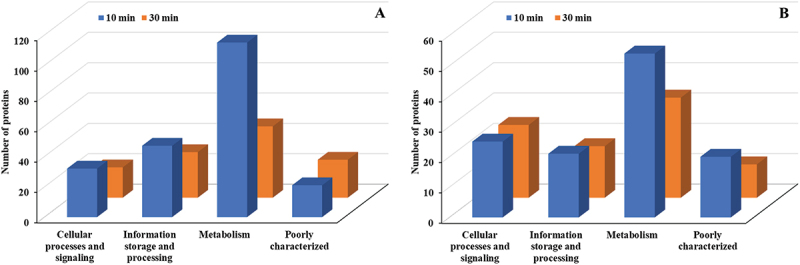
COG functional classification (major categories) of differentially expressed proteins in *P. mirabilis* ATCC 7002 after CuO/Cu₂O NP treatment. (A) Upregulated proteins. (B) Downregulated proteins.

In *P. mirabilis* treated with CuO/Cu₂O NPs, the distribution of differentially expressed proteins across the four main COG categories showed distinct temporal patterns at 10 and 30 min. The Metabolism category displayed the highest abundance of upregulated proteins, with 115 and 47 proteins identified at 10 and 30 min, respectively (Figure 11(A)); followed by Information storage and processing and Cellular processes and signaling. Downregulated proteins also peaked in the Metabolism category, with 54 and 33 proteins observed at 10 and 30 min, respectively, while the other COG categories exhibited smaller changes in protein expression (Figure 11(B)).

**Figure 12.**
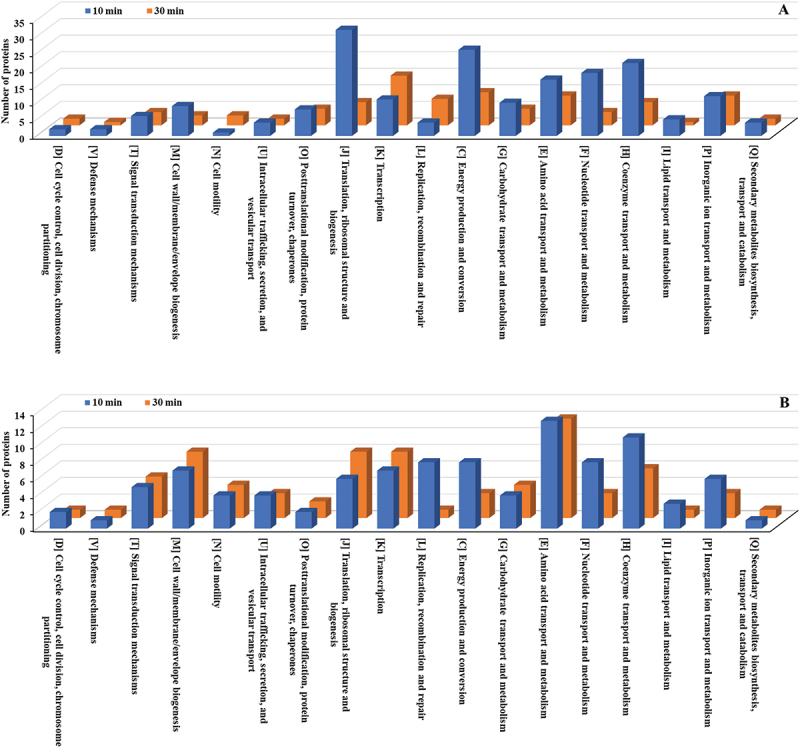
COG functional classification (detailed categories) of differentially expressed proteins in *P. mirabilis* ATCC 7002 after CuO/Cu₂O NP treatment. (A) Upregulated proteins. (B) Downregulated proteins. COG categories are as defined in Figure 5.

The upregulated proteins at 10 min were predominantly found in the Translation, ribosomal structure and biogenesis (J) category, with 32 proteins; followed by Energy production and conversion (C), with 26 proteins; and Coenzyme transport and metabolism (H), with 22 proteins (Figure 12(A), Table S9). By 30 min, these categories showed decreased activity. Among the downregulated proteins at 10 min, Amino Acid Transport and Metabolism (E) had the highest count (13 proteins), followed by Coenzyme Transport and Metabolism (H) (11 proteins) (Figure 12(B), Table S9), both of which showed further reductions by 30 min.

**Figure 13.**
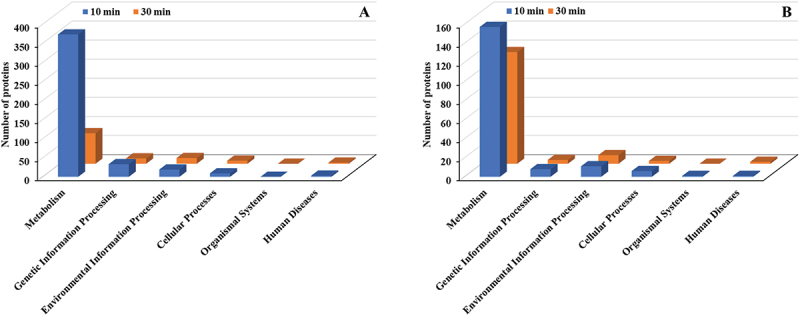
KEGG pathway classification (major groups) of differentially expressed proteins in *P. mirabilis* ATCC 7002 after CuO/Cu₂O NP treatment. (A) Upregulated proteins. (B) Downregulated proteins.

The six main KEGG category pathway analysis of *P. mirabilis* treated with CuO/Cu₂O NPs showed the Metabolism category as the most affected. Among upregulated proteins, Metabolism decreased from 373 proteins at 10 min to 80 at 30 min (Figure 13(A)). Similarly, among downregulated proteins, Metabolism dropped from 157 proteins at 10 min to 117 at 30 min (Figure 13(B)). Other KEGG categories exhibited relatively minor fluctuations in protein expression over time.

**Figure 14.**
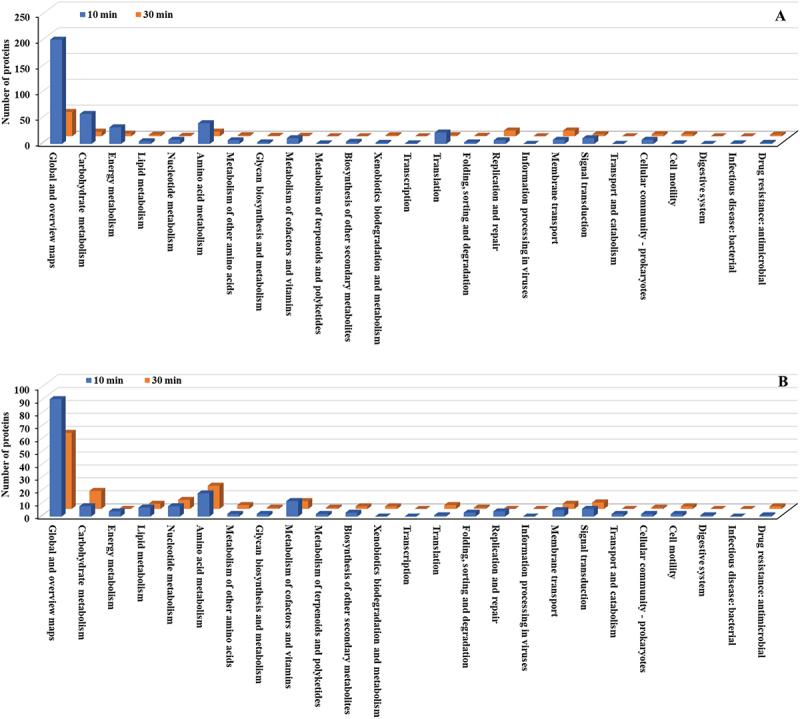
KEGG pathway classification (detailed pathways) of differentially expressed proteins in *P. mirabilis* ATCC 7002 after CuO/Cu₂O NP treatment. (A) Upregulated proteins. (B) Downregulated proteins.

At 10 min, the Global and overview maps category showed the highest upregulation with 202 proteins, followed by Carbohydrate metabolism (58 proteins) and Amino acid metabolism (40 proteins). Other significant pathways included Energy metabolism (32 proteins), Translation (22 proteins), Signal transduction (11 proteins), and Metabolism of cofactors and vitamins (11 proteins) (Figure 14(A), Table S10). By 30 min, most categories showed a substantial reduction. For downregulated proteins, the Global and overview maps category was most affected, with 91 proteins at 10 min, decreasing to 59 proteins at 30 min (Figure 14(B), Table S11). The Amino acid metabolism category remained consistently downregulated, with 18 proteins at both time points. Other categories with significant downregulation included Metabolism of cofactors and vitamins (12 proteins at 10 min, 6 at 30 min) and Carbohydrate metabolism (8 proteins at 10 min, increasing to 14 at 30 min).

**Figure 15.**
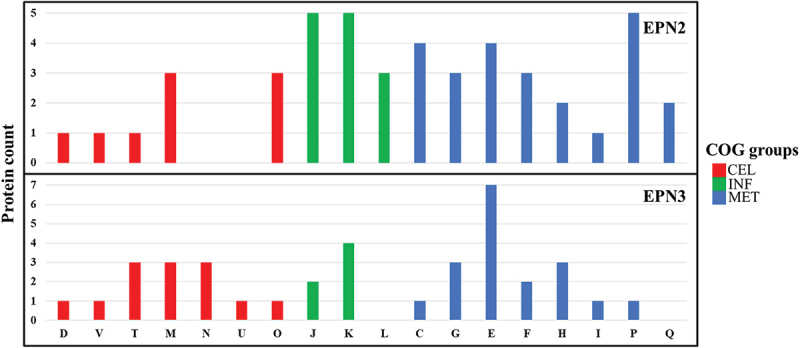
Protein expression patterns and functional distribution in *P. mirabilis* ATCC 7002 after CuO/Cu₂O NP treatment. cel, cellular processes and signaling; inf, Information storage and processing; met, metabolism. COG categories are as defined in Figure 5.

A total of nine significant EPNs were clustered in CuO/Cu₂O NP-treated *P. mirabilis* (Table S8). In the EPN2 dataset, which included 54 proteins, a consistent upregulation was seen throughout the treatment period (Figure 15, Table S13). Notably, proteins involved in Translation, ribosomal structure and biogenesis (J), Transcription (K), and Inorganic ion transport and metabolism (P) exhibited the most significant upregulation. In contrast, the EPN3 dataset included 40 proteins that consistently showed downregulation (Figure 15, Table S14). Among these downregulated proteins, those associated with Amino acid transport and metabolism (E) and Transcription (K) exhibited the most pronounced decreases in expression levels.

Twenty-six differentially expressed proteins associated with virulence were identified in *P. mirabilis* following CuO/Cu₂O NP treatment (Table 2). These included proteins involved in motility (CheR, FliC, FlgI, FlgK, FliM, HyfR, KdsB), immune modulation (GalE, LpxB, Pgi, RfaC, RffH, Ugd), nutrient acquisition (CarA, HmuR), and stress response (ClpP, RpoS, UreC). Notably, CheR, ClpV, and LpxB exhibited consistent upregulation, while FliC, FlgI, and TssQ_1 were consistently downregulated throughout the time course of CuO/Cu₂O NP treatment.

**Table 2.** Differentially expressed proteins associated with virulence in *P. mirabilis* ATCC 7002 following CuO/Cu₂O NP treatment.

				Fold ratio	
Protein ID	Protein name	Gene name	COG	10 min	30 min
KGA88983.1	Lipid-A-disaccharide synthase	lpxB	M	4.94	5.14
KGA90250.1	Type VI secretion ATPase, ClpV1 family	clpV	O	1.41	2.73
KGA91183.1	Chemotaxis protein methyltransferase	cheR	H	9.97	9.97
KGA90754.1	Flagellin 1	fliC	N	−1.14	−1.34
KGA90908.1	Flagellin 1	fliC	N	−1.29	−1.11
KGA91119.1	Flagellar P-ring family protein	flgI	T	−1.64	−1.53
KGA91580.1	Type VI secretion system effector, Hcp1 family protein	tssQ_1	S	−1.21	−1.01
KGA90291.1	Carbon storage regulator	csrA	J	−1.57	0.81
KGA91468.1	Carbamoyl-phosphate synthase small chain	carA	F	−1.00	−0.20
KGA91697.1	Urease, alpha subunit	ureC	E	−6.39	−0.03
KGA92484.1	Lipopolysaccharide heptosyltransferase I	rfaC	M	−2.35	−0.22
KGA89213.1	Glucose-6-phosphate isomerase	pgi	F	1.34	−0.19
KGA90076.1	3-Deoxy-D-manno-octulosonate cytidylyltransferase	kdsB	F	1.70	0.72
KGA90462.1	AAA domain family protein	hyfR	KT	1.45	0.11
KGA90503.1	UDP-glucose 4-epimerase	galE	M	1.64	0.20
KGA91159.1	TonB-dependent hemoglobin/transferrin/lactoferrin receptor family protein	hmuR	P	1.73	0.13
KGA91642.1	Acriflavine resistance protein B	acrB	V	1.26	0.28
KGA91807.1	ATP-dependent Clp endopeptidase, proteolytic subunit	clpP	O	1.12	−0.34
KGA92101.1	Glucose-1-phosphate thymidylyltransferase	rffH	H	1.28	0.67
KGA92502.1	UDP-glucose 6-dehydrogenase	ugd	C	1.10	−0.26
KGA90657.1	Aldehyde-alcohol dehydrogenase	adhE	C	0.08	−1.02
KGA90664.1	RTX toxin RtxA	rtxA	MQ	−0.34	−1.59
KGA90582.1	Flagellar motor switch protein	fliM	N	0.86	2.07
KGA90976.1	Flagellar hook-associated protein	flgK	N	0.63	1.29
KGA91385.1	RNA polymerase sigma factor RpoS	rpoS	K	0.87	2.24
KGA92257.1	DNA-binding response regulator	yehT	KT	0.35	4.01

## 4. Discussion

This study investigated the antibacterial activity of CuO/Cu₂O NPs against a panel of clinically relevant urinary pathogens and explored the molecular mechanisms underlying bacterial responses to CuO/Cu₂O NP stress. Our findings demonstrated substantial reductions in bacterial cell counts across all tested species, with complete inhibition of *P. aeruginosa* and *E. coli* after 30 min of exposure, while *E. faecalis*, a Gram-positive bacterium, showed greater resistance. These results align with previous studies, such as those by Shehabeldine et al., which reported higher susceptibility of Gram-negative *Klebsiella oxytoca* and *E. coli* to copper NPs, whereas Gram-positive *S. aureus* and *B. cereus* exhibited higher minimum inhibitory concentration (MIC) values [17]. Similarly, other studies have highlighted the superior antibacterial effects of CuO/Cu₂O NPs against Gram-negative bacteria, particularly *E. coli* and *P. aeruginosa*, compared to Gram-positive species [26]. This increased susceptibility in Gram-negative bacteria is likely due to their thinner and less complex peptidoglycan layer, which facilitates nanoparticle penetration and internalization [26–28].

To further understand the bacterial response to CuO/Cu₂O NPstress, we conducted a comparative analysis of the adaptive mechanisms in *E. faecalis* and *P. mirabilis*. The global proteomic data revealed both shared and distinct metabolic adaptations. Commonly upregulated COGs in both bacteria included Energy production and conversion (C), Amino acid transport and metabolism (E), Transcription (K), and Coenzyme transport and metabolism (H), suggesting conserved strategies to maintain metabolic homeostasis. This aligns with previous findings where similar increases in energy production and transcription-related proteins were reported in bacteria exposed to silver and zinc oxide NPs [29,30]. The increased activity in these pathways likely reflects heightened energy demands necessary for cellular repair and stress adaptation, as well as transcriptional activation of genes involved in detoxification and metabolic reprogramming.

Despite these shared responses, species-specific adaptations were evident. In *E. faecalis*, the overexpression of Carbohydrate transport and metabolism (G), Cell wall/membrane/envelope biogenesis (M), and Coenzyme transport and metabolism (H) indicates an enhanced ability to metabolize carbohydrates, reinforce cell envelope integrity, and support enzymatic functions. The increased carbohydrate metabolism may provide additional energy to sustain stress-related pathways, a phenomenon also observed in bacteria exposed to antimicrobial peptides [31,32]. Upregulation of cell wall biogenesis suggests development of a crucial defense mechanism to counter CuO/Cu₂O NP-induced damage, consistent with bacterial adaptations to metal stress, which often involves remodeling of the cell envelope to limit toxic ion penetration [33].

In contrast, *P. mirabilis* showed distinct upregulation in Translation, ribosomal structure and biogenesis (J), Inorganic ion transport and metabolism (P), and Nucleotide transport and metabolism (F). The observed increase in ribosomal biogenesis aligns with findings in *Streptococcus suis* exposed to metal NPs [29] and likely reflects an increased demand for protein synthesis to replace damaged cellular components. Upregulation of inorganic ion transport pathways, a response also reported in *P. aeruginosa* under copper stress [34], suggests active regulation of ion homeostasis to mitigate toxicity. This mechanism is crucial for maintaining intracellular ionic balance and expelling excess heavy metal ions, thereby preventing their accumulation to toxic levels [35].

Both *E. faecalis* and *P. mirabilis* exhibited significant changes in metabolic pathways as identified through KEGG pathway analysis. Metabolic processes such as Metabolic pathways, Biosynthesis of secondary metabolites, Biosynthesis of cofactors, and Microbial metabolism in diverse environments were highly affected in both bacteria, with proteins in these pathways showing both upregulation and downregulation. Among the most prominently overexpressed pathways in both species were ABC transporters within the Membrane transport category and Quorum sensing within the Cellular community category. ABC transporters are critical for bacterial survival under stress conditions, using ATP hydrolysis to drive the transport of essential nutrients, efflux of toxins, and maintenance of homeostasis [36]. Their significant upregulation in *E. faecalis* and *P. mirabilis* suggests an essential role in bacterial adaptation to CuO/Cu₂O NP exposure by facilitating the removal of toxic metal ions and stress-induced metabolites. Notably, in *E. faecalis*, 26 proteins associated with ABC transporters were significantly upregulated after 30 min of CuO/Cu₂O NP exposure, including permease and substrate-binding proteins such as OpuBD, LplB, LplC, BlpA, MetQ, MsmX, OppA, CycB, and LplA (Tables S1, S3). The role of ABC transporters in bacterial stress responses is well documented across various species, further reinforcing their conserved function in coping with environmental stressors [37–43].

Quorum sensing (QS), a bacterial communication system that coordinates gene expression based on population density [44], was also significantly upregulated in both *E. faecalis* and *P. mirabilis*. Proteins such as OppA, OppC, BlpA, SecY, GlrK, and Zur exhibited increased expression, suggesting that bacterial populations may activate QS pathways to enhance survival under Cu NP stress (Tables S1, S3). This finding aligns with studies reporting QS activation in bacteria exposed to metals, nanomaterials, and other environmental stressors [45–49]. QS may play a critical role in bacterial adaptation by modulating virulence factor expression, biofilm formation, and stress tolerance mechanisms.

The phosphotransferase system (PTS), the primary sugar transport mechanism in many Gram-positive bacteria [50], was specifically upregulated in *E. faecalis*. After 30 min of CuO/Cu₂O NP treatment, 16 PTS-related proteins were upregulated, including GatA, GatB, SrlB, ManX, UlaB, DgaB, FruK, and CelB (Tables S1, S3). This increased expression suggests that *E. faecalis* enhances sugar uptake to sustain metabolic activity and energy production under NP-induced stress. The upregulation of PTS proteins under various stressors has been previously observed in other Gram-positive bacteria, supporting its role in bacterial stress responses [51–55].

Ribosomes play a central role in bacterial growth and proliferation by translating genetic code and driving protein synthesis, one of the most energy-intensive cellular processes [56]. In *P. mirabilis*, exposure to CuO/Cu₂O NPs led to the significant upregulation of 20 proteins associated with the Ribosome pathway (Tables S8, S10). Among these, seven belonged to the Rps family of 30S ribosomal proteins, while 13 were part of the Rpl family of 50S ribosomal proteins. The upregulation of these proteins suggests an increased demand for ribosomal function, likely supporting enhanced protein synthesis necessary for cellular repair and adaptation under NP-induced stress. The observed upregulation of ribosomal proteins in *P. mirabilis* is consistent with previous studies that have linked ribosome-related responses to various environmental stressors.

KEGG pathway analysis revealed that exposure to slightly acidic electrolyzed water combined with high-pressure processing led to the overexpression of ribosomal proteins in *L. monocytogenes* [57], mirroring findings by Bowman et al. [58], where high-pressure processing induced the upregulation of ribosomal genes. Similarly, cold temperatures have been shown to induce ribosome protein upregulation in lactic acid bacteria [59]. Additionally, increased expression of ribosome-related proteins has been observed in *Staphylococcus epidermidis* following antibiotic treatment and in *Lacticaseibacillus rhamnosus* under acid and osmotic stress [3,46,60]. These observations suggest that the upregulation of ribosomal proteins may represent a common adaptive response to various stress conditions, including antimicrobial challenges.

The two-component system (TCS), a bacterial signaling mechanism that enables environmental stress detection and adaptive gene regulation [61], was notably upregulated in *P. mirabilis* in response to CuO/Cu₂O NP exposure. Key TCS-related proteins, including FrdA, FrdC, CydA, CydB, CheR, GlnD, GlrK, and CpxA, were differentially expressed, suggesting a reliance on TCS pathways to cope with CuO/Cu₂O NP stress (Tables S8, S10). These findings are consistent with previous studies reporting TCS activation in *Campylobacter jejuni* following chloramphenicol exposure [62] and in *Bacillus subtilis* under ultrasound stress [63], reinforcing the importance of TCS in bacterial survival and adaptation.

Finally, the differential expression of virulence-associated proteins in *E. faecalis* and *P. mirabilis* suggests a complex bacterial stress response to CuO/Cu₂O NP exposure. In *E. faecalis*, proteins associated with adherence, exotoxin production, nutrient acquisition, immune modulation, and stress survival were differentially regulated; whereas *P. mirabilis* exhibited changes in proteins involved in motility, immune modulation, nutrient acquisition, and stress response. These findings align with previous studies demonstrating that various stressors, including antibiotics and nutrient limitations, can alter virulence factor expression in bacterial pathogens [64–70]. The interplay between stress adaptation and virulence regulation highlights the need for further research to elucidate how CuO/Cu₂O NP exposure influences bacterial pathogenicity.

The stronger antibacterial effects observed against *E. coli* and *P. aeruginosa* compared with *P. mirabilis* can be attributed to species-specific structural and physiological differences. Both *E. coli* and *P. aeruginosa* possess relatively permeable outer membranes and surface charge properties that favor nanoparticle association and permeabilization, facilitating Cu(I/II) ion entry and diffusion. This leads to a faster onset of ROS generation, envelope stress, and membrane rupture. In contrast, *P. mirabilis* displayed proteomic adaptations emphasizing ribosome biogenesis and protein replacement, two-component signaling, and inorganic ion transport/homeostasis, consistent with efficient copper handling, detoxification, and damage repair. Additional resistance mechanisms in *P. mirabilis*, including robust ion transport systems, urease-mediated pH modulation, and upregulation of stress-response proteins, likely confer improved ability to neutralize or detoxify copper ions. These adaptations align with the observed slower killing kinetics, FESEM morphologies, and downshift in motility components, suggesting that ion homeostasis and envelope remodeling are key determinants of *P. mirabilis*’ comparatively resilient phenotype under CuO/Cu₂O NP exposure.

**Figure 16.**
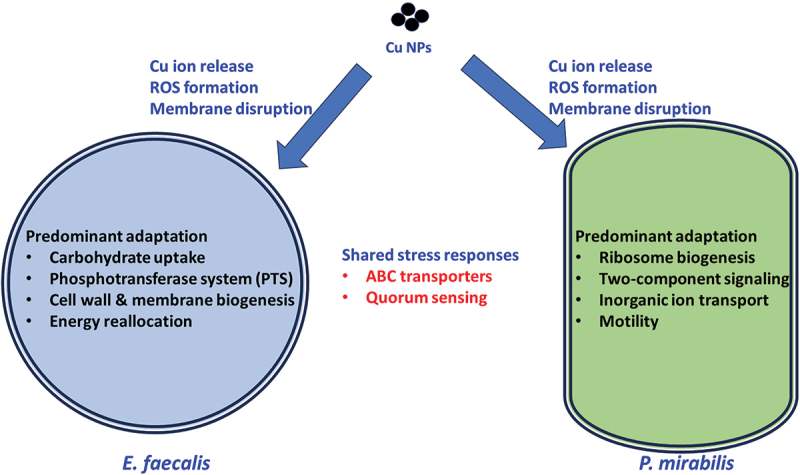
Proposed antibacterial mechanisms of CuO/Cu₂O NPs in *E. faecalis* and *P. mirabilis*.

Integrating rapid (10–60 min) bactericidal effects (Figure 1), FESEM evidence of envelope damage (Figure 3), and quantitative proteomics (Figures. 4–15), we propose the model summarized in Figure 16. Shared early events include Cu(I/II) ion release, ROS generation, and membrane disruption, which activate conserved stress programs (e.g., ABC transporters, quorum sensing). In Gram‑positive *E. faecalis*, predominant adaptations involve carbohydrate uptake/PTS and cell‑wall/membrane biogenesis, consistent with energy reallocation and envelope fortification under metal stress. In Gram‑negative *P. mirabilis*, responses emphasize ribosome biogenesis/protein synthesis, two‑component signaling, and inorganic‑ion transport/homeostasis, consistent with rapid replacement of damaged proteins and ion detoxification; motility components (e.g., FliC/FlgI) are comparatively downregulated. These species‑resolved programs likely underlie the observed differences in killing kinetics and FESEM morphologies under CuO/Cu₂O nanoparticle exposure.

## 5. Conclusion

Using time-kill assays, FESEM imaging, and DIA-based quantitative proteomics, we characterized the early (10–60 min) antibacterial responses of urinary pathogens to PLAL-generated CuO/Cu₂O NPs and resolved species-specific adaptations in *E. faecalis* and *P. mirabilis*. Across the four pathogens tested, *P. aeruginosa* and *E. coli* exhibited the highest susceptibility, while *P. mirabilis* showed relative resistance. FESEM analysis confirmed substantial nanoparticle-induced envelope disruption, whereas proteomic profiling revealed both conserved and species-specific stress adaptations. Conserved responses included Cu ion release, ROS generation, and membrane damage, which activated shared stress programs such as ABC transporters and quorum sensing pathways. In contrast, *E. faecalis* primarily reallocated resources toward carbohydrate uptake and cell wall biogenesis, reflecting energy reallocation and envelope fortification, while *P. mirabilis* emphasized ribosome biogenesis, two-component signaling, and inorganic ion transport/homeostasis, consistent with rapid protein replacement, ion detoxification, and motility downregulation. Together, these findings provide mechanistic insights into how urinary pathogens respond to copper nanoparticle exposure and highlight ion homeostasis and envelope remodeling as key determinants of susceptibility. They also underscore the potential of CuO/Cu₂O NPs as a foundation for alternative antimicrobial strategies against multidrug-resistant infections. Limitations of this study include the absence of soluble copper salt controls to distinguish ionic versus nanoparticle effects and the restriction of proteomic analysis to two species and early time windows. Future studies should extend analyses to additional strains, disentangle ionic and particulate effects, and evaluate pathogen responses in in situ biofilm models.

## Supplementary Material

Supplementary Table.docx

## Article highlights

- Copper oxide nanoparticles (CuO/Cu₂O), synthesized by pulsed laser ablation in liquid (PLAL), showed rapid bactericidal activity against four clinically relevant urinary pathogens, with *E. coli* and *P. aeruginosa* being highly susceptible and *P. mirabilis* more resistant.
- FESEM imaging confirmed substantial nanoparticle-induced structural damage, including envelope disruption and cell deformation, in treated bacteria compared to untreated controls.
- Quantitative proteomics revealed time-dependent and species-specific responses, with *E. faecalis* upregulating carbohydrate transport and cell wall biogenesis pathways, while *P. mirabilis* emphasized ribosome biogenesis, two-component signaling, and ion homeostasis.
- Conserved bacterial adaptations included activation of ABC transporters, quorum sensing pathways, and energy metabolism, whereas species-specific pathways reflected Gram-positive versus Gram-negative strategies to withstand copper nanoparticle stress.
- Proteomic analyses also showed differential regulation of virulence-associated proteins, suggesting that CuO/Cu₂O nanoparticles may impact not only viability but also pathogenic potential.
- Together, these findings support a mechanistic model involving Cu ion release, ROS generation, and membrane disruption as shared antibacterial triggers, modulated by species-specific adaptations in ion balance and envelope remodeling.
- This work highlights the potential of CuO/Cu₂O nanoparticles as alternative antimicrobials against multidrug-resistant urinary pathogens, while noting study limitations such as the absence of soluble copper salt controls, focus on two species for proteomics, and early exposure time windows.
