# Supplementary material for: Quantitative proteomic and phenotypic responses of urinary pathogens to CuO/Cu₂O nanoparticles
Source: Nanomedicine (Lond). 2025 Oct 29;20(24):2917–34. doi: 10.1080/17435889.2025.2579616 (PMC12710891; doi:10.1080/17435889.2025.2579616)
Supplement: Supplementary Table.docx [file INNM_A_2579616_SM5357.docx]

**Table. S1**. Proteins identified from *E. faecalis* ATCC 29212 following copper NP treatment.

| **Protein ID** |  | **Gene name** | **Protein description** | **Fold Ratio** | | | **Expression Pattern** | | | **EP group** | **COG** | **KEGG number** |
| --- | --- | --- | --- | --- | --- | --- | --- | --- | --- | --- | --- | --- |
|  |  |  |  | **10 min** | **30 min** | **60 min** | **10 min** | **30 min** | **60 min** |  |  |  |
| AIL03101.1 |  | - | hypothetical protein DR75_2991 (plasmid) | 0.86 | 0.16 | -0.16 | NC | NC | NC | 1 | - | - |
| AIL03102.1 |  | - | replication initiator A family protein (plasmid) | -0.31 | 0.18 | -0.14 | NC | NC | NC | 1 | - | - |
| AIL03104.1 |  | - | hypothetical protein DR75_2992 (plasmid) | 0.66 | 0.24 | 0.21 | NC | NC | NC | 1 | - | - |
| AIL03106.1 |  | - | peptidase S41 family protein (plasmid) | 0.34 | -0.03 | 0.21 | NC | NC | NC | 1 | - | - |
| AIL03107.1 |  | arsR | bacterial regulatory, arsR family protein (plasmid) | -0.01 | -0.19 | -0.11 | NC | NC | NC | 1 | K | ko:K03892 |
| AIL03111.1 |  | lgt | prolipoprotein diacylglyceryl transferase (plasmid) | -0.36 | -0.41 | 0.22 | NC | NC | NC | 1 | M | ko:K13292 |
| AIL03113.1 |  | - | putative replication-associated protein (plasmid) | 0.12 | -0.29 | -0.34 | NC | NC | NC | 1 | - | - |
| AIL03114.1 |  | - | addiction module antitoxin, RelB/DinJ family protein (plasmid) | 0.74 | 0.84 | 1.36 | NC | NC | U | 5 | L | ko:K07473 |
| AIL03115.1 |  | - | addiction module toxin, RelE/StbE family protein (plasmid) | 0.53 | -0.31 | -0.27 | NC | NC | NC | 1 | S | ko:K19157 |
| AIL03116.1 |  | merA | mercuric reductase (plasmid) | 0.94 | 0.04 | 0.03 | NC | NC | NC | 1 | C | ko:K00520 |
| AIL03118.1 |  | - | bacterial regulatory, arsR family protein (plasmid) | 0.17 | -0.26 | -0.69 | NC | NC | NC | 1 | K | ko:K03892 |
| AIL03120.1 |  | arsA | arsenical pump-driving ATPase (plasmid) | 0.10 | -0.44 | -0.24 | NC | NC | NC | 1 | D | ko:K01551 |
| AIL03122.1 |  | - | putative transposon DNA-invertase bin3 | 0.44 | -1.47 | -1.20 | NC | D | D | 22 | - | - |
| AIL03123.1 |  | - | lactococcin-G-processing and transport ATP-binding protein LagD (plasmid) | 0.01 | -0.42 | -0.18 | NC | NC | NC | 1 | V | ko:K20344 |
| AIL03125.1 |  | - | ASCH domain protein (plasmid) | 0.79 | 0.79 | -0.50 | NC | NC | NC | 1 | S | - |
| AIL03126.1 |  | - | putative replication control protein PrgN (plasmid) | 0.22 | 0.20 | 0.18 | NC | NC | NC | 1 | - | - |
| AIL03130.1 |  | repA | putative prgW (plasmid) | -0.74 | -0.68 | -0.60 | NC | NC | NC | 1 | S | - |
| AIL03131.1 |  | soj | cobQ/CobB/MinD/ParA nucleotide binding domain protein (plasmid) | 0.00 | -0.34 | -0.14 | NC | NC | NC | 1 | D | - |
| AIL03135.1 |  | - | arsenical resistance operon trans-acting repressor ArsD (plasmid) | 0.84 | -0.17 | -0.21 | NC | NC | NC | 1 | S | - |
| AIL03136.1 |  | - | Hg(II)-responsive transcriptional regulator (plasmid) | 0.86 | -0.08 | 0.27 | NC | NC | NC | 1 | K | - |
| AIL03148.1 |  | - | hypothetical protein DR75_2897 (plasmid) | -0.38 | -0.92 | -0.59 | NC | NC | NC | 1 | L | - |
| AIL03149.1 |  | - | thioredoxin family protein (plasmid) | -1.75 | 0.89 | 0.21 | D | NC | NC | 6 | - | - |
| AIL03153.1 |  | - | hypothetical protein DR75_2888 (plasmid) | -0.49 | -0.02 | -0.07 | NC | NC | NC | 1 | KLT | ko:K00703 |
| AIL03157.1 |  | - | fic/DOC family protein (plasmid) | 0.17 | 0.13 | 0.10 | NC | NC | NC | 1 | D | - |
| AIL03158.1 |  | traB | TraB family protein (plasmid) | 0.90 | -0.12 | -0.30 | NC | NC | NC | 1 | S | - |
| AIL03160.1 |  | - | phoH-like family protein (plasmid) | 0.37 | 0.53 | 0.32 | NC | NC | NC | 1 | L | - |
| AIL03161.1 |  | - | hypothetical protein DR75_2884 (plasmid) | 1.04 | 0.28 | -0.33 | U | NC | NC | 11 | - | - |
| AIL03174.1 |  | - | hypothetical protein DR75_2906 (plasmid) | 0.09 | -0.28 | -0.54 | NC | NC | NC | 1 | - | - |
| AIL03175.1 |  | - | ABC transporter transmembrane region family protein (plasmid) | -1.19 | 1.20 | 1.20 | D | U | U | 21 | V | ko:K20344 |
| AIL03178.1 |  | - | ABC transporter family protein (plasmid) | 2.47 | 1.46 | 1.80 | U | U | U | 2 | V | ko:K06148 |
| AIL03179.1 |  | - | putative nucleotidyltransferase (plasmid) | 0.25 | 0.06 | 0.17 | NC | NC | NC | 1 | J | - |
| AIL03180.1 |  | - | putative uvrC (plasmid) | -0.01 | -0.45 | -0.03 | NC | NC | NC | 1 | - | - |
| AIL03181.1 |  | - | hypothetical protein DR75_2890 (plasmid) | 0.41 | 0.75 | 0.80 | NC | NC | NC | 1 | - | - |
| AIL03183.1 |  | - | subtilase family protein (plasmid) | -1.00 | -1.21 | -1.31 | D | D | D | 3 | O | ko:K20486 |
| AIL03187.1 |  | ssb | single-stranded DNA-binding family protein (plasmid) | -0.16 | -0.28 | -0.53 | NC | NC | NC | 1 | L | ko:K03111 |
| AIL03189.1 |  | salB | type 2 lantibiotic biosynthesis LanM family protein (plasmid) | -0.45 | 0.10 | 0.27 | NC | NC | NC | 1 | V | ko:K20385 |
| AIL03190.1 |  | - | type 2 lantibiotic, family protein (plasmid) | 0.10 | -1.54 | -1.31 | NC | D | D | 22 | S | ko:K20384 |
| AIL03195.1 |  | - | hypothetical protein DR75_2891 (plasmid) | 0.00 | 0.19 | 0.27 | NC | NC | NC | 1 | - | - |
| AIL03196.1 |  | - | putative lipoprotein (plasmid) | 0.06 | -0.53 | -0.10 | NC | NC | NC | 1 | CO | - |
| AIL03200.1 |  | - | cobQ/CobB/MinD/ParA nucleotide binding domain protein (plasmid) | -0.30 | -0.18 | -0.03 | NC | NC | NC | 1 | D | - |
| AIL03202.1 |  | lanT | ABC transporter family protein (plasmid) | -0.22 | 0.17 | -0.34 | NC | NC | NC | 1 | V | ko:K06148 |
| AIL03203.1 |  | - | hypothetical protein DR75_2901 (plasmid) | -0.87 | -1.18 | -0.63 | NC | D | NC | 23 | - | - |
| AIL03205.1 |  | - | glycosyl transferase 2 family protein (plasmid) | 0.46 | 1.56 | 1.39 | NC | U | U | 4 | M | - |
| AIL03207.1 |  | - | putative cylI (plasmid) | -0.40 | -0.23 | -0.18 | NC | NC | NC | 1 | S | - |
| AIL03215.1 |  | panD | aspartate 1-decarboxylase | -0.44 | -0.35 | -0.38 | NC | NC | NC | 1 | H | ko:K01579 |
| AIL03218.1 |  | gap | glyceraldehyde-3-phosphate dehydrogenase, type I | 1.25 | 0.77 | 0.95 | U | NC | NC | 11 | G | ko:K00134 |
| AIL03219.1 |  | pstA | phosphate ABC transporter, permease protein PstA | -0.55 | -0.41 | -0.35 | NC | NC | NC | 1 | P | ko:K02038 |
| AIL03223.1 |  | purA | adenylosuccinate synthase | -0.05 | -0.06 | -0.08 | NC | NC | NC | 1 | F | ko:K01939 |
| AIL03224.1 |  | - | hypothetical protein DR75_834 | 1.19 | -0.38 | -0.08 | U | NC | NC | 11 | K | - |
| AIL03225.1 |  | mutL | DNA mismatch repair MutL family protein | 0.22 | -0.27 | -0.10 | NC | NC | NC | 1 | L | ko:K03572 |
| AIL03226.1 |  | sigA | RNA polymerase sigma factor RpoD | 0.11 | -0.15 | -0.17 | NC | NC | NC | 1 | K | ko:K03086 |
| AIL03230.1 |  | murA | UDP-N-acetylglucosamine 1-carboxyvinyltransferase | 0.00 | -0.35 | -0.29 | NC | NC | NC | 1 | M | ko:K00790 |
| AIL03231.1 |  | fhs | formate--tetrahydrofolate ligase family protein | -0.09 | 0.17 | 0.10 | NC | NC | NC | 1 | F | ko:K01938 |
| AIL03232.1 |  | guaB | inosine-5'-monophosphate dehydrogenase | 0.08 | -0.42 | -0.32 | NC | NC | NC | 1 | F | ko:K00088 |
| AIL03235.1 |  | rdrB | hypothetical protein DR75_2604 | 0.15 | 0.08 | -0.78 | NC | NC | NC | 1 | K | ko:K02444 |
| AIL03242.1 |  | rseP | RIP metalloprotease RseP | -0.19 | -0.14 | -0.23 | NC | NC | NC | 1 | M | ko:K11749 |
| AIL03243.1 |  | cydD | thiol reductant ABC exporter, CydC subunit | -0.09 | -0.35 | -0.41 | NC | NC | NC | 1 | CO | ko:K16012 |
| AIL03244.1 |  | - | glycosyl transferase 2 family protein | 0.08 | 0.10 | 0.00 | NC | NC | NC | 1 | S | ko:K07011 |
| AIL03245.1 |  | tetR3 | bacterial regulatory s, tetR family protein | 0.79 | 2.05 | 1.61 | NC | U | U | 4 | K | ko:K18476 |
| AIL03248.1 |  | ytoI | thioesterase superfamily protein | -0.12 | 0.20 | 0.00 | NC | NC | NC | 1 | K | - |
| AIL03250.1 |  | malE | bacterial extracellular solute-binding family protein | 1.79 | 2.13 | 1.81 | U | U | U | 2 | G | ko:K15770 |
| AIL03252.1 |  | rplJ | ribosomal L10 family protein | -0.23 | -0.19 | -0.31 | NC | NC | NC | 1 | J | ko:K02864 |
| AIL03254.1 |  | yabO | S4 domain protein | 0.72 | -0.18 | -0.25 | NC | NC | NC | 1 | J | - |
| AIL03256.1 |  | - | mga helix-turn-helix domain protein | 0.91 | 0.34 | 0.41 | NC | NC | NC | 1 | K | - |
| AIL03257.1 |  | ydiF | heme ABC exporter, ATP-binding protein CcmA | -0.11 | -0.21 | 0.11 | NC | NC | NC | 1 | S | ko:K06158 |
| AIL03258.1 |  | ycaM | inner membrane transporter ycaM | -0.27 | -0.47 | -0.52 | NC | NC | NC | 1 | E | - |
| AIL03259.1 |  | yhaN | AAA domain protein | 0.64 | 0.79 | 0.52 | NC | NC | NC | 1 | L | - |
| AIL03260.1 |  | - | glycosyl transferase 2 family protein | -0.26 | -0.03 | 0.00 | NC | NC | NC | 1 | M | ko:K20444 |
| AIL03262.1 |  | nusB | transcription antitermination factor NusB | 0.13 | 0.09 | 0.05 | NC | NC | NC | 1 | K | ko:K03625 |
| AIL03264.1 |  | - | bacterial PH domain protein | -0.27 | -0.37 | -0.37 | NC | NC | NC | 1 | S | - |
| AIL03265.1 |  | pflB | formate acetyltransferase | -0.48 | -1.03 | -0.59 | NC | D | NC | 23 | C | ko:K00656 |
| AIL03266.1 |  | rpsH | 30S ribosomal protein S8 | -0.25 | -0.27 | -0.37 | NC | NC | NC | 1 | J | ko:K02994 |
| AIL03267.1 |  | yrvD | hypothetical protein DR75_690 | -0.18 | -0.29 | -0.21 | NC | NC | NC | 1 | S | - |
| AIL03269.1 |  | cdd | cytidine deaminase | 0.41 | 0.35 | 0.23 | NC | NC | NC | 1 | F | ko:K01489 |
| AIL03270.1 |  | ptb | branched-chain phosphotransacylase | -0.19 | 1.78 | 1.74 | NC | U | U | 4 | C | ko:K00634 |
| AIL03271.1 |  | ribU | hypothetical protein DR75_545 | -0.97 | -0.60 | -0.91 | NC | NC | NC | 1 | U | - |
| AIL03272.1 |  | rpsI | ribosomal S9/S16 family protein | -0.28 | 0.14 | -0.20 | NC | NC | NC | 1 | J | ko:K02996 |
| AIL03274.1 |  | hlyX | transporter associated domain protein | -0.51 | -0.36 | -0.35 | NC | NC | NC | 1 | S | ko:K03699 |
| AIL03275.1 |  | nnrD | yjeF-related protein | 0.24 | 0.03 | 0.00 | NC | NC | NC | 1 | H | ko:K17758 |
| AIL03276.1 |  | ytgP | polysaccharide biosynthesis family protein | -0.09 | -0.81 | 0.37 | NC | NC | NC | 1 | S | ko:K03328 |
| AIL03277.1 |  | rumA_2 | 23S rRNA (uracil-5-)-methyltransferase RumA | -0.13 | -0.31 | -0.13 | NC | NC | NC | 1 | J | ko:K03215 |
| AIL03278.1 |  | yeeN | DNA-binding regulatory, YebC/PmpR family protein | -0.30 | -0.68 | -0.55 | NC | NC | NC | 1 | K | - |
| AIL03279.1 |  | trxB | thioredoxin-disulfide reductase | 0.22 | 0.07 | -0.18 | NC | NC | NC | 1 | C | ko:K00384 |
| AIL03282.1 |  | ybcH | dienelactone hydrolase family protein | -0.54 | -0.14 | -0.22 | NC | NC | NC | 1 | S | ko:K06889 |
| AIL03284.1 |  | pepA | glutamyl aminopeptidase | 0.44 | 0.12 | 0.08 | NC | NC | NC | 1 | E | ko:K01261 |
| AIL03285.1 |  | strH | glycosyl hydrolase family 20, catalytic domain protein | 0.04 | 0.08 | 0.35 | NC | NC | NC | 1 | G | ko:K12373 |
| AIL03287.1 |  | queT | queT transporter family protein | 0.20 | -0.37 | -0.21 | NC | NC | NC | 1 | S | - |
| AIL03288.1 |  | prfB | peptide chain release factor 2 | -0.29 | -0.40 | -0.34 | NC | NC | NC | 1 | J | ko:K02836 |
| AIL03289.1 |  | vicX | metallo-beta-lactamase superfamily protein | -0.28 | -0.11 | -0.53 | NC | NC | NC | 1 | S | ko:K00784 |
| AIL03290.1 |  | frdC | flavocytochrome c family protein | -0.21 | -0.22 | -0.23 | NC | NC | NC | 1 | C | ko:K00239 |
| AIL03291.1 |  | - | PTS system fructose IIA component family protein | -0.47 | -0.14 | -0.43 | NC | NC | NC | 1 | G | ko:K02793 |
| AIL03292.1 |  | - | hypothetical protein DR75_2452 | -0.38 | 0.05 | -0.34 | NC | NC | NC | 1 | S | - |
| AIL03293.1 |  | - | general stress protein A | -0.10 | -0.64 | -0.04 | NC | NC | NC | 1 | M | - |
| AIL03294.1 |  | - | antibiotic biosynthesis monooxygenase family protein | -0.33 | 0.18 | -0.16 | NC | NC | NC | 1 | S | - |
| AIL03295.1 |  | ykuT | mechanosensitive ion channel family protein | -0.26 | -0.02 | -0.12 | NC | NC | NC | 1 | M | ko:K16052 |
| AIL03296.1 |  | hpf | ribosomal subunit interface protein | 0.62 | 0.39 | 0.31 | NC | NC | NC | 1 | J | ko:K05808 |
| AIL03297.1 |  | - | hypothetical protein DR75_900 | 0.20 | 0.07 | -0.16 | NC | NC | NC | 1 | - | ko:K02029 |
| AIL03301.1 |  | nifJ | ferredoxin (flavodoxin) oxidoreductase | 0.48 | -2.55 | 1.44 | NC | D | U | 7 | C | ko:K03737 |
| AIL03303.1 |  | feoA | feoA domain protein | -0.34 | 0.41 | -0.02 | NC | NC | NC | 1 | P | ko:K04758 |
| AIL03305.1 |  | - | hypothetical protein DR75_1624 | -0.14 | -0.72 | -0.40 | NC | NC | NC | 1 | - | - |
| AIL03306.1 |  | ohrA | organic hydroperoxide resistance protein ohrB | 1.32 | 0.66 | 0.55 | U | NC | NC | 11 | O | - |
| AIL03308.1 |  | rpsG | ribosomal protein S7 | -0.40 | -0.47 | -0.86 | NC | NC | NC | 1 | J | ko:K02992 |
| AIL03309.1 |  | - | HTH domain protein | 0.53 | -0.28 | 0.95 | NC | NC | NC | 1 | S | - |
| AIL03310.1 |  | - | helix-turn-helix family protein | -0.19 | 0.28 | -0.05 | NC | NC | NC | 1 | K | - |
| AIL03311.1 |  | napA | transporter, monovalent cation:proton antiporter-2 family protein | 0.28 | 0.22 | 0.07 | NC | NC | NC | 1 | P | - |
| AIL03312.1 |  | engB | ribosome biogenesis GTP-binding protein YsxC | -0.11 | -0.20 | -0.12 | NC | NC | NC | 1 | D | ko:K03978 |
| AIL03313.1 |  | clpX | ATP-dependent Clp protease, ATP-binding subunit ClpX | -0.20 | -0.27 | -0.37 | NC | NC | NC | 1 | O | ko:K03544 |
| AIL03314.1 |  | def | peptide deformylase | 0.54 | 0.14 | 0.21 | NC | NC | NC | 1 | J | ko:K01462 |
| AIL03315.1 |  | lspA | signal peptidase II | 0.20 | -0.13 | 0.08 | NC | NC | NC | 1 | MU | ko:K03101 |
| AIL03316.1 |  | - | ecsC family protein | -2.37 | 0.77 | -2.51 | D | NC | D | 8 | - | - |
| AIL03317.1 |  | rnjA | hypothetical protein DR75_1618 | 0.08 | -0.28 | -0.16 | NC | NC | NC | 1 | J | ko:K12574 |
| AIL03318.1 |  | gyrA | DNA gyrase, A subunit | -0.12 | -0.86 | 0.26 | NC | NC | NC | 1 | L | ko:K02469 |
| AIL03319.1 |  | murD | UDP-N-acetylmuramoylalanine--D-glutamate ligase | -0.42 | -0.12 | -0.11 | NC | NC | NC | 1 | M | ko:K01925 |
| AIL03320.1 |  | cinA | hypothetical protein DR75_1888 | 0.51 | 0.30 | 0.06 | NC | NC | NC | 1 | S | ko:K03742 |
| AIL03323.1 |  | lytR | cell envelope-related function transcriptional attenuator common domain protein | 0.17 | -0.11 | 0.24 | NC | NC | NC | 1 | IK | - |
| AIL03324.1 |  | yojA | citrate transporter family protein | 0.28 | -0.16 | 0.21 | NC | NC | NC | 1 | EG | ko:K03299 |
| AIL03327.1 |  | - | type I restriction modification DNA specificity domain protein | 1.04 | -0.58 | -0.08 | U | NC | NC | 11 | S | - |
| AIL03329.1 |  | folP | dihydropteroate synthase | 0.75 | 0.07 | 0.18 | NC | NC | NC | 1 | H | ko:K00796 |
| AIL03330.1 |  | - | hypothetical protein DR75_442 | 0.73 | 0.37 | 0.36 | NC | NC | NC | 1 | S | - |
| AIL03331.1 |  | glxK | glycerate kinase family protein | -0.50 | -0.37 | -0.14 | NC | NC | NC | 1 | G | ko:K00865 |
| AIL03333.1 |  | rrmJ | hemolysin TlyA family protein | 0.21 | 0.33 | 0.14 | NC | NC | NC | 1 | J | ko:K06442 |
| AIL03334.1 |  | pnp | polyribonucleotide nucleotidyltransferase | -0.14 | -0.20 | -0.28 | NC | NC | NC | 1 | J | ko:K00962 |
| AIL03335.1 |  | ccpN | CBS domain protein | -0.08 | 0.13 | -0.10 | NC | NC | NC | 1 | K | - |
| AIL03338.1 |  | gatA | aspartyl/glutamyl-tRNA(Asn/Gln) amidotransferase, A subunit | -0.05 | -0.02 | -0.07 | NC | NC | NC | 1 | F | ko:K02433 |
| AIL03341.1 |  | addA | helicase-exonuclease AddAB, AddA subunit | -0.09 | -0.08 | -0.07 | NC | NC | NC | 1 | L | ko:K16898 |
| AIL03342.1 |  | tcsA | basic membrane family protein | 0.32 | 0.36 | 0.20 | NC | NC | NC | 1 | S | ko:K02058 |
| AIL03344.1 |  | radA | DNA repair protein RadA | 0.47 | 0.02 | -0.02 | NC | NC | NC | 1 | O | ko:K04485 |
| AIL03347.1 |  | - | 2-dehydro-3-deoxyphosphogluconate aldolase | 0.44 | 0.74 | 0.41 | NC | NC | NC | 1 | S | ko:K17463 |
| AIL03349.1 |  | tmk | thymidylate kinase | -0.81 | -0.22 | -0.24 | NC | NC | NC | 1 | F | ko:K00943 |
| AIL03350.1 |  | bfmBAB | 2-oxoisovalerate dehydrogenase subunit beta | -0.42 | 0.43 | 0.44 | NC | NC | NC | 1 | C | ko:K00167 |
| AIL03351.1 |  | hslU | ATP-dependent protease HslVU, ATPase subunit | -0.45 | 0.06 | -0.10 | NC | NC | NC | 1 | O | ko:K03667 |
| AIL03352.1 |  | birA | biotin--[acetyl-CoA-carboxylase] ligase | 0.02 | 0.22 | -0.01 | NC | NC | NC | 1 | K | ko:K03524 |
| AIL03353.1 |  | - | hypothetical protein DR75_255 | 0.02 | -1.09 | 0.19 | NC | D | NC | 23 | K | - |
| AIL03359.1 |  | - | mga helix-turn-helix domain protein | 0.27 | 0.53 | -0.36 | NC | NC | NC | 1 | K | - |
| AIL03362.1 |  | - | hypothetical protein DR75_2725 | 0.42 | 0.05 | 0.08 | NC | NC | NC | 1 | - | - |
| AIL03364.1 |  | cspA | 'Cold-shock' DNA-binding domain protein | 0.20 | 1.03 | 0.95 | NC | U | NC | 15 | K | ko:K03704 |
| AIL03365.1 |  | - | ABC transporter family protein | -0.04 | -0.40 | -0.34 | NC | NC | NC | 1 | V | ko:K02003 |
| AIL03366.1 |  | paiA | protease synthase and sporulation negative regulatory protein PAI 1 | 0.03 | -0.16 | 0.34 | NC | NC | NC | 1 | K | ko:K22441 |
| AIL03369.1 |  | - | cation diffusion facilitator transporter family protein | -0.83 | -0.76 | -0.39 | NC | NC | NC | 1 | U | - |
| AIL03373.1 |  | rexB | PD-(D/E)XK nuclease superfamily protein | 0.69 | 0.96 | 0.37 | NC | NC | NC | 1 | L | ko:K16899 |
| AIL03374.1 |  | - | PTS system sugar-specific permease component family protein | 0.53 | 0.35 | -1.29 | NC | NC | D | 19 | G | ko:K02775 |
| AIL03376.1 |  | manA | mannose-6-phosphate isomerase, class I | 0.02 | 0.35 | 0.15 | NC | NC | NC | 1 | G | ko:K01809 |
| AIL03379.1 |  | topB | DNA topoisomerase III family protein | -0.06 | -0.86 | -1.02 | NC | NC | D | 19 | L | ko:K03169 |
| AIL03380.1 |  | ccl | queT transporter family protein | -0.20 | -0.93 | -0.86 | NC | NC | NC | 1 | S | - |
| AIL03382.1 |  | - | hypothetical protein DR75_1668 | 0.02 | -0.97 | -1.16 | NC | NC | D | 19 | - | - |
| AIL03384.1 |  | pyk | pyruvate kinase | 0.01 | 0.21 | -0.04 | NC | NC | NC | 1 | G | ko:K00873 |
| AIL03385.1 |  | msrR | regulatory msrR domain protein | -0.66 | -0.56 | -0.33 | NC | NC | NC | 1 | K | - |
| AIL03386.1 |  | codY | GTP-sensing transcriptional pleiotropic repressor CodY | -0.09 | 0.18 | 0.04 | NC | NC | NC | 1 | K | ko:K03706 |
| AIL03388.1 |  | msrB | peptide methionine sulfoxide reductase MsrB | 0.79 | 0.12 | -0.11 | NC | NC | NC | 1 | O | ko:K07305 |
| AIL03389.1 |  | nadK | ATP-NAD kinase family protein | 0.42 | 0.87 | -0.08 | NC | NC | NC | 1 | H | ko:K00858 |
| AIL03390.1 |  | yclP | ABC transporter family protein | 0.42 | -0.34 | -0.51 | NC | NC | NC | 1 | P | ko:K02013 |
| AIL03391.1 |  | - | hypothetical protein DR75_1893 | 0.74 | 0.34 | 0.34 | NC | NC | NC | 1 | - | - |
| AIL03392.1 |  | XK27_02070 | nitroreductase family protein | -0.57 | -0.80 | -0.58 | NC | NC | NC | 1 | S | ko:K07078 |
| AIL03396.1 |  | glmU | UDP-N-acetylglucosamine diphosphorylase/glucosamine-1-phosphate N-acetyltransferase | -0.10 | -0.22 | -0.24 | NC | NC | NC | 1 | M | ko:K04042 |
| AIL03397.1 |  | - | helix-turn-helix family protein | 0.50 | -0.04 | -0.07 | NC | NC | NC | 1 | K | - |
| AIL03401.1 |  | - | bacterial regulatory s, tetR family protein | 0.65 | 0.28 | 0.43 | NC | NC | NC | 1 | K | - |
| AIL03402.1 |  | - | hypothetical protein DR75_693 | 0.37 | 0.34 | 0.41 | NC | NC | NC | 1 | S | - |
| AIL03403.1 |  | potA11 | ABC transporter family protein | -0.43 | 0.75 | -0.86 | NC | NC | NC | 1 | E | ko:K02010 |
| AIL03404.1 |  | nylA | amidase family protein | 0.73 | 1.19 | 0.30 | NC | U | NC | 15 | J | ko:K01426 |
| AIL03405.1 |  | groL | chaperonin GroL | -0.09 | -0.38 | -0.41 | NC | NC | NC | 1 | O | ko:K04077 |
| AIL03406.1 |  | dgs | glycosyl transferases group 1 family protein | 0.22 | 0.21 | 0.06 | NC | NC | NC | 1 | M | ko:K13677 |
| AIL03408.1 |  | - | helix-turn-helix family protein | -2.55 | -3.60 | -1.91 | D | D | D | 3 | K | - |
| AIL03409.1 |  | rpsP | ribosomal protein S16 | -0.01 | 0.03 | 0.06 | NC | NC | NC | 1 | J | ko:K02959 |
| AIL03410.1 |  | - | sensory box protein | -0.63 | -0.32 | -0.32 | NC | NC | NC | 1 | S | - |
| AIL03411.1 |  | - | hypothetical protein DR75_2749 | 0.03 | -0.66 | -0.13 | NC | NC | NC | 1 | S | - |
| AIL03412.1 |  | yisX | pentapeptide repeats family protein | 1.37 | 0.19 | 1.84 | U | NC | U | 14 | S | - |
| AIL03414.1 |  | - | helix-turn-helix family protein | 0.74 | 0.35 | 0.31 | NC | NC | NC | 1 | K | - |
| AIL03415.1 |  | cah | eukaryotic-type carbonic anhydrase family protein | 0.55 | 0.53 | 0.14 | NC | NC | NC | 1 | P | ko:K01674 |
| AIL03416.1 |  | menE | O-succinylbenzoate-CoA ligase | 1.60 | 0.71 | 0.57 | U | NC | NC | 11 | H | ko:K01911 |
| AIL03417.1 |  | nanE | thiazole biosynthesis ThiG family protein | 0.04 | 0.34 | 0.08 | NC | NC | NC | 1 | G | ko:K01788 |
| AIL03418.1 |  | - | formate/nitrite transporter family protein | -0.10 | -0.60 | -0.51 | NC | NC | NC | 1 | P | ko:K02598 |
| AIL03420.1 |  | ydgI | nitroreductase | -0.14 | -0.31 | -0.10 | NC | NC | NC | 1 | C | - |
| AIL03421.1 |  | - | binding--dependent transport system inner membrane component family protein | -0.23 | 0.54 | 0.21 | NC | NC | NC | 1 | U | ko:K02025 |
| AIL03425.1 |  | fabI | enoyl-[acyl-carrier-protein] reductase [NADH] | -0.16 | 0.23 | 0.01 | NC | NC | NC | 1 | I | ko:K00208 |
| AIL03427.1 |  | dmpI | 4-oxalocrotonate tautomerase enzyme family protein | -0.75 | 0.12 | 0.16 | NC | NC | NC | 1 | G | ko:K01821 |
| AIL03428.1 |  | pyrR | bifunctional protein pyrR | -1.73 | -0.15 | -0.07 | D | NC | NC | 6 | F | ko:K02825 |
| AIL03429.1 |  | yybT | DHH family protein | 0.15 | 0.32 | -0.08 | NC | NC | NC | 1 | T | - |
| AIL03431.1 |  | - | hypothetical protein DR75_163 | 0.00 | -0.30 | -0.19 | NC | NC | NC | 1 | S | - |
| AIL03433.1 |  | pdhA | pyruvate dehydrogenase (acetyl-transferring) E1 component, alpha subunit | 0.29 | 0.39 | 0.19 | NC | NC | NC | 1 | C | ko:K00161 |
| AIL03436.1 |  | ulaA | PTS system sugar-specific permease component family protein | 0.47 | -0.48 | -0.72 | NC | NC | NC | 1 | S | ko:K03475 |
| AIL03437.1 |  | argR1 | hypothetical protein DR75_2141 | 0.74 | -0.26 | -0.05 | NC | NC | NC | 1 | K | ko:K03402 |
| AIL03439.1 |  | phoR | sensory box protein | -0.18 | 0.33 | 0.17 | NC | NC | NC | 1 | T | ko:K07636 |
| AIL03440.1 |  | vex2 | ABC transporter family protein | 0.09 | 0.19 | -0.07 | NC | NC | NC | 1 | V | ko:K02003 |
| AIL03441.1 |  | - | ankyrin repeat family domain protein | 0.88 | 0.02 | 0.12 | NC | NC | NC | 1 | S | ko:K06867 |
| AIL03442.1 |  | metQ | NLPA lipofamily protein | 0.55 | 0.46 | 0.65 | NC | NC | NC | 1 | M | ko:K02072 |
| AIL03443.1 |  | yodB | hxlR-like helix-turn-helix family protein | 0.39 | -0.18 | -0.06 | NC | NC | NC | 1 | K | - |
| AIL03444.1 |  | dacA | disA bacterial checkpoint controller nucleotide-binding family protein | -0.05 | 0.22 | 0.31 | NC | NC | NC | 1 | S | ko:K18672 |
| AIL03445.1 |  | yfnB | putative HAD-hydrolase yfnB | 0.30 | 0.05 | -0.05 | NC | NC | NC | 1 | S | ko:K01560 |
| AIL03446.1 |  | glnQ | ABC transporter family protein | -0.48 | -0.11 | -0.33 | NC | NC | NC | 1 | E | ko:K17076 |
| AIL03449.1 |  | XK27_00220 | alpha/beta hydrolase family protein | 0.62 | 0.36 | 0.25 | NC | NC | NC | 1 | S | ko:K06999 |
| AIL03452.1 |  | gpsA | ketopantoate reductase PanE/ApbA family protein | 0.03 | -0.12 | -0.21 | NC | NC | NC | 1 | I | ko:K00057 |
| AIL03453.1 |  | yvgN | aldo/keto reductase family protein | 0.74 | 0.38 | 0.11 | NC | NC | NC | 1 | S | - |
| AIL03455.1 |  | alsS | acetolactate synthase, catabolic | -0.52 | 0.63 | 0.43 | NC | NC | NC | 1 | EH | ko:K01652 |
| AIL03457.1 |  | murA | UDP-N-acetylglucosamine 1-carboxyvinyltransferase | -1.48 | -0.49 | -0.87 | D | NC | NC | 6 | M | ko:K00790 |
| AIL03458.1 |  | peb1A | bacterial extracellular solute-binding s, 3 family protein | 0.61 | -0.05 | 0.09 | NC | NC | NC | 1 | ET | ko:K10039 |
| AIL03459.1 |  | oadB | sodium ion-translocating decarboxylase, beta subunit | 0.21 | 0.55 | 0.69 | NC | NC | NC | 1 | C | ko:K01572 |
| AIL03461.1 |  | - | helix-turn-helix family protein | 0.70 | 0.56 | 1.64 | NC | NC | U | 5 | K | - |
| AIL03462.1 |  | ytfP | flavo, family protein | 0.24 | -0.04 | 0.17 | NC | NC | NC | 1 | S | ko:K07007 |
| AIL03463.1 |  | - | hypothetical protein DR75_382 | -0.25 | -0.44 | 0.80 | NC | NC | NC | 1 | - | - |
| AIL03465.1 |  | yvcC | von Willebrand factor type A domain protein | 0.18 | 1.48 | 0.45 | NC | U | NC | 15 | M | - |
| AIL03467.1 |  | bglA | putative phospho-beta-glucosidase | 0.71 | 1.29 | -0.25 | NC | U | NC | 15 | G | ko:K01223 |
| AIL03468.1 |  | aspB | aminotransferase class I and II family protein | -0.06 | -0.07 | -0.11 | NC | NC | NC | 1 | E | ko:K00812 |
| AIL03469.1 |  | aroC | chorismate synthase | -0.63 | -0.61 | -0.66 | NC | NC | NC | 1 | E | ko:K01736 |
| AIL03471.1 |  | thrS | threonine--tRNA ligase | -0.29 | -0.16 | -0.14 | NC | NC | NC | 1 | J | ko:K01868 |
| AIL03473.1 |  | ispE | 4-(cytidine 5'-diphospho)-2-C-methyl-D-erythritol kinase | 0.14 | -0.26 | -0.26 | NC | NC | NC | 1 | F | ko:K00919 |
| AIL03474.1 |  | ysjB | oxidoreductase, NAD-binding Rossmann fold family protein | 0.07 | -0.01 | -0.07 | NC | NC | NC | 1 | S | ko:K03810 |
| AIL03475.1 |  | citC | [citrate (pro-3S)-lyase] ligase | 0.84 | 0.46 | 0.25 | NC | NC | NC | 1 | H | ko:K01910 |
| AIL03477.1 |  | mnmA | tRNA (5-methylaminomethyl-2-thiouridylate)-methyltransferase | 0.57 | -0.12 | -0.04 | NC | NC | NC | 1 | J | ko:K00566 |
| AIL03482.1 |  | - | hypothetical protein DR75_210 | -0.14 | 0.26 | -0.03 | NC | NC | NC | 1 | S | ko:K07010 |
| AIL03483.1 |  | pmr1 | calcium-translocating P-type ATPase, PMCA-type | 0.17 | -0.07 | -0.36 | NC | NC | NC | 1 | P | ko:K01537 |
| AIL03484.1 |  | ypmS | hypothetical protein DR75_683 | 0.08 | -0.40 | -0.31 | NC | NC | NC | 1 | S | - |
| AIL03485.1 |  | - | hypothetical protein DR75_1062 | 2.08 | 0.23 | 0.38 | U | NC | NC | 11 | J | - |
| AIL03486.1 |  | - | hypothetical protein DR75_2656 | 0.10 | 0.35 | 1.21 | NC | NC | U | 5 | S | - |
| AIL03487.1 |  | yaaT | hypothetical protein DR75_1454 | -0.57 | -0.32 | -0.09 | NC | NC | NC | 1 | S | - |
| AIL03488.1 |  | vraS | histidine kinase family protein | -0.59 | 0.36 | 0.75 | NC | NC | NC | 1 | T | ko:K07681 |
| AIL03490.1 |  | ptbA | PTS system, glucose subfamily, IIA component domain protein | 0.86 | 0.51 | 0.49 | NC | NC | NC | 1 | G | ko:K02755 |
| AIL03491.1 |  | secY | preprotein translocase, SecY subunit | 0.03 | 0.03 | -0.27 | NC | NC | NC | 1 | U | ko:K03076 |
| AIL03493.1 |  | yitV | phospholipase/Carboxylesterase family protein | 0.49 | 0.04 | -0.17 | NC | NC | NC | 1 | S | ko:K06889 |
| AIL03494.1 |  | mnaA | UDP-N-acetylglucosamine 2-epimerase | 0.17 | 0.06 | 0.17 | NC | NC | NC | 1 | G | ko:K01791 |
| AIL03495.1 |  | - | hypothetical protein DR75_1096 | -0.42 | -0.03 | 0.69 | NC | NC | NC | 1 | G | - |
| AIL03497.1 |  | - | periplasmic binding family protein | -0.77 | -0.31 | -0.37 | NC | NC | NC | 1 | P | ko:K02016 |
| AIL03498.1 |  | acmA | mannosyl-glycoendo-beta-N-acetylglucosaminidase family protein | -0.85 | -0.35 | 0.04 | NC | NC | NC | 1 | NU | ko:K01185 |
| AIL03500.1 |  | spxA | regulatory protein spx | -1.00 | -0.60 | -1.06 | NC | NC | D | 19 | K | ko:K16509 |
| AIL03501.1 |  | citX | holo-ACP synthase CitX | 0.71 | -0.02 | -0.58 | NC | NC | NC | 1 | HI | ko:K05964 |
| AIL03502.1 |  | - | recombinase family protein | 0.46 | 0.49 | -0.78 | NC | NC | NC | 1 | L | - |
| AIL03505.1 |  | - | hypothetical protein DR75_1808 | -0.08 | -0.52 | -0.20 | NC | NC | NC | 1 | S | - |
| AIL03506.1 |  | - | glycine cleavage H-family protein | -0.35 | 0.91 | 0.42 | NC | NC | NC | 1 | E | ko:K02437 |
| AIL03507.1 |  | tcyP | L-cystine uptake protein tcyP | -1.36 | -0.78 | -0.88 | D | NC | NC | 6 | U | ko:K06956 |
| AIL03508.1 |  | - | lysM domain protein | -0.63 | -0.16 | -0.28 | NC | NC | NC | 1 | M | - |
| AIL03510.1 |  | manL | PTS system, mannose/fructose/sorbose, IIB component family protein | -0.63 | 0.18 | 0.04 | NC | NC | NC | 1 | G | ko:K02769 |
| AIL03511.1 |  | nifJ | ferredoxin (flavodoxin) oxidoreductase | -1.11 | 0.61 | -0.54 | D | NC | NC | 6 | C | ko:K03737 |
| AIL03513.1 |  | tsf | translation elongation factor Ts | -0.07 | 0.08 | 0.08 | NC | NC | NC | 1 | J | ko:K02357 |
| AIL03514.1 |  | aroF | phospho-2-dehydro-3-deoxyheptonate aldolase | -1.24 | -0.08 | -0.24 | D | NC | NC | 6 | E | ko:K03856 |
| AIL03515.1 |  | yrzL | hypothetical protein DR75_267 | 0.43 | 0.20 | -0.12 | NC | NC | NC | 1 | S | - |
| AIL03516.1 |  | ntpG | ATP synthase (F/14-kDa) subunit | 0.10 | -0.52 | -0.25 | NC | NC | NC | 1 | C | ko:K02122 |
| AIL03521.1 |  | yjhE | hypothetical protein DR75_415 | 0.94 | 0.26 | -0.08 | NC | NC | NC | 1 | S | - |
| AIL03522.1 |  | - | acetyltransferase domain protein | -0.09 | -0.12 | -0.23 | NC | NC | NC | 1 | K | ko:K06977 |
| AIL03523.1 |  | ylmE | hypothetical protein DR75_58 | -0.52 | -0.56 | -0.08 | NC | NC | NC | 1 | S | ko:K06997 |
| AIL03527.1 |  | - | D-isomer specific 2-hydroxyacid dehydrogenase, NAD binding domain protein | 0.20 | -0.42 | 0.00 | NC | NC | NC | 1 | CH | - |
| AIL03529.1 |  | cysE | serine O-acetyltransferase | -0.38 | -0.63 | 0.09 | NC | NC | NC | 1 | E | ko:K00640 |
| AIL03531.1 |  | mreC | rod shape-determining protein MreC | -1.12 | -0.92 | -1.31 | D | NC | D | 8 | M | ko:K03570 |
| AIL03532.1 |  | fabG | 3-oxoacyl-[acyl-carrier-protein] reductase | -0.53 | 0.04 | -0.17 | NC | NC | NC | 1 | IQ | ko:K00059 |
| AIL03533.1 |  | nagB | glucosamine-6-phosphate deaminase | 0.34 | 0.37 | 0.26 | NC | NC | NC | 1 | G | ko:K02564 |
| AIL03534.1 |  | gmk | guanylate kinase | -1.05 | -0.26 | -0.20 | D | NC | NC | 6 | F | ko:K00942 |
| AIL03535.1 |  | - | peptidase M20/M25/M40 family protein | 0.77 | -0.86 | -0.26 | NC | NC | NC | 1 | E | - |
| AIL03536.1 |  | yunD | hypothetical protein DR75_251 | -0.16 | -0.22 | -0.78 | NC | NC | NC | 1 | F | ko:K01081 |
| AIL03537.1 |  | rnhC | ribonuclease HIII | -1.66 | 0.26 | 0.28 | D | NC | NC | 6 | L | ko:K03471 |
| AIL03538.1 |  | secG | preprotein translocase, SecG subunit | -0.22 | -0.41 | -0.95 | NC | NC | NC | 1 | U | ko:K03075 |
| AIL03539.1 |  | sbcC | exonuclease SbcCD, C subunit | -0.07 | 0.15 | 0.34 | NC | NC | NC | 1 | L | ko:K03546 |
| AIL03540.1 |  | - | coA-ligase family protein | 1.11 | 0.68 | 0.32 | U | NC | NC | 11 | C | - |
| AIL03542.1 |  | - | PGAP1-like family protein | 2.01 | 1.02 | 1.26 | U | U | U | 2 | S | - |
| AIL03543.1 |  | - | amidohydrolase family protein | 0.15 | 0.36 | 0.17 | NC | NC | NC | 1 | F | - |
| AIL03544.1 |  | vicK | sensory box protein | -0.02 | 0.01 | -0.51 | NC | NC | NC | 1 | T | ko:K07652 |
| AIL03545.1 |  | - | hypothetical protein DR75_2114 | 1.85 | 1.88 | 1.87 | U | U | U | 2 | M | ko:K03931 |
| AIL03546.1 |  | rsmE | RNA methyltransferase, RsmE family protein | -0.72 | -0.42 | -0.31 | NC | NC | NC | 1 | J | ko:K09761 |
| AIL03548.1 |  | scrB | sucrose-6-phosphate hydrolase family protein | -1.07 | -0.65 | -1.12 | D | NC | D | 8 | G | ko:K01193 |
| AIL03550.1 |  | - | NUDIX domain protein | -0.04 | -0.25 | -0.42 | NC | NC | NC | 1 | F | ko:K01515 |
| AIL03551.1 |  | ldh | L-lactate dehydrogenase | 0.94 | 0.37 | 0.11 | NC | NC | NC | 1 | C | ko:K00016 |
| AIL03553.1 |  | - | hypothetical protein DR75_985 | -0.24 | -0.76 | -0.35 | NC | NC | NC | 1 | S | - |
| AIL03554.1 |  | yqfL | kinase/pyrophosphorylase family protein | -0.48 | -0.33 | -0.97 | NC | NC | NC | 1 | S | ko:K09773 |
| AIL03559.1 |  | - | ABC transporter family protein | -0.20 | 0.13 | 0.12 | NC | NC | NC | 1 | V | ko:K02003 |
| AIL03562.1 |  | agaS | SIS domain protein | -0.43 | -0.23 | -0.25 | NC | NC | NC | 1 | M | ko:K02082 |
| AIL03563.1 |  | nrdI | nrdI Flavodoxin like family protein | 0.11 | -0.05 | -0.13 | NC | NC | NC | 1 | F | ko:K03647 |
| AIL03565.1 |  | - | hypothetical protein DR75_150 | 0.31 | -0.22 | -0.17 | NC | NC | NC | 1 | - | - |
| AIL03566.1 |  | fnq20 | FAD-NAD(P)-binding family protein | 0.12 | 0.17 | 0.92 | NC | NC | NC | 1 | S | - |
| AIL03569.1 |  | nt5e | HAD hydrolase, IA, variant 1 family protein | 0.16 | -0.11 | -0.36 | NC | NC | NC | 1 | S | ko:K01091 |
| AIL03570.1 |  | der | ribosome-associated GTPase EngA | 0.12 | -0.07 | 0.03 | NC | NC | NC | 1 | S | ko:K03977 |
| AIL03571.1 |  | phaJ | maoC like domain protein | -0.36 | 0.63 | 0.53 | NC | NC | NC | 1 | I | - |
| AIL03573.1 |  | - | phage major tail protein, TP901-1 family | 0.63 | -0.22 | 0.67 | NC | NC | NC | 1 | S | - |
| AIL03575.1 |  | yqjA | hypothetical protein DR75_878 | 0.57 | 1.13 | -0.54 | NC | U | NC | 15 | S | - |
| AIL03576.1 |  | scpB | segregation and condensation protein B | 0.07 | 0.15 | -0.11 | NC | NC | NC | 1 | D | ko:K06024 |
| AIL03578.1 |  | aspC | aminotransferase class-V family protein | -0.28 | -0.14 | -0.12 | NC | NC | NC | 1 | E | ko:K14260 |
| AIL03579.1 |  | glyQ | glycine--tRNA ligase, alpha subunit | -0.45 | -0.19 | -0.03 | NC | NC | NC | 1 | J | ko:K01878 |
| AIL03582.1 |  | ruvB | Holliday junction DNA helicase RuvB | 0.10 | -0.38 | -0.39 | NC | NC | NC | 1 | L | ko:K03551 |
| AIL03583.1 |  | ykuL | CBS domain protein | -1.41 | -0.20 | -0.57 | D | NC | NC | 6 | S | - |
| AIL03586.1 |  | mhqA | glyoxalase/Bleomycin resistance /Dioxygenase superfamily protein | 0.84 | 0.38 | 0.10 | NC | NC | NC | 1 | E | ko:K15975 |
| AIL03587.1 |  | mnmE | tRNA modification GTPase TrmE | 1.00 | -0.22 | -0.04 | NC | NC | NC | 1 | S | ko:K03650 |
| AIL03591.1 |  | pocR1 | helix-turn-helix domain protein | 0.20 | -0.82 | -1.34 | NC | NC | D | 19 | K | - |
| AIL03592.1 |  | - | sugar-specific transcriptional regulator TrmB family protein | -0.75 | -0.67 | -0.87 | NC | NC | NC | 1 | K | - |
| AIL03593.1 |  | - | universal stress family protein | -0.88 | 0.07 | -0.33 | NC | NC | NC | 1 | T | - |
| AIL03596.1 |  | rplD | 50S ribosomal protein L4 | -0.45 | -0.21 | -0.30 | NC | NC | NC | 1 | J | ko:K02926 |
| AIL03597.1 |  | - | hypothetical protein DR75_1523 | 1.58 | -0.32 | -0.19 | U | NC | NC | 11 | - | - |
| AIL03599.1 |  | - | LPXTG cell wall anchor domain protein | 0.34 | 0.71 | -0.20 | NC | NC | NC | 1 | S | - |
| AIL03600.1 |  | cdr | FAD dependent oxidoreductase family protein | 9.97 | 9.97 | - | U | U | NC | 9 | P | - |
| AIL03601.1 |  | - | dihydroorotate dehydrogenase family protein | 1.79 | 1.90 | 1.47 | U | U | U | 2 | G | ko:K01625 |
| AIL03602.1 |  | srlB | PTS system glucitol/sorbitol-specific IIA component family protein | 0.13 | 2.27 | 2.09 | NC | U | U | 4 | G | ko:K02781 |
| AIL03606.1 |  | ung | uracil-DNA glycosylase | -0.29 | -0.04 | 0.00 | NC | NC | NC | 1 | L | ko:K03648 |
| AIL03608.1 |  | ulaB | PTS system, Lactose/Cellobiose specific IIB subunit | -0.15 | 1.41 | 1.31 | NC | U | U | 4 | G | ko:K02822 |
| AIL03609.1 |  | rplK | ribosomal protein L11 | -0.21 | -0.03 | -0.47 | NC | NC | NC | 1 | J | ko:K02867 |
| AIL03610.1 |  | ribF | riboflavin biosynthesis protein RibF | 0.03 | -0.06 | 0.24 | NC | NC | NC | 1 | H | ko:K11753 |
| AIL03611.1 |  | - | putative lipoprotein | -0.35 | 0.00 | 0.11 | NC | NC | NC | 1 | - | - |
| AIL03613.1 |  | era | GTP-binding protein Era | 0.26 | 0.39 | 0.22 | NC | NC | NC | 1 | S | ko:K03595 |
| AIL03615.1 |  | - | glycosyl Hydrolase Family 88 family protein | -0.14 | 0.86 | 0.32 | NC | NC | NC | 1 | S | ko:K18581 |
| AIL03616.1 |  | - | hypothetical protein DR75_2147 | -0.36 | -0.43 | 0.10 | NC | NC | NC | 1 | S | - |
| AIL03617.1 |  | ykuJ | hypothetical protein DR75_1581 | -0.76 | -0.10 | -0.56 | NC | NC | NC | 1 | S | - |
| AIL03618.1 |  | bglC | glycosyl hydrolase 1 family protein | 0.34 | 0.55 | 0.38 | NC | NC | NC | 1 | G | ko:K01223 |
| AIL03620.1 |  | yjhB | NUDIX domain protein | 1.22 | -0.69 | 1.34 | U | NC | U | 14 | F | ko:K01515 |
| AIL03621.1 |  | - | PTS system sorbose subIIB component family protein | -1.20 | 0.26 | -0.13 | D | NC | NC | 6 | G | ko:K19507 |
| AIL03622.1 |  | algC | phosphoglucomutase/phosphomannomutase, alpha/beta/alpha domain III family protein | -0.24 | 0.29 | 0.19 | NC | NC | NC | 1 | G | ko:K01840 |
| AIL03623.1 |  | ispF | 2-C-methyl-D-erythritol 2,4-cyclodiphosphate synthase | 0.80 | -0.65 | -0.17 | NC | NC | NC | 1 | F | ko:K01770 |
| AIL03624.1 |  | ykoD | heme ABC exporter, ATP-binding protein CcmA | -0.51 | -0.25 | -0.15 | NC | NC | NC | 1 | P | ko:K16786 |
| AIL03625.1 |  | oadG | hlyD secretion family protein | 0.90 | 0.17 | 0.83 | NC | NC | NC | 1 | I | - |
| AIL03627.1 |  | nagD | putative phosphatases involved in N-acetyl-glucosamine catabolism | 0.39 | -0.03 | 0.04 | NC | NC | NC | 1 | G | ko:K01101 |
| AIL03630.1 |  | - | shikimate / quinate 5-dehydrogenase family protein | 0.36 | -0.42 | -0.32 | NC | NC | NC | 1 | E | ko:K01750 |
| AIL03633.1 |  | - | osmC-like family protein | 0.64 | 0.91 | 0.53 | NC | NC | NC | 1 | O | - |
| AIL03634.1 |  | - | HAD hydrolase, IIB family protein | -0.60 | -0.20 | -0.22 | NC | NC | NC | 1 | S | - |
| AIL03637.1 |  | - | corA-like Mg2+ transporter family protein | 0.02 | 0.01 | 1.28 | NC | NC | U | 5 | P | ko:K03284 |
| AIL03638.1 |  | ylbM | hypothetical protein DR75_1561 | -0.06 | -0.68 | -1.02 | NC | NC | D | 19 | S | - |
| AIL03639.1 |  | - | bacterial extracellular solute-binding s, 5 Middle family protein | -0.45 | 0.18 | 0.38 | NC | NC | NC | 1 | E | ko:K15580 |
| AIL03640.1 |  | M1-560 | bacterial regulatory helix-turn-helix, lysR family protein | -1.58 | -0.38 | -0.56 | D | NC | NC | 6 | K | - |
| AIL03641.1 |  | trmH | RNA 2'-O ribose methyltransferase substrate binding family protein | -0.19 | 0.20 | -0.16 | NC | NC | NC | 1 | J | ko:K03218 |
| AIL03642.1 |  | - | hypothetical protein DR75_2374 | 0.67 | 1.00 | 1.60 | NC | U | U | 4 | I | - |
| AIL03643.1 |  | fbp | fructose-1,6-bisphosphatase class 3 | 1.93 | 0.84 | 0.22 | U | NC | NC | 11 | G | ko:K04041 |
| AIL03644.1 |  | lplC | binding--dependent transport system inner membrane component family protein | 0.42 | 1.24 | 1.06 | NC | U | U | 4 | U | ko:K17320 |
| AIL03645.1 |  | - | acetyltransferase family protein | -0.60 | 0.05 | -0.02 | NC | NC | NC | 1 | K | - |
| AIL03647.1 |  | - | phage portal, SPP1 Gp6-like family protein | 0.03 | -0.26 | 0.00 | NC | NC | NC | 1 | S | - |
| AIL03648.1 |  | - | hypothetical protein DR75_1326 | -1.44 | -0.58 | -0.25 | D | NC | NC | 6 | S | - |
| AIL03650.1 |  | ftsX | ftsX-like permease family protein | 0.08 | -0.03 | -0.23 | NC | NC | NC | 1 | D | ko:K09811 |
| AIL03651.1 |  | gntK | hypothetical protein DR75_1944 | -0.42 | 0.16 | -0.17 | NC | NC | NC | 1 | G | ko:K00851 |
| AIL03653.1 |  | yidC | membrane insertase, YidC/Oxa1 family domain protein | -0.25 | 0.06 | -0.16 | NC | NC | NC | 1 | U | ko:K03217 |
| AIL03654.1 |  | atpG | ATP synthase F1, gamma subunit | -0.45 | -0.52 | -0.35 | NC | NC | NC | 1 | C | ko:K02115 |
| AIL03655.1 |  | - | hypothetical protein DR75_1664 | 0.21 | -0.64 | -0.15 | NC | NC | NC | 1 | - | - |
| AIL03656.1 |  | srtA | sortase family protein | 0.68 | 0.19 | 0.13 | NC | NC | NC | 1 | M | ko:K07284 |
| AIL03657.1 |  | XK27_10405 | bacterial membrane YfhO family protein | 0.15 | -0.17 | 0.52 | NC | NC | NC | 1 | S | - |
| AIL03659.1 |  | - | hypothetical protein DR75_157 | 1.16 | 0.21 | 0.48 | U | NC | NC | 11 | - | - |
| AIL03660.1 |  | ybhE | hypothetical protein DR75_539 | -0.01 | 0.66 | 0.49 | NC | NC | NC | 1 | S | ko:K09963 |
| AIL03661.1 |  | nox | NADH oxidase | 0.44 | 0.15 | 0.18 | NC | NC | NC | 1 | S | ko:K17869 |
| AIL03662.1 |  | - | hypothetical protein DR75_1498 | -0.28 | -2.25 | -0.28 | NC | D | NC | 23 | - | - |
| AIL03663.1 |  | - | putative membrane protein | -0.02 | 0.27 | -0.34 | NC | NC | NC | 1 | - | - |
| AIL03664.1 |  | - | histidine phosphatase super family protein | 0.35 | 0.11 | -0.17 | NC | NC | NC | 1 | G | ko:K15634 |
| AIL03666.1 |  | oppB | binding--dependent transport system inner membrane component family protein | -0.94 | -0.48 | -0.72 | NC | NC | NC | 1 | EP | ko:K15581 |
| AIL03668.1 |  | tdk | thymidine kinase family protein | -0.14 | 0.03 | -0.09 | NC | NC | NC | 1 | F | ko:K00857 |
| AIL03670.1 |  | clpE | ATP-dependent Clp protease ATP-binding subunit ClpE | 0.64 | 0.00 | -0.08 | NC | NC | NC | 1 | O | ko:K03697 |
| AIL03671.1 |  | pgm | phosphoglucomutase/phosphomannomutase, alpha/beta/alpha domain III family protein | -0.22 | 0.09 | 0.07 | NC | NC | NC | 1 | G | ko:K01835 |
| AIL03672.1 |  | cca | poly A polymerase head domain protein | -0.42 | -0.25 | -0.37 | NC | NC | NC | 1 | J | ko:K00974 |
| AIL03673.1 |  | dltD | D-alanyl-lipoteichoic acid biosynthesis protein DltD | -0.34 | -0.80 | -0.53 | NC | NC | NC | 1 | M | ko:K03740 |
| AIL03674.1 |  | pspC | pspC domain protein | 0.93 | 0.56 | 0.91 | NC | NC | NC | 1 | KT | ko:K03973 |
| AIL03675.1 |  | npr | NADH peroxidase | 0.42 | 0.53 | 0.27 | NC | NC | NC | 1 | S | ko:K05910 |
| AIL03676.1 |  | - | kinase/pyrophosphorylase family protein | -0.21 | -0.12 | -0.05 | NC | NC | NC | 1 | F | ko:K09773 |
| AIL03677.1 |  | accC | acetyl-CoA carboxylase, biotin carboxylase subunit | -0.55 | -0.13 | -0.17 | NC | NC | NC | 1 | I | ko:K01961 |
| AIL03679.1 |  | tagD | glycerol-3-phosphate cytidylyltransferase | -0.23 | -0.22 | -0.06 | NC | NC | NC | 1 | IM | ko:K00968 |
| AIL03681.1 |  | accB | acetyl-CoA carboxylase, biotin carboxyl carrier protein | -0.39 | -0.01 | -0.13 | NC | NC | NC | 1 | I | ko:K02160 |
| AIL03682.1 |  | - | PTS system sorbose subIIB component family protein | 0.48 | 0.10 | 0.45 | NC | NC | NC | 1 | G | ko:K02794 |
| AIL03683.1 |  | cspR | tRNA (cytidine(34)-2'-O)-methyltransferase | -0.16 | -0.42 | -0.21 | NC | NC | NC | 1 | J | ko:K03216 |
| AIL03684.1 |  | - | tyrosine phosphatase family protein | -0.73 | -0.30 | -0.16 | NC | NC | NC | 1 | T | ko:K01104 |
| AIL03688.1 |  | aatB | polar amino acid ABC uptake transporter substrate binding protein | 0.04 | -0.55 | 0.60 | NC | NC | NC | 1 | ET | ko:K02030 |
| AIL03689.1 |  | rnhB | ribonuclease HII family protein | -0.73 | -0.27 | -0.26 | NC | NC | NC | 1 | L | ko:K03470 |
| AIL03690.1 |  | kdgK | pfkB carbohydrate kinase family protein | 0.34 | -0.34 | 0.89 | NC | NC | NC | 1 | G | ko:K00874 |
| AIL03691.1 |  | uvrB | excinuclease ABC subunit B | 0.49 | -0.06 | 0.14 | NC | NC | NC | 1 | L | ko:K03702 |
| AIL03692.1 |  | levR | sigma-54 interaction domain protein | 0.05 | 0.09 | -0.14 | NC | NC | NC | 1 | K | - |
| AIL03693.1 |  | metN | methionine import ATP-binding protein MetN 2 | 0.61 | 0.85 | 0.72 | NC | NC | NC | 1 | P | ko:K02071 |
| AIL03694.1 |  | thiM | hydroxyethylthiazole kinase | 0.55 | 0.86 | 0.64 | NC | NC | NC | 1 | H | ko:K00878 |
| AIL03697.1 |  | tagB | Poly(glycerophosphate) glycerophosphotransferase family protein | -0.22 | -0.27 | -0.41 | NC | NC | NC | 1 | M | ko:K09809 |
| AIL03699.1 |  | - | NADPH-dependent FMN reductase family protein | 0.85 | -0.35 | -0.53 | NC | NC | NC | 1 | S | - |
| AIL03700.1 |  | upp | uracil phosphoribosyltransferase | -0.18 | -0.16 | -0.15 | NC | NC | NC | 1 | F | ko:K00761 |
| AIL03701.1 |  | - | putative membrane protein | 0.38 | 0.01 | 0.25 | NC | NC | NC | 1 | - | - |
| AIL03703.1 |  | - | putative gGDEF domain protein | -0.04 | 0.16 | 0.01 | NC | NC | NC | 1 | T | - |
| AIL03704.1 |  | - | phage regulatory, Rha family protein | 0.99 | 3.60 | 0.82 | NC | U | NC | 15 | S | - |
| AIL03705.1 |  | pepQ | metallopeptidase M24 family protein | 0.17 | 0.23 | 0.14 | NC | NC | NC | 1 | E | ko:K01262 |
| AIL03709.1 |  | - | alpha/beta hydrolase fold family protein | -0.61 | -0.34 | -0.52 | NC | NC | NC | 1 | I | - |
| AIL03712.1 |  | pbpX | PASTA domain protein | 0.09 | -0.11 | -0.01 | NC | NC | NC | 1 | M | ko:K08724 |
| AIL03713.1 |  | XK27_10035 | ABC transporter family protein | 0.08 | -0.19 | -0.09 | NC | NC | NC | 1 | V | ko:K06147 |
| AIL03714.1 |  | - | metallopeptidase M24 family protein | -0.08 | -0.05 | 0.17 | NC | NC | NC | 1 | E | - |
| AIL03715.1 |  | glxI | glyoxalase-like domain protein | 0.59 | 0.00 | 0.15 | NC | NC | NC | 1 | E | ko:K01759 |
| AIL03716.1 |  | asp1 | response regulator | 0.82 | 0.10 | 0.05 | NC | NC | NC | 1 | S | - |
| AIL03718.1 |  | - | merR regulatory family protein | 0.69 | 1.15 | 0.50 | NC | U | NC | 15 | K | - |
| AIL03719.1 |  | nadD | nicotinate (nicotinamide) nucleotide adenylyltransferase | -0.25 | -1.19 | -0.90 | NC | D | NC | 23 | H | ko:K00969 |
| AIL03721.1 |  | - | 3-demethylubiquinone-9 3-methyltransferase family protein | -0.36 | 1.07 | -0.21 | NC | U | NC | 15 | S | - |
| AIL03722.1 |  | gdhA | glu/Leu/Phe/Val dehydrogenase, dimerization domain protein | -0.48 | -0.14 | -0.06 | NC | NC | NC | 1 | E | ko:K00262 |
| AIL03723.1 |  | ndh | FAD dependent oxidoreductase family protein | -0.35 | -0.68 | -0.49 | NC | NC | NC | 1 | C | ko:K03885 |
| AIL03725.1 |  | apt | adenine phosphoribosyltransferase | -0.17 | -0.03 | 0.00 | NC | NC | NC | 1 | F | ko:K00759 |
| AIL03726.1 |  | gltX | glutamate--tRNA ligase | 0.26 | 0.15 | 0.05 | NC | NC | NC | 1 | J | ko:K09698 |
| AIL03727.1 |  | copY | copper transport repressor, CopY/TcrY family | -2.60 | -1.69 | -13.29 | D | D | D | 3 | K | ko:K02171 |
| AIL03728.1 |  | opuCB | binding--dependent transport system inner membrane component family protein | -1.05 | -0.14 | -0.75 | D | NC | NC | 6 | E | ko:K05846 |
| AIL03730.1 |  | ywnA | putative HTH-type transcriptional regulator ywnA | 0.87 | -0.53 | -1.00 | NC | NC | NC | 1 | K | - |
| AIL03732.1 |  | rimI | ribosomal-protein-alanine acetyltransferase | 0.32 | 0.11 | -0.30 | NC | NC | NC | 1 | K | ko:K03789 |
| AIL03735.1 |  | WQ51_02665 | hypothetical protein DR75_1475 | 0.69 | -0.18 | 0.42 | NC | NC | NC | 1 | S | - |
| AIL03736.1 |  | recG | ATP-dependent DNA helicase RecG | 0.64 | 0.14 | -0.29 | NC | NC | NC | 1 | L | ko:K03655 |
| AIL03737.1 |  | menD | 2-succinyl-5-enolpyruvyl-6-hydroxy-3-cyclohexene-1-carboxylic-acid synthase | 0.69 | -0.22 | 0.39 | NC | NC | NC | 1 | H | ko:K02551 |
| AIL03739.1 |  | - | bacterial regulatory helix-turn-helix, lysR family protein | 0.34 | 0.16 | 0.23 | NC | NC | NC | 1 | K | - |
| AIL03740.1 |  | copY | copper transport repressor, CopY/TcrY family | -0.16 | 0.02 | 0.21 | NC | NC | NC | 1 | K | ko:K02171 |
| AIL03741.1 |  | rplM | ribosomal protein L13 | -0.21 | -0.17 | -0.25 | NC | NC | NC | 1 | J | ko:K02871 |
| AIL03742.1 |  | - | merR regulatory family protein | 0.31 | 0.60 | 0.25 | NC | NC | NC | 1 | K | - |
| AIL03745.1 |  | - | DNA (cytosine-5-)-methyltransferase family protein | 1.35 | 1.45 | 0.43 | U | U | NC | 9 | H | ko:K00558 |
| AIL03746.1 |  | - | hypothetical protein DR75_1892 | 0.35 | -0.08 | -0.16 | NC | NC | NC | 1 | S | - |
| AIL03747.1 |  | - | glycosyl transferase 2 family protein | -0.82 | 0.07 | -0.04 | NC | NC | NC | 1 | M | ko:K19425 |
| AIL03750.1 |  | - | hypothetical protein DR75_2222 | -1.33 | -0.06 | -0.02 | D | NC | NC | 6 | L | - |
| AIL03751.1 |  | - | sortase family protein | 0.25 | 0.22 | -0.82 | NC | NC | NC | 1 | M | ko:K07284 |
| AIL03752.1 |  | thrC | threonine synthase | 0.22 | 0.04 | -0.01 | NC | NC | NC | 1 | E | ko:K01733 |
| AIL03755.1 |  | oppA | bacterial extracellular solute-binding s, 5 Middle family protein | 0.22 | -0.26 | -0.24 | NC | NC | NC | 1 | E | ko:K02035 |
| AIL03757.1 |  | ilvE | branched-chain amino acid aminotransferase | -0.06 | 0.06 | -0.03 | NC | NC | NC | 1 | E | ko:K00826 |
| AIL03758.1 |  | potD3 | bacterial extracellular solute-binding family protein | -0.53 | -0.04 | 0.38 | NC | NC | NC | 1 | E | ko:K02055 |
| AIL03759.1 |  | - | siphovirus Gp157 family protein | 1.20 | -0.25 | -0.11 | U | NC | NC | 11 | S | - |
| AIL03761.1 |  | yjqA | bacterial PH domain protein | 0.11 | 0.88 | 0.64 | NC | NC | NC | 1 | S | - |
| AIL03762.1 |  | - | hypothetical protein DR75_2390 | -0.38 | -0.04 | -0.39 | NC | NC | NC | 1 | S | - |
| AIL03763.1 |  | lysC | aspartate kinase domain protein | 0.25 | -0.27 | -0.16 | NC | NC | NC | 1 | E | ko:K00928 |
| AIL03764.1 |  | yrxA | 3H domain protein | 0.30 | 0.05 | 0.23 | NC | NC | NC | 1 | S | ko:K07105 |
| AIL03766.1 |  | ydjH | hypothetical protein DR75_1227 | 0.70 | 0.35 | 0.33 | NC | NC | NC | 1 | S | ko:K06872 |
| AIL03767.1 |  | tagH | lysM domain protein | 0.06 | -0.61 | -0.87 | NC | NC | NC | 1 | GM | ko:K09693 |
| AIL03768.1 |  | - | hypothetical protein DR75_1648 | 0.35 | 0.08 | -0.21 | NC | NC | NC | 1 | - | - |
| AIL03769.1 |  | zur | zinc-specific metallo-regulatory protein | -0.24 | 0.75 | -0.04 | NC | NC | NC | 1 | P | ko:K02076 |
| AIL03770.1 |  | carB | carbamoyl-phosphate synthase, large subunit | -1.06 | 0.67 | 0.59 | D | NC | NC | 6 | EF | ko:K01955 |
| AIL03773.1 |  | yeaO | hypothetical protein DR75_1477 | -0.03 | 0.09 | -0.25 | NC | NC | NC | 1 | S | - |
| AIL03774.1 |  | - | putative transcriptional regulator | 0.01 | 1.33 | 1.15 | NC | U | U | 4 | T | - |
| AIL03775.1 |  | - | sulfatase family protein | -0.39 | 0.05 | -0.70 | NC | NC | NC | 1 | M | - |
| AIL03777.1 |  | - | hypothetical protein DR75_2657 | -0.41 | 0.10 | -0.05 | NC | NC | NC | 1 | - | - |
| AIL03779.1 |  | rpoE | putative DNA-directed RNA polymerase subunit delta | -0.27 | -0.83 | -0.55 | NC | NC | NC | 1 | K | ko:K03048 |
| AIL03783.1 |  | - | YGGT family protein | -0.33 | -0.26 | 0.00 | NC | NC | NC | 1 | S | ko:K02221 |
| AIL03784.1 |  | yvcJ | P-loop ATPase family protein | 0.64 | 0.01 | 0.03 | NC | NC | NC | 1 | S | ko:K06958 |
| AIL03785.1 |  | dnaD | DnaD domain protein | -0.43 | -0.52 | -0.48 | NC | NC | NC | 1 | L | ko:K02086 |
| AIL03787.1 |  | rpsJ | ribosomal protein S10 | -0.61 | -0.47 | -0.60 | NC | NC | NC | 1 | J | ko:K02946 |
| AIL03791.1 |  | rnz | ribonuclease Z | -0.15 | -0.22 | -0.24 | NC | NC | NC | 1 | S | ko:K00784 |
| AIL03793.1 |  | - | response regulator | -0.23 | 0.01 | -0.29 | NC | NC | NC | 1 | K | - |
| AIL03796.1 |  | rnc | ribonuclease III | -0.37 | -0.04 | -0.25 | NC | NC | NC | 1 | J | ko:K03685 |
| AIL03797.1 |  | aroB | 3-dehydroquinate synthase | -1.13 | -0.65 | -0.70 | D | NC | NC | 6 | E | ko:K01735 |
| AIL03798.1 |  | rplV | ribosomal protein L22 | -0.12 | 0.16 | -0.06 | NC | NC | NC | 1 | J | ko:K02890 |
| AIL03799.1 |  | msrA | peptide-methionine (S)-S-oxide reductase | -0.39 | 0.31 | 0.03 | NC | NC | NC | 1 | O | ko:K07304 |
| AIL03802.1 |  | pyrC | dihydroorotase | 0.00 | 0.66 | -0.40 | NC | NC | NC | 1 | F | ko:K01465 |
| AIL03803.1 |  | metE | cobalamin-independent synthase, Catalytic domain protein | -0.71 | -0.43 | -0.10 | NC | NC | NC | 1 | E | ko:K00549 |
| AIL03805.1 |  | - | hypothetical protein DR75_613 | -0.03 | -0.25 | -0.18 | NC | NC | NC | 1 | S | - |
| AIL03807.1 |  | uxuA | mannonate dehydratase | 0.46 | 1.36 | 1.14 | NC | U | U | 4 | G | ko:K01686 |
| AIL03808.1 |  | - | helix-turn-helix domain protein | 0.00 | 0.39 | -0.21 | NC | NC | NC | 1 | KT | ko:K07720 |
| AIL03809.1 |  | coaB | phosphopantothenate--cysteine ligase | 0.23 | 0.00 | 0.20 | NC | NC | NC | 1 | H | ko:K21977 |
| AIL03811.1 |  | ytkL | metallo-beta-lactamase superfamily protein | 0.31 | 0.42 | 0.49 | NC | NC | NC | 1 | S | - |
| AIL03812.1 |  | recU | recombination protein U | -0.60 | -0.23 | 0.13 | NC | NC | NC | 1 | L | ko:K03700 |
| AIL03815.1 |  | polC | DNA polymerase III, alpha subunit, Gram-positive type | 0.67 | 0.35 | 0.35 | NC | NC | NC | 1 | L | ko:K03763 |
| AIL03816.1 |  | yvyE | hypothetical protein DR75_767 | 3.72 | 3.56 | 3.30 | U | U | U | 2 | S | ko:K01271 |
| AIL03817.1 |  | rpiA | ribose 5-phosphate isomerase A | -0.28 | 0.13 | -0.10 | NC | NC | NC | 1 | G | ko:K01807 |
| AIL03819.1 |  | - | glycosyl hydrolases 25 family protein | 0.32 | -0.03 | 0.17 | NC | NC | NC | 1 | M | - |
| AIL03820.1 |  | ptsI | phosphoenolpyruvate-protein phosphotransferase | -0.31 | -0.15 | -0.22 | NC | NC | NC | 1 | G | ko:K08483 |
| AIL03823.1 |  | - | hypothetical protein DR75_1646 | 0.85 | 2.23 | 0.71 | NC | U | NC | 15 | - | - |
| AIL03824.1 |  | - | hypothetical protein DR75_2783 | -1.38 | -1.07 | -1.26 | D | D | D | 3 | S | ko:K09768 |
| AIL03825.1 |  | nagA | N-acetylglucosamine-6-phosphate deacetylase | 0.04 | 0.19 | 0.05 | NC | NC | NC | 1 | G | ko:K01443 |
| AIL03827.1 |  | - | glycine-rich SFCGS family protein | -0.27 | 1.34 | 1.05 | NC | U | U | 4 | S | - |
| AIL03828.1 |  | mtlR | deoR-like helix-turn-helix domain protein | -0.24 | -0.20 | 0.29 | NC | NC | NC | 1 | K | ko:K03483 |
| AIL03829.1 |  | tpiA | triose-phosphate isomerase | 0.12 | 0.11 | -0.16 | NC | NC | NC | 1 | G | ko:K01803 |
| AIL03830.1 |  | yvcK | hypothetical protein DR75_2720 | 0.51 | 0.05 | 0.17 | NC | NC | NC | 1 | S | - |
| AIL03831.1 |  | recR | recombination protein RecR | 0.40 | -0.08 | 0.02 | NC | NC | NC | 1 | L | ko:K06187 |
| AIL03832.1 |  | yjbO | pseudouridine synthase, RluA family protein | -0.50 | -0.29 | -0.40 | NC | NC | NC | 1 | J | ko:K06177 |
| AIL03834.1 |  | yitU | HAD hydrolase, IIB family protein | -0.27 | 0.41 | 0.32 | NC | NC | NC | 1 | S | ko:K21064 |
| AIL03837.1 |  | - | acetyltransferase family protein | -0.35 | 0.63 | -1.26 | NC | NC | D | 19 | K | - |
| AIL03839.1 |  | queA | tRNA ribosyltransferase-isomerase | 0.24 | 0.01 | -0.02 | NC | NC | NC | 1 | J | ko:K07568 |
| AIL03840.1 |  | coaBC | phosphopantothenoylcysteine decarboxylase | 0.46 | -0.23 | -0.21 | NC | NC | NC | 1 | H | ko:K01598 |
| AIL03841.1 |  | - | PTS system mannose/fructose/sorbose IID component family protein | 0.12 | -0.51 | -0.61 | NC | NC | NC | 1 | G | ko:K02796 |
| AIL03842.1 |  | - | endo-beta-N-acetylglucosaminidase H | -1.38 | 0.27 | -0.57 | D | NC | NC | 6 | G | - |
| AIL03843.1 |  | yvlB | hypothetical protein DR75_751 | 0.41 | 0.30 | 0.28 | NC | NC | NC | 1 | D | - |
| AIL03844.1 |  | ytlR | lipid kinase, YegS//BmrU family protein | 0.30 | 0.08 | -0.19 | NC | NC | NC | 1 | I | - |
| AIL03846.1 |  | - | universal stress family protein | -0.03 | -0.49 | -0.34 | NC | NC | NC | 1 | T | - |
| AIL03847.1 |  | purR | pur operon repressor PurR | -0.56 | -0.11 | -0.18 | NC | NC | NC | 1 | F | ko:K09685 |
| AIL03848.1 |  | murQ | N-acetylmuramic acid 6-phosphate etherase | -0.30 | 0.25 | 0.41 | NC | NC | NC | 1 | G | ko:K07106 |
| AIL03849.1 |  | - | NADPH-dependent nitro/flavin reductase | 0.50 | -0.06 | 0.18 | NC | NC | NC | 1 | C | - |
| AIL03850.1 |  | bglC | aryl-phospho-beta-D-glucosidase BglC | 0.27 | 0.26 | 0.52 | NC | NC | NC | 1 | G | ko:K01223 |
| AIL03851.1 |  | mfd | transcription-repair coupling factor | 0.42 | 0.07 | -0.09 | NC | NC | NC | 1 | L | ko:K03723 |
| AIL03852.1 |  | recD2 | viral (Super1) RNA helicase family protein | -0.30 | -0.50 | -0.26 | NC | NC | NC | 1 | L | ko:K03581 |
| AIL03855.1 |  | pfkB | 1-phosphofructokinase | -0.11 | 0.26 | 0.24 | NC | NC | NC | 1 | H | ko:K00882 |
| AIL03858.1 |  | norG_2 | bacterial regulatory s, gntR family protein | 0.18 | 0.83 | -0.21 | NC | NC | NC | 1 | K | - |
| AIL03859.1 |  | relA | RelA/SpoT family protein | 0.04 | -0.11 | -0.18 | NC | NC | NC | 1 | KT | ko:K00951 |
| AIL03861.1 |  | dnaI | istB-like ATP binding family protein | 0.01 | -0.34 | -0.11 | NC | NC | NC | 1 | L | ko:K11144 |
| AIL03864.1 |  | ykuP | flavodoxin | -0.39 | 0.71 | 0.33 | NC | NC | NC | 1 | C | ko:K03839 |
| AIL03865.1 |  | coaA | pantothenate kinase | -0.42 | -0.96 | -0.15 | NC | NC | NC | 1 | F | ko:K00867 |
| AIL03868.1 |  | ypsC | THUMP domain protein | -0.07 | 0.11 | 0.09 | NC | NC | NC | 1 | L | ko:K07444 |
| AIL03871.1 |  | - | ABC transporter family protein | 0.15 | 0.03 | -0.50 | NC | NC | NC | 1 | V | ko:K11635 |
| AIL03872.1 |  | trpS | tryptophan--tRNA ligase | -0.09 | 0.74 | 0.04 | NC | NC | NC | 1 | J | ko:K01867 |
| AIL03873.1 |  | rluA | pseudouridine synthase, RluA family protein | -0.25 | -0.13 | -0.36 | NC | NC | NC | 1 | J | ko:K06177 |
| AIL03874.1 |  | aroA | 3-phosphoshikimate 1-carboxyvinyltransferase | -1.10 | 0.25 | -0.63 | D | NC | NC | 6 | E | ko:K00800 |
| AIL03875.1 |  | pfoS/R | phosphotransferase system, EIIC family protein | -0.59 | -1.52 | -0.56 | NC | D | NC | 23 | S | ko:K07035 |
| AIL03877.1 |  | rnmV | ribonuclease M5 | -0.07 | -0.42 | 1.49 | NC | NC | U | 5 | J | ko:K05985 |
| AIL03878.1 |  | - | putative membrane protein | 0.51 | -0.51 | -0.44 | NC | NC | NC | 1 | - | - |
| AIL03879.1 |  | phoH | phoH-like family protein | -0.71 | -0.23 | -0.25 | NC | NC | NC | 1 | T | ko:K06217 |
| AIL03880.1 |  | pgi | phosphoglucose isomerase family protein | 0.12 | 0.04 | 0.03 | NC | NC | NC | 1 | G | ko:K01810 |
| AIL03883.1 |  | prsA | PPIC-type PPIASE domain protein | -0.17 | -0.19 | -0.45 | NC | NC | NC | 1 | M | ko:K01802 |
| AIL03886.1 |  | albE | hypothetical protein DR75_1869 | 0.22 | 0.41 | 0.12 | NC | NC | NC | 1 | S | - |
| AIL03887.1 |  | gmuD | 6-phospho-beta-glucosidase gmuD | 0.29 | 0.35 | 0.23 | NC | NC | NC | 1 | G | ko:K01223 |
| AIL03888.1 |  | rpsO | ribosomal protein S15 | -0.31 | -0.37 | -0.45 | NC | NC | NC | 1 | J | ko:K02956 |
| AIL03890.1 |  | rarA | AAA ATPase, central region | 0.90 | 0.10 | -0.04 | NC | NC | NC | 1 | O | ko:K07478 |
| AIL03892.1 |  | tdc | tyrosine decarboxylase | -0.14 | -0.51 | -0.18 | NC | NC | NC | 1 | E | ko:K22330 |
| AIL03894.1 |  | dnaQ | exonuclease, DNA polymerase III, epsilon subunit family domain protein | -0.90 | -1.42 | -1.38 | NC | D | D | 22 | L | ko:K02342 |
| AIL03897.1 |  | - | hypothetical protein DR75_1590 | -0.21 | -0.45 | -0.32 | NC | NC | NC | 1 | - | - |
| AIL03899.1 |  | - | GDSL-like Lipase/Acylhydrolase family protein | 0.98 | 0.33 | 0.50 | NC | NC | NC | 1 | E | - |
| AIL03900.1 |  | mtnN | MTA/SAH nucleosidase | 0.53 | 0.25 | 0.04 | NC | NC | NC | 1 | E | ko:K01243 |
| AIL03902.1 |  | - | hypothetical protein DR75_2375 | -0.40 | 0.19 | -0.37 | NC | NC | NC | 1 | QT | ko:K09684 |
| AIL03905.1 |  | glnQ | ABC transporter family protein | -0.54 | -0.12 | -0.25 | NC | NC | NC | 1 | E | ko:K02028 |
| AIL03906.1 |  | est | esterase D | 0.22 | 0.00 | -0.11 | NC | NC | NC | 1 | S | ko:K03928 |
| AIL03907.1 |  | atpA | ATP synthase F1, alpha subunit | -0.28 | -0.03 | -0.32 | NC | NC | NC | 1 | C | ko:K02111 |
| AIL03909.1 |  | infC | translation initiation factor IF-3 | -0.62 | -0.37 | -0.62 | NC | NC | NC | 1 | J | ko:K02520 |
| AIL03910.1 |  | XK27_04120 | amino acid metabolism family protein | 0.32 | 0.25 | 0.32 | NC | NC | NC | 1 | S | - |
| AIL03912.1 |  | fabF | beta-ketoacyl-acyl-carrier-protein synthase II | -0.39 | 0.05 | 0.20 | NC | NC | NC | 1 | I | ko:K09458 |
| AIL03916.1 |  | pyrG | CTP synthase | -0.29 | -0.08 | -0.06 | NC | NC | NC | 1 | F | ko:K01937 |
| AIL03917.1 |  | ebsA | putative ebsA protein | 0.77 | 0.29 | 0.05 | NC | NC | NC | 1 | S | - |
| AIL03918.1 |  | - | acetyltransferase family protein | 0.50 | 0.84 | 0.81 | NC | NC | NC | 1 | S | ko:K03824 |
| AIL03919.1 |  | pepC | peptidase C1-like family protein | 0.02 | 0.17 | 0.15 | NC | NC | NC | 1 | E | ko:K01372 |
| AIL03921.1 |  | murF | UDP-N-acetylmuramoyl-tripeptide--D-alanyl-D-alanine ligase family protein | -0.34 | -0.31 | -0.01 | NC | NC | NC | 1 | M | ko:K01929 |
| AIL03922.1 |  | - | cell division ZapA family protein | -0.43 | -0.38 | -0.18 | NC | NC | NC | 1 | D | ko:K09888 |
| AIL03923.1 |  | - | peptidase M48 family protein | -0.25 | 0.11 | -0.03 | NC | NC | NC | 1 | O | - |
| AIL03924.1 |  | ssb | single-stranded DNA-binding family protein | 0.03 | -0.49 | -0.15 | NC | NC | NC | 1 | L | ko:K03111 |
| AIL03925.1 |  | - | carbamate kinase | 1.03 | 0.05 | 0.39 | U | NC | NC | 11 | E | ko:K00926 |
| AIL03926.1 |  | cutC | cutC family protein | -0.27 | -0.49 | -0.28 | NC | NC | NC | 1 | P | ko:K06201 |
| AIL03927.1 |  | cad | FMN-binding domain protein | -2.45 | -2.25 | -1.52 | D | D | D | 3 | S | ko:K20379 |
| AIL03928.1 |  | mglA | heme ABC exporter, ATP-binding protein CcmA | -0.14 | 0.18 | 0.13 | NC | NC | NC | 1 | S | ko:K02056 |
| AIL03930.1 |  | - | O-antigen ligase like membrane family protein | -0.75 | -0.87 | -1.21 | NC | NC | D | 19 | M | - |
| AIL03931.1 |  | - | O-Antigen ligase family protein | -0.44 | -0.33 | -0.24 | NC | NC | NC | 1 | M | - |
| AIL03932.1 |  | panE | 2-dehydropantoate 2-reductase family protein | -0.82 | -0.74 | -0.55 | NC | NC | NC | 1 | H | ko:K00077 |
| AIL03933.1 |  | rho | transcription termination factor Rho | -0.01 | 0.19 | 0.12 | NC | NC | NC | 1 | K | ko:K03628 |
| AIL03934.1 |  | yugP | neutral zinc metallopeptidase family protein | -2.14 | -1.76 | -0.71 | D | D | NC | 20 | S | ko:K06973 |
| AIL03935.1 |  | ftsY | signal recognition particle-docking protein FtsY | 0.09 | -0.36 | -0.08 | NC | NC | NC | 1 | U | ko:K03110 |
| AIL03936.1 |  | - | carbamate kinase | -3.60 | -3.77 | -2.02 | D | D | D | 3 | E | ko:K00926 |
| AIL03937.1 |  | rsuA | pseudouridine synthase family protein | 1.01 | -0.06 | -0.21 | U | NC | NC | 11 | J | ko:K06183 |
| AIL03938.1 |  | XK27_05795 | amino ABC transporter, permease, 3-TM region, His/Glu/Gln/Arg/opine family domain protein | 0.07 | -0.27 | -0.23 | NC | NC | NC | 1 | P | ko:K17073 |
| AIL03940.1 |  | fruK-1 | 1-phosphofructokinase | 1.46 | 0.77 | 0.72 | U | NC | NC | 11 | H | ko:K00882 |
| AIL03941.1 |  | cspB | cold shock protein CspD | 0.28 | 1.07 | 1.19 | NC | U | U | 4 | K | ko:K03704 |
| AIL03942.1 |  | truB | tRNA pseudouridine(55) synthase | -0.20 | -0.20 | -0.40 | NC | NC | NC | 1 | J | ko:K03177 |
| AIL03943.1 |  | dho | hypothetical protein DR75_2777 | 1.07 | -0.71 | 1.43 | U | NC | U | 14 | S | ko:K01465 |
| AIL03945.1 |  | - | glycosyl transferase 2 family protein | -0.19 | -0.36 | -0.04 | NC | NC | NC | 1 | M | - |
| AIL03946.1 |  | yumC | ferredoxin--NADP reductase 2 | -0.21 | -0.18 | -0.20 | NC | NC | NC | 1 | C | ko:K21567 |
| AIL03949.1 |  | - | hypothetical protein DR75_1756 | 0.92 | 0.53 | 0.61 | NC | NC | NC | 1 | S | - |
| AIL03950.1 |  | pvaA | lysozyme-like family protein | 0.06 | -0.87 | -0.90 | NC | NC | NC | 1 | M | - |
| AIL03952.1 |  | - | ABC transporter family protein | 0.81 | -0.47 | -0.83 | NC | NC | NC | 1 | V | ko:K06147 |
| AIL03953.1 |  | - | ftsX-like permease family protein | 0.34 | 0.70 | -0.17 | NC | NC | NC | 1 | V | ko:K02004 |
| AIL03956.1 |  | lplA | lipoyltransferase and lipoate-ligase family protein | -0.12 | 0.11 | 0.00 | NC | NC | NC | 1 | H | ko:K03800 |
| AIL03957.1 |  | - | UTRA domain protein | 0.46 | 0.10 | 0.15 | NC | NC | NC | 1 | K | - |
| AIL03958.1 |  | ptsB | putative phosphotransferase enzyme IIB component | 0.32 | 1.44 | 1.48 | NC | U | U | 4 | G | ko:K02794 |
| AIL03959.1 |  | ctc | ribosomal protein L25, Ctc-form | 0.92 | 0.27 | 0.43 | NC | NC | NC | 1 | J | ko:K02897 |
| AIL03962.1 |  | - | ABC transporter family protein | -0.14 | 0.82 | -0.07 | NC | NC | NC | 1 | P | ko:K02071 |
| AIL03963.1 |  | - | transcriptional regulator, GntR family | 0.35 | -0.06 | 0.14 | NC | NC | NC | 1 | K | - |
| AIL03968.1 |  | ybfQ | rhodanese-like domain protein | -1.69 | -0.07 | -0.47 | D | NC | NC | 6 | S | ko:K07146 |
| AIL03970.1 |  | - | PTS system sorbose subIIB component family protein | 0.10 | 1.38 | -0.45 | NC | U | NC | 15 | G | ko:K17465 |
| AIL03972.1 |  | - | hypothetical protein DR75_308 | 0.13 | 0.10 | 0.16 | NC | NC | NC | 1 | S | - |
| AIL03975.1 |  | - | bacterial transcriptional regulator family protein | 0.04 | -0.14 | -0.50 | NC | NC | NC | 1 | K | ko:K19333 |
| AIL03976.1 |  | nrdI | nrdI protein | 0.78 | 0.09 | 0.09 | NC | NC | NC | 1 | F | ko:K03647 |
| AIL03977.1 |  | dnaA | chromosomal replication initiator protein DnaA | 0.09 | 0.02 | -0.13 | NC | NC | NC | 1 | L | ko:K02313 |
| AIL03980.1 |  | malR | helix-turn-helix family protein | -0.21 | -0.08 | -0.25 | NC | NC | NC | 1 | K | ko:K02529 |
| AIL03981.1 |  | - | crp-like helix-turn-helix domain protein | -0.56 | -0.07 | -0.17 | NC | NC | NC | 1 | K | - |
| AIL03982.1 |  | - | SIS domain protein | -0.85 | -0.53 | -0.48 | NC | NC | NC | 1 | G | ko:K19510 |
| AIL03983.1 |  | ahpC | peroxiredoxin | -1.96 | -1.21 | -0.97 | D | D | NC | 20 | O | ko:K03386 |
| AIL03985.1 |  | yfiC | ABC transporter family protein | 0.21 | 0.29 | 0.03 | NC | NC | NC | 1 | V | ko:K06147 |
| AIL03987.1 |  | - | nucleotidyltransferase domain protein | 0.84 | 0.49 | 0.68 | NC | NC | NC | 1 | S | ko:K07075 |
| AIL03988.1 |  | hemN | radical SAM superfamily protein | -0.28 | -0.19 | -0.39 | NC | NC | NC | 1 | H | - |
| AIL03989.1 |  | mntH | metal ion transporter, metal ion family protein | 0.33 | -0.20 | 0.39 | NC | NC | NC | 1 | U | ko:K03322 |
| AIL03990.1 |  | rpsF | ribosomal protein S6 | -0.12 | -0.01 | -0.25 | NC | NC | NC | 1 | J | ko:K02990 |
| AIL03991.1 |  | deoA | pyrimidine-nucleoside phosphorylase | 0.10 | 0.20 | 0.36 | NC | NC | NC | 1 | F | ko:K00756 |
| AIL03994.1 |  | - | hypothetical protein DR75_905 | 0.04 | 0.68 | 0.42 | NC | NC | NC | 1 | S | - |
| AIL03995.1 |  | lacD | tagatose 1,6-diphosphate aldolase | 0.57 | 0.54 | 0.39 | NC | NC | NC | 1 | G | ko:K01635 |
| AIL03997.1 |  | - | hypothetical protein DR75_156 | 1.99 | 3.31 | 3.47 | U | U | U | 2 | - | - |
| AIL03998.1 |  | xerC | tyrosine recombinase XerC | -0.06 | 0.03 | -0.27 | NC | NC | NC | 1 | D | ko:K03733 |
| AIL04000.1 |  | - | spermine/spermidine acetyltransferase | 1.65 | 0.62 | -0.52 | U | NC | NC | 11 | J | ko:K00657 |
| AIL04002.1 |  | metQ3 | NLPA lipofamily protein | 0.63 | 1.32 | 0.85 | NC | U | NC | 15 | P | ko:K02073 |
| AIL04003.1 |  | tig | trigger factor | -0.22 | -0.05 | -0.19 | NC | NC | NC | 1 | D | ko:K03545 |
| AIL04005.1 |  | - | hypothetical protein DR75_1526 | 0.68 | -0.49 | -0.07 | NC | NC | NC | 1 | S | - |
| AIL04007.1 |  | - | bacterial regulatory s, tetR family protein | -0.54 | -0.97 | 0.30 | NC | NC | NC | 1 | K | - |
| AIL04008.1 |  | nhaC | Na+/H+ antiporter NhaC | -0.87 | -1.08 | -0.86 | NC | D | NC | 23 | C | ko:K03315 |
| AIL04009.1 |  | fabZ | beta-hydroxyacyl-(acyl-carrier-protein) dehydratase FabZ | -0.66 | -0.24 | -0.42 | NC | NC | NC | 1 | I | ko:K02372 |
| AIL04012.1 |  | spoU | RNA 2'-O ribose methyltransferase substrate binding family protein | 0.33 | 0.37 | 0.00 | NC | NC | NC | 1 | J | ko:K03437 |
| AIL04013.1 |  | folK | 2-amino-4-hydroxy-6-hydroxymethyldihydropteridine diphosphokinase | -0.56 | -0.94 | -0.40 | NC | NC | NC | 1 | H | ko:K00950 |
| AIL04016.1 |  | - | 2-dehydro-3-deoxyphosphogluconate aldolase/4-hydroxy-2-oxoglutarate aldolase family protein | 0.80 | 0.08 | 0.07 | NC | NC | NC | 1 | G | ko:K01625 |
| AIL04024.1 |  | - | hypothetical protein DR75_2870 | -0.11 | -0.47 | 0.22 | NC | NC | NC | 1 | L | ko:K03657 |
| AIL04026.1 |  | - | hypothetical protein DR75_1500 | 0.24 | -0.79 | -0.91 | NC | NC | NC | 1 | S | - |
| AIL04027.1 |  | - | beta-eliminating lyase family protein | 0.01 | 0.20 | -0.17 | NC | NC | NC | 1 | - | - |
| AIL04028.1 |  | yitL | S1 domain protein | 0.72 | 0.35 | 0.34 | NC | NC | NC | 1 | S | ko:K00243 |
| AIL04029.1 |  | oppB | binding--dependent transport system inner membrane component family protein | 0.74 | 0.11 | -0.15 | NC | NC | NC | 1 | EP | ko:K02033 |
| AIL04030.1 |  | yfmR | heme ABC exporter, ATP-binding protein CcmA | -0.64 | 0.21 | -0.13 | NC | NC | NC | 1 | S | ko:K15738 |
| AIL04032.1 |  | menA | ubiA prenyltransferase family protein | 4.23 | 0.56 | 0.49 | U | NC | NC | 11 | H | ko:K02548 |
| AIL04033.1 |  | acpS | holo-[acyl-carrier-protein] synthase | 0.85 | 0.48 | 1.16 | NC | NC | U | 5 | I | ko:K00997 |
| AIL04035.1 |  | gnd | 6-phosphogluconate dehydrogenase | -0.07 | 0.00 | 0.07 | NC | NC | NC | 1 | H | ko:K00033 |
| AIL04036.1 |  | - | beta-phosphoglucomutase | 0.03 | 0.19 | -0.03 | NC | NC | NC | 1 | S | ko:K01838 |
| AIL04039.1 |  | - | periplasmic binding s and sugar binding domain of LacI family protein | 0.61 | 0.09 | 0.18 | NC | NC | NC | 1 | K | ko:K02529 |
| AIL04040.1 |  | aha1 | HAD ATPase, P-type, IC family protein | 0.01 | -0.11 | -0.52 | NC | NC | NC | 1 | P | ko:K12952 |
| AIL04041.1 |  | mgtE | magnesium transporter | 0.15 | -0.43 | -0.49 | NC | NC | NC | 1 | P | ko:K06213 |
| AIL04042.1 |  | cvpA | colicin V production family protein | -1.59 | -1.32 | -0.70 | D | D | NC | 20 | S | - |
| AIL04044.1 |  | - | hypothetical protein DR75_1100 | 0.13 | 0.31 | 0.28 | NC | NC | NC | 1 | S | - |
| AIL04045.1 |  | aspS | aspartate--tRNA ligase | -0.23 | -0.12 | -0.12 | NC | NC | NC | 1 | J | ko:K01876 |
| AIL04048.1 |  | atpC | ATP synthase F1, epsilon subunit | -0.29 | -0.49 | -0.27 | NC | NC | NC | 1 | C | ko:K02114 |
| AIL04050.1 |  | yloU | asp23 family protein | 0.13 | -0.14 | 0.10 | NC | NC | NC | 1 | S | - |
| AIL04051.1 |  | yebE | hypothetical protein DR75_1557 | -0.17 | 0.00 | -0.47 | NC | NC | NC | 1 | S | - |
| AIL04052.1 |  | mprF | hypothetical protein DR75_87 | -0.19 | 0.86 | 0.52 | NC | NC | NC | 1 | S | ko:K14205 |
| AIL04057.1 |  | vraR | bacterial regulatory s, luxR family protein | 0.23 | 0.08 | -0.09 | NC | NC | NC | 1 | K | ko:K07694 |
| AIL04058.1 |  | dhaK | dihydroxyacetone kinase, DhaK subunit | 0.77 | 0.93 | 0.94 | NC | NC | NC | 1 | G | ko:K05878 |
| AIL04059.1 |  | - | acetyltransferase family protein | -0.82 | 1.18 | 0.63 | NC | U | NC | 15 | J | - |
| AIL04060.1 |  | rfbD | dTDP-4-dehydrorhamnose reductase | -0.24 | -0.03 | -0.11 | NC | NC | NC | 1 | M | ko:K00067 |
| AIL04061.1 |  | yqjQ | short chain dehydrogenase family protein | 0.00 | -0.11 | -0.23 | NC | NC | NC | 1 | S | ko:K07124 |
| AIL04062.1 |  | - | linear amide C-N hydrolase, choloylglycine hydrolase family protein | 0.54 | 0.76 | 0.63 | NC | NC | NC | 1 | M | ko:K01442 |
| AIL04063.1 |  | exoA | exodeoxyribonuclease III | 0.04 | 0.06 | -0.03 | NC | NC | NC | 1 | L | ko:K01142 |
| AIL04064.1 |  | accD | acetyl-CoA carboxylase, carboxyl transferase, beta subunit | -0.05 | 0.04 | -0.13 | NC | NC | NC | 1 | I | ko:K01963 |
| AIL04065.1 |  | pyc | pyruvate carboxylase | -0.23 | 0.20 | 0.08 | NC | NC | NC | 1 | C | ko:K01958 |
| AIL04066.1 |  | - | putative membrane protein | -0.31 | 0.46 | -0.29 | NC | NC | NC | 1 | - | - |
| AIL04067.1 |  | XK27_00215 | glyoxalase/Bleomycin resistance /Dioxygenase superfamily protein | 1.14 | 0.86 | 0.66 | U | NC | NC | 11 | E | ko:K15975 |
| AIL04068.1 |  | ypwA | carboxypeptidase Taq (M32) metallopeptidase family protein | 0.17 | -0.18 | -0.11 | NC | NC | NC | 1 | E | ko:K01299 |
| AIL04069.1 |  | bmr3_1 | sugar (and other) transporter family protein | -1.50 | -0.46 | -0.60 | D | NC | NC | 6 | U | ko:K18935 |
| AIL04070.1 |  | rrmA | S-adenosyl-L-methionine-dependent methyltransferase family protein | 0.45 | 0.33 | 0.27 | NC | NC | NC | 1 | Q | ko:K00563 |
| AIL04071.1 |  | soj | sporulation initiation inhibitor protein soj | -0.03 | -0.11 | 0.05 | NC | NC | NC | 1 | D | ko:K03496 |
| AIL04073.1 |  | recX | recX family protein | -0.78 | -0.26 | 0.02 | NC | NC | NC | 1 | S | ko:K03565 |
| AIL04074.1 |  | yqeY | gatB domain protein | -0.05 | -0.34 | -0.33 | NC | NC | NC | 1 | S | ko:K09117 |
| AIL04075.1 |  | nrnA | DHH family protein | -0.18 | 0.19 | 0.06 | NC | NC | NC | 1 | S | ko:K06881 |
| AIL04079.1 |  | ldhB | L-lactate dehydrogenase | 0.31 | -0.31 | -0.04 | NC | NC | NC | 1 | C | ko:K00016 |
| AIL04080.1 |  | sufS | cysteine desulfurase, SufS family protein | -0.49 | 0.32 | 0.03 | NC | NC | NC | 1 | E | ko:K11717 |
| AIL04081.1 |  | gloA | glyoxalase/Bleomycin resistance /Dioxygenase superfamily protein | 1.00 | 0.47 | 0.41 | NC | NC | NC | 1 | E | ko:K01759 |
| AIL04082.1 |  | pgl | lactonase, 7-bladed beta-propeller family protein | 0.06 | 0.49 | 0.23 | NC | NC | NC | 1 | G | ko:K07404 |
| AIL04084.1 |  | rlmH | rRNA large subunit m3Psi methyltransferase RlmH | -0.13 | -0.89 | -0.77 | NC | NC | NC | 1 | J | ko:K00783 |
| AIL04085.1 |  | uvrC | excinuclease ABC subunit C | 0.41 | -0.23 | -0.19 | NC | NC | NC | 1 | L | ko:K03703 |
| AIL04086.1 |  | cggR | putative sugar-binding domain protein | 1.90 | 0.72 | 0.42 | U | NC | NC | 11 | K | ko:K05311 |
| AIL04087.1 |  | hgdC | putative CoA-substrate-specific enzyme activase domain protein | -1.72 | -3.01 | -2.80 | D | D | D | 3 | I | - |
| AIL04088.1 |  | lemA | lemA protein | -0.64 | -0.59 | -0.49 | NC | NC | NC | 1 | S | ko:K03744 |
| AIL04089.1 |  | lrp | bacterial regulatory, Fis family protein | -0.04 | 0.55 | 0.28 | NC | NC | NC | 1 | QT | - |
| AIL04090.1 |  | arpJ | amino ABC transporter, permease, 3-TM region, His/Glu/Gln/Arg/opine family domain protein | 1.60 | 0.40 | 0.32 | U | NC | NC | 11 | U | ko:K02029 |
| AIL04091.1 |  | groS | 10 kDa chaperonin | 0.16 | -0.06 | -0.30 | NC | NC | NC | 1 | O | ko:K04078 |
| AIL04092.1 |  | - | 6-phosphogluconate dehydrogenase | -0.45 | 0.40 | -1.00 | NC | NC | D | 19 | G | ko:K00033 |
| AIL04095.1 |  | punA | purine nucleoside phosphorylase I, inosine and guanosine-specific | 0.19 | 0.30 | 0.15 | NC | NC | NC | 1 | F | ko:K00772 |
| AIL04096.1 |  | - | hypothetical protein DR75_2723 | 1.15 | 0.99 | 0.86 | U | NC | NC | 11 | O | - |
| AIL04097.1 |  | XK27_01785 | hypothetical protein DR75_927 | 0.40 | 0.31 | -0.47 | NC | NC | NC | 1 | S | - |
| AIL04100.1 |  | yviA | hypothetical protein DR75_2662 | 0.53 | 0.40 | 0.01 | NC | NC | NC | 1 | S | - |
| AIL04102.1 |  | rpmJ | ribosomal protein L36 | 0.08 | 0.36 | 0.04 | NC | NC | NC | 1 | J | ko:K02919 |
| AIL04103.1 |  | ndoA | mRNA interferase EndoA | -1.49 | -1.56 | -1.10 | D | D | D | 3 | L | ko:K07171 |
| AIL04105.1 |  | yrrK | hypothetical protein DR75_268 | 0.76 | 0.55 | 0.21 | NC | NC | NC | 1 | L | ko:K07447 |
| AIL04106.1 |  | murB | UDP-N-acetylenolpyruvoylglucosamine reductase | -0.96 | -0.39 | -0.07 | NC | NC | NC | 1 | M | ko:K00075 |
| AIL04110.1 |  | - | glycosyl transferase 2 family protein | 0.44 | -0.17 | -0.18 | NC | NC | NC | 1 | M | ko:K20534 |
| AIL04111.1 |  | - | ngoPII restriction endonuclease family protein | 0.00 | -0.03 | -0.52 | NC | NC | NC | 1 | L | - |
| AIL04113.1 |  | fhuD9 | periplasmic binding family protein | 1.15 | 0.27 | 0.04 | U | NC | NC | 11 | P | ko:K02016 |
| AIL04114.1 |  | rplS | ribosomal protein L19 | -0.24 | 0.01 | -0.31 | NC | NC | NC | 1 | J | ko:K02884 |
| AIL04116.1 |  | - | PTS system fructose IIA component family protein | -1.39 | 1.39 | 0.49 | D | U | NC | 13 | G | ko:K02793 |
| AIL04117.1 |  | WQ51_04310 | asp23 family protein | 0.21 | 0.17 | 0.07 | NC | NC | NC | 1 | S | ko:K10947 |
| AIL04118.1 |  | thiN | thiamine pyrophosphokinase | -0.05 | 1.07 | 1.01 | NC | U | U | 4 | H | ko:K00949 |
| AIL04119.1 |  | cmk | cytidylate kinase | 0.47 | 0.08 | 0.05 | NC | NC | NC | 1 | F | ko:K00945 |
| AIL04120.1 |  | oppC | binding--dependent transport system inner membrane component family protein | -0.42 | -0.13 | -0.24 | NC | NC | NC | 1 | EP | ko:K15582 |
| AIL04121.1 |  | ypsA | hypothetical protein DR75_221 | 0.16 | -1.06 | -1.42 | NC | D | D | 22 | S | - |
| AIL04122.1 |  | yfnA | amino acid permease family protein | -0.06 | -1.08 | -0.42 | NC | D | NC | 23 | E | ko:K03294 |
| AIL04129.1 |  | msmR | bacterial regulatory s, lacI family protein | 0.29 | 0.83 | 1.60 | NC | NC | U | 5 | K | ko:K02529 |
| AIL04130.1 |  | - | PTS system, beta-glucoside-specific IIABC component family protein | 2.69 | 0.33 | 0.51 | U | NC | NC | 11 | G | ko:K02755 |
| AIL04132.1 |  | hepT | polyprenyl synthetase family protein | 0.23 | -0.20 | -0.08 | NC | NC | NC | 1 | H | ko:K00805 |
| AIL04134.1 |  | eno | phosphopyruvate hydratase | -0.50 | -0.34 | -0.53 | NC | NC | NC | 1 | G | ko:K01689 |
| AIL04135.1 |  | - | hypothetical protein DR75_2133 | 0.08 | 0.37 | 0.46 | NC | NC | NC | 1 | - | - |
| AIL04138.1 |  | stp | serine/threonine phosphatase stp | 0.36 | 0.33 | 0.61 | NC | NC | NC | 1 | T | ko:K20074 |
| AIL04139.1 |  | ruvA | Holliday junction DNA helicase RuvA | -0.02 | -0.12 | -0.17 | NC | NC | NC | 1 | L | ko:K03550 |
| AIL04140.1 |  | rpmG | ribosomal protein L33 | -0.68 | -0.15 | -0.49 | NC | NC | NC | 1 | J | ko:K02913 |
| AIL04142.1 |  | yvdB | sulfate permease family protein | -1.07 | -0.98 | -0.95 | D | NC | NC | 6 | U | ko:K03321 |
| AIL04144.1 |  | - | putative m repeat protein | 0.66 | 0.21 | -1.79 | NC | NC | D | 19 | D | - |
| AIL04146.1 |  | yebC | DNA-binding regulatory, YebC/PmpR family protein | -0.03 | 0.16 | 0.07 | NC | NC | NC | 1 | K | - |
| AIL04147.1 |  | mutY | A/G-specific adenine glycosylase | -0.09 | -0.22 | 0.29 | NC | NC | NC | 1 | L | ko:K03575 |
| AIL04148.1 |  | - | response regulator | 0.92 | -0.99 | -0.82 | NC | NC | NC | 1 | K | ko:K18349 |
| AIL04151.1 |  | - | quinone oxidoreductase, YhdH/YhfP family protein | 0.09 | 0.54 | 0.47 | NC | NC | NC | 1 | C | ko:K00001 |
| AIL04154.1 |  | yutD | hypothetical protein DR75_252 | 0.32 | -0.31 | 0.31 | NC | NC | NC | 1 | S | - |
| AIL04155.1 |  | rbfA | ribosome-binding factor A | 0.36 | 0.03 | -0.25 | NC | NC | NC | 1 | J | ko:K02834 |
| AIL04156.1 |  | - | phage tail family protein | 3.85 | -13.29 | 0.73 | U | D | NC | 12 | S | - |
| AIL04157.1 |  | ptsG | PTS system, glucose subfamily, IIA component domain protein | 1.55 | 2.11 | 1.83 | U | U | U | 2 | G | ko:K02777 |
| AIL04158.1 |  | psaA | manganese ABC transporter substrate-binding lipoprotein | -0.27 | -0.44 | -0.38 | NC | NC | NC | 1 | P | ko:K11704 |
| AIL04159.1 |  | - | hypothetical protein DR75_2118 | 0.86 | 0.00 | 0.21 | NC | NC | NC | 1 | - | - |
| AIL04160.1 |  | pyrP | uracil-xanthine permease family protein | -0.46 | -0.69 | -0.39 | NC | NC | NC | 1 | F | ko:K02824 |
| AIL04161.1 |  | tkt | transketolase | -0.38 | 0.14 | 0.01 | NC | NC | NC | 1 | G | ko:K00615 |
| AIL04162.1 |  | dapB | dihydrodipicolinate reductase | -0.66 | -0.16 | -0.12 | NC | NC | NC | 1 | E | ko:K00215 |
| AIL04163.1 |  | rpsM | 30S ribosomal protein S13 | -0.30 | -0.20 | -0.78 | NC | NC | NC | 1 | J | ko:K02952 |
| AIL04166.1 |  | menF | isochorismate synthase family protein | -0.18 | -0.12 | -0.16 | NC | NC | NC | 1 | HQ | ko:K02552 |
| AIL04168.1 |  | fruA | PTS system, fructose subfamily, IIA component domain protein | 0.23 | 0.30 | 0.03 | NC | NC | NC | 1 | GT | ko:K02768 |
| AIL04169.1 |  | - | hypothetical protein DR75_1511 | 0.25 | -0.47 | -0.29 | NC | NC | NC | 1 | S | - |
| AIL04170.1 |  | rplA | ribosomal protein L1 | -0.23 | -0.03 | -0.13 | NC | NC | NC | 1 | J | ko:K02863 |
| AIL04174.1 |  | - | hypothetical protein DR75_2824 | -0.16 | -1.30 | -0.88 | NC | D | NC | 23 | S | - |
| AIL04175.1 |  | dnaG | DNA primase | -0.41 | -0.32 | -0.06 | NC | NC | NC | 1 | K | ko:K02316 |
| AIL04177.1 |  | xylA | xylose isomerase | 0.39 | -1.37 | -1.33 | NC | D | D | 22 | G | ko:K01805 |
| AIL04178.1 |  | ypmB | hypothetical protein DR75_1103 | -0.33 | -0.31 | -0.16 | NC | NC | NC | 1 | S | - |
| AIL04181.1 |  | - | endonuclease/Exonuclease/phosphatase family protein | -1.62 | 1.87 | -1.75 | D | U | D | 17 | L | ko:K06896 |
| AIL04182.1 |  | glpQ | glycerophosphoryl diester phosphodiesterase family protein | -1.28 | -0.54 | 0.33 | D | NC | NC | 6 | C | ko:K01126 |
| AIL04184.1 |  | perR | peroxide operon regulator | -0.01 | -0.52 | -0.88 | NC | NC | NC | 1 | P | ko:K09825 |
| AIL04185.1 |  | - | hypothetical protein DR75_1501 | -0.11 | -0.36 | -0.21 | NC | NC | NC | 1 | M | - |
| AIL04186.1 |  | - | NUDIX domain protein | -2.98 | -1.66 | -0.48 | D | D | NC | 20 | F | ko:K03574 |
| AIL04189.1 |  | gap | glyceraldehyde-3-phosphate dehydrogenase, type I | -0.43 | 0.11 | -0.05 | NC | NC | NC | 1 | G | ko:K00134 |
| AIL04190.1 |  | rbsR | helix-turn-helix family protein | 0.01 | 0.55 | -0.02 | NC | NC | NC | 1 | K | ko:K02529 |
| AIL04191.1 |  | yunF | hypothetical protein DR75_2358 | 0.34 | 0.06 | 0.34 | NC | NC | NC | 1 | S | - |
| AIL04194.1 |  | pyrK | oxidoreductase NAD-binding domain protein | 1.24 | 0.59 | -0.01 | U | NC | NC | 11 | C | ko:K02823 |
| AIL04195.1 |  | oppD | hypothetical protein DR75_2847 | -0.48 | -0.19 | -0.20 | NC | NC | NC | 1 | P | ko:K02031 |
| AIL04196.1 |  | metG | methionine--tRNA ligase | -0.21 | -0.01 | 0.02 | NC | NC | NC | 1 | J | ko:K01874 |
| AIL04197.1 |  | XK27_04845 | transcriptional regulator, MarR family | -1.78 | -0.09 | 0.31 | D | NC | NC | 6 | K | - |
| AIL04199.1 |  | fabD | malonyl CoA-acyl carrier protein transacylase | -0.51 | 0.10 | 0.09 | NC | NC | NC | 1 | I | ko:K00645 |
| AIL04201.1 |  | kup | potassium uptake protein | -0.62 | 0.10 | -0.13 | NC | NC | NC | 1 | P | ko:K03549 |
| AIL04202.1 |  | - | putative membrane protein | -0.09 | -1.06 | -0.23 | NC | D | NC | 23 | - | - |
| AIL04203.1 |  | XK27_08840 | branched-chain amino acid transport system / permease component family protein | 0.38 | 0.48 | -0.09 | NC | NC | NC | 1 | U | ko:K05832 |
| AIL04204.1 |  | glnA | glutamine synthetase, type I | -0.58 | -0.06 | -0.09 | NC | NC | NC | 1 | E | ko:K01915 |
| AIL04205.1 |  | yjbM | hypothetical protein DR75_1370 | -0.29 | 0.49 | 0.27 | NC | NC | NC | 1 | S | ko:K07816 |
| AIL04206.1 |  | rpsU | ribosomal protein S21 | 0.31 | 0.19 | -0.19 | NC | NC | NC | 1 | J | ko:K02970 |
| AIL04207.1 |  | rplC | 50S ribosomal protein L3 | -0.11 | 0.04 | -0.38 | NC | NC | NC | 1 | J | ko:K02906 |
| AIL04208.1 |  | drrA | hypothetical protein DR75_1923 | 0.34 | 0.30 | 0.07 | NC | NC | NC | 1 | V | ko:K01990 |
| AIL04210.1 |  | tuf | translation elongation factor Tu | -0.09 | -0.19 | -0.38 | NC | NC | NC | 1 | J | ko:K02358 |
| AIL04212.1 |  | ydaM | glycosyl transferase 2 family protein | -0.25 | 0.24 | 0.05 | NC | NC | NC | 1 | M | - |
| AIL04214.1 |  | - | UTRA domain protein | -0.43 | -0.08 | -0.25 | NC | NC | NC | 1 | K | ko:K03710 |
| AIL04215.1 |  | allD | ureidoglycolate dehydrogenase | 0.68 | 3.62 | 0.17 | NC | U | NC | 15 | C | ko:K00073 |
| AIL04216.1 |  | pncA | isochorismatase family protein | 0.92 | 0.31 | 0.32 | NC | NC | NC | 1 | Q | ko:K16788 |
| AIL04218.1 |  | accA | acetyl-CoA carboxylase, carboxyl transferase, alpha subunit | -0.49 | -0.10 | -0.07 | NC | NC | NC | 1 | I | ko:K01962 |
| AIL04219.1 |  | dnaB | replication initiation and membrane attachment family protein | -0.16 | 0.53 | 0.75 | NC | NC | NC | 1 | L | ko:K03346 |
| AIL04220.1 |  | ytzB | hypothetical protein DR75_1765 | 0.53 | 0.23 | 0.10 | NC | NC | NC | 1 | S | - |
| AIL04221.1 |  | kduI | 4-deoxy-L-threo-5-hexosulose-uronate ketol-isomerase 1 | -0.71 | -0.09 | 0.27 | NC | NC | NC | 1 | G | ko:K01815 |
| AIL04222.1 |  | - | lysM domain protein | -0.45 | -0.69 | -1.01 | NC | NC | D | 19 | M | - |
| AIL04223.1 |  | pbp1B | transglycosylase family protein | -0.34 | -0.09 | -0.30 | NC | NC | NC | 1 | M | ko:K03693 |
| AIL04226.1 |  | - | hypothetical protein DR75_2349 | -13.29 | 1.66 | 1.15 | D | U | U | 21 | K | - |
| AIL04228.1 |  | hrcA | heat-inducible transcription repressor HrcA | 0.52 | 0.44 | 0.01 | NC | NC | NC | 1 | K | ko:K03705 |
| AIL04229.1 |  | uvrA | excinuclease ABC subunit A | 0.66 | -0.11 | 0.04 | NC | NC | NC | 1 | L | ko:K03701 |
| AIL04230.1 |  | tpx | putative thiol peroxidase | 0.86 | 0.65 | 0.54 | NC | NC | NC | 1 | O | ko:K11065 |
| AIL04232.1 |  | luxS | S-Ribosylhomocysteinase family protein | 0.31 | -0.02 | 0.02 | NC | NC | NC | 1 | H | ko:K07173 |
| AIL04235.1 |  | guaC | guanosine monophosphate reductase | -0.09 | -0.43 | -0.37 | NC | NC | NC | 1 | F | ko:K00364 |
| AIL04239.1 |  | - | hypothetical protein DR75_2373 | -0.23 | -0.24 | -0.30 | NC | NC | NC | 1 | - | - |
| AIL04242.1 |  | grpE | grpE family protein | -0.21 | -0.33 | -0.57 | NC | NC | NC | 1 | O | ko:K03687 |
| AIL04243.1 |  | veg | hypothetical protein DR75_2099 | -0.70 | -1.34 | -1.84 | NC | D | D | 22 | S | - |
| AIL04244.1 |  | czrA | HTH-type transcriptional repressor CzrA | 1.65 | 0.37 | -0.10 | U | NC | NC | 11 | K | ko:K22043 |
| AIL04245.1 |  | - | PTS system, Lactose/Cellobiose specific IIA subunit | 0.25 | 0.13 | -0.15 | NC | NC | NC | 1 | G | ko:K02759 |
| AIL04247.1 |  | - | hypothetical protein DR75_1187 | 1.65 | 0.89 | 0.58 | U | NC | NC | 11 | P | ko:K07220 |
| AIL04249.1 |  | nrdF | ribonucleotide reductase, small chain family protein | 0.86 | 0.09 | 0.01 | NC | NC | NC | 1 | F | ko:K00526 |
| AIL04251.1 |  | ywkD | hypothetical protein DR75_1061 | 0.61 | 0.66 | 0.47 | NC | NC | NC | 1 | E | ko:K08234 |
| AIL04254.1 |  | dnaJ | chaperone protein DnaJ | -0.22 | -0.22 | -0.16 | NC | NC | NC | 1 | O | ko:K03686 |
| AIL04255.1 |  | opuCC | substrate binding domain of ABC-type glycine betaine transport system family protein | -0.69 | -0.15 | -0.08 | NC | NC | NC | 1 | M | ko:K05845 |
| AIL04256.1 |  | rsmG | 16S rRNA (guanine(527)-N(7))-methyltransferase GidB | -0.02 | -0.26 | 0.12 | NC | NC | NC | 1 | J | ko:K03501 |
| AIL04258.1 |  | murB | UDP-N-acetylenolpyruvoylglucosamine reductase | -0.58 | -0.06 | -0.16 | NC | NC | NC | 1 | M | ko:K00075 |
| AIL04259.1 |  | ylxR | hypothetical protein DR75_334 | 0.04 | 0.15 | 0.07 | NC | NC | NC | 1 | K | ko:K07742 |
| AIL04261.1 |  | opuCA | glycine betaine/L-proline transport ATP binding subunit | -0.56 | 0.20 | 0.17 | NC | NC | NC | 1 | E | ko:K05847 |
| AIL04262.1 |  | phoP | alkaline phosphatase synthesis transcriptional regulatory protein phoP | 0.13 | -0.26 | -0.11 | NC | NC | NC | 1 | K | ko:K07658 |
| AIL04264.1 |  | dhrS4 | short chain dehydrogenase family protein | 0.69 | 0.32 | 0.13 | NC | NC | NC | 1 | IQ | ko:K00059 |
| AIL04266.1 |  | - | lipase family protein | -0.24 | -0.18 | -0.08 | NC | NC | NC | 1 | I | - |
| AIL04267.1 |  | - | hypothetical protein DR75_47 | 0.62 | 0.65 | 0.25 | NC | NC | NC | 1 | S | - |
| AIL04269.1 |  | fadD | AMP-binding enzyme family protein | -0.57 | -0.53 | -0.49 | NC | NC | NC | 1 | IQ | ko:K01895 |
| AIL04271.1 |  | guaD | guanine deaminase | -0.01 | -0.23 | -0.34 | NC | NC | NC | 1 | F | ko:K01487 |
| AIL04275.1 |  | ssb | single-stranded DNA-binding family protein | -1.85 | -1.94 | 0.16 | D | D | NC | 20 | L | ko:K03111 |
| AIL04276.1 |  | malL | alpha amylase, catalytic domain protein | -0.75 | -0.25 | -0.48 | NC | NC | NC | 1 | G | ko:K01182 |
| AIL04278.1 |  | gatC | aspartyl/glutamyl-tRNA(Asn/Gln) amidotransferase, C subunit | -0.08 | 0.34 | 0.01 | NC | NC | NC | 1 | J | ko:K02435 |
| AIL04279.1 |  | CP_0775 | hypothetical protein DR75_170 | 2.64 | 1.16 | 0.78 | U | U | NC | 9 | S | ko:K09779 |
| AIL04280.1 |  | bfmBB | 2-oxoacid dehydrogenases acyltransferase family protein | -0.55 | 1.12 | 1.20 | NC | U | U | 4 | C | ko:K00627 |
| AIL04282.1 |  | murE | UDP-N-acetylmuramoyl-L-alanyl-D-glutamate--L-lysine ligase | 0.14 | 0.19 | 0.97 | NC | NC | NC | 1 | M | ko:K01928 |
| AIL04285.1 |  | argS | arginine--tRNA ligase | -0.40 | 0.07 | -0.13 | NC | NC | NC | 1 | J | ko:K01887 |
| AIL04286.1 |  | hpaIIM | modification methylase BspRI | -0.28 | -0.31 | -0.35 | NC | NC | NC | 1 | L | ko:K00558 |
| AIL04288.1 |  | rpsA | hypothetical protein DR75_552 | -0.10 | 0.13 | -0.05 | NC | NC | NC | 1 | J | ko:K02945 |
| AIL04289.1 |  | - | hypothetical protein DR75_1724 | 0.06 | -0.24 | 0.17 | NC | NC | NC | 1 | QT | ko:K09684 |
| AIL04292.1 |  | sgcB | PTS system, Lactose/Cellobiose specific IIB subunit | 1.11 | 2.14 | 1.14 | U | U | U | 2 | G | ko:K02774 |
| AIL04293.1 |  | purR1 | bacterial regulatory s, lacI family protein | -0.46 | 0.39 | -0.06 | NC | NC | NC | 1 | K | ko:K02529 |
| AIL04294.1 |  | ytoA | bacterial transferase hexapeptide family protein | 0.46 | -0.25 | -0.06 | NC | NC | NC | 1 | S | - |
| AIL04295.1 |  | - | glutamate/Leucine/Phenylalanine/Valine dehydrogenase family protein | -0.65 | 2.51 | 1.91 | NC | U | U | 4 | C | ko:K00027 |
| AIL04296.1 |  | nylA | amidase family protein | 0.43 | 0.23 | 0.31 | NC | NC | NC | 1 | J | ko:K01426 |
| AIL04297.1 |  | ffh | signal recognition particle protein | -0.88 | -0.48 | -0.39 | NC | NC | NC | 1 | U | ko:K03106 |
| AIL04298.1 |  | yfiB1 | ABC transporter family protein | -0.57 | 0.06 | -0.27 | NC | NC | NC | 1 | V | ko:K06147 |
| AIL04300.1 |  | rplE | 50S ribosomal protein L5 | -0.21 | -0.22 | -0.28 | NC | NC | NC | 1 | J | ko:K02931 |
| AIL04301.1 |  | lysA | diaminopimelate decarboxylase | -0.62 | -0.25 | -0.28 | NC | NC | NC | 1 | E | ko:K01586 |
| AIL04302.1 |  | tilS | tRNA(Ile)-lysidine synthetase | -0.50 | 0.59 | -0.70 | NC | NC | NC | 1 | J | ko:K04075 |
| AIL04303.1 |  | - | hypothetical protein DR75_1667 | 4.16 | 1.41 | 1.76 | U | U | U | 2 | - | - |
| AIL04304.1 |  | recN | DNA repair protein RecN | -0.31 | 0.09 | 0.04 | NC | NC | NC | 1 | L | ko:K03631 |
| AIL04305.1 |  | - | hypothetical protein DR75_480 | 0.13 | 0.78 | 0.46 | NC | NC | NC | 1 | - | - |
| AIL04309.1 |  | rplX | ribosomal protein L24 | 0.06 | 0.20 | -0.16 | NC | NC | NC | 1 | J | ko:K02895 |
| AIL04310.1 |  | vex3 | ftsX-like permease family protein | -0.36 | -0.08 | 0.57 | NC | NC | NC | 1 | V | ko:K02004 |
| AIL04311.1 |  | XK27_07850 | CBS domain protein | 0.41 | 0.99 | -0.74 | NC | NC | NC | 1 | S | - |
| AIL04312.1 |  | WQ51_03320 | hypothetical protein DR75_2696 | 0.59 | 0.38 | 0.26 | NC | NC | NC | 1 | S | - |
| AIL04313.1 |  | acpP | phosphopantetheine attachment site family protein | -0.85 | -0.14 | -0.81 | NC | NC | NC | 1 | IQ | ko:K02078 |
| AIL04315.1 |  | thyA | thymidylate synthase | -0.62 | -0.11 | -0.37 | NC | NC | NC | 1 | F | ko:K00560 |
| AIL04316.1 |  | - | death-on-curing family protein | -1.21 | -0.53 | -2.36 | D | NC | D | 8 | S | ko:K07341 |
| AIL04320.1 |  | ksgA | dimethyladenosine transferase | -0.24 | -0.06 | -0.27 | NC | NC | NC | 1 | J | ko:K02528 |
| AIL04321.1 |  | - | endonuclease/exonuclease/phosphatase family protein | -0.02 | -0.02 | -0.67 | NC | NC | NC | 1 | S | - |
| AIL04322.1 |  | - | acetyltransferase family protein | 0.06 | 0.00 | -0.02 | NC | NC | NC | 1 | S | ko:K06975 |
| AIL04323.1 |  | - | CBS domain protein | 0.37 | -0.01 | -0.15 | NC | NC | NC | 1 | S | - |
| AIL04324.1 |  | deoC | deoxyribose-phosphate aldolase | 0.36 | 0.36 | 0.19 | NC | NC | NC | 1 | F | ko:K01619 |
| AIL04325.1 |  | - | amidohydrolase family protein | 0.98 | 0.57 | 1.97 | NC | NC | U | 5 | E | - |
| AIL04327.1 |  | tcsA | basic membrane family protein | 0.02 | 0.04 | -0.13 | NC | NC | NC | 1 | S | ko:K02058 |
| AIL04328.1 |  | - | hypothetical protein DR75_314 | -0.57 | -0.43 | -0.45 | NC | NC | NC | 1 | - | - |
| AIL04329.1 |  | greA | transcription elongation factor greA | -0.42 | 0.15 | -0.03 | NC | NC | NC | 1 | K | ko:K03624 |
| AIL04330.1 |  | - | helix-turn-helix family protein | 0.13 | -0.51 | -0.44 | NC | NC | NC | 1 | K | ko:K01356 |
| AIL04331.1 |  | ntpA | V-type sodium ATPase catalytic subunit A | -0.20 | -0.14 | -0.14 | NC | NC | NC | 1 | F | ko:K02117 |
| AIL04332.1 |  | yitW | hypothetical protein DR75_513 | -0.83 | -0.58 | -0.52 | NC | NC | NC | 1 | S | - |
| AIL04333.1 |  | - | bacteriophage HK97-gp10, tail-component family protein | 0.27 | -0.53 | -0.25 | NC | NC | NC | 1 | S | - |
| AIL04335.1 |  | galK | galactokinase | 0.40 | 1.33 | 1.23 | NC | U | U | 4 | G | ko:K00849 |
| AIL04338.1 |  | - | double zinc ribbon family protein | -0.12 | 0.41 | 0.40 | NC | NC | NC | 1 | S | - |
| AIL04341.1 |  | fusA | translation elongation factor G | -0.25 | -0.17 | -0.26 | NC | NC | NC | 1 | J | ko:K02355 |
| AIL04342.1 |  | - | yycH family protein | 0.01 | -0.20 | -0.42 | NC | NC | NC | 1 | S | - |
| AIL04344.1 |  | ndk | nucleoside diphosphate kinase B | 0.73 | 0.34 | 0.33 | NC | NC | NC | 1 | F | ko:K00940 |
| AIL04346.1 |  | - | hypothetical protein DR75_66 | 0.90 | 0.38 | 0.55 | NC | NC | NC | 1 | S | - |
| AIL04347.1 |  | hsdR | DEAD/DEAH box helicase family protein | -0.38 | 0.14 | 0.04 | NC | NC | NC | 1 | V | ko:K01153 |
| AIL04349.1 |  | - | hypothetical protein DR75_1625 | 0.79 | 0.04 | 0.36 | NC | NC | NC | 1 | - | - |
| AIL04350.1 |  | rpsK | 30S ribosomal protein S11 | -0.29 | -0.21 | -0.37 | NC | NC | NC | 1 | J | ko:K02948 |
| AIL04352.1 |  | citF | citrate lyase, alpha subunit | 0.72 | 0.49 | 0.12 | NC | NC | NC | 1 | H | ko:K01643 |
| AIL04354.1 |  | rpsN | 30S ribosomal protein S14 type Z | -0.48 | -0.39 | -0.18 | NC | NC | NC | 1 | J | ko:K02954 |
| AIL04355.1 |  | tyrS | tyrosine--tRNA ligase | 0.23 | -0.29 | -0.11 | NC | NC | NC | 1 | J | ko:K01866 |
| AIL04356.1 |  | - | NUDIX domain protein | 0.07 | 0.21 | 0.12 | NC | NC | NC | 1 | L | ko:K03574 |
| AIL04357.1 |  | sipC | signal peptidase I | 0.37 | -0.38 | -0.08 | NC | NC | NC | 1 | U | ko:K03100 |
| AIL04358.1 |  | cfa | methyltransferase domain protein | 0.53 | 0.23 | 0.27 | NC | NC | NC | 1 | M | ko:K00574 |
| AIL04360.1 |  | sacT | CAT RNA binding domain protein | -0.68 | 0.04 | 0.10 | NC | NC | NC | 1 | K | ko:K03488 |
| AIL04362.1 |  | ywbD | S-adenosylmethionine-dependent methyltransferase family protein | 0.23 | -0.39 | -0.57 | NC | NC | NC | 1 | J | ko:K06969 |
| AIL04365.1 |  | hit | HIT domain protein | 0.29 | 0.60 | 0.43 | NC | NC | NC | 1 | FG | ko:K02503 |
| AIL04366.1 |  | - | ABC transporter family protein | 0.24 | -1.20 | -1.32 | NC | D | D | 22 | E | ko:K10041 |
| AIL04368.1 |  | glpD | alpha-glycerophosphate oxidase | 0.48 | 1.02 | 0.60 | NC | U | NC | 15 | C | ko:K00105 |
| AIL04369.1 |  | XK27_04830 | hypothetical protein DR75_2750 | 0.58 | 0.55 | 0.25 | NC | NC | NC | 1 | S | - |
| AIL04370.1 |  | - | hypothetical protein DR75_1627 | 0.64 | -0.31 | -0.29 | NC | NC | NC | 1 | - | - |
| AIL04371.1 |  | - | hypothetical protein DR75_70 | -1.32 | 0.26 | 0.24 | D | NC | NC | 6 | K | - |
| AIL04372.1 |  | galM | galactose mutarotase | 0.78 | 1.34 | 1.28 | NC | U | U | 4 | G | ko:K01785 |
| AIL04374.1 |  | rpmG | ribosomal protein L33 | -0.33 | -0.06 | 0.16 | NC | NC | NC | 1 | J | ko:K02913 |
| AIL04375.1 |  | ecfA2 | ABC transporter family protein | -0.17 | 0.00 | 0.12 | NC | NC | NC | 1 | P | ko:K16787 |
| AIL04377.1 |  | - | hypothetical protein DR75_1010 | -1.70 | -1.60 | -0.96 | D | D | NC | 20 | M | - |
| AIL04379.1 |  | malE | bacterial extracellular solute-binding family protein | 0.32 | 0.65 | 0.58 | NC | NC | NC | 1 | G | ko:K02027 |
| AIL04380.1 |  | fruC | PTS system, Fru family, IIB component domain protein | -1.20 | -0.08 | -0.67 | D | NC | NC | 6 | G | ko:K02769 |
| AIL04384.1 |  | - | putative prophage Lp2 protein 7 | 0.23 | 0.15 | 0.02 | NC | NC | NC | 1 | - | - |
| AIL04385.1 |  | yaaA | S4 domain protein | 0.59 | 0.32 | 0.15 | NC | NC | NC | 1 | S | ko:K14761 |
| AIL04386.1 |  | clpB | ATP-dependent chaperone protein ClpB | 0.50 | 0.12 | 0.15 | NC | NC | NC | 1 | O | ko:K03695 |
| AIL04389.1 |  | - | type I restriction-modification system methyltransferase subunit | -0.10 | -1.35 | 0.64 | NC | D | NC | 23 | V | ko:K03427 |
| AIL04393.1 |  | rfbC | dTDP-4-dehydrorhamnose 3,5-epimerase | -0.19 | -0.12 | -0.16 | NC | NC | NC | 1 | G | ko:K01790 |
| AIL04394.1 |  | yneA | lysM domain protein | -0.58 | -1.25 | 0.41 | NC | D | NC | 23 | M | ko:K06194 |
| AIL04395.1 |  | - | hypothetical protein DR75_1663 | 1.36 | 0.28 | 0.81 | U | NC | NC | 11 | - | - |
| AIL04397.1 |  | dapA | dihydrodipicolinate synthase | 0.02 | 0.08 | 0.19 | NC | NC | NC | 1 | E | ko:K01714 |
| AIL04398.1 |  | fruR | hypothetical protein DR75_2676 | -0.08 | 0.26 | 0.52 | NC | NC | NC | 1 | K | ko:K03436 |
| AIL04399.1 |  | - | hypothetical protein DR75_1513 | 0.60 | 0.15 | -0.03 | NC | NC | NC | 1 | - | - |
| AIL04400.1 |  | - | phenazine biosynthesis, PhzF family protein | -0.40 | 0.53 | -0.72 | NC | NC | NC | 1 | S | - |
| AIL04401.1 |  | - | alpha/beta hydrolase family protein | 0.99 | 0.07 | 0.31 | NC | NC | NC | 1 | S | ko:K06889 |
| AIL04402.1 |  | - | helix-turn-helix family protein | 0.24 | -0.02 | -0.58 | NC | NC | NC | 1 | F | ko:K00852 |
| AIL04405.1 |  | - | hypothetical protein DR75_529 | 0.31 | 0.27 | -0.19 | NC | NC | NC | 1 | D | ko:K20073 |
| AIL04406.1 |  | - | hypothetical protein DR75_1386 | 0.09 | -0.19 | 0.01 | NC | NC | NC | 1 | - | - |
| AIL04408.1 |  | phnA | phnA Zinc-Ribbon family protein | -0.92 | -0.49 | -0.52 | NC | NC | NC | 1 | P | ko:K06193 |
| AIL04409.1 |  | yclK | HAMP domain protein | 3.63 | 3.55 | 3.39 | U | U | U | 2 | T | - |
| AIL04410.1 |  | glnQ | ABC transporter family protein | -0.42 | 0.19 | 0.14 | NC | NC | NC | 1 | E | ko:K02028 |
| AIL04411.1 |  | ywfO | HD domain protein | 0.08 | -0.06 | 0.01 | NC | NC | NC | 1 | S | ko:K06885 |
| AIL04413.1 |  | - | hypothetical protein DR75_912 | 2.34 | 1.62 | -0.95 | U | U | NC | 9 | S | - |
| AIL04414.1 |  | coaE | dephospho-CoA kinase | 0.46 | -0.21 | -0.16 | NC | NC | NC | 1 | F | ko:K00859 |
| AIL04415.1 |  | ntpD | V-type sodium ATPase subunit D | -0.77 | -1.64 | -0.21 | NC | D | NC | 23 | C | ko:K02120 |
| AIL04416.1 |  | lmrA | ABC transporter family protein | -0.18 | 0.14 | 0.02 | NC | NC | NC | 1 | V | ko:K06147 |
| AIL04417.1 |  | gntR | SIS domain protein | 1.21 | -1.64 | -1.79 | U | D | D | 16 | K | - |
| AIL04418.1 |  | oppC | binding--dependent transport system inner membrane component family protein | -0.44 | -0.69 | -0.62 | NC | NC | NC | 1 | EP | ko:K02034 |
| AIL04419.1 |  | nagE | PTS system, N-acetylglucosamine-specific IIBC component | 0.60 | 0.30 | 0.27 | NC | NC | NC | 1 | G | ko:K02802 |
| AIL04421.1 |  | pstC | phosphate ABC transporter, permease protein PstC | -1.13 | -0.30 | -0.40 | D | NC | NC | 6 | P | ko:K02037 |
| AIL04424.1 |  | ponA | penicillin-binding protein 1A | -0.19 | -0.17 | -0.21 | NC | NC | NC | 1 | M | ko:K05366 |
| AIL04425.1 |  | adk | adenylate kinase | -0.31 | -0.10 | -0.14 | NC | NC | NC | 1 | F | ko:K00939 |
| AIL04426.1 |  | pheA | prephenate dehydratase family protein | -0.85 | -0.64 | -0.93 | NC | NC | NC | 1 | E | ko:K04518 |
| AIL04427.1 |  | csrR | hypothetical protein DR75_109 | -0.11 | 0.12 | 0.07 | NC | NC | NC | 1 | K | - |
| AIL04430.1 |  | cps2D | 3-beta hydroxysteroid dehydrogenase/isomerase family protein | -0.79 | -0.21 | -0.38 | NC | NC | NC | 1 | GM | ko:K01784 |
| AIL04431.1 |  | - | putative membrane protein | 0.71 | 0.50 | -0.51 | NC | NC | NC | 1 | - | - |
| AIL04434.1 |  | valS | valine--tRNA ligase | -0.34 | 0.07 | 0.01 | NC | NC | NC | 1 | J | ko:K01873 |
| AIL04435.1 |  | dnaB | replicative DNA helicase | 0.78 | 0.48 | 0.22 | NC | NC | NC | 1 | L | ko:K02314 |
| AIL04436.1 |  | dinG | exonuclease, DNA polymerase III, epsilon subunit family domain protein | -0.28 | 0.65 | -0.14 | NC | NC | NC | 1 | L | ko:K03722 |
| AIL04439.1 |  | nusG | transcription termination/antitermination factor NusG | -0.08 | 0.12 | 0.10 | NC | NC | NC | 1 | K | ko:K02601 |
| AIL04440.1 |  | dtpT | amino acid/peptide transporter family protein | -0.33 | -0.35 | -0.26 | NC | NC | NC | 1 | U | ko:K03305 |
| AIL04442.1 |  | glmS | glutamine-fructose-6-phosphate transaminase | -0.61 | -0.25 | -0.42 | NC | NC | NC | 1 | M | ko:K00820 |
| AIL04445.1 |  | - | HAD hydrolase, IA, variant 1 family protein | -0.31 | -0.59 | -0.24 | NC | NC | NC | 1 | S | - |
| AIL04446.1 |  | - | helix-turn-helix family protein | -3.19 | -3.59 | -0.85 | D | D | NC | 20 | K | - |
| AIL04447.1 |  | ykuP | flavodoxin | -0.60 | -0.36 | -0.15 | NC | NC | NC | 1 | C | ko:K03839 |
| AIL04449.1 |  | priA | primosomal protein N' | 1.24 | 1.17 | 1.11 | U | U | U | 2 | L | ko:K04066 |
| AIL04450.1 |  | ytxG | hypothetical protein DR75_743 | 0.15 | 0.36 | 0.38 | NC | NC | NC | 1 | S | - |
| AIL04452.1 |  | asd | aspartate-semialdehyde dehydrogenase | 0.20 | -0.15 | 0.06 | NC | NC | NC | 1 | E | ko:K00133 |
| AIL04453.1 |  | mapA | hypothetical protein DR75_10 | 1.66 | 1.99 | 1.60 | U | U | U | 2 | G | ko:K00691 |
| AIL04454.1 |  | glnR | merR regulatory family protein | -0.58 | -0.14 | -0.36 | NC | NC | NC | 1 | K | ko:K03713 |
| AIL04455.1 |  | treP | PTS system, trehalose-specific IIBC component | -0.01 | 0.63 | 0.32 | NC | NC | NC | 1 | G | ko:K02817 |
| AIL04457.1 |  | - | alpha/beta hydrolase fold family protein | -0.01 | -0.52 | -0.45 | NC | NC | NC | 1 | I | - |
| AIL04460.1 |  | rpsR | ribosomal protein S18 | 0.17 | 1.24 | 0.60 | NC | U | NC | 15 | J | ko:K02963 |
| AIL04463.1 |  | tuaA | exopolysaccharide biosynthesis polyprenyl glycosylphosphotransferase family protein | -0.37 | 0.00 | -0.15 | NC | NC | NC | 1 | M | - |
| AIL04464.1 |  | lmrB | drug resistance MFS transporter, drug:H+ antiporter-2 family protein | 0.56 | -0.40 | -1.15 | NC | NC | D | 19 | U | - |
| AIL04465.1 |  | rimM | 16S rRNA processing protein RimM | 0.14 | -0.02 | 0.15 | NC | NC | NC | 1 | J | ko:K02860 |
| AIL04466.1 |  | - | PTS system, Lactose/Cellobiose specific IIB subunit | 0.40 | 1.04 | 1.16 | NC | U | U | 4 | G | - |
| AIL04468.1 |  | yneF | hypothetical protein DR75_732 | 0.16 | -0.43 | -0.26 | NC | NC | NC | 1 | S | ko:K09976 |
| AIL04469.1 |  | - | glyoxalase-like domain protein | 0.16 | 0.19 | 0.00 | NC | NC | NC | 1 | S | - |
| AIL04471.1 |  | proC | pyrroline-5-carboxylate reductase | 0.63 | 0.61 | 0.54 | NC | NC | NC | 1 | E | ko:K00286 |
| AIL04472.1 |  | dnaE | DNA polymerase III, alpha subunit | -0.09 | 0.26 | 0.15 | NC | NC | NC | 1 | L | ko:K02337 |
| AIL04473.1 |  | yjcF | acetyltransferase domain protein | 0.13 | -0.02 | 0.21 | NC | NC | NC | 1 | S | - |
| AIL04474.1 |  | - | iron-containing alcohol dehydrogenase family protein | 0.56 | 0.08 | 0.08 | NC | NC | NC | 1 | C | ko:K08317 |
| AIL04476.1 |  | parE | DNA topoisomerase IV, B subunit | -0.65 | -0.08 | -0.11 | NC | NC | NC | 1 | L | ko:K02622 |
| AIL04477.1 |  | arcA | arginine deiminase | 0.57 | 0.36 | 0.11 | NC | NC | NC | 1 | E | ko:K01478 |
| AIL04478.1 |  | divIVA | DivIVA domain protein | 0.40 | -0.08 | -0.01 | NC | NC | NC | 1 | D | ko:K04074 |
| AIL04479.1 |  | psiE | phosphate-starvation-inducible E family protein | -3.85 | -1.77 | -2.85 | D | D | D | 3 | S | ko:K13256 |
| AIL04480.1 |  | - | hypothetical protein DR75_555 | 0.12 | -0.09 | -0.24 | NC | NC | NC | 1 | E | - |
| AIL04481.1 |  | lutC | hypothetical protein DR75_182 | -2.06 | -0.23 | -0.24 | D | NC | NC | 6 | S | ko:K00782 |
| AIL04482.1 |  | yloV | DAK2 domain fusion YloV family protein | -1.15 | -0.18 | -0.20 | D | NC | NC | 6 | S | ko:K07030 |
| AIL04484.1 |  | - | pTS family porter | -0.77 | -0.20 | 0.16 | NC | NC | NC | 1 | - | - |
| AIL04485.1 |  | - | sugar (and other) transporter family protein | 1.31 | 0.63 | -0.19 | U | NC | NC | 11 | G | - |
| AIL04486.1 |  | - | type I restriction modification DNA specificity domain protein | -0.82 | -0.10 | -0.68 | NC | NC | NC | 1 | V | ko:K01154 |
| AIL04487.1 |  | nplT | hypothetical protein DR75_422 | 1.20 | 1.73 | 1.40 | U | U | U | 2 | G | ko:K01208 |
| AIL04489.1 |  | - | ABC transporter family protein | -1.37 | -1.35 | -0.04 | D | D | NC | 20 | V | ko:K02003 |
| AIL04491.1 |  | ligA | DNA ligase, NAD-dependent | 0.33 | 0.04 | 0.01 | NC | NC | NC | 1 | L | ko:K01972 |
| AIL04492.1 |  | ytxK | N-6 DNA Methylase family protein | -0.70 | -0.14 | -0.20 | NC | NC | NC | 1 | L | ko:K00571 |
| AIL04493.1 |  | - | helix-turn-helix family protein | 0.58 | -0.71 | -13.29 | NC | NC | D | 19 | K | - |
| AIL04494.1 |  | atpE | ATP synthase F0, C subunit | -0.38 | -0.05 | -0.08 | NC | NC | NC | 1 | C | ko:K02110 |
| AIL04496.1 |  | ytqA | radical SAM superfamily protein | -0.51 | -0.45 | -0.48 | NC | NC | NC | 1 | S | ko:K07139 |
| AIL04498.1 |  | tex | S1 RNA binding domain protein | -0.14 | -0.08 | -0.06 | NC | NC | NC | 1 | K | ko:K06959 |
| AIL04502.1 |  | XK27_10430 | NADH(P)-binding family protein | 0.34 | 0.10 | 0.22 | NC | NC | NC | 1 | S | ko:K07118 |
| AIL04505.1 |  | - | SIS domain protein | 0.35 | 0.21 | 0.24 | NC | NC | NC | 1 | K | - |
| AIL04506.1 |  | ywrF | flavin reductase like domain protein | 0.36 | 0.02 | 0.03 | NC | NC | NC | 1 | S | - |
| AIL04507.1 |  | - | hypothetical protein DR75_563 | -0.05 | 0.15 | -0.14 | NC | NC | NC | 1 | - | - |
| AIL04508.1 |  | - | bacterial cellulose synthase subunit | 0.12 | 0.18 | -0.08 | NC | NC | NC | 1 | S | - |
| AIL04509.1 |  | - | ATP-dependent DNA helicase RecQ | 0.99 | -0.06 | -0.28 | NC | NC | NC | 1 | L | ko:K03654 |
| AIL04511.1 |  | - | bacterial membrane YfhO family protein | -0.10 | 0.24 | 1.07 | NC | NC | U | 5 | S | - |
| AIL04512.1 |  | - | hypothetical protein DR75_2655 | 1.94 | 1.70 | 0.83 | U | U | NC | 9 | - | - |
| AIL04513.1 |  | - | glyoxalase/Bleomycin resistance /Dioxygenase superfamily protein | 0.49 | 1.02 | 0.47 | NC | U | NC | 15 | E | - |
| AIL04515.1 |  | sdaAB | L-serine dehydratase, iron-sulfur-dependent, beta subunit | -0.80 | -0.91 | -0.06 | NC | NC | NC | 1 | E | ko:K01752 |
| AIL04516.1 |  | mutS | DNA mismatch repair protein MutS | 0.06 | -0.11 | 0.01 | NC | NC | NC | 1 | L | ko:K03555 |
| AIL04519.1 |  | - | amino acid permease family protein | -0.19 | -0.80 | -0.25 | NC | NC | NC | 1 | E | ko:K20265 |
| AIL04522.1 |  | ntpE | putative v-type ATPase, subunit E | -0.07 | 0.16 | -0.22 | NC | NC | NC | 1 | C | ko:K02121 |
| AIL04523.1 |  | rlmN | 23S rRNA methyltransferase | -0.77 | -0.26 | -0.08 | NC | NC | NC | 1 | J | ko:K06941 |
| AIL04524.1 |  | folC | bifunctional FolC family protein | 0.35 | 0.01 | 0.03 | NC | NC | NC | 1 | H | ko:K11754 |
| AIL04525.1 |  | - | acetyltransferase family protein | 0.07 | 0.42 | -0.35 | NC | NC | NC | 1 | J | ko:K03790 |
| AIL04526.1 |  | - | hypothetical protein DR75_877 | -0.54 | 0.08 | -0.14 | NC | NC | NC | 1 | - | - |
| AIL04529.1 |  | XK27_08850 | aminoacyl-tRNA editing domain protein | 0.11 | -0.57 | -0.41 | NC | NC | NC | 1 | S | ko:K19055 |
| AIL04530.1 |  | yacP | yacP-like NYN domain protein | 0.12 | -0.54 | -1.56 | NC | NC | D | 19 | S | ko:K06962 |
| AIL04532.1 |  | ymfM | helix-turn-helix domain protein | -0.47 | -0.28 | -0.38 | NC | NC | NC | 1 | S | ko:K15539 |
| AIL04533.1 |  | - | hypothetical protein DR75_899 | 0.06 | 0.07 | 0.16 | NC | NC | NC | 1 | - | - |
| AIL04534.1 |  | rpsD | ribosomal protein S4 | -0.29 | -0.18 | -0.34 | NC | NC | NC | 1 | J | ko:K02986 |
| AIL04536.1 |  | gatB | aspartyl/glutamyl-tRNA(Asn/Gln) amidotransferase, B subunit | -0.07 | 0.07 | -0.07 | NC | NC | NC | 1 | J | ko:K02434 |
| AIL04537.1 |  | tcaA | hypothetical protein DR75_546 | 0.05 | 0.64 | -0.70 | NC | NC | NC | 1 | S | ko:K21463 |
| AIL04539.1 |  | ygaC | hypothetical protein DR75_2648 | 0.43 | 0.34 | 0.27 | NC | NC | NC | 1 | J | ko:K07586 |
| AIL04540.1 |  | - | hypothetical protein DR75_1674 | 0.13 | -0.31 | -0.07 | NC | NC | NC | 1 | - | - |
| AIL04544.1 |  | - | hypothetical protein DR75_1400 | -0.60 | -0.41 | -0.14 | NC | NC | NC | 1 | - | - |
| AIL04546.1 |  | tyrS | tyrosine--tRNA ligase | -0.35 | -0.09 | -0.04 | NC | NC | NC | 1 | J | ko:K01866 |
| AIL04547.1 |  | rpmD | ribosomal protein L30 | -0.05 | 0.09 | 0.49 | NC | NC | NC | 1 | J | ko:K02907 |
| AIL04548.1 |  | mntB | ABC transporter family protein | -0.30 | 0.23 | -0.25 | NC | NC | NC | 1 | P | ko:K02074 |
| AIL04549.1 |  | - | helix-turn-helix family protein | -0.01 | 0.06 | -0.07 | NC | NC | NC | 1 | K | ko:K20342 |
| AIL04551.1 |  | - | TLP18.3, Psb32 and MOLO-1 founding s of phosphatase family protein | -0.31 | -0.63 | -1.20 | NC | NC | D | 19 | S | ko:K06872 |
| AIL04552.1 |  | - | hypothetical protein DR75_2003 | -0.58 | -0.35 | -0.41 | NC | NC | NC | 1 | S | - |
| AIL04553.1 |  | ptsH | phosphocarrier protein HPr | -0.57 | 0.78 | 0.35 | NC | NC | NC | 1 | G | ko:K11189 |
| AIL04554.1 |  | murE2 | mur ligase middle domain protein | -0.01 | 0.03 | 0.16 | NC | NC | NC | 1 | M | ko:K01928 |
| AIL04556.1 |  | ylxQ | ribosomal L7Ae/L30e/S12e/Gadd45 family protein | -0.03 | -0.38 | -0.63 | NC | NC | NC | 1 | J | - |
| AIL04557.1 |  | nrdE | ribonucleoside-diphosphate reductase, alpha subunit | 0.93 | 0.09 | 0.05 | NC | NC | NC | 1 | F | ko:K00525 |
| AIL04558.1 |  | budA | alpha-acetolactate decarboxylase | -0.49 | 0.79 | 0.45 | NC | NC | NC | 1 | H | ko:K01575 |
| AIL04559.1 |  | yxlJ | DNA-3-methyladenine glycosylase family protein | -0.81 | -0.84 | -0.32 | NC | NC | NC | 1 | L | ko:K03652 |
| AIL04560.1 |  | aroK | shikimate kinase family protein | -0.99 | -0.40 | -0.51 | NC | NC | NC | 1 | F | ko:K00891 |
| AIL04561.1 |  | lytS | 5TMR of 5TMR-LYT family protein | 0.12 | 0.41 | 0.13 | NC | NC | NC | 1 | T | ko:K07704 |
| AIL04562.1 |  | pacL | HAD ATPase, P-type, IC family protein | 0.10 | -0.15 | -0.24 | NC | NC | NC | 1 | P | ko:K01537 |
| AIL04565.1 |  | proB | glutamate 5-kinase | -0.30 | -0.37 | -0.63 | NC | NC | NC | 1 | F | ko:K00931 |
| AIL04566.1 |  | rumA_1 | 23S rRNA (uracil-5-)-methyltransferase RumA | 1.29 | -0.20 | -0.62 | U | NC | NC | 11 | J | ko:K00557 |
| AIL04567.1 |  | rex | oxidoreductase, NAD-binding Rossmann fold family protein | -0.50 | -0.75 | -0.90 | NC | NC | NC | 1 | K | ko:K01926 |
| AIL04570.1 |  | rpoZ | DNA-directed RNA polymerase, omega subunit | -0.07 | -0.12 | -0.28 | NC | NC | NC | 1 | K | ko:K03060 |
| AIL04571.1 |  | rpoA | DNA-directed RNA polymerase, alpha subunit | 0.03 | 0.08 | 0.02 | NC | NC | NC | 1 | K | ko:K03040 |
| AIL04572.1 |  | - | galactose mutarotase related enzyme | 0.07 | 0.53 | 0.16 | NC | NC | NC | 1 | G | - |
| AIL04575.1 |  | rex | DNA-binding family protein | 0.39 | 0.32 | 0.14 | NC | NC | NC | 1 | K | ko:K01926 |
| AIL04577.1 |  | arnC | glycosyl transferase 2 family protein | -0.45 | -0.47 | -0.10 | NC | NC | NC | 1 | M | ko:K00786 |
| AIL04578.1 |  | opuCD | binding--dependent transport system inner membrane component family protein | 2.88 | 2.45 | 2.02 | U | U | U | 2 | E | ko:K05846 |
| AIL04581.1 |  | guaA | GMP synthase | -0.01 | -0.04 | -0.12 | NC | NC | NC | 1 | F | ko:K01951 |
| AIL04583.1 |  | - | putative membrane protein | 0.12 | 0.12 | 0.09 | NC | NC | NC | 1 | - | - |
| AIL04584.1 |  | cshA | hypothetical protein DR75_2786 | -0.26 | -0.39 | -0.52 | NC | NC | NC | 1 | F | ko:K05592 |
| AIL04587.1 |  | lacD | tagatose 1,6-diphosphate aldolase | -1.68 | -0.76 | -0.28 | D | NC | NC | 6 | G | ko:K01635 |
| AIL04588.1 |  | - | oxidoreductase, Gfo/Idh/MocA family | 1.27 | 0.83 | 0.59 | U | NC | NC | 11 | S | - |
| AIL04590.1 |  | yueF | hypothetical protein DR75_2650 | 0.43 | -0.24 | -0.17 | NC | NC | NC | 1 | S | - |
| AIL04591.1 |  | - | hypothetical protein DR75_2722 | 1.48 | 0.76 | 0.66 | U | NC | NC | 11 | O | - |
| AIL04602.1 |  | cspC | cold shock protein CspC | 0.30 | 1.31 | 0.77 | NC | U | NC | 15 | K | ko:K03704 |
| AIL04604.1 |  | - | Cof-like hydrolase family protein | -0.52 | -0.06 | -0.39 | NC | NC | NC | 1 | S | - |
| AIL04605.1 |  | rpsL | ribosomal protein S12 | 0.13 | -0.02 | -0.30 | NC | NC | NC | 1 | J | ko:K02950 |
| AIL04608.1 |  | cshB | hypothetical protein DR75_451 | 0.01 | 0.15 | -0.56 | NC | NC | NC | 1 | JKL | ko:K05592 |
| AIL04609.1 |  | - | fibronectin-binding family protein | -0.66 | 0.75 | -0.25 | NC | NC | NC | 1 | S | - |
| AIL04610.1 |  | - | signal peptidase I | 0.18 | -0.17 | 0.03 | NC | NC | NC | 1 | U | ko:K03100 |
| AIL04612.1 |  | ytmP | phosphotransferase enzyme family protein | -1.95 | -0.82 | -0.77 | D | NC | NC | 6 | M | - |
| AIL04614.1 |  | ycjM | hypothetical protein DR75_1597 | -0.35 | 0.21 | 0.00 | NC | NC | NC | 1 | F | ko:K01119 |
| AIL04615.1 |  | - | D-alanyl-D-alanine carboxypeptidase family protein | -1.79 | -0.61 | -0.25 | D | NC | NC | 6 | M | ko:K01286 |
| AIL04616.1 |  | obg | Obg family GTPase CgtA | -0.33 | -0.16 | -0.15 | NC | NC | NC | 1 | S | ko:K03979 |
| AIL04617.1 |  | atpB | ATP synthase F0, A subunit | -0.55 | -0.28 | -0.48 | NC | NC | NC | 1 | C | ko:K02108 |
| AIL04619.1 |  | gldA | iron-containing alcohol dehydrogenase family protein | 0.92 | 2.81 | 2.55 | NC | U | U | 4 | C | ko:K00005 |
| AIL04620.1 |  | lacC | tagatose-6-phosphate kinase | 3.18 | 1.25 | 1.41 | U | U | U | 2 | F | ko:K00917 |
| AIL04621.1 |  | copZ | heavy-metal-associated domain protein | 0.88 | 0.41 | 0.15 | NC | NC | NC | 1 | P | - |
| AIL04622.1 |  | lysR | bacterial regulatory helix-turn-helix, lysR family protein | 0.57 | 0.17 | 0.18 | NC | NC | NC | 1 | K | - |
| AIL04623.1 |  | yktB | hypothetical protein DR75_1800 | -0.61 | -0.17 | -0.22 | NC | NC | NC | 1 | S | - |
| AIL04624.1 |  | - | acetyltransferase family protein | 0.01 | -0.08 | 0.22 | NC | NC | NC | 1 | S | - |
| AIL04628.1 |  | trpS | tryptophan--tRNA ligase | -0.29 | -0.09 | -0.11 | NC | NC | NC | 1 | J | ko:K01867 |
| AIL04629.1 |  | polA | DNA polymerase I | 0.13 | 0.00 | 0.01 | NC | NC | NC | 1 | L | ko:K02335 |
| AIL04631.1 |  | - | hypothetical protein DR75_2587 | 1.02 | 0.56 | 0.69 | U | NC | NC | 11 | S | - |
| AIL04632.1 |  | sbcD | exonuclease SbcCD, D subunit | -0.91 | -0.67 | -0.02 | NC | NC | NC | 1 | L | ko:K03547 |
| AIL04633.1 |  | panE2 | 2-dehydropantoate 2-reductase family protein | 0.60 | 0.33 | 0.20 | NC | NC | NC | 1 | H | ko:K00077 |
| AIL04634.1 |  | gor | glutathione-disulfide reductase | 0.16 | -0.04 | -0.01 | NC | NC | NC | 1 | C | ko:K00383 |
| AIL04641.1 |  | arcR | cyclic nucleotide-binding domain protein | 0.61 | 1.50 | 1.13 | NC | U | U | 4 | K | ko:K21828 |
| AIL04644.1 |  | - | 3-demethylubiquinone-9 3-methyltransferase family protein | 0.30 | -0.32 | 0.33 | NC | NC | NC | 1 | S | ko:K04750 |
| AIL04645.1 |  | aspB3 | beta-eliminating lyase family protein | -1.01 | -0.29 | -0.17 | D | NC | NC | 6 | E | - |
| AIL04646.1 |  | nfo | putative endonuclease 4 | 0.39 | 0.02 | -0.05 | NC | NC | NC | 1 | L | ko:K01151 |
| AIL04647.1 |  | yqfF | hypothetical protein DR75_1160 | -0.02 | 0.38 | 0.01 | NC | NC | NC | 1 | S | ko:K07037 |
| AIL04649.1 |  | sufD | FeS assembly protein SufD | 0.08 | 0.19 | -0.09 | NC | NC | NC | 1 | O | ko:K07033 |
| AIL04651.1 |  | arlS | HAMP domain protein | -0.46 | -0.51 | -0.61 | NC | NC | NC | 1 | T | ko:K18940 |
| AIL04657.1 |  | pdhB | pyruvate dehydrogenase E1 component subunit beta | 0.50 | 0.47 | 0.29 | NC | NC | NC | 1 | C | ko:K00162 |
| AIL04660.1 |  | ecfA1 | ABC transporter family protein | -0.18 | -0.30 | -0.34 | NC | NC | NC | 1 | P | ko:K16786 |
| AIL04662.1 |  | ctpA | hypothetical protein DR75_680 | 0.63 | 0.22 | 0.85 | NC | NC | NC | 1 | M | ko:K03797 |
| AIL04664.1 |  | - | ftsX-like permease family protein | 0.58 | -0.52 | 0.17 | NC | NC | NC | 1 | V | ko:K11636 |
| AIL04666.1 |  | ytqB | rRNA methylase family protein | -1.97 | -2.10 | -0.19 | D | D | NC | 20 | J | - |
| AIL04667.1 |  | - | mazG nucleotide pyrophosphohydrolase domain protein | -0.22 | -0.29 | -0.53 | NC | NC | NC | 1 | S | - |
| AIL04669.1 |  | mtlA | PTS system, Lactose/Cellobiose specific IIB subunit | -0.47 | -3.24 | -13.29 | NC | D | D | 22 | G | ko:K02799 |
| AIL04671.1 |  | smc | chromosome segregation protein SMC | -0.83 | -0.39 | -0.02 | NC | NC | NC | 1 | D | ko:K03529 |
| AIL04672.1 |  | yeaZ | tRNA threonylcarbamoyl adenosine modification protein YeaZ | 0.02 | 0.22 | 0.01 | NC | NC | NC | 1 | O | ko:K01409 |
| AIL04674.1 |  | yhxD | hypothetical protein DR75_2117 | 1.25 | 0.56 | 0.42 | U | NC | NC | 11 | IQ | - |
| AIL04684.1 |  | lytT | response regulator | 0.50 | 0.17 | 0.10 | NC | NC | NC | 1 | K | ko:K07705 |
| AIL04686.1 |  | XK27_05225 | tetratricopeptide repeat family protein | 0.12 | 0.27 | -0.04 | NC | NC | NC | 1 | S | - |
| AIL04687.1 |  | - | bacterial extracellular solute-binding family protein | -0.09 | 0.00 | -0.20 | NC | NC | NC | 1 | G | ko:K02027 |
| AIL04690.1 |  | - | hypothetical protein DR75_2140 | -1.25 | -0.20 | -0.49 | D | NC | NC | 6 | K | ko:K03402 |
| AIL04693.1 |  | - | hypothetical protein DR75_1978 | 0.11 | -0.07 | -0.01 | NC | NC | NC | 1 | KT | - |
| AIL04696.1 |  | ykcA | glyoxalase/Bleomycin resistance /Dioxygenase superfamily protein | -0.07 | 0.52 | 0.28 | NC | NC | NC | 1 | E | - |
| AIL04697.1 |  | rplW | 50S ribosomal protein L23 | -0.13 | -0.35 | -0.38 | NC | NC | NC | 1 | J | ko:K02892 |
| AIL04698.1 |  | - | lipid kinase, YegS//BmrU family protein | 0.40 | 0.19 | 0.15 | NC | NC | NC | 1 | I | - |
| AIL04699.1 |  | asnS | asparagine--tRNA ligase | -0.17 | -0.07 | -0.22 | NC | NC | NC | 1 | J | ko:K01893 |
| AIL04701.1 |  | cydA | bacterial Cytochrome Ubiquinol Oxidase family protein | -0.82 | -0.01 | -0.26 | NC | NC | NC | 1 | C | ko:K00425 |
| AIL04703.1 |  | dps | DNA protection during starvation protein | 0.15 | -0.42 | 0.12 | NC | NC | NC | 1 | P | ko:K04047 |
| AIL04706.1 |  | pepQ | xaa-Pro dipeptidase | 0.25 | 0.25 | 0.06 | NC | NC | NC | 1 | E | ko:K01271 |
| AIL04707.1 |  | yuaF | putative yuaF like protein | 0.26 | 0.71 | 0.59 | NC | NC | NC | 1 | OU | - |
| AIL04710.1 |  | plsC | acyltransferase family protein | 0.14 | -0.06 | -0.06 | NC | NC | NC | 1 | I | ko:K00655 |
| AIL04711.1 |  | - | hypothetical protein DR75_2613 | 0.52 | 0.71 | 0.65 | NC | NC | NC | 1 | - | - |
| AIL04712.1 |  | - | glycosyl transferase 2 family protein | -0.07 | 0.14 | -0.11 | NC | NC | NC | 1 | M | - |
| AIL04715.1 |  | mrnC | ribonuclease III domain protein | 0.05 | -0.27 | -0.47 | NC | NC | NC | 1 | J | ko:K11145 |
| AIL04718.1 |  | oppA1 | bacterial extracellular solute-binding s, 5 Middle family protein | -0.11 | -0.25 | -0.29 | NC | NC | NC | 1 | E | ko:K02035 |
| AIL04719.1 |  | - | PTS system mannose/fructose/sorbose IID component family protein | 0.05 | -0.04 | 0.94 | NC | NC | NC | 1 | G | ko:K19509 |
| AIL04720.1 |  | - | hypothetical protein DR75_1650 | 0.87 | -1.41 | -0.55 | NC | D | NC | 23 | - | - |
| AIL04721.1 |  | glnP | lysine-arginine-ornithine-binding periplasmic family protein | -0.20 | -0.15 | -0.29 | NC | NC | NC | 1 | P | ko:K02029 |
| AIL04723.1 |  | mleS | malic enzyme, NAD binding domain protein | -0.19 | -0.20 | 0.07 | NC | NC | NC | 1 | C | ko:K00027 |
| AIL04724.1 |  | - | cysteine-rich secretory family protein | -0.53 | -0.45 | -0.45 | NC | NC | NC | 1 | S | ko:K21471 |
| AIL04725.1 |  | yumB | FAD dependent oxidoreductase family protein | -0.47 | -0.10 | -0.07 | NC | NC | NC | 1 | C | ko:K03885 |
| AIL04726.1 |  | - | putative membrane protein | 0.26 | -0.75 | 0.64 | NC | NC | NC | 1 | - | - |
| AIL04727.1 |  | yabR | S1 RNA binding domain protein | -0.12 | 0.07 | -0.08 | NC | NC | NC | 1 | J | ko:K07571 |
| AIL04729.1 |  | ypdD | alpha-1,2-mannosidase family protein | 0.05 | 1.13 | 0.64 | NC | U | NC | 15 | G | - |
| AIL04730.1 |  | yhaO | calcineurin-like phosphoesterase family protein | 0.02 | -0.35 | 0.01 | NC | NC | NC | 1 | L | ko:K03547 |
| AIL04736.1 |  | ecsA | ABC transporter family protein | -0.69 | 0.16 | 0.14 | NC | NC | NC | 1 | V | ko:K01990 |
| AIL04737.1 |  | - | hypothetical protein DR75_1099 | 0.89 | 1.41 | 1.28 | NC | U | U | 4 | - | - |
| AIL04740.1 |  | rhaD | rhamnulose-1-phosphate aldolase | 0.23 | 0.57 | 0.33 | NC | NC | NC | 1 | G | ko:K01629 |
| AIL04741.1 |  | rluD | pseudouridine synthase, RluA family protein | -0.22 | -0.34 | -0.28 | NC | NC | NC | 1 | J | ko:K06180 |
| AIL04742.1 |  | mvk | mevalonate kinase | -0.36 | 0.10 | -0.59 | NC | NC | NC | 1 | I | ko:K00869 |
| AIL04743.1 |  | - | hypothetical protein DR75_1484 | 0.06 | 0.28 | 0.47 | NC | NC | NC | 1 | S | - |
| AIL04744.1 |  | cobB | sir2 family protein | 0.65 | 0.99 | 0.48 | NC | NC | NC | 1 | K | ko:K12410 |
| AIL04745.1 |  | yheA | hypothetical protein DR75_2631 | -0.39 | -0.08 | -0.79 | NC | NC | NC | 1 | S | - |
| AIL04746.1 |  | atpF | ATP synthase F0, B subunit | -0.36 | -0.24 | -0.21 | NC | NC | NC | 1 | C | ko:K02109 |
| AIL04747.1 |  | - | hypothetical protein DR75_1089 | 0.35 | -0.17 | -0.31 | NC | NC | NC | 1 | F | - |
| AIL04749.1 |  | tsaD | tRNA threonylcarbamoyl adenosine modification protein YgjD | -0.18 | -0.19 | -0.23 | NC | NC | NC | 1 | O | ko:K01409 |
| AIL04750.1 |  | mleR2 | bacterial regulatory helix-turn-helix, lysR family protein | -0.46 | 1.13 | 0.25 | NC | U | NC | 15 | K | - |
| AIL04752.1 |  | oppF | ABC transporter family protein | -0.77 | -0.43 | -0.43 | NC | NC | NC | 1 | E | ko:K10823 |
| AIL04753.1 |  | aldA | acyl-CoA reductase family protein | 0.87 | 1.17 | 1.01 | NC | U | U | 4 | C | ko:K00128 |
| AIL04754.1 |  | rpoC | DNA-directed RNA polymerase, beta' subunit | -0.06 | 0.02 | -0.06 | NC | NC | NC | 1 | K | ko:K03046 |
| AIL04755.1 |  | rsmI | ribosomal RNA small subunit methyltransferase I | 0.06 | -0.48 | -0.47 | NC | NC | NC | 1 | H | ko:K07056 |
| AIL04756.1 |  | mta | merR regulatory family protein | -0.45 | -0.58 | 0.05 | NC | NC | NC | 1 | K | - |
| AIL04757.1 |  | - | hypothetical protein DR75_1510 | 0.80 | -0.27 | 0.28 | NC | NC | NC | 1 | - | - |
| AIL04761.1 |  | xerD | tyrosine recombinase XerD | 0.45 | 0.07 | -0.04 | NC | NC | NC | 1 | D | ko:K04763 |
| AIL04762.1 |  | lepA | GTP-binding protein LepA | -0.11 | -0.03 | -0.04 | NC | NC | NC | 1 | M | ko:K03596 |
| AIL04764.1 |  | recJ | single-stranded-DNA-specific exonuclease RecJ | -0.18 | -0.38 | 0.15 | NC | NC | NC | 1 | L | ko:K07462 |
| AIL04765.1 |  | iscS | aminotransferase class-V family protein | -0.01 | -0.13 | -0.29 | NC | NC | NC | 1 | E | ko:K04487 |
| AIL04768.1 |  | - | hypothetical protein DR75_1652 | 0.23 | -0.08 | -0.41 | NC | NC | NC | 1 | S | - |
| AIL04770.1 |  | gshF | glutamate--cysteine ligase/gamma-glutamylcysteine synthetase | 0.29 | 0.25 | 0.16 | NC | NC | NC | 1 | F | ko:K01919 |
| AIL04772.1 |  | - | hypothetical protein DR75_1658 | 1.86 | 0.07 | 0.33 | U | NC | NC | 11 | - | - |
| AIL04773.1 |  | aroE | shikimate 5-dehydrogenase | -1.06 | -0.52 | -0.43 | D | NC | NC | 6 | E | ko:K00014 |
| AIL04774.1 |  | pgmB | beta-phosphoglucomutase | -0.31 | -0.08 | -0.09 | NC | NC | NC | 1 | S | ko:K01838 |
| AIL04776.1 |  | ykzG | hypothetical protein DR75_1617 | -0.22 | 0.43 | 0.47 | NC | NC | NC | 1 | S | - |
| AIL04778.1 |  | rimP | hypothetical protein DR75_332 | -0.39 | -0.36 | -0.14 | NC | NC | NC | 1 | S | ko:K09748 |
| AIL04782.1 |  | - | hypothetical protein DR75_6 | -0.30 | 0.28 | -0.22 | NC | NC | NC | 1 | - | - |
| AIL04783.1 |  | oppD | hypothetical protein DR75_1822 | -0.07 | -0.18 | 0.26 | NC | NC | NC | 1 | P | ko:K02031 |
| AIL04784.1 |  | ytpP | thioredoxin family protein | 0.50 | 1.03 | 0.62 | NC | U | NC | 15 | CO | - |
| AIL04785.1 |  | yhaH | ytxH-like family protein | -0.24 | 0.20 | 0.31 | NC | NC | NC | 1 | D | - |
| AIL04786.1 |  | - | hypothetical protein DR75_1451 | -0.33 | -0.01 | 0.23 | NC | NC | NC | 1 | S | ko:K02068 |
| AIL04788.1 |  | - | cyclophilin type peptidyl-prolyl cis-trans isomerase/CLD family protein | 0.51 | -0.08 | -0.18 | NC | NC | NC | 1 | M | ko:K03767 |
| AIL04789.1 |  | dut | dUTPase family protein | -0.01 | 0.21 | 0.19 | NC | NC | NC | 1 | F | ko:K01520 |
| AIL04790.1 |  | rpmG | ribosomal protein L33 | -0.57 | -0.85 | -0.06 | NC | NC | NC | 1 | J | ko:K02913 |
| AIL04792.1 |  | - | hlyD secretion family protein | -0.14 | 0.02 | 0.26 | NC | NC | NC | 1 | M | ko:K02005 |
| AIL04795.1 |  | argR2 | arginine repressor | -0.24 | -0.45 | -0.75 | NC | NC | NC | 1 | K | ko:K03402 |
| AIL04796.1 |  | - | phosphoribulokinase / Uridine kinase family protein | -0.34 | 0.00 | -2.31 | NC | NC | D | 19 | F | - |
| AIL04797.1 |  | ddl | D-alanine--D-alanine ligase family protein | -0.09 | 0.16 | 0.06 | NC | NC | NC | 1 | F | ko:K01921 |
| AIL04798.1 |  | rbsR | periplasmic binding s and sugar binding domain of LacI family protein | -0.18 | -0.09 | -0.35 | NC | NC | NC | 1 | K | ko:K02529 |
| AIL04799.1 |  | scrR | helix-turn-helix family protein | 0.06 | -0.06 | -0.32 | NC | NC | NC | 1 | K | ko:K02529 |
| AIL04802.1 |  | gdh | glucose 1-dehydrogenase | 0.27 | -0.36 | 0.06 | NC | NC | NC | 1 | IQ | ko:K00034 |
| AIL04803.1 |  | purK | D-ala D-ala ligase family protein | -0.21 | -0.13 | -0.14 | NC | NC | NC | 1 | F | ko:K01589 |
| AIL04804.1 |  | - | csbD-like family protein | 0.51 | 1.12 | 0.57 | NC | U | NC | 15 | S | - |
| AIL04806.1 |  | lutB | iron-sulfur cluster-binding protein | -0.46 | -0.19 | -0.22 | NC | NC | NC | 1 | C | ko:K18929 |
| AIL04807.1 |  | sirR | feoA domain protein | 0.25 | -0.03 | -0.14 | NC | NC | NC | 1 | K | ko:K03709 |
| AIL04809.1 |  | dgoD | hypothetical protein DR75_2526 | 1.15 | 1.36 | 0.79 | U | U | NC | 9 | M | ko:K01684 |
| AIL04810.1 |  | XK27_09600 | ABC transporter family protein | -0.78 | 0.29 | -0.89 | NC | NC | NC | 1 | V | ko:K06147 |
| AIL04811.1 |  | - | peptidase, ArgE/DapE family protein | 0.83 | 0.47 | 0.32 | NC | NC | NC | 1 | E | ko:K01439 |
| AIL04812.1 |  | rpsQ | 30S ribosomal protein S17 | -0.47 | -0.46 | -0.63 | NC | NC | NC | 1 | J | ko:K02961 |
| AIL04813.1 |  | - | amidinotransferase family protein | 0.13 | 0.11 | 0.28 | NC | NC | NC | 1 | E | - |
| AIL04814.1 |  | dus | TIM-barrel, nifR3 family protein | -0.50 | -0.07 | -0.10 | NC | NC | NC | 1 | J | ko:K05540 |
| AIL04815.1 |  | yneT | coA binding domain protein | 0.80 | 0.62 | 0.24 | NC | NC | NC | 1 | S | ko:K06929 |
| AIL04816.1 |  | ftsZ | cell division protein FtsZ | -0.22 | -0.03 | -0.10 | NC | NC | NC | 1 | D | ko:K03531 |
| AIL04818.1 |  | nudF | NUDIX domain protein | 0.15 | 0.18 | 0.43 | NC | NC | NC | 1 | L | ko:K01515 |
| AIL04819.1 |  | rfbA5 | ABC-2 type transporter family protein | 0.25 | -0.40 | -2.02 | NC | NC | D | 19 | U | ko:K09692 |
| AIL04820.1 |  | divIB | cell division FtsQ family protein | -0.57 | -0.25 | -0.26 | NC | NC | NC | 1 | D | ko:K03589 |
| AIL04822.1 |  | - | hypothetical protein DR75_301 | -0.88 | 0.08 | -0.51 | NC | NC | NC | 1 | L | - |
| AIL04824.1 |  | yjbK | hypothetical protein DR75_1371 | 0.27 | 0.48 | 2.37 | NC | NC | U | 5 | S | - |
| AIL04825.1 |  | yqeM | methyltransferase domain protein | -0.71 | -0.10 | -0.02 | NC | NC | NC | 1 | Q | - |
| AIL04828.1 |  | - | peptidase propeptide and YPEB domain protein | 0.03 | 0.01 | -0.11 | NC | NC | NC | 1 | S | - |
| AIL04829.1 |  | fpg | formamidopyrimidine-DNA glycosylase | 0.36 | -0.14 | -0.02 | NC | NC | NC | 1 | L | ko:K10563 |
| AIL04830.1 |  | scpA | segregation and condensation protein A | -1.21 | -0.66 | 0.08 | D | NC | NC | 6 | D | ko:K05896 |
| AIL04831.1 |  | - | putative secreted lipase | -0.54 | -0.61 | -0.73 | NC | NC | NC | 1 | D | ko:K21471 |
| AIL04832.1 |  | mycA | 67 kDa myosin-cross-reactive antigen like family protein | 1.27 | 0.33 | 0.28 | U | NC | NC | 11 | S | ko:K10254 |
| AIL04833.1 |  | ileS | isoleucine--tRNA ligase | -0.30 | 0.05 | 0.06 | NC | NC | NC | 1 | J | ko:K01870 |
| AIL04836.1 |  | leuS | leucine--tRNA ligase | -0.19 | 0.00 | 0.07 | NC | NC | NC | 1 | J | ko:K01869 |
| AIL04845.1 |  | - | hypothetical protein DR75_1224 | -0.10 | -0.05 | -0.07 | NC | NC | NC | 1 | M | ko:K18149 |
| AIL04848.1 |  | hsdM | type I restriction-modification system, M subunit | -0.20 | -0.03 | 0.08 | NC | NC | NC | 1 | V | ko:K03427 |
| AIL04849.1 |  | nifU | SUF system FeS assembly protein, NifU family | 0.37 | 1.61 | -1.01 | NC | U | D | 18 | C | ko:K04488 |
| AIL04850.1 |  | rplQ | ribosomal protein L17 | -0.30 | -0.19 | -0.94 | NC | NC | NC | 1 | J | ko:K02879 |
| AIL04851.1 |  | - | mazG-like family protein | -0.32 | -0.50 | -1.04 | NC | NC | D | 19 | S | - |
| AIL04857.1 |  | - | putative septicolysin | 0.23 | -0.12 | -0.68 | NC | NC | NC | 1 | - | - |
| AIL04858.1 |  | oatA | acyltransferase family protein | -0.20 | -0.04 | 0.17 | NC | NC | NC | 1 | I | - |
| AIL04860.1 |  | yqfO | NIF3 family protein | -0.18 | 0.26 | -0.11 | NC | NC | NC | 1 | S | - |
| AIL04861.1 |  | ywiB | hypothetical protein DR75_216 | 0.06 | 0.04 | -0.01 | NC | NC | NC | 1 | S | - |
| AIL04862.1 |  | - | hypothetical protein DR75_2666 | 0.18 | 0.42 | 0.29 | NC | NC | NC | 1 | - | - |
| AIL04863.1 |  | trmK | hypothetical protein DR75_454 | 0.87 | 0.87 | 0.55 | NC | NC | NC | 1 | S | ko:K06967 |
| AIL04864.1 |  | rpl | SIS domain protein | -0.05 | 0.03 | 0.37 | NC | NC | NC | 1 | K | - |
| AIL04866.1 |  | rplO | ribosomal protein L15 | -0.07 | 0.15 | -0.02 | NC | NC | NC | 1 | J | ko:K02876 |
| AIL04870.1 |  | pflA | pyruvate formate-lyase 1-activating enzyme | -1.47 | -1.88 | -1.12 | D | D | D | 3 | C | ko:K04069 |
| AIL04872.1 |  | ntpC | V-type sodium ATPase subunit C | 0.49 | 0.23 | -0.40 | NC | NC | NC | 1 | C | ko:K02119 |
| AIL04874.1 |  | malK | ABC transporter family protein | -0.79 | 0.00 | 0.09 | NC | NC | NC | 1 | P | ko:K10112 |
| AIL04876.1 |  | tagF1 | Poly(glycerophosphate) glycerophosphotransferase family protein | 0.33 | 0.28 | -0.35 | NC | NC | NC | 1 | M | ko:K09809 |
| AIL04877.1 |  | pstB2 | phosphate ABC transporter, ATP-binding protein | -0.55 | -0.15 | -0.06 | NC | NC | NC | 1 | P | ko:K02036 |
| AIL04878.1 |  | hsdM | type I restriction-modification system, M subunit | -1.19 | -0.33 | -0.29 | D | NC | NC | 6 | V | ko:K03427 |
| AIL04882.1 |  | rplT | ribosomal protein L20 | -0.33 | -0.70 | -0.78 | NC | NC | NC | 1 | J | ko:K02887 |
| AIL04883.1 |  | kdgA | 2-dehydro-3-deoxyphosphogluconate aldolase/4-hydroxy-2-oxoglutarate aldolase family protein | -1.63 | 0.36 | 0.24 | D | NC | NC | 6 | G | ko:K01625 |
| AIL04884.1 |  | - | L,D-transpeptidase catalytic domain protein | -0.02 | 0.02 | -0.61 | NC | NC | NC | 1 | S | - |
| AIL04885.1 |  | allC | allantoate amidohydrolase | 3.34 | 3.44 | 0.03 | U | U | NC | 9 | E | ko:K02083 |
| AIL04887.1 |  | glxR | NAD binding domain of 6-phosphogluconate dehydrogenase family protein | 0.13 | 0.23 | 0.15 | NC | NC | NC | 1 | I | ko:K00020 |
| AIL04888.1 |  | gyrB | DNA gyrase, B subunit | -0.26 | -0.04 | -0.10 | NC | NC | NC | 1 | L | ko:K02470 |
| AIL04890.1 |  | - | hypothetical protein DR75_2880 | -3.70 | -0.78 | -0.57 | D | NC | NC | 6 | S | - |
| AIL04891.1 |  | yleF | SIS domain protein | 0.45 | 0.48 | -0.01 | NC | NC | NC | 1 | K | - |
| AIL04893.1 |  | - | putative lysine decarboxylase family protein | -0.21 | -0.11 | 0.24 | NC | NC | NC | 1 | S | ko:K06966 |
| AIL04894.1 |  | - | femAB family protein | -0.84 | -0.44 | -0.51 | NC | NC | NC | 1 | V | ko:K05363 |
| AIL04896.1 |  | pdhD | dihydrolipoyl dehydrogenase | -0.66 | 0.26 | 0.18 | NC | NC | NC | 1 | C | ko:K00382 |
| AIL04897.1 |  | yclQ | hypothetical protein DR75_1804 | 1.18 | -0.12 | -0.08 | U | NC | NC | 11 | P | ko:K02016 |
| AIL04898.1 |  | yabA | hypothetical protein DR75_1453 | 0.34 | -0.28 | -0.38 | NC | NC | NC | 1 | L | - |
| AIL04899.1 |  | thiD | phosphomethylpyrimidine kinase | 1.07 | 0.30 | 0.46 | U | NC | NC | 11 | H | ko:K00941 |
| AIL04900.1 |  | - | oxidoreductase, NAD-binding Rossmann fold family protein | -0.97 | -0.76 | -0.96 | NC | NC | NC | 1 | S | - |
| AIL04902.1 |  | - | hypothetical protein DR75_2857 | -0.91 | -2.05 | -1.09 | NC | D | D | 22 | S | - |
| AIL04903.1 |  | ymfH | peptidase M16 inactive domain protein | 0.44 | 0.33 | 0.11 | NC | NC | NC | 1 | S | - |
| AIL04904.1 |  | prmC | protein-(glutamine-N5) methyltransferase, release factor-specific | 3.61 | 0.46 | 4.13 | U | NC | U | 14 | J | ko:K02493 |
| AIL04905.1 |  | - | LPXTG cell wall anchor domain protein | 0.03 | 0.55 | 0.33 | NC | NC | NC | 1 | F | ko:K01081 |
| AIL04906.1 |  | zwf | glucose-6-phosphate dehydrogenase | 0.43 | 0.00 | 0.00 | NC | NC | NC | 1 | G | ko:K00036 |
| AIL04908.1 |  | aroD | 3-dehydroquinate dehydratase | 0.25 | 0.45 | 0.06 | NC | NC | NC | 1 | E | ko:K03785 |
| AIL04910.1 |  | XK27_08845 | ABC transporter family protein | -0.30 | -0.59 | -0.37 | NC | NC | NC | 1 | S | ko:K05833 |
| AIL04911.1 |  | rplF | ribosomal protein L6 | 0.01 | 0.08 | 0.03 | NC | NC | NC | 1 | J | ko:K02933 |
| AIL04912.1 |  | XK27_08630 | hypothetical protein DR75_2540 | -0.07 | -0.36 | -0.62 | NC | NC | NC | 1 | T | ko:K07166 |
| AIL04914.1 |  | yaaN | toxic anion resistance family protein | -0.11 | -0.04 | -0.18 | NC | NC | NC | 1 | P | - |
| AIL04915.1 |  | - | hypothetical protein DR75_2586 | 1.82 | 1.42 | 1.12 | U | U | U | 2 | - | - |
| AIL04916.1 |  | ypcG | hypothetical protein DR75_1068 | 1.34 | 2.14 | 1.46 | U | U | U | 2 | G | ko:K17318 |
| AIL04918.1 |  | purB | adenylosuccinate lyase | -0.15 | -0.03 | 0.02 | NC | NC | NC | 1 | F | ko:K01756 |
| AIL04919.1 |  | - | PTS system sorbose subIIB component family protein | 0.34 | -0.15 | 0.00 | NC | NC | NC | 1 | G | ko:K02794 |
| AIL04920.1 |  | - | PTS system, mannose/fructose/sorbose, IIB component family protein | 0.36 | -0.31 | -0.11 | NC | NC | NC | 1 | G | ko:K02745 |
| AIL04921.1 |  | bdhA | NADH-dependent butanol dehydrogenase A | 0.72 | 0.20 | 0.23 | NC | NC | NC | 1 | C | - |
| AIL04923.1 |  | lipL | biotin/lipoate A/B ligase family protein | 0.20 | 0.33 | 0.32 | NC | NC | NC | 1 | H | ko:K16869 |
| AIL04924.1 |  | - | acetyltransferase family protein | 0.37 | 0.06 | 0.03 | NC | NC | NC | 1 | K | ko:K03827 |
| AIL04926.1 |  | mhqA_2 | glyoxalase/Bleomycin resistance /Dioxygenase superfamily protein | -0.49 | -3.50 | 0.33 | NC | D | NC | 23 | E | ko:K15975 |
| AIL04928.1 |  | thiE | thiamine-phosphate pyrophosphorylase | 0.77 | 1.13 | 0.85 | NC | U | NC | 15 | H | ko:K00788 |
| AIL04930.1 |  | yubA | hypothetical protein DR75_2388 | -1.29 | 1.05 | -1.26 | D | U | D | 17 | S | - |
| AIL04931.1 |  | murI | glutamate racemase | 0.03 | -0.29 | -0.21 | NC | NC | NC | 1 | M | ko:K01776 |
| AIL04933.1 |  | ppdK | pyruvate, phosphate dikinase | 3.77 | 0.41 | 0.19 | U | NC | NC | 11 | G | ko:K01006 |
| AIL04934.1 |  | nrdD | anaerobic ribonucleoside-triphosphate reductase | 0.23 | -0.48 | -0.45 | NC | NC | NC | 1 | FK | ko:K21636 |
| AIL04936.1 |  | ylaN | hypothetical protein DR75_1205 | 0.10 | -0.70 | -0.94 | NC | NC | NC | 1 | S | - |
| AIL04937.1 |  | adcC | ABC transporter family protein | 0.75 | 0.98 | -0.31 | NC | NC | NC | 1 | P | ko:K02074 |
| AIL04942.1 |  | ydaO | amino acid permease family protein | -0.35 | -0.41 | -0.42 | NC | NC | NC | 1 | E | - |
| AIL04943.1 |  | brpA | transcriptional regulator lytR | -0.27 | -0.43 | -0.27 | NC | NC | NC | 1 | K | - |
| AIL04944.1 |  | - | helix-turn-helix domain protein | 0.43 | -0.24 | -0.16 | NC | NC | NC | 1 | K | - |
| AIL04945.1 |  | yhfA | beta-phosphoglucomutase, putative | -0.06 | 0.18 | 0.00 | NC | NC | NC | 1 | S | - |
| AIL04946.1 |  | yaaQ | hypothetical protein DR75_1456 | 0.95 | 0.52 | 0.72 | NC | NC | NC | 1 | S | - |
| AIL04947.1 |  | nrdR | transcriptional regulator NrdR | 0.13 | -0.30 | -0.08 | NC | NC | NC | 1 | K | ko:K07738 |
| AIL04948.1 |  | lplA | lipoyltransferase and lipoate-ligase family protein | -1.66 | -1.43 | -1.88 | D | D | D | 3 | H | ko:K03800 |
| AIL04949.1 |  | - | alpha amylase, catalytic domain protein | 0.82 | 1.34 | 0.95 | NC | U | NC | 15 | G | ko:K01215 |
| AIL04953.1 |  | XK27_03960 | hypothetical protein DR75_937 | -0.10 | -0.29 | -0.40 | NC | NC | NC | 1 | S | - |
| AIL04954.1 |  | XK27_10395 | hypothetical protein DR75_1811 | 0.58 | 0.45 | 0.60 | NC | NC | NC | 1 | S | - |
| AIL04955.1 |  | pts26BCA | PTS system, IIabc component | -1.93 | 0.29 | -0.29 | D | NC | NC | 6 | G | ko:K02808 |
| AIL04957.1 |  | bglX | hypothetical protein DR75_302 | 0.50 | 0.60 | 0.30 | NC | NC | NC | 1 | G | ko:K01207 |
| AIL04959.1 |  | - | sigma-54 interaction domain protein | 0.13 | 0.08 | -0.20 | NC | NC | NC | 1 | K | - |
| AIL04960.1 |  | chbC | PTS system, lactose/cellobiose IIC component family protein | 1.01 | 0.89 | 0.51 | U | NC | NC | 11 | U | ko:K02761 |
| AIL04962.1 |  | pgsA | CDP-diacylglycerol--glycerol-3-phosphate 3-phosphatidyltransferase | 0.58 | 0.12 | -0.01 | NC | NC | NC | 1 | I | ko:K00995 |
| AIL04963.1 |  | - | acetyltransferase domain protein | 0.57 | 0.31 | 0.27 | NC | NC | NC | 1 | S | - |
| AIL04965.1 |  | yajC | preprotein translocase, YajC subunit | -0.06 | -0.61 | -0.29 | NC | NC | NC | 1 | U | ko:K03210 |
| AIL04966.1 |  | acyP | acylphosphatase | 0.25 | 0.16 | 0.48 | NC | NC | NC | 1 | C | ko:K01512 |
| AIL04970.1 |  | XK27_05220 | hypothetical protein DR75_1308 | 0.70 | 0.33 | 0.43 | NC | NC | NC | 1 | S | - |
| AIL04971.1 |  | - | glyoxalase/Bleomycin resistance /Dioxygenase superfamily protein | -0.34 | 0.01 | -0.06 | NC | NC | NC | 1 | S | ko:K07104 |
| AIL04972.1 |  | pepF | oligoendopeptidase F | -0.24 | 0.03 | 0.00 | NC | NC | NC | 1 | E | ko:K08602 |
| AIL04973.1 |  | pyrE | orotate phosphoribosyltransferase | -1.46 | 0.09 | 0.25 | D | NC | NC | 6 | F | ko:K00762 |
| AIL04974.1 |  | lepB | signal peptidase I | 0.04 | -0.31 | -0.04 | NC | NC | NC | 1 | U | ko:K03100 |
| AIL04975.1 |  | mntA | manganese ABC transporter substrate-binding lipoprotein | 0.06 | -0.13 | -0.22 | NC | NC | NC | 1 | P | ko:K19971 |
| AIL04976.1 |  | dnaX | DNA polymerase III, subunit gamma and tau | 0.09 | 0.25 | 0.13 | NC | NC | NC | 1 | L | ko:K02343 |
| AIL04979.1 |  | nadE | NAD+ synthetase | 0.47 | 0.05 | -0.07 | NC | NC | NC | 1 | H | ko:K01916 |
| AIL04981.1 |  | trmD | tRNA (guanine(37)-N(1))-methyltransferase | -0.68 | -0.54 | -0.07 | NC | NC | NC | 1 | J | ko:K00554 |
| AIL04982.1 |  | - | PTS system, Lactose/Cellobiose specific IIB subunit | 0.44 | -0.02 | 0.25 | NC | NC | NC | 1 | - | - |
| AIL04983.1 |  | - | short chain dehydrogenase family protein | -0.14 | 0.30 | 0.44 | NC | NC | NC | 1 | IQ | ko:K00046 |
| AIL04984.1 |  | rny | ribonuclease Y | -0.18 | -0.09 | 0.00 | NC | NC | NC | 1 | S | ko:K18682 |
| AIL04985.1 |  | prfA | peptide chain release factor 1 | -0.56 | -0.49 | -0.21 | NC | NC | NC | 1 | J | ko:K02835 |
| AIL04987.1 |  | msmX | ABC transporter family protein | 0.73 | 1.72 | 1.59 | NC | U | U | 4 | E | ko:K10112 |
| AIL04989.1 |  | - | DnaD domain protein | 1.92 | 0.43 | 0.31 | U | NC | NC | 11 | L | - |
| AIL04990.1 |  | oppA2 | bacterial extracellular solute-binding s, 5 Middle family protein | 0.08 | 1.04 | 1.10 | NC | U | U | 4 | E | ko:K15580 |
| AIL04991.1 |  | topA | DNA topoisomerase I | -0.64 | -0.21 | -0.47 | NC | NC | NC | 1 | L | ko:K03168 |
| AIL04992.1 |  | - | hypothetical protein DR75_1780 | 0.20 | 0.15 | -1.40 | NC | NC | D | 19 | - | - |
| AIL04994.1 |  | - | hypothetical protein DR75_436 | 1.24 | -1.15 | 0.29 | U | D | NC | 12 | - | - |
| AIL04995.1 |  | pstB1 | phosphate ABC transporter, ATP-binding protein | -0.20 | -0.14 | -0.09 | NC | NC | NC | 1 | P | ko:K02036 |
| AIL04996.1 |  | yhbY | CRS1 / YhbY domain protein | -0.01 | -0.59 | -0.60 | NC | NC | NC | 1 | J | ko:K07574 |
| AIL04999.1 |  | yleF | SIS domain protein | 0.69 | 1.60 | 0.81 | NC | U | NC | 15 | K | - |
| AIL05000.1 |  | ltrC | hypothetical protein DR75_2588 | 0.01 | 0.49 | 0.19 | NC | NC | NC | 1 | I | - |
| AIL05003.1 |  | spo0J | stage 0 sporulation protein J | -0.31 | -0.14 | -0.42 | NC | NC | NC | 1 | K | ko:K03497 |
| AIL05004.1 |  | ctsR | transcriptional regulator CtsR | 0.34 | -0.16 | -0.20 | NC | NC | NC | 1 | K | ko:K03708 |
| AIL05007.1 |  | ysnB | phosphodiesterase, family protein | 0.93 | 0.89 | 0.61 | NC | NC | NC | 1 | S | ko:K07095 |
| AIL05009.1 |  | miaA | tRNA dimethylallyltransferase | -1.94 | -1.27 | -1.39 | D | D | D | 3 | F | ko:K00791 |
| AIL05010.1 |  | pgk | phosphoglycerate kinase | 0.46 | 0.47 | 0.34 | NC | NC | NC | 1 | F | ko:K00927 |
| AIL05011.1 |  | - | mepB family protein | 0.10 | -0.33 | -0.50 | NC | NC | NC | 1 | S | - |
| AIL05012.1 |  | glnQ | ABC transporter family protein | -0.02 | -0.21 | -0.31 | NC | NC | NC | 1 | E | ko:K02028 |
| AIL05015.1 |  | oppF | ABC transporter family protein | -0.25 | -0.39 | -0.39 | NC | NC | NC | 1 | E | ko:K02032 |
| AIL05018.1 |  | XK27_08635 | hypothetical protein DR75_2541 | -0.79 | -0.07 | -0.12 | NC | NC | NC | 1 | S | ko:K09157 |
| AIL05019.1 |  | manO | hypothetical protein DR75_2072 | 0.59 | 0.65 | 0.38 | NC | NC | NC | 1 | S | - |
| AIL05020.1 |  | licT | transcription antiterminator LicT | 0.13 | 1.08 | 0.28 | NC | U | NC | 15 | K | ko:K03488 |
| AIL05023.1 |  | - | hypothetical protein DR75_2412 | 0.39 | -0.04 | -0.14 | NC | NC | NC | 1 | S | - |
| AIL05026.1 |  | pyrH | UMP kinase | -0.38 | -0.04 | -0.06 | NC | NC | NC | 1 | F | ko:K09903 |
| AIL05027.1 |  | hpk31 | his Kinase A domain protein | 0.88 | 0.21 | -0.42 | NC | NC | NC | 1 | T | - |
| AIL05028.1 |  | thiI | tRNA sulfurtransferase ThiI | -0.26 | -0.45 | 0.31 | NC | NC | NC | 1 | H | ko:K03151 |
| AIL05030.1 |  | - | beta-lactamase family protein | 2.43 | 2.11 | 2.04 | U | U | U | 2 | V | - |
| AIL05031.1 |  | rgpD | ABC transporter family protein | -0.19 | -0.37 | -0.24 | NC | NC | NC | 1 | GM | ko:K01990 |
| AIL05032.1 |  | nth | endonuclease III | -0.55 | -0.29 | -0.68 | NC | NC | NC | 1 | L | ko:K10773 |
| AIL05034.1 |  | potD | bacterial extracellular solute-binding family protein | -1.04 | 0.75 | -0.32 | D | NC | NC | 6 | E | ko:K11069 |
| AIL05035.1 |  | rnpA | ribonuclease P protein component | 0.44 | -0.15 | -0.18 | NC | NC | NC | 1 | J | ko:K03536 |
| AIL05036.1 |  | yvqF | hypothetical protein DR75_1608 | 0.21 | -0.20 | -0.12 | NC | NC | NC | 1 | S | ko:K11622 |
| AIL05037.1 |  | ecfT | cobalt transport family protein | -0.41 | -0.60 | -0.64 | NC | NC | NC | 1 | U | ko:K16785 |
| AIL05039.1 |  | sufC | FeS assembly ATPase SufC | -0.04 | 0.08 | 0.43 | NC | NC | NC | 1 | O | ko:K09013 |
| AIL05041.1 |  | dapF | diaminopimelate epimerase | -0.24 | -0.03 | 0.14 | NC | NC | NC | 1 | E | ko:K01778 |
| AIL05043.1 |  | arbF3 | PTS system, beta-glucoside-specific IIABC component family protein | 2.30 | 2.18 | 1.87 | U | U | U | 2 | G | ko:K02755 |
| AIL05047.1 |  | fat | acyl-ACP thioesterase family protein | 0.14 | 0.10 | 0.00 | NC | NC | NC | 1 | I | ko:K01071 |
| AIL05049.1 |  | cadA | cadmium-translocating P-type ATPase | -1.47 | -1.62 | -0.88 | D | D | NC | 20 | P | - |
| AIL05051.1 |  | recO | DNA repair protein RecO | 0.87 | 0.81 | 0.26 | NC | NC | NC | 1 | L | ko:K03584 |
| AIL05052.1 |  | citE | citrate (pro-3S)-lyase, beta subunit | 0.66 | 0.47 | 0.14 | NC | NC | NC | 1 | H | ko:K01644 |
| AIL05053.1 |  | sdaAA | L-serine dehydratase, iron-sulfur-dependent, alpha subunit | 0.04 | 0.29 | 0.05 | NC | NC | NC | 1 | E | ko:K01752 |
| AIL05054.1 |  | - | hypothetical protein DR75_1202 | -0.35 | -0.10 | -0.21 | NC | NC | NC | 1 | S | - |
| AIL05055.1 |  | azoR | FMN-dependent NADH-azoreductase 2 | -1.01 | -0.34 | -0.77 | D | NC | NC | 6 | I | ko:K01118 |
| AIL05056.1 |  | - | putative coenzyme F420 hydrogenase domain protein | 0.01 | -0.04 | -1.13 | NC | NC | D | 19 | - | - |
| AIL05058.1 |  | ytsP | putative gAF domain protein | 0.40 | 0.48 | 0.62 | NC | NC | NC | 1 | T | ko:K08968 |
| AIL05059.1 |  | comEB | ComE operon protein 2 | 0.47 | 0.72 | -0.30 | NC | NC | NC | 1 | F | ko:K01493 |
| AIL05060.1 |  | ltaS | type I phosphodiesterase / nucleotide pyrophosphatase family protein | 0.51 | 0.72 | 0.48 | NC | NC | NC | 1 | M | ko:K19005 |
| AIL05062.1 |  | - | bacteriocin-protection, YdeI/OmpD-Associated family protein | -0.94 | -0.34 | 0.14 | NC | NC | NC | 1 | S | - |
| AIL05065.1 |  | menB | naphthoate synthase | 0.32 | -0.21 | 0.11 | NC | NC | NC | 1 | H | ko:K01661 |
| AIL05066.1 |  | yitK | hypothetical protein DR75_236 | 0.49 | 0.06 | -0.04 | NC | NC | NC | 1 | S | ko:K09767 |
| AIL05067.1 |  | dtd | D-tyrosyl-tRNA(Tyr) deacylase | 0.57 | 0.12 | 0.51 | NC | NC | NC | 1 | J | ko:K07560 |
| AIL05068.1 |  | - | aryl-phospho-beta-D-glucosidase BglA | 0.28 | 2.71 | 2.03 | NC | U | U | 4 | G | ko:K01223 |
| AIL05071.1 |  | - | phosphoglycerate mutase 1 family protein | -0.31 | -0.79 | -0.30 | NC | NC | NC | 1 | G | ko:K01834 |
| AIL05075.1 |  | pta | phosphate acetyltransferase | -0.46 | 0.01 | -0.13 | NC | NC | NC | 1 | C | ko:K00625 |
| AIL05076.1 |  | ybbM | hypothetical protein DR75_1450 | -0.76 | 0.02 | -0.14 | NC | NC | NC | 1 | S | ko:K02069 |
| AIL05078.1 |  | acmA | muramidase-2 | -0.28 | -0.29 | -0.53 | NC | NC | NC | 1 | MNU | ko:K02395 |
| AIL05079.1 |  | tenA | thiaminase II | 0.72 | 0.49 | 0.74 | NC | NC | NC | 1 | K | ko:K03707 |
| AIL05080.1 |  | cadA | copper-translocating P-type ATPase | 1.53 | -0.27 | -0.49 | U | NC | NC | 11 | P | ko:K01534 |
| AIL05081.1 |  | - | wxL domain surface cell wall-binding family protein | 2.39 | 2.05 | 1.14 | U | U | U | 2 | S | - |
| AIL05082.1 |  | maa | chorismate mutase | 0.07 | -0.20 | 0.00 | NC | NC | NC | 1 | E | ko:K00661 |
| AIL05083.1 |  | hipO | amidohydrolase family protein | 3.14 | 3.08 | 3.09 | U | U | U | 2 | E | ko:K05823 |
| AIL05085.1 |  | ftsK | DNA translocase ftsK | 0.05 | -0.26 | -0.37 | NC | NC | NC | 1 | D | ko:K03466 |
| AIL05090.1 |  | metK | methionine adenosyltransferase | 0.66 | 0.35 | 0.10 | NC | NC | NC | 1 | H | ko:K00789 |
| AIL05091.1 |  | - | hypothetical protein DR75_1504 | 0.54 | -1.08 | 0.17 | NC | D | NC | 23 | - | - |
| AIL05092.1 |  | - | hypothetical protein DR75_433 | -0.09 | 1.02 | 0.90 | NC | U | NC | 15 | G | ko:K05881 |
| AIL05095.1 |  | fhuC | ABC transporter family protein | 1.20 | 0.23 | 0.08 | U | NC | NC | 11 | HP | ko:K02013 |
| AIL05096.1 |  | rbsK | ribokinase | 0.02 | -1.02 | -0.25 | NC | D | NC | 23 | H | ko:K00852 |
| AIL05098.1 |  | - | SPFH domain / Band 7 family protein | 0.18 | 0.59 | 0.58 | NC | NC | NC | 1 | S | ko:K07192 |
| AIL05100.1 |  | vanR | hypothetical protein DR75_1998 | 0.29 | -0.21 | -0.09 | NC | NC | NC | 1 | K | - |
| AIL05101.1 |  | - | hypothetical protein DR75_73 | 0.88 | 0.80 | 0.72 | NC | NC | NC | 1 | - | - |
| AIL05103.1 |  | gmk2 | guanylate kinase family protein | 0.65 | -0.77 | -0.03 | NC | NC | NC | 1 | F | ko:K00942 |
| AIL05105.1 |  | rfbA | glucose-1-phosphate thymidylyltransferase | -0.35 | -0.14 | -0.19 | NC | NC | NC | 1 | H | ko:K00973 |
| AIL05106.1 |  | mccA | pyridoxal-phosphate dependent enzyme family protein | -1.52 | -0.26 | -0.26 | D | NC | NC | 6 | E | ko:K01738 |
| AIL05107.1 |  | - | hypothetical protein DR75_1731 | -0.19 | 0.59 | -0.13 | NC | NC | NC | 1 | - | - |
| AIL05110.1 |  | - | bacterial regulatory s, tetR family protein | 0.16 | 0.12 | -0.64 | NC | NC | NC | 1 | K | - |
| AIL05111.1 |  | panB | 3-methyl-2-oxobutanoate hydroxymethyltransferase | -0.63 | -0.12 | -0.12 | NC | NC | NC | 1 | H | ko:K00606 |
| AIL05113.1 |  | fni | isopentenyl-diphosphate delta-isomerase, type 2 | -0.18 | -0.32 | -0.10 | NC | NC | NC | 1 | C | ko:K01823 |
| AIL05114.1 |  | yhcC | hypothetical protein DR75_1950 | 0.21 | 0.87 | 1.04 | NC | NC | U | 5 | S | ko:K07069 |
| AIL05117.1 |  | pnuC | nicotinamide mononucleotide transporter PnuC family protein | 1.40 | 0.06 | -0.21 | U | NC | NC | 11 | H | ko:K03811 |
| AIL05120.1 |  | serS | serine--tRNA ligase | -0.48 | 0.05 | -1.11 | NC | NC | D | 19 | J | ko:K01875 |
| AIL05122.1 |  | rnjB | metallo-beta-lactamase superfamily protein | 0.20 | 0.03 | -0.01 | NC | NC | NC | 1 | J | ko:K12574 |
| AIL05123.1 |  | - | phosphoenolpyruvate-dependent sugar phosphotransferase system, EIIA 2 family protein | 1.67 | 1.16 | 0.61 | U | U | NC | 9 | G | ko:K02773 |
| AIL05124.1 |  | ppiB | cyclophilin type peptidyl-prolyl cis-trans isomerase/CLD family protein | 0.09 | 0.20 | 0.24 | NC | NC | NC | 1 | O | ko:K03768 |
| AIL05126.1 |  | ylbF | hypothetical protein DR75_1201 | 0.21 | -0.14 | 0.08 | NC | NC | NC | 1 | S | - |
| AIL05127.1 |  | hflX | GTP-binding protein HflX | 0.38 | -0.19 | -0.32 | NC | NC | NC | 1 | S | ko:K03665 |
| AIL05128.1 |  | frr | ribosome recycling factor | -0.11 | 0.17 | -0.33 | NC | NC | NC | 1 | J | ko:K02838 |
| AIL05131.1 |  | - | C4-dicarboxylate anaerobic carrier family protein | 0.95 | 0.99 | 0.84 | NC | NC | NC | 1 | S | - |
| AIL05132.1 |  | ypjC | hypothetical protein DR75_558 | -0.51 | -0.22 | -0.35 | NC | NC | NC | 1 | S | - |
| AIL05133.1 |  | - | hypothetical protein DR75_1666 | 0.89 | -0.73 | -0.52 | NC | NC | NC | 1 | - | - |
| AIL05134.1 |  | ung2 | uracil DNA glycosylase superfamily protein | 1.32 | 0.44 | -0.18 | U | NC | NC | 11 | L | ko:K21929 |
| AIL05136.1 |  | lexA | repressor LexA | -0.57 | 0.07 | 0.18 | NC | NC | NC | 1 | K | ko:K01356 |
| AIL05138.1 |  | glpF | MIP channel s family protein | 0.33 | 0.84 | 0.33 | NC | NC | NC | 1 | U | ko:K02440 |
| AIL05140.1 |  | rnhA | 14.7 kDa ribonuclease H-like protein | -1.92 | -0.89 | -1.92 | D | NC | D | 8 | L | ko:K03469 |
| AIL05143.1 |  | yvrA | ABC transporter family protein | -0.23 | 0.21 | 0.38 | NC | NC | NC | 1 | HP | ko:K02013 |
| AIL05145.1 |  | yrrN | peptidase U32 family protein | -0.38 | -0.35 | -0.37 | NC | NC | NC | 1 | O | ko:K08303 |
| AIL05147.1 |  | - | hypothetical protein DR75_707 | 0.21 | 0.90 | 0.28 | NC | NC | NC | 1 | S | ko:K09704 |
| AIL05148.1 |  | - | hypothetical protein DR75_1040 | -0.02 | 0.02 | 0.17 | NC | NC | NC | 1 | - | - |
| AIL05151.1 |  | ypiB | hypothetical protein DR75_557 | 5.91 | -0.11 | -0.24 | U | NC | NC | 11 | S | - |
| AIL05152.1 |  | rrp1 | response regulator | -0.27 | -0.07 | -0.21 | NC | NC | NC | 1 | K | ko:K02483 |
| AIL05156.1 |  | - | hypothetical protein DR75_2043 | 1.47 | 1.35 | 0.34 | U | U | NC | 9 | - | - |
| AIL05157.1 |  | sufB | FeS assembly protein SufB | 0.14 | 0.01 | 0.00 | NC | NC | NC | 1 | O | ko:K07033 |
| AIL05159.1 |  | recF | DNA replication and repair RecF family protein | -0.70 | 0.76 | 0.55 | NC | NC | NC | 1 | L | ko:K03629 |
| AIL05160.1 |  | yphA | 3-beta hydroxysteroid dehydrogenase/isomerase family protein | 1.02 | -0.56 | -0.17 | U | NC | NC | 11 | GM | - |
| AIL05161.1 |  | opuAA | glycine betaine/L-proline transport ATP binding subunit | -0.02 | 0.12 | -0.10 | NC | NC | NC | 1 | E | ko:K02000 |
| AIL05162.1 |  | pncB | nicotinate phosphoribosyltransferase family protein | 0.12 | 0.04 | -0.03 | NC | NC | NC | 1 | F | ko:K00763 |
| AIL05164.1 |  | yrrO | peptidase U32 family protein | -0.63 | -0.62 | -0.50 | NC | NC | NC | 1 | O | ko:K08303 |
| AIL05165.1 |  | mvaS | hydroxymethylglutaryl-CoA synthase | -0.07 | -0.15 | -0.33 | NC | NC | NC | 1 | I | ko:K01641 |
| AIL05167.1 |  | - | lipid kinase, YegS//BmrU family protein | 0.60 | -0.04 | 0.55 | NC | NC | NC | 1 | I | - |
| AIL05168.1 |  | gpmB | histidine phosphatase super family protein | 0.35 | 0.04 | -0.08 | NC | NC | NC | 1 | G | ko:K15640 |
| AIL05169.1 |  | ykpA | ABC transporter family protein | -0.16 | -0.01 | 0.01 | NC | NC | NC | 1 | S | - |
| AIL05170.1 |  | gldA | iron-containing alcohol dehydrogenase family protein | -0.29 | 0.36 | 0.28 | NC | NC | NC | 1 | C | ko:K08317 |
| AIL05172.1 |  | ccpA | catabolite control protein A | -0.73 | -0.23 | -0.21 | NC | NC | NC | 1 | K | ko:K02529 |
| AIL05173.1 |  | gpsB | DivIVA domain protein | 0.08 | 0.02 | 0.01 | NC | NC | NC | 1 | D | - |
| AIL05174.1 |  | glcK | ROK family protein | -0.18 | -0.18 | -0.04 | NC | NC | NC | 1 | G | ko:K00845 |
| AIL05178.1 |  | - | hypothetical protein DR75_790 | 1.10 | 0.61 | 0.47 | U | NC | NC | 11 | M | - |
| AIL05180.1 |  | copB | copper-translocating P-type ATPase | 0.42 | 0.36 | -0.04 | NC | NC | NC | 1 | P | ko:K01533 |
| AIL05181.1 |  | M1-1000 | response regulator | 0.15 | 0.33 | -0.15 | NC | NC | NC | 1 | K | ko:K02475 |
| AIL05183.1 |  | - | GDSL-like Lipase/Acylhydrolase family protein | 1.26 | 0.14 | 0.14 | U | NC | NC | 11 | E | - |
| AIL05184.1 |  | araT | beta-eliminating lyase family protein | -0.51 | -0.35 | -0.32 | NC | NC | NC | 1 | E | ko:K00841 |
| AIL05185.1 |  | yvoA_2 | UTRA domain protein | -0.60 | -0.09 | 0.00 | NC | NC | NC | 1 | K | ko:K03710 |
| AIL05189.1 |  | - | HAD hydrolase, IA, variant 1 family protein | -0.21 | 0.10 | -0.10 | NC | NC | NC | 1 | S | - |
| AIL05191.1 |  | hepT | polyprenyl synthetase family protein | -0.02 | 0.04 | 0.07 | NC | NC | NC | 1 | H | ko:K00805 |
| AIL05192.1 |  | ftsA | cell division protein FtsA | -0.15 | -0.08 | -0.10 | NC | NC | NC | 1 | D | ko:K03590 |
| AIL05193.1 |  | - | phosphoribosyl-ATP pyrophosphohydrolase family protein | 0.12 | 0.02 | -0.22 | NC | NC | NC | 1 | S | ko:K16785 |
| AIL05194.1 |  | xseB | exodeoxyribonuclease VII, small subunit | -0.70 | 0.16 | 0.46 | NC | NC | NC | 1 | L | ko:K03602 |
| AIL05195.1 |  | femA | femAB family protein | -0.10 | -0.20 | -0.06 | NC | NC | NC | 1 | V | ko:K12554 |
| AIL05196.1 |  | secA | preprotein translocase, SecA subunit | -0.46 | -0.17 | -0.23 | NC | NC | NC | 1 | U | ko:K03070 |
| AIL05197.1 |  | XK27_08360 | EDD, DegV family domain protein | -0.34 | -0.26 | -0.13 | NC | NC | NC | 1 | S | - |
| AIL05199.1 |  | - | hypothetical protein DR75_493 | 1.91 | -0.99 | -1.27 | U | NC | D | 10 | - | ko:K02107 |
| AIL05200.1 |  | uvrA2 | sigma-54 interaction domain protein | -0.08 | -0.07 | 0.47 | NC | NC | NC | 1 | L | - |
| AIL05201.1 |  | - | helix-turn-helix family protein | -0.79 | -0.35 | -0.32 | NC | NC | NC | 1 | K | ko:K20342 |
| AIL05202.1 |  | - | putative yycH protein | -0.17 | -0.71 | -0.60 | NC | NC | NC | 1 | S | - |
| AIL05203.1 |  | yqeK | HD domain protein | 0.36 | -0.47 | -0.92 | NC | NC | NC | 1 | H | - |
| AIL05204.1 |  | plsX | fatty acid/phospholipid synthesis protein PlsX | 0.15 | -0.26 | -0.13 | NC | NC | NC | 1 | I | ko:K03621 |
| AIL05205.1 |  | rpoN | RNA polymerase sigma-54 factor | -0.29 | -0.34 | -0.29 | NC | NC | NC | 1 | K | ko:K03092 |
| AIL05206.1 |  | rplR | ribosomal protein L18 | -0.53 | -0.36 | -0.37 | NC | NC | NC | 1 | J | ko:K02881 |
| AIL05207.1 |  | - | hypothetical protein DR75_1226 | 0.30 | -0.03 | 0.28 | NC | NC | NC | 1 | S | - |
| AIL05209.1 |  | pyrD | dihydroorotate dehydrogenase | -0.09 | 0.32 | 0.04 | NC | NC | NC | 1 | F | ko:K00226 |
| AIL05210.1 |  | cadA | copper-translocating P-type ATPase | -0.58 | -0.10 | -0.21 | NC | NC | NC | 1 | P | ko:K01534 |
| AIL05213.1 |  | iscS2 | beta-eliminating lyase family protein | -0.56 | -0.34 | -0.42 | NC | NC | NC | 1 | E | ko:K04487 |
| AIL05215.1 |  | proS | proline--tRNA ligase | -0.41 | -0.13 | -0.21 | NC | NC | NC | 1 | J | ko:K01881 |
| AIL05216.1 |  | ansB | asparaginase family protein | 0.54 | -0.05 | 0.04 | NC | NC | NC | 1 | EJ | ko:K01424 |
| AIL05217.1 |  | - | recT family protein | 0.98 | -0.43 | -0.13 | NC | NC | NC | 1 | L | ko:K07455 |
| AIL05218.1 |  | - | helix-turn-helix domain protein | 1.06 | -1.07 | -0.70 | U | D | NC | 12 | L | - |
| AIL05219.1 |  | - | ABC transporter family protein | 0.87 | 0.31 | 0.22 | NC | NC | NC | 1 | V | ko:K06147 |
| AIL05221.1 |  | mvaD | diphosphomevalonate decarboxylase | -0.26 | -0.15 | -0.50 | NC | NC | NC | 1 | I | ko:K01597 |
| AIL05223.1 |  | - | voltage gated chloride channel family protein | 0.50 | 0.87 | 1.00 | NC | NC | NC | 1 | P | - |
| AIL05224.1 |  | - | hypothetical protein DR75_576 | -0.80 | -1.02 | -0.81 | NC | D | NC | 23 | - | - |
| AIL05225.1 |  | rplP | ribosomal protein L16 | -0.01 | -0.04 | -0.12 | NC | NC | NC | 1 | J | ko:K02878 |
| AIL05226.1 |  | fba | fructose-1,6-bisphosphate aldolase, class II | -0.64 | -0.10 | -0.16 | NC | NC | NC | 1 | G | ko:K01624 |
| AIL05228.1 |  | - | pyridine nucleotide-disulfide oxidoreductase family protein | -1.91 | -1.08 | -0.76 | D | D | NC | 20 | O | ko:K00384 |
| AIL05229.1 |  | vncS | HAMP domain protein | -1.89 | 0.07 | -0.13 | D | NC | NC | 6 | T | ko:K10819 |
| AIL05230.1 |  | - | AAA domain protein | -0.32 | -0.51 | -0.37 | NC | NC | NC | 1 | E | - |
| AIL05231.1 |  | ytpR | putative tRNA binding domain protein | -0.08 | -0.50 | -0.32 | NC | NC | NC | 1 | J | ko:K06878 |
| AIL05232.1 |  | - | dihydrofolate reductase | 0.37 | 0.24 | 0.15 | NC | NC | NC | 1 | H | - |
| AIL05234.1 |  | ydiB | tRNA threonylcarbamoyl adenosine modification protein YjeE | -0.04 | 0.08 | 0.09 | NC | NC | NC | 1 | S | ko:K06925 |
| AIL05235.1 |  | - | hypothetical protein DR75_2327 | 0.27 | 2.08 | -0.43 | NC | U | NC | 15 | S | ko:K06950 |
| AIL05236.1 |  | pstS | phosphate binding family protein | 0.06 | -0.79 | -0.53 | NC | NC | NC | 1 | P | ko:K02040 |
| AIL05239.1 |  | - | hypothetical protein DR75_1230 | -1.69 | -0.15 | 0.19 | D | NC | NC | 6 | - | - |
| AIL05240.1 |  | yusI | putative arsenate reductase | 0.07 | 0.14 | -0.04 | NC | NC | NC | 1 | P | ko:K00537 |
| AIL05241.1 |  | xylR | ROK family protein | -0.44 | -0.28 | -0.01 | NC | NC | NC | 1 | GK | - |
| AIL05242.1 |  | - | iron-sulfur cluster biosynthesis family protein | 0.57 | 0.19 | 0.01 | NC | NC | NC | 1 | S | - |
| AIL05243.1 |  | thiD | hydroxyethylthiazole kinase family protein | -0.46 | -0.43 | -0.55 | NC | NC | NC | 1 | H | ko:K00868 |
| AIL05245.1 |  | cryZ | zinc-binding dehydrogenase family protein | 0.76 | 0.56 | 0.42 | NC | NC | NC | 1 | C | - |
| AIL05247.1 |  | ydhF | aldo/keto reductase family protein | 0.24 | 0.35 | 0.26 | NC | NC | NC | 1 | S | - |
| AIL05248.1 |  | - | hypothetical protein DR75_2009 | 0.42 | -0.08 | -0.27 | NC | NC | NC | 1 | S | - |
| AIL05249.1 |  | cls | cardiolipin synthase | -0.01 | 0.18 | -0.32 | NC | NC | NC | 1 | I | ko:K06131 |
| AIL05250.1 |  | mdlB | ABC transporter family protein | 0.19 | 0.43 | -0.78 | NC | NC | NC | 1 | V | ko:K06147 |
| AIL05253.1 |  | rodA | cell cycle family protein | 0.21 | -0.40 | -0.62 | NC | NC | NC | 1 | D | ko:K05837 |
| AIL05254.1 |  | estA | alpha/beta hydrolase fold family protein | 0.34 | -0.09 | 0.06 | NC | NC | NC | 1 | S | ko:K03930 |
| AIL05257.1 |  | - | putative thioesterase/dihydrolipoamide acyltransferase | -0.49 | -0.65 | -0.10 | NC | NC | NC | 1 | S | - |
| AIL05258.1 |  | infB | translation initiation factor IF-2 | 0.15 | -0.47 | -0.45 | NC | NC | NC | 1 | J | ko:K02519 |
| AIL05259.1 |  | adh | aldehyde dehydrogenase family protein | -0.34 | 0.23 | -0.65 | NC | NC | NC | 1 | C | ko:K04072 |
| AIL05261.1 |  | hom | oxidoreductase, NAD-binding Rossmann fold family protein | 0.26 | -0.25 | -0.25 | NC | NC | NC | 1 | E | ko:K00003 |
| AIL05262.1 |  | citR | bacterial regulatory s, gntR family protein | 0.41 | 0.62 | 0.51 | NC | NC | NC | 1 | K | - |
| AIL05264.1 |  | rpsC | ribosomal protein S3 | -0.11 | 0.21 | -0.24 | NC | NC | NC | 1 | J | ko:K02982 |
| AIL05265.1 |  | - | phage family protein | 1.10 | 0.62 | -0.24 | U | NC | NC | 11 | S | - |
| AIL05267.1 |  | yleB | hypothetical protein DR75_1183 | -0.29 | 0.54 | -0.22 | NC | NC | NC | 1 | S | ko:K07106 |
| AIL05268.1 |  | yybA | marR family protein | 0.48 | 0.30 | 0.04 | NC | NC | NC | 1 | K | - |
| AIL05271.1 |  | galU | UTP-glucose-1-phosphate uridylyltransferase | -0.09 | -0.22 | -0.31 | NC | NC | NC | 1 | M | ko:K00963 |
| AIL05272.1 |  | uppS | di-trans,poly-cis-decaprenylcistransferase | -0.65 | -0.15 | -0.06 | NC | NC | NC | 1 | I | ko:K00806 |
| AIL05273.1 |  | cls | cardiolipin synthase | 0.73 | 1.11 | 0.87 | NC | U | NC | 15 | I | ko:K06131 |
| AIL05274.1 |  | - | phosphoenolpyruvate-dependent sugar phosphotransferase system, EIIA 2 family protein | -2.09 | -0.17 | -0.31 | D | NC | NC | 6 | G | ko:K02798 |
| AIL05276.1 |  | ywlG | hypothetical protein DR75_928 | -0.15 | -0.22 | -0.36 | NC | NC | NC | 1 | S | - |
| AIL05278.1 |  | cydC | thiol reductant ABC exporter, CydD subunit | -0.49 | -0.13 | -0.34 | NC | NC | NC | 1 | CO | ko:K16013 |
| AIL05279.1 |  | cysS | cysteine--tRNA ligase | -0.48 | -0.01 | -0.19 | NC | NC | NC | 1 | J | ko:K01883 |
| AIL05280.1 |  | - | hypothetical protein DR75_789 | 0.94 | -0.07 | 0.03 | NC | NC | NC | 1 | S | - |
| AIL05282.1 |  | - | glycosyl transferase 2 family protein | -0.27 | -0.53 | -0.27 | NC | NC | NC | 1 | S | - |
| AIL05283.1 |  | psaA3 | periplasmic solute binding family protein | -0.34 | -0.22 | -0.02 | NC | NC | NC | 1 | P | ko:K09815 |
| AIL05285.1 |  | - | DNA/RNA non-specific endonuclease family protein | -0.10 | 0.03 | 0.20 | NC | NC | NC | 1 | F | ko:K15051 |
| AIL05286.1 |  | yrrM | cephalosporin hydroxylase family protein | 0.09 | 0.46 | -0.32 | NC | NC | NC | 1 | S | ko:K00588 |
| AIL05289.1 |  | pi112 | hypothetical protein DR75_1661 | 0.41 | -0.06 | 0.02 | NC | NC | NC | 1 | L | - |
| AIL05290.1 |  | - | ABC transporter family protein | -0.85 | -0.55 | -0.35 | NC | NC | NC | 1 | V | ko:K02003 |
| AIL05295.1 |  | rsmD | RNA methyltransferase, RsmD family | -0.10 | 0.09 | -0.17 | NC | NC | NC | 1 | L | ko:K08316 |
| AIL05297.1 |  | yqgX | hypothetical protein DR75_1178 | 0.29 | 0.58 | 0.24 | NC | NC | NC | 1 | S | ko:K01069 |
| AIL05300.1 |  | - | ribosomal L23 family protein | 0.91 | -0.48 | 1.36 | NC | NC | U | 5 | - | - |
| AIL05301.1 |  | srlB | PTS system glucitol/sorbitol-specific IIA component family protein | 0.11 | 0.45 | 0.43 | NC | NC | NC | 1 | G | ko:K02781 |
| AIL05302.1 |  | xpt | xanthine phosphoribosyltransferase | -0.44 | -0.05 | -0.12 | NC | NC | NC | 1 | F | ko:K03816 |
| AIL05309.1 |  | pyrB | aspartate carbamoyltransferase | -1.88 | 0.44 | 0.40 | D | NC | NC | 6 | F | ko:K00609 |
| AIL05312.1 |  | - | hypothetical protein DR75_2764 | 0.75 | 0.26 | 0.15 | NC | NC | NC | 1 | K | - |
| AIL05313.1 |  | glyA | beta-eliminating lyase family protein | 0.18 | 0.21 | 0.21 | NC | NC | NC | 1 | E | ko:K00600 |
| AIL05314.1 |  | - | putative lipoprotein | -0.18 | -0.71 | -0.30 | NC | NC | NC | 1 | - | - |
| AIL05315.1 |  | apc3 | hydantoinase/oxoprolinase family protein | -1.15 | -2.23 | -2.49 | D | D | D | 3 | EQ | - |
| AIL05316.1 |  | ascB | glycosyl hydrolase 1 family protein | 0.47 | 1.92 | 1.45 | NC | U | U | 4 | G | ko:K01223 |
| AIL05319.1 |  | fabZ | beta-hydroxyacyl-(acyl-carrier-protein) dehydratase FabZ | -0.28 | 0.33 | 0.06 | NC | NC | NC | 1 | I | ko:K02372 |
| AIL05321.1 |  | ntpI | V-type ATPase subunit | -0.38 | -0.17 | -0.05 | NC | NC | NC | 1 | U | ko:K02123 |
| AIL05322.1 |  | copB | copper-translocating P-type ATPase | 0.82 | 0.00 | -0.06 | NC | NC | NC | 1 | P | ko:K01533 |
| AIL05323.1 |  | - | transcriptional regulator PadR-like family protein | 0.29 | -1.62 | -0.57 | NC | D | NC | 23 | K | ko:K10947 |
| AIL05325.1 |  | - | phage major tail, phi13 family protein | 0.30 | -0.01 | -0.10 | NC | NC | NC | 1 | N | - |
| AIL05326.1 |  | - | amidase family protein | 0.06 | -0.11 | -0.22 | NC | NC | NC | 1 | J | ko:K01426 |
| AIL05327.1 |  | sorD | short chain dehydrogenase family protein | 0.52 | 2.39 | 1.70 | NC | U | U | 4 | IQ | ko:K00068 |
| AIL05328.1 |  | ktrA | trkA-N domain protein | 0.22 | 0.12 | 0.15 | NC | NC | NC | 1 | P | ko:K03499 |
| AIL05330.1 |  | XK27_11280 | hypothetical protein DR75_1753 | 0.85 | 0.94 | 0.20 | NC | NC | NC | 1 | S | - |
| AIL05333.1 |  | copA | copper-translocating P-type ATPase | 0.56 | 0.10 | 0.01 | NC | NC | NC | 1 | P | ko:K17686 |
| AIL05335.1 |  | - | hypothetical protein DR75_379 | -0.24 | -0.08 | -0.25 | NC | NC | NC | 1 | - | - |
| AIL05337.1 |  | yacL | PIN domain protein | 0.20 | -0.26 | -0.20 | NC | NC | NC | 1 | S | - |
| AIL05338.1 |  | mscL | large conductance mechanosensitive channel protein | 0.49 | -0.05 | -0.10 | NC | NC | NC | 1 | M | ko:K03282 |
| AIL05340.1 |  | pepS | aminopeptidase pepS | 0.23 | 0.18 | -0.03 | NC | NC | NC | 1 | E | ko:K19689 |
| AIL05342.1 |  | rpoB | DNA-directed RNA polymerase, beta subunit | -0.08 | -0.05 | -0.08 | NC | NC | NC | 1 | K | ko:K03043 |
| AIL05344.1 |  | ydcN1 | helix-turn-helix family protein | -0.14 | -0.11 | -0.03 | NC | NC | NC | 1 | K | - |
| AIL05345.1 |  | - | hypothetical protein DR75_491 | -0.34 | -0.30 | -0.40 | NC | NC | NC | 1 | - | - |
| AIL05346.1 |  | manX | PTS system, mannose/fructose/sorbose, IIB component family protein | -1.33 | -0.86 | -0.92 | D | NC | NC | 6 | G | ko:K02769 |
| AIL05349.1 |  | - | hypothetical protein DR75_1512 | -0.40 | -0.92 | 0.64 | NC | NC | NC | 1 | S | - |
| AIL05350.1 |  | serS | serine--tRNA ligase | 0.05 | 0.07 | 0.02 | NC | NC | NC | 1 | J | ko:K01875 |
| AIL05351.1 |  | murC | UDP-N-acetylmuramate--alanine ligase | 0.25 | -0.33 | -0.12 | NC | NC | NC | 1 | M | ko:K01924 |
| AIL05352.1 |  | yfdH | glycosyl transferase 2 family protein | -0.44 | -0.06 | 0.01 | NC | NC | NC | 1 | M | ko:K12999 |
| AIL05353.1 |  | tgt | queuine tRNA-ribosyltransferase | -0.42 | -0.20 | -0.25 | NC | NC | NC | 1 | F | ko:K00773 |
| AIL05354.1 |  | cps2D | 3-beta hydroxysteroid dehydrogenase/isomerase family protein | -0.85 | -0.31 | -0.03 | NC | NC | NC | 1 | GM | ko:K01784 |
| AIL05355.1 |  | rpe | ribulose-phosphate 3-epimerase | -0.28 | -0.20 | -0.20 | NC | NC | NC | 1 | G | ko:K01783 |
| AIL05356.1 |  | rfbB | dTDP-glucose 4,6-dehydratase | -0.13 | -0.16 | -0.26 | NC | NC | NC | 1 | M | ko:K01710 |
| AIL05357.1 |  | alaS | alanine--tRNA ligase | -0.23 | 0.22 | -0.03 | NC | NC | NC | 1 | J | ko:K01872 |
| AIL05359.1 |  | ybeY | putative rRNA maturation factor YbeY | -0.09 | 0.09 | -0.18 | NC | NC | NC | 1 | S | ko:K07042 |
| AIL05361.1 |  | dus | dihydrouridine synthase family protein | 0.67 | 0.09 | 0.19 | NC | NC | NC | 1 | J | - |
| AIL05362.1 |  | lytR | transcriptional regulator lytR | -0.43 | -0.18 | -0.21 | NC | NC | NC | 1 | K | - |
| AIL05363.1 |  | yufQ | branched-chain amino acid transport system / permease component family protein | 0.19 | 0.42 | -0.11 | NC | NC | NC | 1 | U | ko:K02057 |
| AIL05364.1 |  | aldR | reactive intermediate/imine deaminase family protein | 0.56 | -0.33 | -0.35 | NC | NC | NC | 1 | J | ko:K09022 |
| AIL05365.1 |  | - | hypothetical protein DR75_391 | 0.34 | 0.23 | 0.08 | NC | NC | NC | 1 | - | - |
| AIL05366.1 |  | deoB | phosphopentomutase | 0.79 | 0.66 | 0.34 | NC | NC | NC | 1 | G | ko:K01839 |
| AIL05367.1 |  | - | glycosyl transferase 2 family protein | -0.37 | -0.33 | -0.04 | NC | NC | NC | 1 | S | - |
| AIL05370.1 |  | prkC | phosphotransferase enzyme family protein | -0.24 | -0.23 | -0.12 | NC | NC | NC | 1 | KLT | ko:K08884 |
| AIL05371.1 |  | adcA | periplasmic solute binding family protein | 0.17 | -0.17 | -0.03 | NC | NC | NC | 1 | P | ko:K09815 |
| AIL05372.1 |  | add | adenosine deaminase | 0.39 | 0.59 | 0.94 | NC | NC | NC | 1 | F | ko:K01488 |
| AIL05373.1 |  | ywjA | ABC transporter family protein | 0.51 | 0.38 | 0.16 | NC | NC | NC | 1 | V | ko:K06147 |
| AIL05374.1 |  | rpmF | ribosomal protein L32 | -0.52 | -1.40 | -1.02 | NC | D | D | 22 | J | ko:K02911 |
| AIL05375.1 |  | - | ftsX-like permease family protein | 0.13 | -0.15 | -0.19 | NC | NC | NC | 1 | V | ko:K02004 |
| AIL05376.1 |  | ysdB | ABC transporter family protein | -0.56 | -0.41 | 0.09 | NC | NC | NC | 1 | S | ko:K01990 |
| AIL05377.1 |  | nusA | transcription termination factor NusA | -0.30 | -0.03 | -0.13 | NC | NC | NC | 1 | K | ko:K02600 |
| AIL05378.1 |  | hisS | histidine--tRNA ligase | -0.43 | -0.22 | -0.08 | NC | NC | NC | 1 | J | ko:K01892 |
| AIL05380.1 |  | hslV | ATP-dependent protease HslVU, peptidase subunit | -0.39 | 0.06 | 0.10 | NC | NC | NC | 1 | O | ko:K01419 |
| AIL05382.1 |  | atpD | ATP synthase F1, beta subunit | -0.25 | -0.11 | -0.13 | NC | NC | NC | 1 | C | ko:K02112 |
| AIL05384.1 |  | dinB | hypothetical protein DR75_1449 | 2.84 | 1.90 | 1.04 | U | U | U | 2 | L | ko:K02346 |
| AIL05387.1 |  | rpsT | ribosomal protein S20 | -0.17 | -0.64 | 0.22 | NC | NC | NC | 1 | J | ko:K02968 |
| AIL05388.1 |  | menH | 2-succinyl-6-hydroxy-2,4-cyclohexadiene-1-carboxylate synthase | 1.00 | -0.64 | -0.62 | NC | NC | NC | 1 | I | ko:K08680 |
| AIL05389.1 |  | - | xylose isomerase-like TIM barrel family protein | 0.48 | -0.40 | -0.03 | NC | NC | NC | 1 | G | - |
| AIL05390.1 |  | rnr | ribonuclease R | -0.40 | -0.14 | -0.15 | NC | NC | NC | 1 | J | ko:K12573 |
| AIL05391.1 |  | - | HAD hydrolase, IIB family protein | -0.34 | 0.86 | 0.46 | NC | NC | NC | 1 | S | - |
| AIL05392.1 |  | sun | ribosomal RNA small subunit methyltransferase B | -0.38 | -0.20 | -0.16 | NC | NC | NC | 1 | J | ko:K03500 |
| AIL05393.1 |  | rpmC | ribosomal protein L29 | -0.35 | -0.22 | -0.38 | NC | NC | NC | 1 | J | ko:K02904 |
| AIL05395.1 |  | - | hypothetical protein DR75_1656 | 1.28 | -0.55 | -0.19 | U | NC | NC | 11 | - | - |
| AIL05398.1 |  | argF | ornithine carbamoyltransferase | 0.79 | 0.57 | 0.28 | NC | NC | NC | 1 | E | ko:K00611 |
| AIL05399.1 |  | ynzC | hypothetical protein DR75_583 | -0.21 | -0.29 | -0.41 | NC | NC | NC | 1 | S | - |
| AIL05400.1 |  | - | hypothetical protein DR75_910 | -0.79 | -2.22 | -1.82 | NC | D | D | 22 | - | - |
| AIL05401.1 |  | rliB | periplasmic binding s and sugar binding domain of LacI family protein | -0.03 | -0.04 | 0.55 | NC | NC | NC | 1 | K | - |
| AIL05402.1 |  | mglC | branched-chain amino acid transport system / permease component family protein | 0.18 | 0.45 | 0.19 | NC | NC | NC | 1 | U | ko:K02057 |
| AIL05403.1 |  | parC | DNA topoisomerase IV, A subunit | -0.66 | -0.21 | -0.16 | NC | NC | NC | 1 | L | ko:K02621 |
| AIL05405.1 |  | lon | PDZ domain protein | 0.67 | 0.29 | 0.52 | NC | NC | NC | 1 | T | ko:K07177 |
| AIL05406.1 |  | carA | carbamoyl-phosphate synthase, small subunit | -0.93 | 0.51 | 0.34 | NC | NC | NC | 1 | F | ko:K01956 |
| AIL05408.1 |  | suhB | inositol monophosphatase family protein | 0.28 | 0.04 | -0.02 | NC | NC | NC | 1 | G | ko:K01092 |
| AIL05409.1 |  | mccB | cystathionine beta-lyase | -1.57 | -0.09 | -0.13 | D | NC | NC | 6 | E | ko:K01760 |
| AIL05410.1 |  | dagK | lipid kinase, YegS//BmrU family protein | -0.23 | 0.04 | -0.04 | NC | NC | NC | 1 | I | ko:K07029 |
| AIL05411.1 |  | rpmB | ribosomal protein L28 | -0.32 | -0.33 | -0.25 | NC | NC | NC | 1 | J | ko:K02902 |
| AIL05412.1 |  | cdsA | cytidylyltransferase family protein | -0.31 | -0.22 | 0.48 | NC | NC | NC | 1 | S | ko:K00981 |
| AIL05414.1 |  | - | 5-bromo-4-chloroindolyl phosphate hydrolysis family protein | -1.00 | -1.67 | -0.19 | NC | D | NC | 23 | S | - |
| AIL05416.1 |  | msbA_1 | ABC transporter family protein | 0.01 | 0.17 | -0.06 | NC | NC | NC | 1 | V | ko:K18887 |
| AIL05418.1 |  | trxA | thioredoxin | -0.18 | 0.66 | 0.40 | NC | NC | NC | 1 | O | ko:K03671 |
| AIL05419.1 |  | hup | DNA-binding protein HU | -0.17 | -0.16 | -0.58 | NC | NC | NC | 1 | L | ko:K03530 |
| AIL05420.1 |  | - | hypothetical protein DR75_991 | -0.29 | -0.29 | -0.11 | NC | NC | NC | 1 | S | ko:K16924 |
| AIL05423.1 |  | rbgA | ribosome biogenesis GTP-binding protein YlqF | -0.50 | -0.42 | -0.37 | NC | NC | NC | 1 | S | ko:K14540 |
| AIL05425.1 |  | yebE | hypothetical protein DR75_612 | 0.41 | 0.01 | -0.23 | NC | NC | NC | 1 | S | - |
| AIL05426.1 |  | - | SPFH domain / Band 7 family protein | 0.39 | 0.00 | 0.00 | NC | NC | NC | 1 | O | - |
| AIL05427.1 |  | - | phosphate binding family protein | -0.58 | -0.33 | -0.40 | NC | NC | NC | 1 | P | ko:K02040 |
| AIL05432.1 |  | - | hypothetical protein DR75_2796 | -0.17 | 0.16 | -0.42 | NC | NC | NC | 1 | - | - |
| AIL05435.1 |  | pheT | phenylalanine--tRNA ligase, beta subunit | -0.35 | 0.11 | 0.15 | NC | NC | NC | 1 | J | ko:K01890 |
| AIL05436.1 |  | mgsA | methylglyoxal synthase | 0.68 | 0.20 | 0.14 | NC | NC | NC | 1 | G | ko:K01734 |
| AIL05438.1 |  | - | hypothetical protein DR75_1845 | 0.55 | 1.03 | 1.05 | NC | U | U | 4 | - | - |
| AIL05439.1 |  | pyrD | dihydroorotate dehydrogenase B, catalytic subunit | 0.58 | 0.54 | 0.28 | NC | NC | NC | 1 | F | ko:K00226 |
| AIL05440.1 |  | clpC | istB-like ATP binding family protein | -0.42 | -0.14 | -0.41 | NC | NC | NC | 1 | O | ko:K03696 |
| AIL05441.1 |  | efp | translation elongation factor P | -0.02 | -0.02 | 0.03 | NC | NC | NC | 1 | J | ko:K02356 |
| AIL05442.1 |  | natB | ABC-2 transporter family protein | 0.28 | -0.36 | 0.06 | NC | NC | NC | 1 | CP | ko:K01992 |
| AIL05443.1 |  | - | endo-alpha-N-acetylgalactosaminidase family protein | -1.17 | -1.04 | -0.59 | D | D | NC | 20 | G | ko:K17624 |
| AIL05446.1 |  | malP | citrate carrier, CCS family protein | -0.35 | 3.23 | 2.46 | NC | U | U | 4 | U | ko:K11616 |
| AIL05447.1 |  | - | hypothetical protein DR75_2591 | 0.94 | 0.94 | 0.17 | NC | NC | NC | 1 | S | - |
| AIL05450.1 |  | def1 | peptide deformylase | -0.23 | 0.41 | 0.21 | NC | NC | NC | 1 | J | ko:K01462 |
| AIL05452.1 |  | clpP | ATP-dependent Clp endopeptidase, proteolytic subunit ClpP | 0.55 | 0.04 | -0.28 | NC | NC | NC | 1 | O | ko:K01358 |
| AIL05453.1 |  | - | hypothetical protein DR75_2046 | 0.67 | -0.05 | 0.32 | NC | NC | NC | 1 | K | - |
| AIL05454.1 |  | yloC | hypothetical protein DR75_1846 | -0.36 | -0.63 | -0.55 | NC | NC | NC | 1 | S | - |
| AIL05456.1 |  | ptsG | PTS system, glucose-like IIB component domain protein | -1.17 | -1.26 | -0.16 | D | D | NC | 20 | G | ko:K02777 |
| AIL05457.1 |  | rpmE2 | ribosomal protein L31 | -0.70 | 0.45 | 0.06 | NC | NC | NC | 1 | J | ko:K02909 |
| AIL05459.1 |  | rpsS | ribosomal protein S19 | -0.38 | 0.00 | -0.53 | NC | NC | NC | 1 | J | ko:K02965 |
| AIL05460.1 |  | - | HAMP domain protein | 1.00 | 2.70 | 1.29 | NC | U | U | 4 | T | ko:K07718 |
| AIL05461.1 |  | rsmH | 16S rRNA (cytosine(1402)-N(4))-methyltransferase | -0.62 | -0.46 | -0.54 | NC | NC | NC | 1 | J | ko:K03438 |
| AIL05463.1 |  | - | thioredoxin family protein | 0.80 | -0.38 | -0.64 | NC | NC | NC | 1 | O | - |
| AIL05465.1 |  | oppA | bacterial extracellular solute-binding s, 5 Middle family protein | -0.13 | -0.06 | -0.08 | NC | NC | NC | 1 | E | ko:K02035 |
| AIL05467.1 |  | - | bacterial regulatory, arsR family protein | 0.33 | -0.19 | 0.06 | NC | NC | NC | 1 | K | - |
| AIL05468.1 |  | trmB | tRNA (guanine-N(7)-)-methyltransferase | -0.12 | 0.08 | -0.36 | NC | NC | NC | 1 | J | ko:K03439 |
| AIL05469.1 |  | - | glucosyl transferase GtrII family protein | -0.76 | -0.51 | -0.66 | NC | NC | NC | 1 | S | - |
| AIL05472.1 |  | htrA | PDZ domain protein | 0.90 | 0.17 | 0.17 | NC | NC | NC | 1 | O | ko:K04771 |
| AIL05474.1 |  | - | bacterial regulatory s, tetR family protein | -0.45 | -0.59 | -0.06 | NC | NC | NC | 1 | K | - |
| AIL05475.1 |  | prfC | peptide chain release factor 3 | -0.50 | -0.39 | -0.34 | NC | NC | NC | 1 | J | ko:K02837 |
| AIL05476.1 |  | ybjQ | heavy-metal-binding family protein | 0.34 | 0.38 | 0.49 | NC | NC | NC | 1 | S | - |
| AIL05477.1 |  | pbp2A | penicillin-binding, 1A family protein | 0.19 | -0.15 | -0.07 | NC | NC | NC | 1 | M | ko:K12555 |
| AIL05478.1 |  | rplB | ribosomal protein L2 | -0.15 | -0.48 | -0.39 | NC | NC | NC | 1 | J | ko:K02886 |
| AIL05481.1 |  | yitT | hypothetical protein DR75_237 | -0.13 | -0.40 | -0.03 | NC | NC | NC | 1 | S | - |
| AIL05483.1 |  | arcC | carbamate kinase | 0.80 | 0.81 | 0.56 | NC | NC | NC | 1 | E | ko:K00926 |
| AIL05485.1 |  | dltA | D-alanine--poly(phosphoribitol) ligase, subunit 1 | -0.09 | -0.04 | 0.05 | NC | NC | NC | 1 | Q | ko:K03367 |
| AIL05486.1 |  | ymdB | ymdB-like family protein | -0.08 | 0.02 | -0.08 | NC | NC | NC | 1 | S | ko:K02029 |
| AIL05487.1 |  | udk | uridine kinase | -1.39 | -0.34 | -0.34 | D | NC | NC | 6 | F | ko:K00876 |
| AIL05488.1 |  | pth | peptidyl-tRNA hydrolase | 0.69 | 0.03 | 0.02 | NC | NC | NC | 1 | J | ko:K01056 |
| AIL05489.1 |  | XK27_09605 | ABC transporter family protein | -0.11 | 0.52 | 0.04 | NC | NC | NC | 1 | V | ko:K18892 |
| AIL05490.1 |  | - | DNA gyrase B family protein | -0.01 | 0.11 | 0.14 | NC | NC | NC | 1 | L | - |
| AIL05492.1 |  | - | beta-lactamase family protein | 0.12 | -0.22 | -0.08 | NC | NC | NC | 1 | V | - |
| AIL05493.1 |  | iunH3 | inosine-uridine preferring nucleoside hydrolase family protein | -0.64 | -0.41 | 0.40 | NC | NC | NC | 1 | F | ko:K01239 |
| AIL05496.1 |  | ezrA | septation ring formation regulator, EzrA family protein | -0.42 | -0.16 | -0.15 | NC | NC | NC | 1 | D | ko:K06286 |
| AIL05501.1 |  | fabK | nitronate monooxygenase family protein | -1.33 | -0.13 | -0.40 | D | NC | NC | 6 | S | ko:K02371 |
| AIL05502.1 |  | rph | ribonuclease PH | -0.02 | -0.10 | -0.18 | NC | NC | NC | 1 | F | ko:K00989 |
| AIL05505.1 |  | ypjD | hypothetical protein DR75_559 | -0.03 | -0.52 | -0.45 | NC | NC | NC | 1 | S | - |
| AIL05506.1 |  | degV | EDD, DegV family domain protein | -0.14 | 0.03 | 0.04 | NC | NC | NC | 1 | K | - |
| AIL05511.1 |  | pimB | glycosyl transferases group 1 family protein | 0.20 | 0.12 | -0.36 | NC | NC | NC | 1 | M | ko:K19002 |
| AIL05512.1 |  | dak | deoxynucleoside kinase family protein | 0.53 | -0.03 | -0.19 | NC | NC | NC | 1 | F | - |
| AIL05513.1 |  | rpmG | ribosomal protein L33 | -0.17 | 0.29 | 0.10 | NC | NC | NC | 1 | J | ko:K02913 |
| AIL05515.1 |  | bfmBC | dihydrolipoyl dehydrogenase | -1.09 | 1.51 | 1.38 | D | U | U | 21 | C | ko:K00382 |
| AIL05517.1 |  | glmM | phosphoglucosamine mutase | 0.11 | -0.01 | -0.04 | NC | NC | NC | 1 | G | ko:K03431 |
| AIL05518.1 |  | - | hypothetical protein DR75_1344 | 0.57 | 0.31 | 0.31 | NC | NC | NC | 1 | S | ko:K03595 |
| AIL05519.1 |  | tatD | hydrolase, TatD family protein | 0.18 | 0.16 | -0.06 | NC | NC | NC | 1 | L | ko:K03424 |
| AIL05520.1 |  | alr | alanine racemase | 0.15 | -0.07 | 0.03 | NC | NC | NC | 1 | M | ko:K01775 |
| AIL05525.1 |  | - | muramidase-2 | -0.63 | -1.48 | -1.29 | NC | D | D | 22 | NU | - |
| AIL05527.1 |  | rpsE | ribosomal protein S5 | -0.05 | 0.07 | -0.09 | NC | NC | NC | 1 | J | ko:K02988 |
| AIL05528.1 |  | - | PTS system sorbose-specific iic component family protein | -1.04 | 0.61 | -0.27 | D | NC | NC | 6 | G | ko:K19508 |
| AIL05529.1 |  | pcrA | ATP-dependent DNA helicase PcrA | -0.09 | -0.17 | -0.14 | NC | NC | NC | 1 | L | ko:K03657 |
| AIL05530.1 |  | - | hypothetical protein DR75_1059 | 0.79 | 0.45 | 0.27 | NC | NC | NC | 1 | S | - |
| AIL05531.1 |  | mipB | transaldolase family protein | 0.31 | 2.74 | 2.26 | NC | U | U | 4 | H | ko:K00616 |
| AIL05532.1 |  | - | wxL domain surface cell wall-binding family protein | -0.67 | -0.58 | -0.54 | NC | NC | NC | 1 | S | - |
| AIL05534.1 |  | rbsD | rbsD / FucU transport family protein | 0.06 | -0.14 | -0.17 | NC | NC | NC | 1 | G | ko:K06726 |
| AIL05535.1 |  | - | hypothetical protein DR75_316 | 0.48 | 0.20 | 0.13 | NC | NC | NC | 1 | S | ko:K01989 |
| AIL05536.1 |  | binR | hypothetical protein DR75_2488 | 0.09 | 0.65 | 0.42 | NC | NC | NC | 1 | L | - |
| AIL05539.1 |  | thrB | homoserine kinase | 0.40 | 0.05 | -0.08 | NC | NC | NC | 1 | F | ko:K00872 |
| AIL05540.1 |  | - | universal stress family protein | 0.67 | 0.51 | 0.46 | NC | NC | NC | 1 | T | - |
| AIL05542.1 |  | manN | PTS system, mannose/fructose/sorbose, IID component family protein | -0.42 | 0.30 | 0.18 | NC | NC | NC | 1 | G | ko:K02796 |
| AIL05544.1 |  | maf | septum formation protein Maf | 0.00 | -1.14 | -0.54 | NC | D | NC | 23 | D | ko:K06287 |
| AIL05545.1 |  | ycsE | HAD hydrolase, IIB family protein | 0.34 | 0.11 | 0.15 | NC | NC | NC | 1 | S | - |
| AIL05546.1 |  | ftsH | ATP-dependent metallopeptidase HflB family protein | 0.18 | -0.18 | -0.12 | NC | NC | NC | 1 | O | ko:K03798 |
| AIL05547.1 |  | pfkA | 6-phosphofructokinase | 0.10 | -0.01 | -0.07 | NC | NC | NC | 1 | F | ko:K00850 |
| AIL05549.1 |  | - | arginine repressor | -0.10 | 0.04 | -0.34 | NC | NC | NC | 1 | K | ko:K03402 |
| AIL05550.1 |  | ctpE | HAD ATPase, P-type, IC family protein | -0.58 | 0.03 | 0.21 | NC | NC | NC | 1 | P | ko:K12952 |
| AIL05551.1 |  | lutA | lactate utilization protein A | -2.08 | -1.01 | -0.61 | D | D | NC | 20 | C | ko:K18928 |
| AIL05552.1 |  | gidA | tRNA uridine 5-carboxymethylaminomethyl modification enzyme GidA | 1.14 | 0.09 | 0.11 | U | NC | NC | 11 | D | ko:K03495 |
| AIL05554.1 |  | - | hypothetical protein DR75_488 | 0.25 | 0.71 | 0.64 | NC | NC | NC | 1 | S | ko:K07504 |
| AIL05559.1 |  | ftsE | cell division ATP-binding protein FtsE | 0.22 | 0.07 | -0.08 | NC | NC | NC | 1 | D | ko:K09812 |
| AIL05560.1 |  | - | bacterial regulatory s, tetR family protein | -0.26 | -0.80 | 0.11 | NC | NC | NC | 1 | K | - |
| AIL05561.1 |  | pucD | [2Fe-2S] binding domain protein | 1.30 | 1.66 | 1.11 | U | U | U | 2 | C | ko:K00087 |
| AIL05562.1 |  | ybbR | ybbR-like family protein | 0.03 | -0.12 | -0.23 | NC | NC | NC | 1 | S | - |
| AIL05566.1 |  | glyS | glycine--tRNA ligase, beta subunit | -0.46 | -0.24 | -0.11 | NC | NC | NC | 1 | J | ko:K01879 |
| AIL05567.1 |  | yycF | transcriptional regulatory protein yycF | 0.46 | 0.13 | 0.29 | NC | NC | NC | 1 | K | ko:K07668 |
| AIL05568.1 |  | prs2 | ribose-phosphate pyrophosphokinase 2 | -0.35 | -0.12 | 0.00 | NC | NC | NC | 1 | F | ko:K00948 |
| AIL05572.1 |  | queG | epoxyqueuosine reductase | -1.24 | -0.83 | -0.54 | D | NC | NC | 6 | C | ko:K18979 |
| AIL05573.1 |  | prs | ribose-phosphate pyrophosphokinase | -0.58 | -0.14 | -0.25 | NC | NC | NC | 1 | F | ko:K00948 |
| AIL05574.1 |  | pucG | purine catabolism protein PucG | 1.65 | -0.55 | 0.36 | U | NC | NC | 11 | E | ko:K00830 |
| AIL05576.1 |  | atoB | hydroxymethylglutaryl-CoA reductase, degradative | 0.08 | -0.19 | -0.02 | NC | NC | NC | 1 | I | ko:K00054 |
| AIL05577.1 |  | - | dipeptidase family protein | 0.64 | 0.50 | 0.49 | NC | NC | NC | 1 | E | - |
| AIL05578.1 |  | mltG | yceG-like family protein | -0.37 | -0.17 | -0.23 | NC | NC | NC | 1 | S | ko:K07082 |
| AIL05579.1 |  | fur | ferric uptake regulation protein | -0.68 | -0.53 | -0.63 | NC | NC | NC | 1 | P | ko:K03711 |
| AIL05580.1 |  | pcp | pyroglutamyl-peptidase I | -0.23 | -0.24 | -0.33 | NC | NC | NC | 1 | O | ko:K01304 |
| AIL05581.1 |  | glpK | glycerol kinase | -0.12 | 0.26 | 0.04 | NC | NC | NC | 1 | F | ko:K00864 |
| AIL05583.1 |  | yneR | putative yneR | 0.55 | 0.38 | 0.19 | NC | NC | NC | 1 | S | - |
| AIL05585.1 |  | - | hypothetical protein DR75_388 | -0.02 | 0.30 | 0.30 | NC | NC | NC | 1 | S | - |
| AIL05586.1 |  | XK27_02735 | marR family protein | -0.13 | -0.63 | -0.84 | NC | NC | NC | 1 | K | - |
| AIL05587.1 |  | asp23 | response regulator | 0.83 | 0.24 | 0.10 | NC | NC | NC | 1 | S | - |
| AIL05589.1 |  | deoD | purine nucleoside phosphorylase | 0.56 | 0.30 | 0.26 | NC | NC | NC | 1 | F | ko:K03784 |
| AIL05593.1 |  | pdtaR | ANTAR domain protein | -0.37 | -0.54 | -0.28 | NC | NC | NC | 1 | K | ko:K22010 |
| AIL05594.1 |  | yqeH | ribosome biogenesis GTPase YqeH | -0.08 | -0.17 | -0.29 | NC | NC | NC | 1 | S | ko:K06948 |
| AIL05596.1 |  | sodA | superoxide dismutase Mn | 1.60 | 1.13 | 1.25 | U | U | U | 2 | C | ko:K04564 |
| AIL05597.1 |  | - | hypothetical protein DR75_2022 | 1.02 | 0.73 | -0.98 | U | NC | NC | 11 | - | - |
| AIL05598.1 |  | pheS | phenylalanine--tRNA ligase, alpha subunit | -0.51 | -0.11 | -0.18 | NC | NC | NC | 1 | J | ko:K01889 |
| AIL05599.1 |  | fabH | 3-oxoacyl-[acyl-carrier-protein] synthase 3 | -0.55 | 0.11 | 0.08 | NC | NC | NC | 1 | I | ko:K00648 |
| AIL05601.1 |  | nanE | putative N-acetylmannosamine-6-phosphate 2-epimerase | -0.42 | 0.12 | 0.32 | NC | NC | NC | 1 | G | ko:K01788 |
| AIL05604.1 |  | rplI | ribosomal protein L9 | 0.80 | 0.23 | -0.08 | NC | NC | NC | 1 | J | ko:K02939 |
| AIL05605.1 |  | nhaP1 | sodium/hydrogen exchanger family protein | 1.12 | 0.73 | -0.33 | U | NC | NC | 11 | P | ko:K03316 |
| AIL05607.1 |  | - | ROK family protein | -0.09 | 0.97 | 0.63 | NC | NC | NC | 1 | GK | ko:K00847 |
| AIL05608.1 |  | acmB | mannosyl-glycoendo-beta-N-acetylglucosaminidase family protein | -0.58 | -0.71 | -0.85 | NC | NC | NC | 1 | NU | ko:K01227 |
| AIL05611.1 |  | ackA | acetate kinase | -0.39 | 0.14 | 0.13 | NC | NC | NC | 1 | F | ko:K00925 |
| AIL05612.1 |  | yclJ | response regulator | 0.50 | 0.14 | 0.41 | NC | NC | NC | 1 | K | ko:K02483 |
| AIL05613.1 |  | bfmBAA | 2-oxoisovalerate dehydrogenase subunit alpha | -0.19 | 0.27 | 0.48 | NC | NC | NC | 1 | C | ko:K00166 |
| AIL05614.1 |  | panC | pantoate--beta-alanine ligase | 0.13 | -0.20 | -0.81 | NC | NC | NC | 1 | H | ko:K01918 |
| AIL05615.1 |  | - | hypothetical protein DR75_1848 | 0.72 | 1.18 | -0.12 | NC | U | NC | 15 | S | - |
| AIL05617.1 |  | yidA | SIS domain protein | 0.45 | 0.02 | 0.16 | NC | NC | NC | 1 | K | - |
| AIL05618.1 |  | murQ | N-acetylmuramic acid 6-phosphate etherase | -0.13 | 0.31 | 0.51 | NC | NC | NC | 1 | G | ko:K07106 |
| AIL05619.1 |  | mocA | oxidoreductase, NAD-binding Rossmann fold family protein | 0.79 | 0.25 | 0.32 | NC | NC | NC | 1 | S | - |
| AIL05620.1 |  | ysdC | putative aminopeptidase ysdC | 0.17 | 0.14 | 0.19 | NC | NC | NC | 1 | G | - |
| AIL05621.1 |  | whiA | hypothetical protein DR75_2721 | -0.85 | -0.01 | 0.06 | NC | NC | NC | 1 | K | ko:K09762 |
| AIL05622.1 |  | ylbN | hypothetical protein DR75_106 | 0.11 | -0.22 | -0.19 | NC | NC | NC | 1 | S | ko:K07040 |
| AIL05624.1 |  | snf | DEAD/DEAH box helicase family protein | 0.04 | -0.02 | -0.19 | NC | NC | NC | 1 | KL | - |
| AIL05626.1 |  | folB | dihydroneopterin aldolase | 0.26 | -1.36 | -0.39 | NC | D | NC | 23 | H | ko:K01633 |
| AIL05630.1 |  | yozE | hypothetical protein DR75_681 | 0.28 | 0.91 | -0.71 | NC | NC | NC | 1 | S | - |
| AIL05631.1 |  | ybbF | phosphotransferase system, EIIB family protein | -0.22 | -0.10 | -0.13 | NC | NC | NC | 1 | G | ko:K02808 |
| AIL05633.1 |  | - | acetyltransferase family protein | -0.58 | -0.39 | -0.13 | NC | NC | NC | 1 | J | - |
| AIL05637.1 |  | ychF | 50S ribosome-binding GTPase family protein | -0.61 | -0.49 | -0.36 | NC | NC | NC | 1 | J | ko:K06942 |
| AIL05638.1 |  | - | PTS system, lactose/cellobiose IIC component family protein | 1.03 | -0.04 | 0.12 | U | NC | NC | 11 | U | ko:K02761 |
| AIL05643.1 |  | mutS2 | MutS2 family protein | -0.12 | -0.05 | -0.22 | NC | NC | NC | 1 | L | ko:K07456 |
| AIL05644.1 |  | pyrF | orotidine 5'-phosphate decarboxylase | -1.24 | 0.17 | 0.37 | D | NC | NC | 6 | F | ko:K01591 |
| AIL05646.1 |  | yidC | membrane insertase, YidC/Oxa1 family domain protein | -0.83 | -1.00 | -1.21 | NC | NC | D | 19 | U | ko:K03217 |
| AIL05649.1 |  | atpH | ATP synthase F1, delta subunit | -0.56 | -0.68 | -0.73 | NC | NC | NC | 1 | C | ko:K02113 |
| AIL05650.1 |  | - | helix-turn-helix family protein | 0.62 | -0.42 | 0.36 | NC | NC | NC | 1 | K | - |
| AIL05651.1 |  | - | restriction endonuclease family protein | -0.96 | -0.23 | -0.35 | NC | NC | NC | 1 | L | ko:K07448 |
| AIL05653.1 |  | folD | tetrahydrofolate dehydrogenase/cyclohydrolase, catalytic domain protein | -0.01 | -0.06 | -0.23 | NC | NC | NC | 1 | F | ko:K01491 |
| AIL05654.1 |  | - | beta-ketoacyl-acyl-carrier-protein synthase II | -0.66 | 0.00 | -0.19 | NC | NC | NC | 1 | I | ko:K09458 |
| AIL05655.1 |  | pdxK | pyridoxine kinase | 0.28 | 0.20 | 0.24 | NC | NC | NC | 1 | H | ko:K00868 |
| AIL05656.1 |  | ywlC | tRNA threonylcarbamoyl adenosine modification protein, Sua5/YciO/YrdC/YwlC family | -0.17 | 0.16 | -0.27 | NC | NC | NC | 1 | J | ko:K07566 |
| AIL05657.1 |  | ppaC | putative manganese-dependent inorganic pyrophosphatase | -0.16 | 0.09 | -0.29 | NC | NC | NC | 1 | C | ko:K15986 |
| AIL05660.1 |  | - | hypothetical protein DR75_1649 | 1.08 | -1.79 | -0.62 | U | D | NC | 12 | S | - |
| AIL05661.1 |  | - | type I restriction modification DNA specificity domain protein | -0.54 | -0.24 | -0.39 | NC | NC | NC | 1 | V | ko:K01154 |
| AIL05663.1 |  | XK27_09445 | hypothetical protein DR75_2664 | 0.63 | 0.36 | 0.40 | NC | NC | NC | 1 | S | - |
| AIL05664.1 |  | ybaK | aminoacyl-tRNA editing domain protein | 0.56 | 0.24 | 0.00 | NC | NC | NC | 1 | J | ko:K03976 |
| AIL05665.1 |  | - | hypothetical protein DR75_1889 | -0.71 | -1.10 | -0.34 | NC | D | NC | 23 | M | - |
| AIL05666.1 |  | pbp2b | penicillin binding transpeptidase domain protein | -0.33 | -0.54 | -0.50 | NC | NC | NC | 1 | M | ko:K00687 |
| AIL05669.1 |  | ytbE | hypothetical protein DR75_2579 | 0.46 | 0.22 | 0.07 | NC | NC | NC | 1 | S | - |
| AIL05670.1 |  | - | hypothetical protein DR75_320 | 1.81 | 0.29 | 1.09 | U | NC | U | 14 | S | - |
| AIL05671.1 |  | xseA | exodeoxyribonuclease VII, large subunit | -0.97 | 0.16 | -0.36 | NC | NC | NC | 1 | L | ko:K03601 |
| AIL05674.1 |  | pbg9 | aryl-phospho-beta-D-glucosidase BglH | 0.54 | 2.67 | 2.12 | NC | U | U | 4 | G | ko:K01223 |
| AIL05677.1 |  | buk | butyrate kinase | 0.19 | 1.11 | 0.97 | NC | U | NC | 15 | F | ko:K00929 |
| AIL05678.1 |  | yabE | 3D domain protein | -1.47 | 0.09 | -0.51 | D | NC | NC | 6 | S | ko:K02424 |
| AIL05679.1 |  | - | hypothetical protein DR75_1257 | 2.61 | 2.80 | 2.36 | U | U | U | 2 | - | - |
| AIL05680.1 |  | yqhL | rhodanese-like domain protein | -0.37 | -0.62 | -0.54 | NC | NC | NC | 1 | P | - |
| AIL05684.1 |  | nrdG | anaerobic ribonucleoside-triphosphate reductase activating protein | 0.59 | -0.58 | -0.61 | NC | NC | NC | 1 | O | ko:K04068 |
| AIL05685.1 |  | map | methionine aminopeptidase, type I | 0.28 | 0.27 | 0.42 | NC | NC | NC | 1 | E | ko:K01265 |
| AIL05687.1 |  | - | alpha amylase, catalytic domain protein | 0.91 | 1.34 | 0.46 | NC | U | NC | 15 | G | ko:K01187 |
| AIL05688.1 |  | - | ROK family protein | -0.08 | 0.51 | 0.24 | NC | NC | NC | 1 | GK | - |
| AIL05691.1 |  | YSH1 | hypothetical protein DR75_1599 | 0.20 | 0.51 | 0.37 | NC | NC | NC | 1 | S | ko:K12574 |
| AIL05692.1 |  | yhaM | OB-fold nucleic acid binding domain protein | 0.57 | 0.07 | 0.01 | NC | NC | NC | 1 | S | ko:K03698 |
| AIL05693.1 |  | - | hypothetical protein DR75_1813 | -1.46 | -1.26 | -1.32 | D | D | D | 3 | S | ko:K09705 |
| AIL05694.1 |  | yitS | EDD, DegV family domain protein | 0.87 | 0.12 | 0.22 | NC | NC | NC | 1 | S | - |
| AIL05702.1 |  | - | glucosyl transferase GtrII family protein | -0.59 | 0.13 | 0.11 | NC | NC | NC | 1 | S | - |
| AIL05705.1 |  | - | hypothetical protein DR75_298 | -0.15 | 0.00 | -0.12 | NC | NC | NC | 1 | G | ko:K17318 |
| AIL05707.1 |  | - | core-2/I-Branching enzyme family protein | 1.79 | 0.20 | -0.10 | U | NC | NC | 11 | S | - |
| AIL05710.1 |  | - | hypothetical protein DR75_663 | 0.29 | 0.26 | 3.30 | NC | NC | U | 5 | S | - |
| AIL05713.1 |  | fabG | NAD(P)H binding domain of trans-2-enoyl-CoA reductase family protein | -0.46 | -0.88 | -0.44 | NC | NC | NC | 1 | IQ | ko:K00059 |
| AIL05714.1 |  | folA | dihydrofolate reductase | -0.09 | 0.15 | 0.06 | NC | NC | NC | 1 | H | ko:K00287 |
| AIL05715.1 |  | proA | glutamate-5-semialdehyde dehydrogenase | 0.42 | 0.05 | 0.15 | NC | NC | NC | 1 | E | ko:K00147 |
| AIL05716.1 |  | potA | polyamine ABC transporter, ATP-binding family protein | -0.66 | -0.48 | -0.59 | NC | NC | NC | 1 | E | ko:K02010 |
| AIL05718.1 |  | infA | translation initiation factor IF-1 | -0.28 | 0.35 | 0.27 | NC | NC | NC | 1 | J | ko:K02518 |
| AIL05719.1 |  | galE | UDP-glucose 4-epimerase GalE | 1.75 | 1.56 | 1.36 | U | U | U | 2 | M | ko:K01784 |
| AIL05722.1 |  | yurR | FAD dependent oxidoreductase family protein | -0.09 | -0.13 | -0.01 | NC | NC | NC | 1 | E | ko:K00285 |
| AIL05724.1 |  | serA | D-isomer specific 2-hydroxyacid dehydrogenase, NAD binding domain protein | 0.60 | 0.15 | 0.30 | NC | NC | NC | 1 | EH | ko:K00058 |
| AIL05731.1 |  | dnaN | DNA polymerase III, beta subunit | 0.27 | 0.07 | 0.09 | NC | NC | NC | 1 | L | ko:K02338 |
| AIL05732.1 |  | tyrA | ACT domain protein | -1.49 | -1.01 | -1.02 | D | D | D | 3 | E | ko:K04517 |
| AIL05733.1 |  | hslO | 33 kDa chaperonin | 0.00 | -0.23 | -0.31 | NC | NC | NC | 1 | O | ko:K04083 |
| AIL05734.1 |  | pip | hypothetical protein DR75_2797 | 0.78 | 0.08 | -0.13 | NC | NC | NC | 1 | S | ko:K01421 |
| AIL05735.1 |  | - | HAD hydrolase, IIB family protein | -0.23 | -0.05 | 0.05 | NC | NC | NC | 1 | S | - |
| AIL05736.1 |  | mraZ | mraZ family protein | -0.24 | -0.12 | -0.16 | NC | NC | NC | 1 | K | ko:K03925 |
| AIL05738.1 |  | mgtA | magnesium-translocating P-type ATPase | 0.20 | 0.18 | -0.21 | NC | NC | NC | 1 | P | ko:K01531 |
| AIL05739.1 |  | pdhC | dihydrolipoyllysine-residue acetyltransferase component of pyruvate dehydrogenase complex | 0.22 | 0.42 | 0.16 | NC | NC | NC | 1 | C | ko:K00627 |
| AIL05743.1 |  | - | PTS system sorbose subIIB component family protein | 0.75 | 0.93 | 1.35 | NC | NC | U | 5 | G | ko:K02794 |
| AIL05745.1 |  | holB | DNA polymerase III, delta' subunit | -0.22 | 0.26 | 0.52 | NC | NC | NC | 1 | L | ko:K02341 |
| AIL05750.1 |  | sepF | cell division protein sepF | -0.62 | -0.35 | -0.54 | NC | NC | NC | 1 | D | ko:K09772 |
| AIL05753.1 |  | - | hypothetical protein DR75_1559 | 0.27 | -0.30 | -0.27 | NC | NC | NC | 1 | - | - |
| AIL05754.1 |  | - | hypothetical protein DR75_1491 | 0.37 | 0.01 | 0.05 | NC | NC | NC | 1 | S | - |
| AIL05758.1 |  | - | peptidase T | -0.80 | -0.44 | -0.89 | NC | NC | NC | 1 | E | ko:K01258 |
| AIL05759.1 |  | phoU | phosphate transport system regulatory protein PhoU | 0.08 | -0.24 | -0.44 | NC | NC | NC | 1 | P | ko:K02039 |
| AIL05760.1 |  | - | hypothetical protein DR75_1847 | -0.58 | -0.41 | -0.70 | NC | NC | NC | 1 | S | - |
| AIL05761.1 |  | - | hypothetical protein DR75_882 | -0.45 | 0.34 | 0.72 | NC | NC | NC | 1 | - | - |
| AIL05763.1 |  | rpsB | ribosomal protein S2 | -0.54 | -0.16 | -0.43 | NC | NC | NC | 1 | J | ko:K02967 |
| AIL05764.1 |  | - | hypothetical protein DR75_1533 | -0.49 | -1.01 | -0.37 | NC | D | NC | 23 | L | - |
| AIL05765.1 |  | - | hypothetical protein DR75_986 | 0.61 | 1.47 | 0.13 | NC | U | NC | 15 | - | - |
| AIL05770.1 |  | rplL | ribosomal protein L7/L12 | -0.35 | -0.56 | -0.80 | NC | NC | NC | 1 | J | ko:K02935 |
| AIL05771.1 |  | yyzM | hypothetical protein DR75_2005 | -1.21 | -0.80 | -0.11 | D | NC | NC | 6 | S | - |
| AIL05773.1 |  | rluB | pseudouridine synthase family protein | 0.03 | 0.42 | -0.17 | NC | NC | NC | 1 | J | ko:K06178 |
| AIL05775.1 |  | - | adenylate and Guanylate cyclase catalytic domain protein | 0.47 | 0.33 | 0.60 | NC | NC | NC | 1 | T | ko:K01768 |
| AIL05777.1 |  | ntpK | V-type sodium ATPase subunit K | -0.16 | 0.23 | 0.00 | NC | NC | NC | 1 | U | ko:K02124 |
| AIL05779.1 |  | - | zinc-binding dehydrogenase family protein | -0.55 | -0.01 | -0.06 | NC | NC | NC | 1 | C | ko:K00001 |
| AIL05781.1 |  | - | SIS domain protein | -0.32 | 0.08 | -0.37 | NC | NC | NC | 1 | M | ko:K19504 |
| AIL05782.1 |  | rsmC | tRNA (Uracil-5-)-methyltransferase family protein | 0.10 | -0.13 | -0.02 | NC | NC | NC | 1 | J | ko:K00564 |
| AIL05783.1 |  | XK27_09615 | NADPH-dependent FMN reductase family protein | 0.70 | -0.58 | -0.38 | NC | NC | NC | 1 | S | ko:K19784 |
| AIL05784.1 |  | ytcD | hxlR-like helix-turn-helix family protein | -0.52 | -0.57 | -0.77 | NC | NC | NC | 1 | K | - |
| AIL05785.1 |  | dhaL | dihydroxyacetone kinase, L subunit | 0.64 | 1.04 | 1.00 | NC | U | U | 4 | G | ko:K05879 |
| AIL05786.1 |  | metI | binding--dependent transport system inner membrane component family protein | 0.57 | 0.76 | 0.47 | NC | NC | NC | 1 | U | ko:K02069 |
| AIL05787.1 |  | chpA | pemK-like family protein | 0.22 | -0.15 | -0.14 | NC | NC | NC | 1 | T | ko:K07171 |
| AIL05789.1 |  | ylbG | hypothetical protein DR75_1200 | -0.19 | 0.11 | -0.03 | NC | NC | NC | 1 | S | - |
| AIL05791.1 |  | yaaK | DNA-binding protein, YbaB/EbfC family | 0.21 | 0.19 | -0.09 | NC | NC | NC | 1 | S | ko:K09747 |
| AIL05800.1 |  | ycfI | ABC transporter family protein | -0.09 | -0.07 | -0.35 | NC | NC | NC | 1 | V | ko:K06147 |
| AIL05801.1 |  | rimI | acetyltransferase family protein | -0.97 | -0.02 | -0.06 | NC | NC | NC | 1 | K | ko:K03789 |
| AIL05803.1 |  | truA | tRNA pseudouridine(38-40) synthase | -0.24 | -0.21 | -0.08 | NC | NC | NC | 1 | J | ko:K06173 |
| AIL05804.1 |  | pepT | peptidase T | -0.13 | 0.09 | 0.09 | NC | NC | NC | 1 | E | ko:K01258 |
| AIL05805.1 |  | - | PTS system, beta-glucoside-specific IIABC component family protein | 0.47 | 2.17 | 2.44 | NC | U | U | 4 | G | ko:K02755 |
| AIL05806.1 |  | - | glycosyl transferase 2 family protein | -0.67 | -0.18 | -0.36 | NC | NC | NC | 1 | M | ko:K20444 |
| AIL05807.1 |  | XK27_00835 | Cof-like hydrolase family protein | -0.38 | -0.08 | 0.08 | NC | NC | NC | 1 | S | - |
| AIL05808.1 |  | yhfI | metallo-beta-lactamase superfamily protein | 0.23 | 0.22 | 0.24 | NC | NC | NC | 1 | S | - |
| AIL05809.1 |  | mecA | negative regulator of genetic competence family protein | -1.02 | 0.53 | -0.97 | D | NC | NC | 6 | NOT | ko:K16511 |
| AIL05812.1 |  | - | hypothetical protein DR75_706 | 0.71 | 1.48 | 0.90 | NC | U | NC | 15 | G | ko:K01191 |
| AIL05814.1 |  | tadA | cytidine and deoxycytidylate deaminase zinc-binding region family protein | -1.34 | -0.11 | -0.50 | D | NC | NC | 6 | F | ko:K11991 |
| AIL05815.1 |  | - | ABC transporter family protein | 0.03 | 0.03 | -0.19 | NC | NC | NC | 1 | V | ko:K02003 |
| AIL05816.1 |  | folE | GTP cyclohydrolase I | 0.46 | 0.85 | 0.82 | NC | NC | NC | 1 | F | ko:K00950 |
| AIL05819.1 |  | - | helix-turn-helix family protein | 0.13 | 0.14 | -0.08 | NC | NC | NC | 1 | K | - |
| AIL05820.1 |  | dapH | 2,3,4,5-tetrahydropyridine-2,6-dicarboxylate N-acetyltransferase | -0.12 | -0.25 | -0.25 | NC | NC | NC | 1 | E | ko:K00674 |
| AIL05822.1 |  | - | cytidine and deoxycytidylate deaminase zinc-binding region family protein | 0.20 | 0.64 | 0.21 | NC | NC | NC | 1 | F | - |
| AIL05823.1 |  | rsgA | ribosome small subunit-dependent GTPase A | -0.45 | 0.11 | -0.34 | NC | NC | NC | 1 | S | ko:K06949 |
| AIL05824.1 |  | citD | citrate lyase acyl carrier protein | 0.95 | 0.92 | 0.66 | NC | NC | NC | 1 | C | ko:K01646 |
| AIL05825.1 |  | - | glyoxalase-like domain protein | 1.05 | 0.76 | 0.89 | U | NC | NC | 11 | S | ko:K07032 |
| AIL05826.1 |  | fer | 4Fe-4S binding domain protein | -3.20 | 0.61 | 0.44 | D | NC | NC | 6 | C | ko:K05337 |
| AIL05827.1 |  | trmFO | m(5)U-54 methyltransferase | 0.12 | -0.10 | -0.10 | NC | NC | NC | 1 | J | ko:K04094 |
| AIL05828.1 |  | - | helix-turn-helix domain protein | 0.90 | -0.24 | 0.13 | NC | NC | NC | 1 | L | - |
| AIL05831.1 |  | - | ham1 family protein | 0.11 | -0.09 | 0.00 | NC | NC | NC | 1 | F | ko:K02428 |
| AIL05833.1 |  | mvaK2 | phosphomevalonate kinase | -0.41 | -0.46 | -0.33 | NC | NC | NC | 1 | I | ko:K00938 |
| AIL05834.1 |  | citG | triphosphoribosyl-dephospho-CoA synthase CitG | 0.50 | 0.18 | 0.56 | NC | NC | NC | 1 | H | ko:K05966 |
| AIL05835.1 |  | dnaK | chaperone protein DnaK | 0.07 | -0.10 | -0.28 | NC | NC | NC | 1 | O | ko:K04043 |
| AIL05836.1 |  | - | polysaccharide biosynthesis family protein | -2.01 | -0.28 | -0.57 | D | NC | NC | 6 | S | ko:K03328 |
| AIL05837.1 |  | - | hypothetical protein DR75_537 | 0.82 | -0.06 | -0.32 | NC | NC | NC | 1 | S | - |
| AIL05839.1 |  | - | peptidase propeptide and YPEB domain protein | -0.14 | -0.44 | -0.34 | NC | NC | NC | 1 | - | - |
| AIL05840.1 |  | - | uvrB/uvrC motif family protein | 0.36 | 0.05 | 0.10 | NC | NC | NC | 1 | L | ko:K03703 |
| AIL05841.1 |  | ykhA | thioesterase superfamily protein | -0.51 | -0.30 | -0.15 | NC | NC | NC | 1 | I | ko:K01073 |
| AIL05845.1 |  | pepV | dipeptidase PepV | 0.32 | 0.26 | 0.25 | NC | NC | NC | 1 | E | ko:K01270 |
| AIL05846.1 |  | - | PTS system sorbose subIIB component family protein | 0.65 | 0.06 | 0.07 | NC | NC | NC | 1 | G | ko:K02794 |
| AIL05847.1 |  | panE | 2-dehydropantoate 2-reductase family protein | -0.30 | 0.63 | 0.54 | NC | NC | NC | 1 | H | ko:K00077 |
| AIL05848.1 |  | oadA | HMGL-like family protein | 0.60 | 0.49 | 0.18 | NC | NC | NC | 1 | C | ko:K01571 |
| AIL05849.1 |  | gpmA | phosphoglycerate mutase 1 family protein | 0.20 | 0.17 | 0.14 | NC | NC | NC | 1 | G | ko:K01834 |
| AIL05850.1 |  | copZ | heavy-metal-associated domain protein | 0.39 | 0.12 | 0.32 | NC | NC | NC | 1 | P | - |
| AIL05852.1 |  | - | wxL domain surface cell wall-binding family protein | -13.29 | -0.51 | 0.25 | D | NC | NC | 6 | S | - |
| AIL05855.1 |  | lysS | lysine--tRNA ligase | -0.21 | -0.25 | -0.17 | NC | NC | NC | 1 | J | ko:K04567 |
| AIL05856.1 |  | galE | UDP-glucose 4-epimerase GalE | -0.05 | -0.61 | -0.34 | NC | NC | NC | 1 | M | ko:K01784 |
| AIL05857.1 |  | - | hypothetical protein DR75_2329 | 0.35 | -0.02 | -0.02 | NC | NC | NC | 1 | S | - |
| AIL05859.1 |  | typA | GTP-binding protein TypA/BipA | -0.07 | 0.17 | -0.01 | NC | NC | NC | 1 | T | ko:K06207 |
| AIL05861.1 |  | yugI | S1 RNA binding domain protein | -0.15 | -0.15 | -0.56 | NC | NC | NC | 1 | J | ko:K07570 |
| AIL05862.1 |  | busR | trkA-C domain protein | 0.65 | -0.12 | -0.05 | NC | NC | NC | 1 | K | - |
| AIL05863.1 |  | menC | o-succinylbenzoate synthase | -0.13 | 0.07 | 0.54 | NC | NC | NC | 1 | H | ko:K02549 |
| AIL05864.1 |  | rplN | ribosomal protein L14 | -0.27 | -0.31 | -0.29 | NC | NC | NC | 1 | J | ko:K02874 |
| AIL05866.1 |  | apbE | apbE family protein | -0.14 | 0.26 | -1.92 | NC | NC | D | 19 | H | ko:K03734 |
| AIL05867.1 |  | pgm6 | histidine phosphatase super family protein | -0.49 | 0.33 | -0.95 | NC | NC | NC | 1 | G | - |
| AIL05868.1 |  | ylmH | S4 domain protein | 0.50 | 0.26 | 0.13 | NC | NC | NC | 1 | S | - |
| AIL05869.1 |  | truA | tRNA pseudouridine(38-40) synthase | 0.13 | -0.18 | 0.20 | NC | NC | NC | 1 | J | ko:K06173 |
| AIL05872.1 |  | murG | undecaprenyldiphospho-muramoylpentapeptide beta-N-acetylglucosaminyltransferase | -1.03 | -0.26 | -0.34 | D | NC | NC | 6 | M | ko:K02563 |
| AIL05873.1 |  | ptpA | low molecular weight protein-tyrosine-phosphatase yfkJ | -0.07 | 0.18 | 0.61 | NC | NC | NC | 1 | T | ko:K01104 |
| AIL05875.1 |  | - | hemolysin XhlA family protein | 0.82 | 1.01 | 0.68 | NC | U | NC | 15 | S | - |
| AIL05876.1 |  | prmA | ribosomal protein L11 methyltransferase | 0.00 | -0.19 | 0.04 | NC | NC | NC | 1 | J | ko:K02687 |
| AIL05877.1 |  | mtlD | UDP-glucose/GDP-mannose dehydrogenase family, NAD binding domain protein | 0.28 | 1.53 | 0.85 | NC | U | NC | 15 | G | ko:K00009 |
| AIL05878.1 |  | coaD | pantetheine-phosphate adenylyltransferase | -0.22 | 1.15 | -0.33 | NC | U | NC | 15 | H | ko:K00954 |
| AIL05881.1 |  | sitB | ABC transporter family protein | -0.30 | -1.06 | -1.28 | NC | D | D | 22 | P | ko:K02074 |
| AIL05882.1 |  | hprK | HPr(Ser) kinase/phosphatase | -0.03 | 0.23 | 0.02 | NC | NC | NC | 1 | F | ko:K06023 |
| AIL05883.1 |  | - | hypothetical protein DR75_1519 | -0.34 | -0.48 | -0.52 | NC | NC | NC | 1 | - | - |
| AIL05885.1 |  | ytxH | ytxH-like family protein | 1.16 | 0.14 | 0.09 | U | NC | NC | 11 | S | - |
| AIL05887.1 |  | uspA | universal stress family protein | 0.52 | 0.37 | 0.58 | NC | NC | NC | 1 | T | - |
| AIL05892.1 |  | mdlA | ABC transporter family protein | -0.43 | -0.04 | -0.17 | NC | NC | NC | 1 | V | ko:K06148 |
| AIL05895.1 |  | - | PTS system, glucose subfamily, IIA component domain protein | -0.66 | 0.17 | -0.06 | NC | NC | NC | 1 | G | ko:K02777 |
| AIL05897.1 |  | ypdA | binding--dependent transport system inner membrane component family protein | 0.65 | 1.94 | 1.78 | NC | U | U | 4 | U | ko:K17319 |
| AIL05899.1 |  | cysK | cysteine synthase A | 0.77 | 0.49 | 0.49 | NC | NC | NC | 1 | E | ko:K01738 |
| AIL05900.1 |  | rplU | ribosomal protein L21 | -0.09 | 0.04 | -0.21 | NC | NC | NC | 1 | J | ko:K02888 |
| AIL05903.1 |  | - | reactive intermediate/imine deaminase family protein | 0.45 | 0.71 | 0.80 | NC | NC | NC | 1 | J | - |
| AIL05905.1 |  | pgm7 | histidine phosphatase super family protein | 0.10 | 0.74 | 0.84 | NC | NC | NC | 1 | G | - |
| AIL05906.1 |  | yqeG | HAD hydrolase, IA, variant 1 family protein | -0.34 | -0.87 | -0.99 | NC | NC | NC | 1 | S | ko:K07015 |
| AIL05907.1 |  | - | hypothetical protein DR75_1058 | 0.16 | -0.41 | 0.08 | NC | NC | NC | 1 | S | - |
| AIL05910.1 |  | cobQ | cobB/CobQ-like glutamine amidotransferase domain protein | 0.01 | -0.15 | 0.02 | NC | NC | NC | 1 | S | ko:K07009 |
| AIL05911.1 |  | hpt | hypoxanthine phosphoribosyltransferase | -0.14 | -0.07 | -0.07 | NC | NC | NC | 1 | F | ko:K00760 |
| AIL05913.1 |  | ntpB | V-type sodium ATPase subunit B | -0.09 | -0.16 | -0.05 | NC | NC | NC | 1 | C | ko:K02118 |
| AIL05914.1 |  | - | hypothetical protein DR75_1358 | -0.07 | -3.08 | -2.82 | NC | D | D | 22 | S | - |
| AIL05917.1 |  | fmt | methionyl-tRNA formyltransferase | 0.07 | -0.12 | -0.08 | NC | NC | NC | 1 | J | ko:K00604 |
| AIL05919.1 |  | ispA | farnesyl diphosphate synthase | -0.48 | -0.25 | -0.18 | NC | NC | NC | 1 | H | ko:K13789 |
| AIL05920.1 |  | WQ51_01275 | EDD, DegV family domain protein | 0.21 | -0.01 | 0.11 | NC | NC | NC | 1 | S | - |
| AIL05921.1 |  | cat | cation diffusion facilitator transporter family protein | 0.12 | -0.03 | -0.36 | NC | NC | NC | 1 | P | - |
| AIL05923.1 |  | galT | galactose-1-phosphate uridylyltransferase | -0.06 | 1.40 | 1.46 | NC | U | U | 4 | G | ko:K00965 |
| AIL05924.1 |  | - | alpha/beta hydrolase fold family protein | -0.08 | -0.06 | -0.12 | NC | NC | NC | 1 | I | - |
| AIL05925.1 |  | - | hypothetical protein DR75_1438 | -0.32 | -0.54 | 0.10 | NC | NC | NC | 1 | S | - |
| AIL05927.1 |  | - | cbp21 | 1.63 | 0.16 | 0.71 | U | NC | NC | 11 | S | ko:K21713 |
| AIL05928.1 |  | - | hypothetical protein DR75_1135 | -0.45 | -1.85 | 1.58 | NC | D | U | 7 | KL | - |
| AIL05929.1 |  | - | putative lipoprotein | -0.99 | -0.41 | -0.29 | NC | NC | NC | 1 | - | - |
| AIL05930.1 |  | - | SIS domain protein | 0.08 | -0.05 | -0.37 | NC | NC | NC | 1 | K | - |
| AIL05931.1 |  | manY | PTS system sorbose-specific iic component family protein | -0.26 | 1.72 | 1.19 | NC | U | U | 4 | G | ko:K02746 |
| AIL05933.1 |  | trePP | trehalose 6-phosphate phosphorylase | -0.06 | 0.66 | 0.49 | NC | NC | NC | 1 | G | ko:K00691 |
| AIL05934.1 |  | - | hypothetical protein DR75_898 | 1.01 | 0.24 | -0.09 | U | NC | NC | 11 | - | - |
| AIL05936.1 |  | malY | C-S lyase family protein | 0.55 | 1.05 | 0.87 | NC | U | NC | 15 | E | ko:K14155 |
| AIL05937.1 |  | uppP | undecaprenyl-diphosphatase UppP | -0.22 | -1.54 | -1.73 | NC | D | D | 22 | V | ko:K06153 |
| AIL05938.1 |  | pphA | calcineurin-like phosphoesterase family protein | -0.13 | -0.48 | -1.41 | NC | NC | D | 19 | T | ko:K07313 |
| AIL05940.1 |  | yfmL | hypothetical protein DR75_69 | -0.16 | 0.55 | -0.35 | NC | NC | NC | 1 | L | - |
| AIL05944.1 |  | - | hypothetical protein DR75_1473 | -0.60 | -0.92 | -0.80 | NC | NC | NC | 1 | G | ko:K00965 |

**Table. S2**. COG functional classification (detailed categories) of differentially expressed proteins in *E. faecalis* ATCC 29212 after copper NP treatment.

|  |  | **Cellular processes and signaling** | | | | | | | **Information storage and processing** | | | **Metabolism** | | | | | | | | **Poorly characterized** |
| --- | --- | --- | --- | --- | --- | --- | --- | --- | --- | --- | --- | --- | --- | --- | --- | --- | --- | --- | --- | --- |
|  |  | **D** | **V** | **T** | **M** | **N** | **U** | **O** | **J** | **K** | **L** | **C** | **G** | **E** | **F** | **H** | **I** | **P** | **Q** | **S** |
| **Upregulated** | **10 min** | 1 | 2 | 1 | 5 | 0 | 3 | 3 | 5 | 4 | 5 | 4 | 15 | 7 | 2 | 7 | 1 | 7 | 1 | 21 |
|  | **30 min** | 0 | 3 | 3 | 5 | 0 | 3 | 1 | 3 | 10 | 3 | 12 | 32 | 7 | 2 | 5 | 3 | 2 | 1 | 14 |
|  | **60 min** | 0 | 3 | 3 | 3 | 0 | 3 | 0 | 2 | 7 | 4 | 9 | 23 | 5 | 2 | 2 | 3 | 1 | 1 | 12 |
| **Downregulated** | **10 min** | 1 | 3 | 2 | 6 | 1 | 3 | 4 | 1 | 8 | 6 | 7 | 13 | 13 | 10 | 1 | 2 | 2 | 1 | 19 |
|  | **30 min** | 1 | 3 | 0 | 3 | 1 | 1 | 3 | 2 | 7 | 6 | 6 | 4 | 6 | 2 | 4 | 1 | 2 | 1 | 16 |
|  | **60 min** | 1 | 1 | 1 | 3 | 1 | 4 | 1 | 2 | 7 | 5 | 2 | 5 | 4 | 2 | 2 | 1 | 1 | 1 | 15 |

**Table. S3**. KEGG pathways of upregulated proteins from *E. faecalis* ATCC 29212 following copper NP treatment.

| **General description** | **Functional category** | **KEGG pathway** | **Protein count** | | |
| --- | --- | --- | --- | --- | --- |
|  |  |  | **10 min** | **30 min** | **60 min** |
| Metabolism | Global and overview maps | 01100 Metabolic pathways | 35 | 58 | 39 |
|  |  | 01110 Biosynthesis of secondary metabolites | 12 | 10 | 9 |
|  |  | 01120 Microbial metabolism in diverse environments | 12 | 16 | 12 |
|  |  | 01200 Carbon metabolism | 9 | 7 | 9 |
|  |  | 01210 2-Oxocarboxylic acid metabolism | 0 | 2 | 4 |
|  |  | 01212 Fatty acid metabolism | 0 | 0 | 0 |
|  |  | 01230 Biosynthesis of amino acids | 3 | 3 | 1 |
|  |  | 01232 Nucleotide metabolism | 0 | 0 | 0 |
|  |  | 01250 Biosynthesis of nucleotide sugars | 4 | 7 | 7 |
|  |  | 01240 Biosynthesis of cofactors | 7 | 5 | 5 |
|  |  | 01220 Degradation of aromatic compounds | 0 | 0 | 0 |
|  | Carbohydrate metabolism | 00010 Glycolysis / Gluconeogenesis | 4 | 11 | 13 |
|  |  | 00020 Citrate cycle (TCA cycle) | 0 | 2 | 4 |
|  |  | 00030 Pentose phosphate pathway | 4 | 4 | 3 |
|  |  | 00040 Pentose and glucuronate interconversions | 0 | 1 | 1 |
|  |  | 00051 Fructose and mannose metabolism | 2 | 12 | 6 |
|  |  | 00052 Galactose metabolism | 8 | 15 | 11 |
|  |  | 00053 Ascorbate and aldarate metabolism | 0 | 3 | 3 |
|  |  | 00500 Starch and sucrose metabolism | 10 | 12 | 9 |
|  |  | 00520 Amino sugar and nucleotide sugar metabolism | 4 | 15 | 10 |
|  |  | 00541 Biosynthesis of various nucleotide sugars | 4 | 4 | 4 |
|  |  | 00620 Pyruvate metabolism | 1 | 4 | 6 |
|  |  | 00630 Glyoxylate and dicarboxylate metabolism | 3 | 5 | 5 |
|  |  | 00640 Propanoate metabolism | 0 | 3 | 3 |
|  |  | 00650 Butanoate metabolism | 0 | 2 | 3 |
|  |  | 00660 C5-Branched dibasic acid metabolism | 0 | 0 | 0 |
|  |  | 00562 Inositol phosphate metabolism | 0 | 0 | 0 |
|  | Energy metabolism | 00190 Oxidative phosphorylation | 1 | 0 | 0 |
|  |  | 00710 Carbon fixation by Calvin cycle | 4 | 0 | 0 |
|  |  | 00720 Other carbon fixation pathways | 1 | 0 | 2 |
|  |  | 00680 Methane metabolism | 1 | 0 | 0 |
|  |  | 00910 Nitrogen metabolism | 3 | 0 | 0 |
|  |  | 00920 Sulfur metabolism | 0 | 0 | 0 |
|  | Lipid metabolism | 00061 Fatty acid biosynthesis | 0 | 0 | 0 |
|  |  | 00071 Fatty acid degradation | 0 | 0 | 0 |
|  |  | 00074 Mycolic acid biosynthesis | 0 | 0 | 1 |
|  |  | 00561 Glycerolipid metabolism | 0 | 3 | 2 |
|  |  | 00564 Glycerophospholipid metabolism | 0 | 2 | 0 |
|  |  | 00600 Sphingolipid metabolism | 0 | 0 | 0 |
|  | Nucleotide metabolism | 00230 Purine metabolism | 5 | 2 | 1 |
|  |  | 00240 Pyrimidine metabolism | 3 | 0 | 2 |
|  | Amino acid metabolism | 00250 Alanine, aspartate and glutamate metabolism | 0 | 0 | 0 |
|  |  | 00260 Glycine, serine and threonine metabolism | 0 | 2 | 2 |
|  |  | 00270 Cysteine and methionine metabolism | 2 | 4 | 0 |
|  |  | 00280 Valine, leucine and isoleucine degradation | 0 | 2 | 2 |
|  |  | 00290 Valine, leucine and isoleucine biosynthesis | 0 | 0 | 0 |
|  |  | 00300 Lysine biosynthesis | 1 | 1 | 1 |
|  |  | 00310 Lysine degradation | 0 | 2 | 2 |
|  |  | 00220 Arginine biosynthesis | 3 | 0 | 0 |
|  |  | 00330 Arginine and proline metabolism | 1 | 2 | 0 |
|  |  | 00350 Tyrosine metabolism | 0 | 0 | 0 |
|  |  | 00380 Tryptophan metabolism | 0 | 4 | 2 |
|  |  | 00400 Phenylalanine, tyrosine and tryptophan biosynthesis | 0 | 0 | 0 |
|  | Metabolism of other amino acids | 00410 beta-Alanine metabolism | 0 | 0 | 0 |
|  |  | 00430 Taurine and hypotaurine metabolism | 0 | 0 | 0 |
|  |  | 00450 Selenocompound metabolism | 0 | 2 | 0 |
|  |  | 00460 Cyanoamino acid metabolism | 0 | 0 | 0 |
|  |  | 00470 D-Amino acid metabolism | 0 | 0 | 0 |
|  |  | 00480 Glutathione metabolism | 0 | 0 | 0 |
|  | Glycan biosynthesis and metabolism | 00511 Other glycan degradation | 0 | 0 | 0 |
|  |  | 00542 O-Antigen repeat unit biosynthesis | 0 | 0 | 0 |
|  |  | 00550 Peptidoglycan biosynthesis | 0 | 0 | 0 |
|  |  | 00552 Teichoic acid biosynthesis | 0 | 0 | 0 |
|  |  | 00543 Exopolysaccharide biosynthesis | 0 | 0 | 0 |
|  | Metabolism of cofactors and vitamins | 00730 Thiamine metabolism | 1 | 2 | 1 |
|  |  | 00740 Riboflavin metabolism | 1 | 0 | 1 |
|  |  | 00750 Vitamin B6 metabolism | 0 | 0 | 0 |
|  |  | 00760 Nicotinate and nicotinamide metabolism | 0 | 0 | 0 |
|  |  | 00770 Pantothenate and CoA biosynthesis | 0 | 1 | 1 |
|  |  | 00780 Biotin metabolism | 0 | 0 | 0 |
|  |  | 00785 Lipoic acid metabolism | 0 | 2 | 2 |
|  |  | 00790 Folate biosynthesis | 0 | 0 | 0 |
|  |  | 00670 One carbon pool by folate | 0 | 2 | 2 |
|  |  | 00860 Porphyrin metabolism | 0 | 0 | 0 |
|  |  | 00130 Ubiquinone and other terpenoid-quinone biosynthesis | 3 | 0 | 0 |
|  | Metabolism of terpenoids and polyketides | 00900 Terpenoid backbone biosynthesis | 0 | 0 | 0 |
|  |  | 00523 Polyketide sugar unit biosynthesis | 0 | 0 | 0 |
|  | Biosynthesis of other secondary metabolites | 00946 Degradation of flavonoids | 0 | 0 | 0 |
|  |  | 00332 Carbapenem biosynthesis | 0 | 0 | 0 |
|  |  | 00261 Monobactam biosynthesis | 0 | 0 | 0 |
|  |  | 00521 Streptomycin biosynthesis | 0 | 0 | 0 |
|  |  | 00525 Acarbose and validamycin biosynthesis | 0 | 0 | 0 |
|  |  | 00999 Biosynthesis of various plant secondary metabolites | 0 | 0 | 0 |
|  | Xenobiotics biodegradation and metabolism | 00362 Benzoate degradation | 0 | 0 | 0 |
|  |  | 00627 Aminobenzoate degradation | 0 | 2 | 0 |
|  |  | 00625 Chloroalkane and chloroalkene degradation | 0 | 0 | 0 |
|  |  | 00361 Chlorocyclohexane and chlorobenzene degradation | 0 | 0 | 0 |
|  |  | 00622 Xylene degradation | 0 | 0 | 0 |
|  |  | 00643 Styrene degradation | 0 | 2 | 0 |
|  |  | 00626 Naphthalene degradation | 0 | 0 | 0 |
| Genetic Information Processing | Transcription | 03020 RNA polymerase | 0 | 0 | 0 |
|  | Translation | 03010 Ribosome | 0 | 1 | 0 |
|  |  | 00970 Aminoacyl-tRNA biosynthesis | 0 | 0 | 0 |
|  | Folding, sorting and degradation | 03060 Protein export | 0 | 0 | 0 |
|  |  | 04122 Sulfur relay system | 0 | 0 | 0 |
|  |  | 03018 RNA degradation | 0 | 0 | 0 |
|  | Replication and repair | 03030 DNA replication | 0 | 0 | 0 |
|  |  | 03410 Base excision repair | 1 | 0 | 0 |
|  |  | 03420 Nucleotide excision repair | 0 | 0 | 0 |
|  |  | 03430 Mismatch repair | 0 | 0 | 0 |
|  |  | 03440 Homologous recombination | 1 | 1 | 1 |
|  | Information processing in viruses | 03250 Viral life cycle - HIV-1 | 0 | 0 | 0 |
| Environmental Information Processing | Membrane transport | 02010 ABC transporters | 6 | 26 | 23 |
|  |  | 02060 Phosphotransferase system (PTS) | 11 | 16 | 9 |
|  |  | 03070 Bacterial secretion system | 0 | 0 | 0 |
|  | Signal transduction | 02020 Two-component system | 0 | 4 | 4 |
| Cellular Processes | Transport and catabolism | 04146 Peroxisome | 1 | 1 | 1 |
|  | Cellular community - prokaryotes | 02024 Quorum sensing | 0 | 11 | 11 |
|  | Cell motility | 02040 Flagellar assembly | 0 | 0 | 0 |
| Organismal Systems | Digestive system | 04981 Folate transport and metabolism | 0 | 0 | 0 |
| Human Diseases | Drug resistance: antimicrobial | 01501 beta-Lactam resistance | 0 | 9 | 9 |
|  |  | 01502 Vancomycin resistance | 0 | 0 | 0 |
|  |  | 01503 Cationic antimicrobial peptide (CAMP) resistance | 0 | 0 | 0 |

**Table. S4**. KEGG pathways of downregulated proteins from *E. faecalis* ATCC 29212 following copper NP treatment.

| **General description** | **Functional category** | **KEGG pathway** | **Protein count** | | |
| --- | --- | --- | --- | --- | --- |
|  |  |  | **10 min** | **30 min** | **60 min** |
| Metabolism | Global and overview maps | 01100 Metabolic pathways | 40 | 17 | 14 |
|  |  | 01110 Biosynthesis of secondary metabolites | 14 | 7 | 8 |
|  |  | 01120 Microbial metabolism in diverse environments | 10 | 6 | 5 |
|  |  | 01200 Carbon metabolism | 10 | 5 | 5 |
|  |  | 01210 2-Oxocarboxylic acid metabolism | 4 | 2 | 0 |
|  |  | 01212 Fatty acid metabolism | 1 | 0 | 0 |
|  |  | 01230 Biosynthesis of amino acids | 4 | 1 | 1 |
|  |  | 01232 Nucleotide metabolism | 4 | 1 | 0 |
|  |  | 01250 Biosynthesis of nucleotide sugars | 2 | 0 | 0 |
|  |  | 01240 Biosynthesis of cofactors | 8 | 4 | 2 |
|  |  | 01220 Degradation of aromatic compounds | 0 | 0 | 0 |
|  | Carbohydrate metabolism | 00010 Glycolysis / Gluconeogenesis | 4 | 2 | 0 |
|  |  | 00020 Citrate cycle (TCA cycle) | 4 | 2 | 0 |
|  |  | 00030 Pentose phosphate pathway | 3 | 2 | 2 |
|  |  | 00040 Pentose and glucuronate interconversions | 0 | 1 | 1 |
|  |  | 00051 Fructose and mannose metabolism | 7 | 1 | 1 |
|  |  | 00052 Galactose metabolism | 3 | 0 | 2 |
|  |  | 00053 Ascorbate and aldarate metabolism | 0 | 0 | 0 |
|  |  | 00500 Starch and sucrose metabolism | 2 | 0 | 2 |
|  |  | 00520 Amino sugar and nucleotide sugar metabolism | 7 | 0 | 0 |
|  |  | 00541 Biosynthesis of various nucleotide sugars | 0 | 0 | 0 |
|  |  | 00620 Pyruvate metabolism | 4 | 3 | 0 |
|  |  | 00630 Glyoxylate and dicarboxylate metabolism | 5 | 0 | 0 |
|  |  | 00640 Propanoate metabolism | 2 | 1 | 0 |
|  |  | 00650 Butanoate metabolism | 2 | 3 | 0 |
|  |  | 00660 C5-Branched dibasic acid metabolism | 0 | 0 | 0 |
|  |  | 00562 Inositol phosphate metabolism | 0 | 0 | 0 |
|  | Energy metabolism | 00190 Oxidative phosphorylation | 0 | 1 | 1 |
|  |  | 00710 Carbon fixation by Calvin cycle | 0 | 0 | 0 |
|  |  | 00720 Other carbon fixation pathways | 2 | 2 | 0 |
|  |  | 00680 Methane metabolism | 0 | 0 | 0 |
|  |  | 00910 Nitrogen metabolism | 3 | 3 | 3 |
|  |  | 00920 Sulfur metabolism | 0 | 0 | 0 |
|  | Lipid metabolism | 00061 Fatty acid biosynthesis | 1 | 0 | 0 |
|  |  | 00071 Fatty acid degradation | 0 | 0 | 0 |
|  |  | 00074 Mycolic acid biosynthesis | 0 | 0 | 0 |
|  |  | 00561 Glycerolipid metabolism | 0 | 0 | 0 |
|  |  | 00564 Glycerophospholipid metabolism | 3 | 0 | 0 |
|  |  | 00600 Sphingolipid metabolism | 0 | 0 | 0 |
|  | Nucleotide metabolism | 00230 Purine metabolism | 5 | 3 | 3 |
|  |  | 00240 Pyrimidine metabolism | 6 | 1 | 0 |
|  | Amino acid metabolism | 00250 Alanine, aspartate and glutamate metabolism | 2 | 0 | 0 |
|  |  | 00260 Glycine, serine and threonine metabolism | 2 | 0 | 0 |
|  |  | 00270 Cysteine and methionine metabolism | 0 | 0 | 0 |
|  |  | 00280 Valine, leucine and isoleucine degradation | 2 | 0 | 0 |
|  |  | 00290 Valine, leucine and isoleucine biosynthesis | 0 | 0 | 0 |
|  |  | 00300 Lysine biosynthesis | 0 | 0 | 0 |
|  |  | 00310 Lysine degradation | 2 | 0 | 0 |
|  |  | 00220 Arginine biosynthesis | 4 | 3 | 3 |
|  |  | 00330 Arginine and proline metabolism | 0 | 0 | 0 |
|  |  | 00350 Tyrosine metabolism | 0 | 0 | 0 |
|  |  | 00380 Tryptophan metabolism | 2 | 0 | 0 |
|  |  | 00400 Phenylalanine, tyrosine and tryptophan biosynthesis | 4 | 1 | 1 |
|  | Metabolism of other amino acids | 00410 beta-Alanine metabolism | 0 | 0 | 0 |
|  |  | 00430 Taurine and hypotaurine metabolism | 0 | 0 | 0 |
|  |  | 00450 Selenocompound metabolism | 2 | 2 | 0 |
|  |  | 00460 Cyanoamino acid metabolism | 0 | 0 | 0 |
|  |  | 00470 D-Amino acid metabolism | 0 | 0 | 0 |
|  |  | 00480 Glutathione metabolism | 0 | 0 | 2 |
|  | Glycan biosynthesis and metabolism | 00511 Other glycan degradation | 0 | 0 | 0 |
|  |  | 00542 O-Antigen repeat unit biosynthesis | 0 | 0 | 0 |
|  |  | 00550 Peptidoglycan biosynthesis | 3 | 1 | 1 |
|  |  | 00552 Teichoic acid biosynthesis | 0 | 1 | 1 |
|  |  | 00543 Exopolysaccharide biosynthesis | 0 | 0 | 0 |
|  | Metabolism of cofactors and vitamins | 00730 Thiamine metabolism | 0 | 0 | 0 |
|  |  | 00740 Riboflavin metabolism | 0 | 0 | 0 |
|  |  | 00750 Vitamin B6 metabolism | 0 | 0 | 0 |
|  |  | 00760 Nicotinate and nicotinamide metabolism | 0 | 1 | 0 |
|  |  | 00770 Pantothenate and CoA biosynthesis | 0 | 0 | 0 |
|  |  | 00780 Biotin metabolism | 0 | 0 | 0 |
|  |  | 00785 Lipoic acid metabolism | 4 | 2 | 2 |
|  |  | 00790 Folate biosynthesis | 0 | 1 | 0 |
|  |  | 00670 One carbon pool by folate | 2 | 0 | 0 |
|  |  | 00860 Porphyrin metabolism | 0 | 0 | 0 |
|  |  | 00130 Ubiquinone and other terpenoid-quinone biosynthesis | 0 | 0 | 0 |
|  | Metabolism of terpenoids and polyketides | 00900 Terpenoid backbone biosynthesis | 0 | 0 | 0 |
|  |  | 00523 Polyketide sugar unit biosynthesis | 0 | 0 | 0 |
|  | Biosynthesis of other secondary metabolites | 00946 Degradation of flavonoids | 0 | 0 | 0 |
|  |  | 00332 Carbapenem biosynthesis | 0 | 0 | 0 |
|  |  | 00261 Monobactam biosynthesis | 0 | 0 | 0 |
|  |  | 00521 Streptomycin biosynthesis | 0 | 0 | 0 |
|  |  | 00525 Acarbose and validamycin biosynthesis | 0 | 0 | 0 |
|  |  | 00999 Biosynthesis of various plant secondary metabolites | 0 | 0 | 0 |
|  | Xenobiotics biodegradation and metabolism | 00362 Benzoate degradation | 0 | 0 | 0 |
|  |  | 00627 Aminobenzoate degradation | 0 | 0 | 0 |
|  |  | 00625 Chloroalkane and chloroalkene degradation | 0 | 0 | 0 |
|  |  | 00361 Chlorocyclohexane and chlorobenzene degradation | 0 | 0 | 0 |
|  |  | 00622 Xylene degradation | 0 | 0 | 0 |
|  |  | 00643 Styrene degradation | 0 | 0 | 0 |
|  |  | 00626 Naphthalene degradation | 0 | 0 | 0 |
| Genetic Information Processing | Transcription | 03020 RNA polymerase | 0 | 0 | 0 |
|  | Translation | 03010 Ribosome | 0 | 2 | 2 |
|  |  | 00970 Aminoacyl-tRNA biosynthesis | 0 | 0 | 2 |
|  | Folding, sorting and degradation | 03060 Protein export | 0 | 0 | 2 |
|  |  | 04122 Sulfur relay system | 0 | 0 | 0 |
|  |  | 03018 RNA degradation | 0 | 0 | 0 |
|  | Replication and repair | 03030 DNA replication | 6 | 5 | 2 |
|  |  | 03410 Base excision repair | 0 | 0 | 0 |
|  |  | 03420 Nucleotide excision repair | 0 | 0 | 0 |
|  |  | 03430 Mismatch repair | 4 | 5 | 1 |
|  |  | 03440 Homologous recombination | 4 | 5 | 1 |
|  | Information processing in viruses | 03250 Viral life cycle - HIV-1 | 0 | 0 | 0 |
| Environmental Information Processing | Membrane transport | 02010 ABC transporters | 7 | 1 | 3 |
|  |  | 02060 Phosphotransferase system (PTS) | 11 | 0 | 1 |
|  |  | 03070 Bacterial secretion system | 0 | 0 | 2 |
|  | Signal transduction | 02020 Two-component system | 1 | 1 | 1 |
| Cellular Processes | Transport and catabolism | 04146 Peroxisome | 0 | 0 | 0 |
|  | Cellular community - prokaryotes | 02024 Quorum sensing | 6 | 3 | 5 |
|  | Cell motility | 02040 Flagellar assembly | 0 | 0 | 0 |
| Organismal Systems | Digestive system | 04981 Folate transport and metabolism | 0 | 0 | 0 |
| Human Diseases | Drug resistance: antimicrobial | 01501 beta-Lactam resistance | 0 | 0 | 0 |
|  |  | 01502 Vancomycin resistance | 1 | 0 | 0 |
|  |  | 01503 Cationic antimicrobial peptide (CAMP) resistance | 0 | 0 | 0 |

**Table. S5**. Proteins that remained consistently unchanged throughout the time course of copper NP treatment in *E. faecalis* ATCC 29212.

| **Protein ID** | **Gene name** | **Protein description** | **Fold Ratio** | | | **Expression Pattern (EP)** | | | **EP group** | **COG** | **KEGG number** |
| --- | --- | --- | --- | --- | --- | --- | --- | --- | --- | --- | --- |
|  |  |  | **10 min** | **30 min** | **60 min** | **10 min** | **30 min** | **60 min** |  |  |  |
| AIL04754.1 | rpoC | DNA-directed RNA polymerase, beta' subunit | -0.06 | 0.02 | -0.06 | NC | NC | NC | 1 | K | ko:K03046 |
| AIL05342.1 | rpoB | DNA-directed RNA polymerase, beta subunit | -0.08 | -0.05 | -0.08 | NC | NC | NC | 1 | K | ko:K03043 |
| AIL04671.1 | smc | chromosome segregation protein SMC | -0.83 | -0.39 | -0.02 | NC | NC | NC | 1 | D | ko:K03529 |
| AIL04229.1 | uvrA | excinuclease ABC subunit A | 0.66 | -0.11 | 0.04 | NC | NC | NC | 1 | L | ko:K03701 |
| AIL04386.1 | clpB | ATP-dependent chaperone protein ClpB | 0.50 | 0.12 | 0.15 | NC | NC | NC | 1 | O | ko:K03695 |
| AIL03670.1 | clpE | ATP-dependent Clp protease ATP-binding subunit ClpE | 0.64 | 0.00 | -0.08 | NC | NC | NC | 1 | O | ko:K03697 |
| AIL04065.1 | pyc | pyruvate carboxylase | -0.23 | 0.20 | 0.08 | NC | NC | NC | 1 | C | ko:K01958 |
| AIL04285.1 | argS | arginine--tRNA ligase | -0.40 | 0.07 | -0.13 | NC | NC | NC | 1 | J | ko:K01887 |
| AIL05835.1 | dnaK | chaperone protein DnaK | 0.07 | -0.10 | -0.28 | NC | NC | NC | 1 | O | ko:K04043 |
| AIL04434.1 | valS | valine--tRNA ligase | -0.34 | 0.07 | 0.01 | NC | NC | NC | 1 | J | ko:K01873 |
| AIL05196.1 | secA | preprotein translocase, SecA subunit | -0.46 | -0.17 | -0.23 | NC | NC | NC | 1 | U | ko:K03070 |
| AIL03851.1 | mfd | transcription-repair coupling factor | 0.42 | 0.07 | -0.09 | NC | NC | NC | 1 | L | ko:K03723 |
| AIL05546.1 | ftsH | ATP-dependent metallopeptidase HflB family protein | 0.18 | -0.18 | -0.12 | NC | NC | NC | 1 | O | ko:K03798 |
| AIL04557.1 | nrdE | ribonucleoside-diphosphate reductase, alpha subunit | 0.93 | 0.09 | 0.05 | NC | NC | NC | 1 | F | ko:K00525 |
| AIL04833.1 | ileS | isoleucine--tRNA ligase | -0.30 | 0.05 | 0.06 | NC | NC | NC | 1 | J | ko:K01870 |
| AIL04629.1 | polA | DNA polymerase I | 0.13 | 0.00 | 0.01 | NC | NC | NC | 1 | L | ko:K02335 |
| AIL04498.1 | tex | S1 RNA binding domain protein | -0.14 | -0.08 | -0.06 | NC | NC | NC | 1 | K | ko:K06959 |
| AIL05357.1 | alaS | alanine--tRNA ligase | -0.23 | 0.22 | -0.03 | NC | NC | NC | 1 | J | ko:K01872 |
| AIL05440.1 | clpC | istB-like ATP binding family protein | -0.42 | -0.14 | -0.41 | NC | NC | NC | 1 | O | ko:K03696 |
| AIL04196.1 | metG | methionine--tRNA ligase | -0.21 | -0.01 | 0.02 | NC | NC | NC | 1 | J | ko:K01874 |
| AIL05806.1 | - | glycosyl transferase 2 family protein | -0.67 | -0.18 | -0.36 | NC | NC | NC | 1 | M | ko:K20444 |
| AIL04106.1 | murB | UDP-N-acetylenolpyruvoylglucosamine reductase | -0.96 | -0.39 | -0.07 | NC | NC | NC | 1 | M | ko:K00075 |
| AIL04347.1 | hsdR | DEAD/DEAH box helicase family protein | -0.38 | 0.14 | 0.04 | NC | NC | NC | 1 | V | ko:K01153 |
| AIL03334.1 | pnp | polyribonucleotide nucleotidyltransferase | -0.14 | -0.20 | -0.28 | NC | NC | NC | 1 | J | ko:K00962 |
| AIL03341.1 | addA | helicase-exonuclease AddAB, AddA subunit | -0.09 | -0.08 | -0.07 | NC | NC | NC | 1 | L | ko:K16898 |
| AIL03843.1 | yvlB | hypothetical protein DR75_751 | 0.41 | 0.30 | 0.28 | NC | NC | NC | 1 | D | - |
| AIL03859.1 | relA | RelA/SpoT family protein | 0.04 | -0.11 | -0.18 | NC | NC | NC | 1 | KT | ko:K00951 |
| AIL04934.1 | nrdD | anaerobic ribonucleoside-triphosphate reductase | 0.23 | -0.48 | -0.45 | NC | NC | NC | 1 | FK | ko:K21636 |
| AIL03318.1 | gyrA | DNA gyrase, A subunit | -0.12 | -0.86 | 0.26 | NC | NC | NC | 1 | L | ko:K02469 |
| AIL03405.1 | groL | chaperonin GroL | -0.09 | -0.38 | -0.41 | NC | NC | NC | 1 | O | ko:K04077 |
| AIL05643.1 | mutS2 | MutS2 family protein | -0.12 | -0.05 | -0.22 | NC | NC | NC | 1 | L | ko:K07456 |
| AIL04491.1 | ligA | DNA ligase, NAD-dependent | 0.33 | 0.04 | 0.01 | NC | NC | NC | 1 | L | ko:K01972 |
| AIL05529.1 | pcrA | ATP-dependent DNA helicase PcrA | -0.09 | -0.17 | -0.14 | NC | NC | NC | 1 | L | ko:K03657 |
| AIL04341.1 | fusA | translation elongation factor G | -0.25 | -0.17 | -0.26 | NC | NC | NC | 1 | J | ko:K02355 |
| AIL05566.1 | glyS | glycine--tRNA ligase, beta subunit | -0.46 | -0.24 | -0.11 | NC | NC | NC | 1 | J | ko:K01879 |
| AIL03189.1 | salB | type 2 lantibiotic biosynthesis LanM family protein (plasmid) | -0.45 | 0.10 | 0.27 | NC | NC | NC | 1 | V | ko:K20385 |
| AIL04536.1 | gatB | aspartyl/glutamyl-tRNA(Asn/Gln) amidotransferase, B subunit | -0.07 | 0.07 | -0.07 | NC | NC | NC | 1 | J | ko:K02434 |
| AIL04562.1 | pacL | HAD ATPase, P-type, IC family protein | 0.10 | -0.15 | -0.24 | NC | NC | NC | 1 | P | ko:K01537 |
| AIL05496.1 | ezrA | septation ring formation regulator, EzrA family protein | -0.42 | -0.16 | -0.15 | NC | NC | NC | 1 | D | ko:K06286 |
| AIL03815.1 | polC | DNA polymerase III, alpha subunit, Gram-positive type | 0.67 | 0.35 | 0.35 | NC | NC | NC | 1 | L | ko:K03763 |
| AIL05098.1 | - | SPFH domain / Band 7 family protein | 0.18 | 0.59 | 0.58 | NC | NC | NC | 1 | S | ko:K07192 |
| AIL03892.1 | tdc | tyrosine decarboxylase | -0.14 | -0.51 | -0.18 | NC | NC | NC | 1 | E | ko:K22330 |
| AIL04581.1 | guaA | GMP synthase | -0.01 | -0.04 | -0.12 | NC | NC | NC | 1 | F | ko:K01951 |
| AIL04718.1 | oppA1 | bacterial extracellular solute-binding s, 5 Middle family protein | -0.11 | -0.25 | -0.29 | NC | NC | NC | 1 | E | ko:K02035 |
| AIL03232.1 | guaB | inosine-5'-monophosphate dehydrogenase | 0.08 | -0.42 | -0.32 | NC | NC | NC | 1 | F | ko:K00088 |
| AIL04888.1 | gyrB | DNA gyrase, B subunit | -0.26 | -0.04 | -0.10 | NC | NC | NC | 1 | L | ko:K02470 |
| AIL03820.1 | ptsI | phosphoenolpyruvate-protein phosphotransferase | -0.31 | -0.15 | -0.22 | NC | NC | NC | 1 | G | ko:K08483 |
| AIL03290.1 | frdC | flavocytochrome c family protein | -0.21 | -0.22 | -0.23 | NC | NC | NC | 1 | C | ko:K00239 |
| AIL04770.1 | gshF | glutamate--cysteine ligase/gamma-glutamylcysteine synthetase | 0.29 | 0.25 | 0.16 | NC | NC | NC | 1 | F | ko:K01919 |
| AIL05933.1 | trePP | trehalose 6-phosphate phosphorylase | -0.06 | 0.66 | 0.49 | NC | NC | NC | 1 | G | ko:K00691 |
| AIL05855.1 | lysS | lysine--tRNA ligase | -0.21 | -0.25 | -0.17 | NC | NC | NC | 1 | J | ko:K04567 |
| AIL03755.1 | oppA | bacterial extracellular solute-binding s, 5 Middle family protein | 0.22 | -0.26 | -0.24 | NC | NC | NC | 1 | E | ko:K02035 |
| AIL04991.1 | topA | DNA topoisomerase I | -0.64 | -0.21 | -0.47 | NC | NC | NC | 1 | L | ko:K03168 |
| AIL05215.1 | proS | proline--tRNA ligase | -0.41 | -0.13 | -0.21 | NC | NC | NC | 1 | J | ko:K01881 |
| AIL03747.1 | - | glycosyl transferase 2 family protein | -0.82 | 0.07 | -0.04 | NC | NC | NC | 1 | M | ko:K19425 |
| AIL05259.1 | adh | aldehyde dehydrogenase family protein | -0.34 | 0.23 | -0.65 | NC | NC | NC | 1 | C | ko:K04072 |
| AIL05122.1 | rnjB | metallo-beta-lactamase superfamily protein | 0.20 | 0.03 | -0.01 | NC | NC | NC | 1 | J | ko:K12574 |
| AIL03691.1 | uvrB | excinuclease ABC subunit B | 0.49 | -0.06 | 0.14 | NC | NC | NC | 1 | L | ko:K03702 |
| AIL05403.1 | parC | DNA topoisomerase IV, A subunit | -0.66 | -0.21 | -0.16 | NC | NC | NC | 1 | L | ko:K02621 |
| AIL05258.1 | infB | translation initiation factor IF-2 | 0.15 | -0.47 | -0.45 | NC | NC | NC | 1 | J | ko:K02519 |
| AIL05734.1 | pip | hypothetical protein DR75_2797 | 0.78 | 0.08 | -0.13 | NC | NC | NC | 1 | S | ko:K01421 |
| AIL05010.1 | pgk | phosphoglycerate kinase | 0.46 | 0.47 | 0.34 | NC | NC | NC | 1 | F | ko:K00927 |
| AIL04721.1 | glnP | lysine-arginine-ornithine-binding periplasmic family protein | -0.20 | -0.15 | -0.29 | NC | NC | NC | 1 | P | ko:K02029 |
| AIL04003.1 | tig | trigger factor | -0.22 | -0.05 | -0.19 | NC | NC | NC | 1 | D | ko:K03545 |
| AIL04442.1 | glmS | glutamine-fructose-6-phosphate transaminase | -0.61 | -0.25 | -0.42 | NC | NC | NC | 1 | M | ko:K00820 |
| AIL04045.1 | aspS | aspartate--tRNA ligase | -0.23 | -0.12 | -0.12 | NC | NC | NC | 1 | J | ko:K01876 |
| AIL05169.1 | ykpA | ABC transporter family protein | -0.16 | -0.01 | 0.01 | NC | NC | NC | 1 | S | - |
| AIL04918.1 | purB | adenylosuccinate lyase | -0.15 | -0.03 | 0.02 | NC | NC | NC | 1 | F | ko:K01756 |
| AIL04477.1 | arcA | arginine deiminase | 0.57 | 0.36 | 0.11 | NC | NC | NC | 1 | E | ko:K01478 |
| AIL03384.1 | pyk | pyruvate kinase | 0.01 | 0.21 | -0.04 | NC | NC | NC | 1 | G | ko:K00873 |
| AIL04379.1 | malE | bacterial extracellular solute-binding family protein | 0.32 | 0.65 | 0.58 | NC | NC | NC | 1 | G | ko:K02027 |
| AIL05465.1 | oppA | bacterial extracellular solute-binding s, 5 Middle family protein | -0.13 | -0.06 | -0.08 | NC | NC | NC | 1 | E | ko:K02035 |
| AIL05435.1 | pheT | phenylalanine--tRNA ligase, beta subunit | -0.35 | 0.11 | 0.15 | NC | NC | NC | 1 | J | ko:K01890 |
| AIL05666.1 | pbp2b | penicillin binding transpeptidase domain protein | -0.33 | -0.54 | -0.50 | NC | NC | NC | 1 | M | ko:K00687 |
| AIL04516.1 | mutS | DNA mismatch repair protein MutS | 0.06 | -0.11 | 0.01 | NC | NC | NC | 1 | L | ko:K03555 |
| AIL04424.1 | ponA | penicillin-binding protein 1A | -0.19 | -0.17 | -0.21 | NC | NC | NC | 1 | M | ko:K05366 |
| AIL04210.1 | tuf | translation elongation factor Tu | -0.09 | -0.19 | -0.38 | NC | NC | NC | 1 | J | ko:K02358 |
| AIL04223.1 | pbp1B | transglycosylase family protein | -0.34 | -0.09 | -0.30 | NC | NC | NC | 1 | M | ko:K03693 |
| AIL04035.1 | gnd | 6-phosphogluconate dehydrogenase | -0.07 | 0.00 | 0.07 | NC | NC | NC | 1 | H | ko:K00033 |
| AIL03471.1 | thrS | threonine--tRNA ligase | -0.29 | -0.16 | -0.14 | NC | NC | NC | 1 | J | ko:K01868 |
| AIL03342.1 | tcsA | basic membrane family protein | 0.32 | 0.36 | 0.20 | NC | NC | NC | 1 | S | ko:K02058 |
| AIL04508.1 | - | bacterial cellulose synthase subunit | 0.12 | 0.18 | -0.08 | NC | NC | NC | 1 | S | - |
| AIL04584.1 | cshA | hypothetical protein DR75_2786 | -0.26 | -0.39 | -0.52 | NC | NC | NC | 1 | F | ko:K05592 |
| AIL05157.1 | sufB | FeS assembly protein SufB | 0.14 | 0.01 | 0.00 | NC | NC | NC | 1 | O | ko:K07033 |
| AIL03712.1 | pbpX | PASTA domain protein | 0.09 | -0.11 | -0.01 | NC | NC | NC | 1 | M | ko:K08724 |
| AIL03116.1 | merA | mercuric reductase (plasmid) | 0.94 | 0.04 | 0.03 | NC | NC | NC | 1 | C | ko:K00520 |
| AIL04161.1 | tkt | transketolase | -0.38 | 0.14 | 0.01 | NC | NC | NC | 1 | G | ko:K00615 |
| AIL03671.1 | pgm | phosphoglucomutase/phosphomannomutase, alpha/beta/alpha domain III family protein | -0.22 | 0.09 | 0.07 | NC | NC | NC | 1 | G | ko:K01835 |
| AIL04134.1 | eno | phosphopyruvate hydratase | -0.50 | -0.34 | -0.53 | NC | NC | NC | 1 | G | ko:K01689 |
| AIL03880.1 | pgi | phosphoglucose isomerase family protein | 0.12 | 0.04 | 0.03 | NC | NC | NC | 1 | G | ko:K01810 |
| AIL04816.1 | ftsZ | cell division protein FtsZ | -0.22 | -0.03 | -0.10 | NC | NC | NC | 1 | D | ko:K03531 |
| AIL04030.1 | yfmR | heme ABC exporter, ATP-binding protein CcmA | -0.64 | 0.21 | -0.13 | NC | NC | NC | 1 | S | ko:K15738 |
| AIL05859.1 | typA | GTP-binding protein TypA/BipA | -0.07 | 0.17 | -0.01 | NC | NC | NC | 1 | T | ko:K06207 |
| AIL03223.1 | purA | adenylosuccinate synthase | -0.05 | -0.06 | -0.08 | NC | NC | NC | 1 | F | ko:K01939 |
| AIL05162.1 | pncB | nicotinate phosphoribosyltransferase family protein | 0.12 | 0.04 | -0.03 | NC | NC | NC | 1 | F | ko:K00763 |
| AIL05848.1 | oadA | HMGL-like family protein | 0.60 | 0.49 | 0.18 | NC | NC | NC | 1 | C | ko:K01571 |
| AIL04476.1 | parE | DNA topoisomerase IV, B subunit | -0.65 | -0.08 | -0.11 | NC | NC | NC | 1 | L | ko:K02622 |
| AIL03277.1 | rumA_2 | 23S rRNA (uracil-5-)-methyltransferase RumA | -0.13 | -0.31 | -0.13 | NC | NC | NC | 1 | J | ko:K03215 |
| AIL04355.1 | tyrS | tyrosine--tRNA ligase | 0.23 | -0.29 | -0.11 | NC | NC | NC | 1 | J | ko:K01866 |
| AIL05279.1 | cysS | cysteine--tRNA ligase | -0.48 | -0.01 | -0.19 | NC | NC | NC | 1 | J | ko:K01883 |
| AIL04836.1 | leuS | leucine--tRNA ligase | -0.19 | 0.00 | 0.07 | NC | NC | NC | 1 | J | ko:K01869 |
| AIL05578.1 | mltG | yceG-like family protein | -0.37 | -0.17 | -0.23 | NC | NC | NC | 1 | S | ko:K07082 |
| AIL04984.1 | rny | ribonuclease Y | -0.18 | -0.09 | 0.00 | NC | NC | NC | 1 | S | ko:K18682 |
| AIL03351.1 | hslU | ATP-dependent protease HslVU, ATPase subunit | -0.45 | 0.06 | -0.10 | NC | NC | NC | 1 | O | ko:K03667 |
| AIL03938.1 | XK27_05795 | amino ABC transporter, permease, 3-TM region, His/Glu/Gln/Arg/opine family domain protein | 0.07 | -0.27 | -0.23 | NC | NC | NC | 1 | P | ko:K17073 |
| AIL03692.1 | levR | sigma-54 interaction domain protein | 0.05 | 0.09 | -0.14 | NC | NC | NC | 1 | K | - |
| AIL04699.1 | asnS | asparagine--tRNA ligase | -0.17 | -0.07 | -0.22 | NC | NC | NC | 1 | J | ko:K01893 |
| AIL05517.1 | glmM | phosphoglucosamine mutase | 0.11 | -0.01 | -0.04 | NC | NC | NC | 1 | G | ko:K03431 |
| AIL04906.1 | zwf | glucose-6-phosphate dehydrogenase | 0.43 | 0.00 | 0.00 | NC | NC | NC | 1 | G | ko:K00036 |
| AIL03726.1 | gltX | glutamate--tRNA ligase | 0.26 | 0.15 | 0.05 | NC | NC | NC | 1 | J | ko:K09698 |
| AIL04297.1 | ffh | signal recognition particle protein | -0.88 | -0.48 | -0.39 | NC | NC | NC | 1 | U | ko:K03106 |
| AIL05554.1 | - | hypothetical protein DR75_488 | 0.25 | 0.71 | 0.64 | NC | NC | NC | 1 | S | ko:K07504 |
| AIL04168.1 | fruA | PTS system, fructose subfamily, IIA component domain protein | 0.23 | 0.30 | 0.03 | NC | NC | NC | 1 | GT | ko:K02768 |
| AIL03365.1 | - | ABC transporter family protein | -0.04 | -0.40 | -0.34 | NC | NC | NC | 1 | V | ko:K02003 |
| AIL04845.1 | - | hypothetical protein DR75_1224 | -0.10 | -0.05 | -0.07 | NC | NC | NC | 1 | M | ko:K18149 |
| AIL05366.1 | deoB | phosphopentomutase | 0.79 | 0.66 | 0.34 | NC | NC | NC | 1 | G | ko:K01839 |
| AIL05913.1 | ntpB | V-type sodium ATPase subunit B | -0.09 | -0.16 | -0.05 | NC | NC | NC | 1 | C | ko:K02118 |
| AIL04571.1 | rpoA | DNA-directed RNA polymerase, alpha subunit | 0.03 | 0.08 | 0.02 | NC | NC | NC | 1 | K | ko:K03040 |
| AIL04204.1 | glnA | glutamine synthetase, type I | -0.58 | -0.06 | -0.09 | NC | NC | NC | 1 | E | ko:K01915 |
| AIL04288.1 | rpsA | hypothetical protein DR75_552 | -0.10 | 0.13 | -0.05 | NC | NC | NC | 1 | J | ko:K02945 |
| AIL03723.1 | ndh | FAD dependent oxidoreductase family protein | -0.35 | -0.68 | -0.49 | NC | NC | NC | 1 | C | ko:K03885 |
| AIL03661.1 | nox | NADH oxidase | 0.44 | 0.15 | 0.18 | NC | NC | NC | 1 | S | ko:K17869 |
| AIL03570.1 | der | ribosome-associated GTPase EngA | 0.12 | -0.07 | 0.03 | NC | NC | NC | 1 | S | ko:K03977 |
| AIL04914.1 | yaaN | toxic anion resistance family protein | -0.11 | -0.04 | -0.18 | NC | NC | NC | 1 | P | - |
| AIL04762.1 | lepA | GTP-binding protein LepA | -0.11 | -0.03 | -0.04 | NC | NC | NC | 1 | M | ko:K03596 |
| AIL03257.1 | ydiF | heme ABC exporter, ATP-binding protein CcmA | -0.11 | -0.21 | 0.11 | NC | NC | NC | 1 | S | ko:K06158 |
| AIL03317.1 | rnjA | hypothetical protein DR75_1618 | 0.08 | -0.28 | -0.16 | NC | NC | NC | 1 | J | ko:K12574 |
| AIL05377.1 | nusA | transcription termination factor NusA | -0.30 | -0.03 | -0.13 | NC | NC | NC | 1 | K | ko:K02600 |
| AIL04331.1 | ntpA | V-type sodium ATPase catalytic subunit A | -0.20 | -0.14 | -0.14 | NC | NC | NC | 1 | F | ko:K02117 |
| AIL04616.1 | obg | Obg family GTPase CgtA | -0.33 | -0.16 | -0.15 | NC | NC | NC | 1 | S | ko:K03979 |
| AIL03928.1 | mglA | heme ABC exporter, ATP-binding protein CcmA | -0.14 | 0.18 | 0.13 | NC | NC | NC | 1 | S | ko:K02056 |
| AIL03956.1 | lplA | lipoyltransferase and lipoate-ligase family protein | -0.12 | 0.11 | 0.00 | NC | NC | NC | 1 | H | ko:K03800 |
| AIL05475.1 | prfC | peptide chain release factor 3 | -0.50 | -0.39 | -0.34 | NC | NC | NC | 1 | J | ko:K02837 |
| AIL04972.1 | pepF | oligoendopeptidase F | -0.24 | 0.03 | 0.00 | NC | NC | NC | 1 | E | ko:K08602 |
| AIL05581.1 | glpK | glycerol kinase | -0.12 | 0.26 | 0.04 | NC | NC | NC | 1 | F | ko:K00864 |
| AIL03338.1 | gatA | aspartyl/glutamyl-tRNA(Asn/Gln) amidotransferase, A subunit | -0.05 | -0.02 | -0.07 | NC | NC | NC | 1 | F | ko:K02433 |
| AIL05550.1 | ctpE | HAD ATPase, P-type, IC family protein | -0.58 | 0.03 | 0.21 | NC | NC | NC | 1 | P | ko:K12952 |
| AIL03477.1 | mnmA | tRNA (5-methylaminomethyl-2-thiouridylate)-methyltransferase | 0.57 | -0.12 | -0.04 | NC | NC | NC | 1 | J | ko:K00566 |
| AIL05195.1 | femA | femAB family protein | -0.10 | -0.20 | -0.06 | NC | NC | NC | 1 | V | ko:K12554 |
| AIL03513.1 | tsf | translation elongation factor Ts | -0.07 | 0.08 | 0.08 | NC | NC | NC | 1 | J | ko:K02357 |
| AIL04896.1 | pdhD | dihydrolipoyl dehydrogenase | -0.66 | 0.26 | 0.18 | NC | NC | NC | 1 | C | ko:K00382 |
| AIL05398.1 | argF | ornithine carbamoyltransferase | 0.79 | 0.57 | 0.28 | NC | NC | NC | 1 | E | ko:K00611 |
| AIL05378.1 | hisS | histidine--tRNA ligase | -0.43 | -0.22 | -0.08 | NC | NC | NC | 1 | J | ko:K01892 |
| AIL03313.1 | clpX | ATP-dependent Clp protease, ATP-binding subunit ClpX | -0.20 | -0.27 | -0.37 | NC | NC | NC | 1 | O | ko:K03544 |
| AIL04358.1 | cfa | methyltransferase domain protein | 0.53 | 0.23 | 0.27 | NC | NC | NC | 1 | M | ko:K00574 |
| AIL05340.1 | pepS | aminopeptidase pepS | 0.23 | 0.18 | -0.03 | NC | NC | NC | 1 | E | ko:K19689 |
| AIL04261.1 | opuCA | glycine betaine/L-proline transport ATP binding subunit | -0.56 | 0.20 | 0.17 | NC | NC | NC | 1 | E | ko:K05847 |
| AIL05382.1 | atpD | ATP synthase F1, beta subunit | -0.25 | -0.11 | -0.13 | NC | NC | NC | 1 | C | ko:K02112 |
| AIL05739.1 | pdhC | dihydrolipoyllysine-residue acetyltransferase component of pyruvate dehydrogenase complex | 0.22 | 0.42 | 0.16 | NC | NC | NC | 1 | C | ko:K00627 |
| AIL03225.1 | mutL | DNA mismatch repair MutL family protein | 0.22 | -0.27 | -0.10 | NC | NC | NC | 1 | L | ko:K03572 |
| AIL03907.1 | atpA | ATP synthase F1, alpha subunit | -0.28 | -0.03 | -0.32 | NC | NC | NC | 1 | C | ko:K02111 |
| AIL05313.1 | glyA | beta-eliminating lyase family protein | 0.18 | 0.21 | 0.21 | NC | NC | NC | 1 | E | ko:K00600 |
| AIL03260.1 | - | glycosyl transferase 2 family protein | -0.26 | -0.03 | 0.00 | NC | NC | NC | 1 | M | ko:K20444 |
| AIL05576.1 | atoB | hydroxymethylglutaryl-CoA reductase, degradative | 0.08 | -0.19 | -0.02 | NC | NC | NC | 1 | I | ko:K00054 |
| AIL03697.1 | tagB | Poly(glycerophosphate) glycerophosphotransferase family protein | -0.22 | -0.27 | -0.41 | NC | NC | NC | 1 | M | ko:K09809 |
| AIL04254.1 | dnaJ | chaperone protein DnaJ | -0.22 | -0.22 | -0.16 | NC | NC | NC | 1 | O | ko:K03686 |
| AIL05192.1 | ftsA | cell division protein FtsA | -0.15 | -0.08 | -0.10 | NC | NC | NC | 1 | D | ko:K03590 |
| AIL03433.1 | pdhA | pyruvate dehydrogenase (acetyl-transferring) E1 component, alpha subunit | 0.29 | 0.39 | 0.19 | NC | NC | NC | 1 | C | ko:K00161 |
| AIL04725.1 | yumB | FAD dependent oxidoreductase family protein | -0.47 | -0.10 | -0.07 | NC | NC | NC | 1 | C | ko:K03885 |
| AIL05172.1 | ccpA | catabolite control protein A | -0.73 | -0.23 | -0.21 | NC | NC | NC | 1 | K | ko:K02529 |
| AIL04546.1 | tyrS | tyrosine--tRNA ligase | -0.35 | -0.09 | -0.04 | NC | NC | NC | 1 | J | ko:K01866 |
| AIL03919.1 | pepC | peptidase C1-like family protein | 0.02 | 0.17 | 0.15 | NC | NC | NC | 1 | E | ko:K01372 |
| AIL04352.1 | citF | citrate lyase, alpha subunit | 0.72 | 0.49 | 0.12 | NC | NC | NC | 1 | H | ko:K01643 |
| AIL03226.1 | sigA | RNA polymerase sigma factor RpoD | 0.11 | -0.15 | -0.17 | NC | NC | NC | 1 | K | ko:K03086 |
| AIL03455.1 | alsS | acetolactate synthase, catabolic | -0.52 | 0.63 | 0.43 | NC | NC | NC | 1 | EH | ko:K01652 |
| AIL05392.1 | sun | ribosomal RNA small subunit methyltransferase B | -0.38 | -0.20 | -0.16 | NC | NC | NC | 1 | J | ko:K03500 |
| AIL03890.1 | rarA | AAA ATPase, central region | 0.90 | 0.10 | -0.04 | NC | NC | NC | 1 | O | ko:K07478 |
| AIL05899.1 | cysK | cysteine synthase A | 0.77 | 0.49 | 0.49 | NC | NC | NC | 1 | E | ko:K01738 |
| AIL04024.1 | - | hypothetical protein DR75_2870 | -0.11 | -0.47 | 0.22 | NC | NC | NC | 1 | L | ko:K03657 |
| AIL04282.1 | murE | UDP-N-acetylmuramoyl-L-alanyl-D-glutamate--L-lysine ligase | 0.14 | 0.19 | 0.97 | NC | NC | NC | 1 | M | ko:K01928 |
| AIL04634.1 | gor | glutathione-disulfide reductase | 0.16 | -0.04 | -0.01 | NC | NC | NC | 1 | C | ko:K00383 |
| AIL05827.1 | trmFO | m(5)U-54 methyltransferase | 0.12 | -0.10 | -0.10 | NC | NC | NC | 1 | J | ko:K04094 |
| AIL03231.1 | fhs | formate--tetrahydrofolate ligase family protein | -0.09 | 0.17 | 0.10 | NC | NC | NC | 1 | F | ko:K01938 |
| AIL05502.1 | rph | ribonuclease PH | -0.02 | -0.10 | -0.18 | NC | NC | NC | 1 | F | ko:K00989 |
| AIL03483.1 | pmr1 | calcium-translocating P-type ATPase, PMCA-type | 0.17 | -0.07 | -0.36 | NC | NC | NC | 1 | P | ko:K01537 |
| AIL05624.1 | snf | DEAD/DEAH box helicase family protein | 0.04 | -0.02 | -0.19 | NC | NC | NC | 1 | KL | - |
| AIL03977.1 | dnaA | chromosomal replication initiator protein DnaA | 0.09 | 0.02 | -0.13 | NC | NC | NC | 1 | L | ko:K02313 |
| AIL03752.1 | thrC | threonine synthase | 0.22 | 0.04 | -0.01 | NC | NC | NC | 1 | E | ko:K01733 |
| AIL04549.1 | - | helix-turn-helix family protein | -0.01 | 0.06 | -0.07 | NC | NC | NC | 1 | K | ko:K20342 |
| AIL03396.1 | glmU | UDP-N-acetylglucosamine diphosphorylase/glucosamine-1-phosphate N-acetyltransferase | -0.10 | -0.22 | -0.24 | NC | NC | NC | 1 | M | ko:K04042 |
| AIL03675.1 | npr | NADH peroxidase | 0.42 | 0.53 | 0.27 | NC | NC | NC | 1 | S | ko:K05910 |
| AIL03510.1 | manL | PTS system, mannose/fructose/sorbose, IIB component family protein | -0.63 | 0.18 | 0.04 | NC | NC | NC | 1 | G | ko:K02769 |
| AIL04478.1 | divIVA | DivIVA domain protein | 0.40 | -0.08 | -0.01 | NC | NC | NC | 1 | D | ko:K04074 |
| AIL04976.1 | dnaX | DNA polymerase III, subunit gamma and tau | 0.09 | 0.25 | 0.13 | NC | NC | NC | 1 | L | ko:K02343 |
| AIL03494.1 | mnaA | UDP-N-acetylglucosamine 2-epimerase | 0.17 | 0.06 | 0.17 | NC | NC | NC | 1 | G | ko:K01791 |
| AIL03578.1 | aspC | aminotransferase class-V family protein | -0.28 | -0.14 | -0.12 | NC | NC | NC | 1 | E | ko:K14260 |
| AIL05370.1 | prkC | phosphotransferase enzyme family protein | -0.24 | -0.23 | -0.12 | NC | NC | NC | 1 | KLT | ko:K08884 |
| AIL03722.1 | gdhA | glu/Leu/Phe/Val dehydrogenase, dimerization domain protein | -0.48 | -0.14 | -0.06 | NC | NC | NC | 1 | E | ko:K00262 |
| AIL03230.1 | murA | UDP-N-acetylglucosamine 1-carboxyvinyltransferase | 0.00 | -0.35 | -0.29 | NC | NC | NC | 1 | M | ko:K00790 |
| AIL05654.1 | - | beta-ketoacyl-acyl-carrier-protein synthase II | -0.66 | 0.00 | -0.19 | NC | NC | NC | 1 | I | ko:K09458 |
| AIL05547.1 | pfkA | 6-phosphofructokinase | 0.10 | -0.01 | -0.07 | NC | NC | NC | 1 | F | ko:K00850 |
| AIL05078.1 | acmA | muramidase-2 | -0.28 | -0.29 | -0.53 | NC | NC | NC | 1 | MNU | ko:K02395 |
| AIL05691.1 | YSH1 | hypothetical protein DR75_1599 | 0.20 | 0.51 | 0.37 | NC | NC | NC | 1 | S | ko:K12574 |
| AIL03916.1 | pyrG | CTP synthase | -0.29 | -0.08 | -0.06 | NC | NC | NC | 1 | F | ko:K01937 |
| AIL04068.1 | ypwA | carboxypeptidase Taq (M32) metallopeptidase family protein | 0.17 | -0.18 | -0.11 | NC | NC | NC | 1 | E | ko:K01299 |
| AIL05085.1 | ftsK | DNA translocase ftsK | 0.05 | -0.26 | -0.37 | NC | NC | NC | 1 | D | ko:K03466 |
| AIL04687.1 | - | bacterial extracellular solute-binding family protein | -0.09 | 0.00 | -0.20 | NC | NC | NC | 1 | G | ko:K02027 |
| AIL05200.1 | uvrA2 | sigma-54 interaction domain protein | -0.08 | -0.07 | 0.47 | NC | NC | NC | 1 | L | - |
| AIL04040.1 | aha1 | HAD ATPase, P-type, IC family protein | 0.01 | -0.11 | -0.52 | NC | NC | NC | 1 | P | ko:K12952 |
| AIL05611.1 | ackA | acetate kinase | -0.39 | 0.14 | 0.13 | NC | NC | NC | 1 | F | ko:K00925 |
| AIL03587.1 | mnmE | tRNA modification GTPase TrmE | 1.00 | -0.22 | -0.04 | NC | NC | NC | 1 | S | ko:K03650 |
| AIL05637.1 | ychF | 50S ribosome-binding GTPase family protein | -0.61 | -0.49 | -0.36 | NC | NC | NC | 1 | J | ko:K06942 |
| AIL05003.1 | spo0J | stage 0 sporulation protein J | -0.31 | -0.14 | -0.42 | NC | NC | NC | 1 | K | ko:K03497 |
| AIL03613.1 | era | GTP-binding protein Era | 0.26 | 0.39 | 0.22 | NC | NC | NC | 1 | S | ko:K03595 |
| AIL03991.1 | deoA | pyrimidine-nucleoside phosphorylase | 0.10 | 0.20 | 0.36 | NC | NC | NC | 1 | F | ko:K00756 |
| AIL03120.1 | arsA | arsenical pump-driving ATPase (plasmid) | 0.10 | -0.44 | -0.24 | NC | NC | NC | 1 | D | ko:K01551 |
| AIL03158.1 | traB | TraB family protein (plasmid) | 0.90 | -0.12 | -0.30 | NC | NC | NC | 1 | S | - |
| AIL05608.1 | acmB | mannosyl-glycoendo-beta-N-acetylglucosaminidase family protein | -0.58 | -0.71 | -0.85 | NC | NC | NC | 1 | NU | ko:K01227 |
| AIL04195.1 | oppD | hypothetical protein DR75_2847 | -0.48 | -0.19 | -0.20 | NC | NC | NC | 1 | P | ko:K02031 |
| AIL05731.1 | dnaN | DNA polymerase III, beta subunit | 0.27 | 0.07 | 0.09 | NC | NC | NC | 1 | L | ko:K02338 |
| AIL04327.1 | tcsA | basic membrane family protein | 0.02 | 0.04 | -0.13 | NC | NC | NC | 1 | S | ko:K02058 |
| AIL05174.1 | glcK | ROK family protein | -0.18 | -0.18 | -0.04 | NC | NC | NC | 1 | G | ko:K00845 |
| AIL04286.1 | hpaIIM | modification methylase BspRI | -0.28 | -0.31 | -0.35 | NC | NC | NC | 1 | L | ko:K00558 |
| AIL03285.1 | strH | glycosyl hydrolase family 20, catalytic domain protein | 0.04 | 0.08 | 0.35 | NC | NC | NC | 1 | G | ko:K12373 |
| AIL03539.1 | sbcC | exonuclease SbcCD, C subunit | -0.07 | 0.15 | 0.34 | NC | NC | NC | 1 | L | ko:K03546 |
| AIL05845.1 | pepV | dipeptidase PepV | 0.32 | 0.26 | 0.25 | NC | NC | NC | 1 | E | ko:K01270 |
| AIL04375.1 | ecfA2 | ABC transporter family protein | -0.17 | 0.00 | 0.12 | NC | NC | NC | 1 | P | ko:K16787 |
| AIL04300.1 | rplE | 50S ribosomal protein L5 | -0.21 | -0.22 | -0.28 | NC | NC | NC | 1 | J | ko:K02931 |
| AIL03469.1 | aroC | chorismate synthase | -0.63 | -0.61 | -0.66 | NC | NC | NC | 1 | E | ko:K01736 |
| AIL04649.1 | sufD | FeS assembly protein SufD | 0.08 | 0.19 | -0.09 | NC | NC | NC | 1 | O | ko:K07033 |
| AIL04534.1 | rpsD | ribosomal protein S4 | -0.29 | -0.18 | -0.34 | NC | NC | NC | 1 | J | ko:K02986 |
| AIL05356.1 | rfbB | dTDP-glucose 4,6-dehydratase | -0.13 | -0.16 | -0.26 | NC | NC | NC | 1 | M | ko:K01710 |
| AIL04608.1 | cshB | hypothetical protein DR75_451 | 0.01 | 0.15 | -0.56 | NC | NC | NC | 1 | JKL | ko:K05592 |
| AIL04614.1 | ycjM | hypothetical protein DR75_1597 | -0.35 | 0.21 | 0.00 | NC | NC | NC | 1 | F | ko:K01119 |
| AIL05477.1 | pbp2A | penicillin-binding, 1A family protein | 0.19 | -0.15 | -0.07 | NC | NC | NC | 1 | M | ko:K12555 |
| AIL04985.1 | prfA | peptide chain release factor 1 | -0.56 | -0.49 | -0.21 | NC | NC | NC | 1 | J | ko:K02835 |
| AIL04646.1 | nfo | putative endonuclease 4 | 0.39 | 0.02 | -0.05 | NC | NC | NC | 1 | L | ko:K01151 |
| AIL04175.1 | dnaG | DNA primase | -0.41 | -0.32 | -0.06 | NC | NC | NC | 1 | K | ko:K02316 |
| AIL04524.1 | folC | bifunctional FolC family protein | 0.35 | 0.01 | 0.03 | NC | NC | NC | 1 | H | ko:K11754 |
| AIL04416.1 | lmrA | ABC transporter family protein | -0.18 | 0.14 | 0.02 | NC | NC | NC | 1 | V | ko:K06147 |
| AIL04472.1 | dnaE | DNA polymerase III, alpha subunit | -0.09 | 0.26 | 0.15 | NC | NC | NC | 1 | L | ko:K02337 |
| AIL04971.1 | - | glyoxalase/Bleomycin resistance /Dioxygenase superfamily protein | -0.34 | 0.01 | -0.06 | NC | NC | NC | 1 | S | ko:K07104 |
| AIL05243.1 | thiD | hydroxyethylthiazole kinase family protein | -0.46 | -0.43 | -0.55 | NC | NC | NC | 1 | H | ko:K00868 |
| AIL03376.1 | manA | mannose-6-phosphate isomerase, class I | 0.02 | 0.35 | 0.15 | NC | NC | NC | 1 | G | ko:K01809 |
| AIL05573.1 | prs | ribose-phosphate pyrophosphokinase | -0.58 | -0.14 | -0.25 | NC | NC | NC | 1 | F | ko:K00948 |
| AIL04296.1 | nylA | amidase family protein | 0.43 | 0.23 | 0.31 | NC | NC | NC | 1 | J | ko:K01426 |
| AIL04764.1 | recJ | single-stranded-DNA-specific exonuclease RecJ | -0.18 | -0.38 | 0.15 | NC | NC | NC | 1 | L | ko:K07462 |
| AIL05598.1 | pheS | phenylalanine--tRNA ligase, alpha subunit | -0.51 | -0.11 | -0.18 | NC | NC | NC | 1 | J | ko:K01889 |
| AIL05763.1 | rpsB | ribosomal protein S2 | -0.54 | -0.16 | -0.43 | NC | NC | NC | 1 | J | ko:K02967 |
| AIL05090.1 | metK | methionine adenosyltransferase | 0.66 | 0.35 | 0.10 | NC | NC | NC | 1 | H | ko:K00789 |
| AIL03622.1 | algC | phosphoglucomutase/phosphomannomutase, alpha/beta/alpha domain III family protein | -0.24 | 0.29 | 0.19 | NC | NC | NC | 1 | G | ko:K01840 |
| AIL04894.1 | - | femAB family protein | -0.84 | -0.44 | -0.51 | NC | NC | NC | 1 | V | ko:K05363 |
| AIL04810.1 | XK27_09600 | ABC transporter family protein | -0.78 | 0.29 | -0.89 | NC | NC | NC | 1 | V | ko:K06147 |
| AIL03386.1 | codY | GTP-sensing transcriptional pleiotropic repressor CodY | -0.09 | 0.18 | 0.04 | NC | NC | NC | 1 | K | ko:K03706 |
| AIL05350.1 | serS | serine--tRNA ligase | 0.05 | 0.07 | 0.02 | NC | NC | NC | 1 | J | ko:K01875 |
| AIL04419.1 | nagE | PTS system, N-acetylglucosamine-specific IIBC component | 0.60 | 0.30 | 0.27 | NC | NC | NC | 1 | G | ko:K02802 |
| AIL03248.1 | ytoI | thioesterase superfamily protein | -0.12 | 0.20 | 0.00 | NC | NC | NC | 1 | K | - |
| AIL04170.1 | rplA | ribosomal protein L1 | -0.23 | -0.03 | -0.13 | NC | NC | NC | 1 | J | ko:K02863 |
| AIL05738.1 | mgtA | magnesium-translocating P-type ATPase | 0.20 | 0.18 | -0.21 | NC | NC | NC | 1 | P | ko:K01531 |
| AIL03624.1 | ykoD | heme ABC exporter, ATP-binding protein CcmA | -0.51 | -0.25 | -0.15 | NC | NC | NC | 1 | P | ko:K16786 |
| AIL03886.1 | albE | hypothetical protein DR75_1869 | 0.22 | 0.41 | 0.12 | NC | NC | NC | 1 | S | - |
| AIL04963.1 | - | acetyltransferase domain protein | 0.57 | 0.31 | 0.27 | NC | NC | NC | 1 | S | - |
| AIL03256.1 | - | mga helix-turn-helix domain protein | 0.91 | 0.34 | 0.41 | NC | NC | NC | 1 | K | - |
| AIL05028.1 | thiI | tRNA sulfurtransferase ThiI | -0.26 | -0.45 | 0.31 | NC | NC | NC | 1 | H | ko:K03151 |
| AIL05671.1 | xseA | exodeoxyribonuclease VII, large subunit | -0.97 | 0.16 | -0.36 | NC | NC | NC | 1 | L | ko:K03601 |
| AIL05804.1 | pepT | peptidase T | -0.13 | 0.09 | 0.09 | NC | NC | NC | 1 | E | ko:K01258 |
| AIL04411.1 | ywfO | HD domain protein | 0.08 | -0.06 | 0.01 | NC | NC | NC | 1 | S | ko:K06885 |
| AIL03883.1 | prsA | PPIC-type PPIASE domain protein | -0.17 | -0.19 | -0.45 | NC | NC | NC | 1 | M | ko:K01802 |
| AIL05478.1 | rplB | ribosomal protein L2 | -0.15 | -0.48 | -0.39 | NC | NC | NC | 1 | J | ko:K02886 |
| AIL04647.1 | yqfF | hypothetical protein DR75_1160 | -0.02 | 0.38 | 0.01 | NC | NC | NC | 1 | S | ko:K07037 |
| AIL05204.1 | plsX | fatty acid/phospholipid synthesis protein PlsX | 0.15 | -0.26 | -0.13 | NC | NC | NC | 1 | I | ko:K03621 |
| AIL05520.1 | alr | alanine racemase | 0.15 | -0.07 | 0.03 | NC | NC | NC | 1 | M | ko:K01775 |
| AIL03330.1 | - | hypothetical protein DR75_442 | 0.73 | 0.37 | 0.36 | NC | NC | NC | 1 | S | - |
| AIL03716.1 | asp1 | response regulator | 0.82 | 0.10 | 0.05 | NC | NC | NC | 1 | S | - |
| AIL03319.1 | murD | UDP-N-acetylmuramoylalanine--D-glutamate ligase | -0.42 | -0.12 | -0.11 | NC | NC | NC | 1 | M | ko:K01925 |
| AIL04338.1 | - | double zinc ribbon family protein | -0.12 | 0.41 | 0.40 | NC | NC | NC | 1 | S | - |
| AIL05353.1 | tgt | queuine tRNA-ribosyltransferase | -0.42 | -0.20 | -0.25 | NC | NC | NC | 1 | F | ko:K00773 |
| AIL05351.1 | murC | UDP-N-acetylmuramate--alanine ligase | 0.25 | -0.33 | -0.12 | NC | NC | NC | 1 | M | ko:K01924 |
| AIL05416.1 | msbA_1 | ABC transporter family protein | 0.01 | 0.17 | -0.06 | NC | NC | NC | 1 | V | ko:K18887 |
| AIL04199.1 | fabD | malonyl CoA-acyl carrier protein transacylase | -0.51 | 0.10 | 0.09 | NC | NC | NC | 1 | I | ko:K00645 |
| AIL03630.1 | - | shikimate / quinate 5-dehydrogenase family protein | 0.36 | -0.42 | -0.32 | NC | NC | NC | 1 | E | ko:K01750 |
| AIL03350.1 | bfmBAB | 2-oxoisovalerate dehydrogenase subunit beta | -0.42 | 0.43 | 0.44 | NC | NC | NC | 1 | C | ko:K00167 |
| AIL04166.1 | menF | isochorismate synthase family protein | -0.18 | -0.12 | -0.16 | NC | NC | NC | 1 | HQ | ko:K02552 |
| AIL03179.1 | - | putative nucleotidyltransferase (plasmid) | 0.25 | 0.06 | 0.17 | NC | NC | NC | 1 | J | - |
| AIL05217.1 | - | recT family protein | 0.98 | -0.43 | -0.13 | NC | NC | NC | 1 | L | ko:K07455 |
| AIL05322.1 | copB | copper-translocating P-type ATPase | 0.82 | 0.00 | -0.06 | NC | NC | NC | 1 | P | ko:K01533 |
| AIL04082.1 | pgl | lactonase, 7-bladed beta-propeller family protein | 0.06 | 0.49 | 0.23 | NC | NC | NC | 1 | G | ko:K07404 |
| AIL05705.1 | - | hypothetical protein DR75_298 | -0.15 | 0.00 | -0.12 | NC | NC | NC | 1 | G | ko:K17318 |
| AIL03861.1 | dnaI | istB-like ATP binding family protein | 0.01 | -0.34 | -0.11 | NC | NC | NC | 1 | L | ko:K11144 |
| AIL04860.1 | yqfO | NIF3 family protein | -0.18 | 0.26 | -0.11 | NC | NC | NC | 1 | S | - |
| AIL04304.1 | recN | DNA repair protein RecN | -0.31 | 0.09 | 0.04 | NC | NC | NC | 1 | L | ko:K03631 |
| AIL04228.1 | hrcA | heat-inducible transcription repressor HrcA | 0.52 | 0.44 | 0.01 | NC | NC | NC | 1 | K | ko:K03705 |
| AIL03618.1 | bglC | glycosyl hydrolase 1 family protein | 0.34 | 0.55 | 0.38 | NC | NC | NC | 1 | G | ko:K01223 |
| AIL05184.1 | araT | beta-eliminating lyase family protein | -0.51 | -0.35 | -0.32 | NC | NC | NC | 1 | E | ko:K00841 |
| AIL05326.1 | - | amidase family protein | 0.06 | -0.11 | -0.22 | NC | NC | NC | 1 | J | ko:K01426 |
| AIL05511.1 | pimB | glycosyl transferases group 1 family protein | 0.20 | 0.12 | -0.36 | NC | NC | NC | 1 | M | ko:K19002 |
| AIL04435.1 | dnaB | replicative DNA helicase | 0.78 | 0.48 | 0.22 | NC | NC | NC | 1 | L | ko:K02314 |
| AIL05783.1 | XK27_09615 | NADPH-dependent FMN reductase family protein | 0.70 | -0.58 | -0.38 | NC | NC | NC | 1 | S | ko:K19784 |
| AIL03462.1 | ytfP | flavo, family protein | 0.24 | -0.04 | 0.17 | NC | NC | NC | 1 | S | ko:K07007 |
| AIL05164.1 | yrrO | peptidase U32 family protein | -0.63 | -0.62 | -0.50 | NC | NC | NC | 1 | O | ko:K08303 |
| AIL03935.1 | ftsY | signal recognition particle-docking protein FtsY | 0.09 | -0.36 | -0.08 | NC | NC | NC | 1 | U | ko:K03110 |
| AIL03654.1 | atpG | ATP synthase F1, gamma subunit | -0.45 | -0.52 | -0.35 | NC | NC | NC | 1 | C | ko:K02115 |
| AIL05264.1 | rpsC | ribosomal protein S3 | -0.11 | 0.21 | -0.24 | NC | NC | NC | 1 | J | ko:K02982 |
| AIL04560.1 | aroK | shikimate kinase family protein | -0.99 | -0.40 | -0.51 | NC | NC | NC | 1 | F | ko:K00891 |
| AIL03385.1 | msrR | regulatory msrR domain protein | -0.66 | -0.56 | -0.33 | NC | NC | NC | 1 | K | - |
| AIL05052.1 | citE | citrate (pro-3S)-lyase, beta subunit | 0.66 | 0.47 | 0.14 | NC | NC | NC | 1 | H | ko:K01644 |
| AIL03284.1 | pepA | glutamyl aminopeptidase | 0.44 | 0.12 | 0.08 | NC | NC | NC | 1 | E | ko:K01261 |
| AIL04425.1 | adk | adenylate kinase | -0.31 | -0.10 | -0.14 | NC | NC | NC | 1 | F | ko:K00939 |
| AIL05594.1 | yqeH | ribosome biogenesis GTPase YqeH | -0.08 | -0.17 | -0.29 | NC | NC | NC | 1 | S | ko:K06948 |
| AIL04526.1 | - | hypothetical protein DR75_877 | -0.54 | 0.08 | -0.14 | NC | NC | NC | 1 | - | - |
| AIL04706.1 | pepQ | xaa-Pro dipeptidase | 0.25 | 0.25 | 0.06 | NC | NC | NC | 1 | E | ko:K01271 |
| AIL04657.1 | pdhB | pyruvate dehydrogenase E1 component subunit beta | 0.50 | 0.47 | 0.29 | NC | NC | NC | 1 | C | ko:K00162 |
| AIL05847.1 | panE | 2-dehydropantoate 2-reductase family protein | -0.30 | 0.63 | 0.54 | NC | NC | NC | 1 | H | ko:K00077 |
| AIL04276.1 | malL | alpha amylase, catalytic domain protein | -0.75 | -0.25 | -0.48 | NC | NC | NC | 1 | G | ko:K01182 |
| AIL03879.1 | phoH | phoH-like family protein | -0.71 | -0.23 | -0.25 | NC | NC | NC | 1 | T | ko:K06217 |
| AIL03972.1 | - | hypothetical protein DR75_308 | 0.13 | 0.10 | 0.16 | NC | NC | NC | 1 | S | - |
| AIL03242.1 | rseP | RIP metalloprotease RseP | -0.19 | -0.14 | -0.23 | NC | NC | NC | 1 | M | ko:K11749 |
| AIL04085.1 | uvrC | excinuclease ABC subunit C | 0.41 | -0.23 | -0.19 | NC | NC | NC | 1 | L | ko:K03703 |
| AIL04848.1 | hsdM | type I restriction-modification system, M subunit | -0.20 | -0.03 | 0.08 | NC | NC | NC | 1 | V | ko:K03427 |
| AIL04905.1 | - | LPXTG cell wall anchor domain protein | 0.03 | 0.55 | 0.33 | NC | NC | NC | 1 | F | ko:K01081 |
| AIL03850.1 | bglC | aryl-phospho-beta-D-glucosidase BglC | 0.27 | 0.26 | 0.52 | NC | NC | NC | 1 | G | ko:K01223 |
| AIL03429.1 | yybT | DHH family protein | 0.15 | 0.32 | -0.08 | NC | NC | NC | 1 | T | - |
| AIL04651.1 | arlS | HAMP domain protein | -0.46 | -0.51 | -0.61 | NC | NC | NC | 1 | T | ko:K18940 |
| AIL05856.1 | galE | UDP-glucose 4-epimerase GalE | -0.05 | -0.61 | -0.34 | NC | NC | NC | 1 | M | ko:K01784 |
| AIL05917.1 | fmt | methionyl-tRNA formyltransferase | 0.07 | -0.12 | -0.08 | NC | NC | NC | 1 | J | ko:K00604 |
| AIL04207.1 | rplC | 50S ribosomal protein L3 | -0.11 | 0.04 | -0.38 | NC | NC | NC | 1 | J | ko:K02906 |
| AIL03796.1 | rnc | ribonuclease III | -0.37 | -0.04 | -0.25 | NC | NC | NC | 1 | J | ko:K03685 |
| AIL04028.1 | yitL | S1 domain protein | 0.72 | 0.35 | 0.34 | NC | NC | NC | 1 | S | ko:K00243 |
| AIL03131.1 | soj | cobQ/CobB/MinD/ParA nucleotide binding domain protein (plasmid) | 0.00 | -0.34 | -0.14 | NC | NC | NC | 1 | D | - |
| AIL05568.1 | prs2 | ribose-phosphate pyrophosphokinase 2 | -0.35 | -0.12 | 0.00 | NC | NC | NC | 1 | F | ko:K00948 |
| AIL03551.1 | ldh | L-lactate dehydrogenase | 0.94 | 0.37 | 0.11 | NC | NC | NC | 1 | C | ko:K00016 |
| AIL04258.1 | murB | UDP-N-acetylenolpyruvoylglucosamine reductase | -0.58 | -0.06 | -0.16 | NC | NC | NC | 1 | M | ko:K00075 |
| AIL05485.1 | dltA | D-alanine--poly(phosphoribitol) ligase, subunit 1 | -0.09 | -0.04 | 0.05 | NC | NC | NC | 1 | Q | ko:K03367 |
| AIL04554.1 | murE2 | mur ligase middle domain protein | -0.01 | 0.03 | 0.16 | NC | NC | NC | 1 | M | ko:K01928 |
| AIL05567.1 | yycF | transcriptional regulatory protein yycF | 0.46 | 0.13 | 0.29 | NC | NC | NC | 1 | K | ko:K07668 |
| AIL05015.1 | oppF | ABC transporter family protein | -0.25 | -0.39 | -0.39 | NC | NC | NC | 1 | E | ko:K02032 |
| AIL03791.1 | rnz | ribonuclease Z | -0.15 | -0.22 | -0.24 | NC | NC | NC | 1 | S | ko:K00784 |
| AIL04921.1 | bdhA | NADH-dependent butanol dehydrogenase A | 0.72 | 0.20 | 0.23 | NC | NC | NC | 1 | C | - |
| AIL05489.1 | XK27_09605 | ABC transporter family protein | -0.11 | 0.52 | 0.04 | NC | NC | NC | 1 | V | ko:K18892 |
| AIL05773.1 | rluB | pseudouridine synthase family protein | 0.03 | 0.42 | -0.17 | NC | NC | NC | 1 | J | ko:K06178 |
| AIL03468.1 | aspB | aminotransferase class I and II family protein | -0.06 | -0.07 | -0.11 | NC | NC | NC | 1 | E | ko:K00812 |
| AIL04189.1 | gap | glyceraldehyde-3-phosphate dehydrogenase, type I | -0.43 | 0.11 | -0.05 | NC | NC | NC | 1 | G | ko:K00134 |
| AIL03868.1 | ypsC | THUMP domain protein | -0.07 | 0.11 | 0.09 | NC | NC | NC | 1 | L | ko:K07444 |
| AIL05559.1 | ftsE | cell division ATP-binding protein FtsE | 0.22 | 0.07 | -0.08 | NC | NC | NC | 1 | D | ko:K09812 |
| AIL05018.1 | XK27_08635 | hypothetical protein DR75_2541 | -0.79 | -0.07 | -0.12 | NC | NC | NC | 1 | S | ko:K09157 |
| AIL04255.1 | opuCC | substrate binding domain of ABC-type glycine betaine transport system family protein | -0.69 | -0.15 | -0.08 | NC | NC | NC | 1 | M | ko:K05845 |
| AIL03527.1 | - | D-isomer specific 2-hydroxyacid dehydrogenase, NAD binding domain protein | 0.20 | -0.42 | 0.00 | NC | NC | NC | 1 | CH | - |
| AIL04492.1 | ytxK | N-6 DNA Methylase family protein | -0.70 | -0.14 | -0.20 | NC | NC | NC | 1 | L | ko:K00571 |
| AIL04724.1 | - | cysteine-rich secretory family protein | -0.53 | -0.45 | -0.45 | NC | NC | NC | 1 | S | ko:K21471 |
| AIL03921.1 | murF | UDP-N-acetylmuramoyl-tripeptide--D-alanyl-D-alanine ligase family protein | -0.34 | -0.31 | -0.01 | NC | NC | NC | 1 | M | ko:K01929 |
| AIL04877.1 | pstB2 | phosphate ABC transporter, ATP-binding protein | -0.55 | -0.15 | -0.06 | NC | NC | NC | 1 | P | ko:K02036 |
| AIL04158.1 | psaA | manganese ABC transporter substrate-binding lipoprotein | -0.27 | -0.44 | -0.38 | NC | NC | NC | 1 | P | ko:K11704 |
| AIL03647.1 | - | phage portal, SPP1 Gp6-like family protein | 0.03 | -0.26 | 0.00 | NC | NC | NC | 1 | S | - |
| AIL04811.1 | - | peptidase, ArgE/DapE family protein | 0.83 | 0.47 | 0.32 | NC | NC | NC | 1 | E | ko:K01439 |
| AIL05361.1 | dus | dihydrouridine synthase family protein | 0.67 | 0.09 | 0.19 | NC | NC | NC | 1 | J | - |
| AIL03425.1 | fabI | enoyl-[acyl-carrier-protein] reductase [NADH] | -0.16 | 0.23 | 0.01 | NC | NC | NC | 1 | I | ko:K00208 |
| AIL04752.1 | oppF | ABC transporter family protein | -0.77 | -0.43 | -0.43 | NC | NC | NC | 1 | E | ko:K10823 |
| AIL04872.1 | ntpC | V-type sodium ATPase subunit C | 0.49 | 0.23 | -0.40 | NC | NC | NC | 1 | C | ko:K02119 |
| AIL05692.1 | yhaM | OB-fold nucleic acid binding domain protein | 0.57 | 0.07 | 0.01 | NC | NC | NC | 1 | S | ko:K03698 |
| AIL04995.1 | pstB1 | phosphate ABC transporter, ATP-binding protein | -0.20 | -0.14 | -0.09 | NC | NC | NC | 1 | P | ko:K02036 |
| AIL05373.1 | ywjA | ABC transporter family protein | 0.51 | 0.38 | 0.16 | NC | NC | NC | 1 | V | ko:K06147 |
| AIL03887.1 | gmuD | 6-phospho-beta-glucosidase gmuD | 0.29 | 0.35 | 0.23 | NC | NC | NC | 1 | G | ko:K01223 |
| AIL04060.1 | rfbD | dTDP-4-dehydrorhamnose reductase | -0.24 | -0.03 | -0.11 | NC | NC | NC | 1 | M | ko:K00067 |
| AIL04132.1 | hepT | polyprenyl synthetase family protein | 0.23 | -0.20 | -0.08 | NC | NC | NC | 1 | H | ko:K00805 |
| AIL05249.1 | cls | cardiolipin synthase | -0.01 | 0.18 | -0.32 | NC | NC | NC | 1 | I | ko:K06131 |
| AIL05390.1 | rnr | ribonuclease R | -0.40 | -0.14 | -0.15 | NC | NC | NC | 1 | J | ko:K12573 |
| AIL04575.1 | rex | DNA-binding family protein | 0.39 | 0.32 | 0.14 | NC | NC | NC | 1 | K | ko:K01926 |
| AIL03784.1 | yvcJ | P-loop ATPase family protein | 0.64 | 0.01 | 0.03 | NC | NC | NC | 1 | S | ko:K06958 |
| AIL05849.1 | gpmA | phosphoglycerate mutase 1 family protein | 0.20 | 0.17 | 0.14 | NC | NC | NC | 1 | G | ko:K01834 |
| AIL05715.1 | proA | glutamate-5-semialdehyde dehydrogenase | 0.42 | 0.05 | 0.15 | NC | NC | NC | 1 | E | ko:K00147 |
| AIL05271.1 | galU | UTP-glucose-1-phosphate uridylyltransferase | -0.09 | -0.22 | -0.31 | NC | NC | NC | 1 | M | ko:K00963 |
| AIL03830.1 | yvcK | hypothetical protein DR75_2720 | 0.51 | 0.05 | 0.17 | NC | NC | NC | 1 | S | - |
| AIL04831.1 | - | putative secreted lipase | -0.54 | -0.61 | -0.73 | NC | NC | NC | 1 | D | ko:K21471 |
| AIL04797.1 | ddl | D-alanine--D-alanine ligase family protein | -0.09 | 0.16 | 0.06 | NC | NC | NC | 1 | F | ko:K01921 |
| AIL03705.1 | pepQ | metallopeptidase M24 family protein | 0.17 | 0.23 | 0.14 | NC | NC | NC | 1 | E | ko:K01262 |
| AIL03817.1 | rpiA | ribose 5-phosphate isomerase A | -0.28 | 0.13 | -0.10 | NC | NC | NC | 1 | G | ko:K01807 |
| AIL05262.1 | citR | bacterial regulatory s, gntR family protein | 0.41 | 0.62 | 0.51 | NC | NC | NC | 1 | K | - |
| AIL04315.1 | thyA | thymidylate synthase | -0.62 | -0.11 | -0.37 | NC | NC | NC | 1 | F | ko:K00560 |
| AIL05733.1 | hslO | 33 kDa chaperonin | 0.00 | -0.23 | -0.31 | NC | NC | NC | 1 | O | ko:K04083 |
| AIL04452.1 | asd | aspartate-semialdehyde dehydrogenase | 0.20 | -0.15 | 0.06 | NC | NC | NC | 1 | E | ko:K00133 |
| AIL05506.1 | degV | EDD, DegV family domain protein | -0.14 | 0.03 | 0.04 | NC | NC | NC | 1 | K | - |
| AIL05147.1 | - | hypothetical protein DR75_707 | 0.21 | 0.90 | 0.28 | NC | NC | NC | 1 | S | ko:K09704 |
| AIL04874.1 | malK | ABC transporter family protein | -0.79 | 0.00 | 0.09 | NC | NC | NC | 1 | P | ko:K10112 |
| AIL03932.1 | panE | 2-dehydropantoate 2-reductase family protein | -0.82 | -0.74 | -0.55 | NC | NC | NC | 1 | H | ko:K00077 |
| AIL03586.1 | mhqA | glyoxalase/Bleomycin resistance /Dioxygenase superfamily protein | 0.84 | 0.38 | 0.10 | NC | NC | NC | 1 | E | ko:K15975 |
| AIL05619.1 | mocA | oxidoreductase, NAD-binding Rossmann fold family protein | 0.79 | 0.25 | 0.32 | NC | NC | NC | 1 | S | - |
| AIL05472.1 | htrA | PDZ domain protein | 0.90 | 0.17 | 0.17 | NC | NC | NC | 1 | O | ko:K04771 |
| AIL05490.1 | - | DNA gyrase B family protein | -0.01 | 0.11 | 0.14 | NC | NC | NC | 1 | L | - |
| AIL03288.1 | prfB | peptide chain release factor 2 | -0.29 | -0.40 | -0.34 | NC | NC | NC | 1 | J | ko:K02836 |
| AIL05587.1 | asp23 | response regulator | 0.83 | 0.24 | 0.10 | NC | NC | NC | 1 | S | - |
| AIL05031.1 | rgpD | ABC transporter family protein | -0.19 | -0.37 | -0.24 | NC | NC | NC | 1 | GM | ko:K01990 |
| AIL04887.1 | glxR | NAD binding domain of 6-phosphogluconate dehydrogenase family protein | 0.13 | 0.23 | 0.15 | NC | NC | NC | 1 | I | ko:K00020 |
| AIL05527.1 | rpsE | ribosomal protein S5 | -0.05 | 0.07 | -0.09 | NC | NC | NC | 1 | J | ko:K02988 |
| AIL05833.1 | mvaK2 | phosphomevalonate kinase | -0.41 | -0.46 | -0.33 | NC | NC | NC | 1 | I | ko:K00938 |
| AIL04061.1 | yqjQ | short chain dehydrogenase family protein | 0.00 | -0.11 | -0.23 | NC | NC | NC | 1 | S | ko:K07124 |
| AIL04111.1 | - | ngoPII restriction endonuclease family protein | 0.00 | -0.03 | -0.52 | NC | NC | NC | 1 | L | - |
| AIL03543.1 | - | amidohydrolase family protein | 0.15 | 0.36 | 0.17 | NC | NC | NC | 1 | F | - |
| AIL03333.1 | rrmJ | hemolysin TlyA family protein | 0.21 | 0.33 | 0.14 | NC | NC | NC | 1 | J | ko:K06442 |
| AIL04064.1 | accD | acetyl-CoA carboxylase, carboxyl transferase, beta subunit | -0.05 | 0.04 | -0.13 | NC | NC | NC | 1 | I | ko:K01963 |
| AIL03579.1 | glyQ | glycine--tRNA ligase, alpha subunit | -0.45 | -0.19 | -0.03 | NC | NC | NC | 1 | J | ko:K01878 |
| AIL03865.1 | coaA | pantothenate kinase | -0.42 | -0.96 | -0.15 | NC | NC | NC | 1 | F | ko:K00867 |
| AIL05775.1 | - | adenylate and Guanylate cyclase catalytic domain protein | 0.47 | 0.33 | 0.60 | NC | NC | NC | 1 | T | ko:K01768 |
| AIL05716.1 | potA | polyamine ABC transporter, ATP-binding family protein | -0.66 | -0.48 | -0.59 | NC | NC | NC | 1 | E | ko:K02010 |
| AIL05577.1 | - | dipeptidase family protein | 0.64 | 0.50 | 0.49 | NC | NC | NC | 1 | E | - |
| AIL04405.1 | - | hypothetical protein DR75_529 | 0.31 | 0.27 | -0.19 | NC | NC | NC | 1 | D | ko:K20073 |
| AIL03439.1 | phoR | sensory box protein | -0.18 | 0.33 | 0.17 | NC | NC | NC | 1 | T | ko:K07636 |
| AIL05333.1 | copA | copper-translocating P-type ATPase | 0.56 | 0.10 | 0.01 | NC | NC | NC | 1 | P | ko:K17686 |
| AIL03373.1 | rexB | PD-(D/E)XK nuclease superfamily protein | 0.69 | 0.96 | 0.37 | NC | NC | NC | 1 | L | ko:K16899 |
| AIL05542.1 | manN | PTS system, mannose/fructose/sorbose, IID component family protein | -0.42 | 0.30 | 0.18 | NC | NC | NC | 1 | G | ko:K02796 |
| AIL04863.1 | trmK | hypothetical protein DR75_454 | 0.87 | 0.87 | 0.55 | NC | NC | NC | 1 | S | ko:K06967 |
| AIL04232.1 | luxS | S-Ribosylhomocysteinase family protein | 0.31 | -0.02 | 0.02 | NC | NC | NC | 1 | H | ko:K07173 |
| AIL05483.1 | arcC | carbamate kinase | 0.80 | 0.81 | 0.56 | NC | NC | NC | 1 | E | ko:K00926 |
| AIL04427.1 | csrR | hypothetical protein DR75_109 | -0.11 | 0.12 | 0.07 | NC | NC | NC | 1 | K | - |
| AIL05065.1 | menB | naphthoate synthase | 0.32 | -0.21 | 0.11 | NC | NC | NC | 1 | H | ko:K01661 |
| AIL03308.1 | rpsG | ribosomal protein S7 | -0.40 | -0.47 | -0.86 | NC | NC | NC | 1 | J | ko:K02992 |
| AIL05371.1 | adcA | periplasmic solute binding family protein | 0.17 | -0.17 | -0.03 | NC | NC | NC | 1 | P | ko:K09815 |
| AIL05759.1 | phoU | phosphate transport system regulatory protein PhoU | 0.08 | -0.24 | -0.44 | NC | NC | NC | 1 | P | ko:K02039 |
| AIL03677.1 | accC | acetyl-CoA carboxylase, biotin carboxylase subunit | -0.55 | -0.13 | -0.17 | NC | NC | NC | 1 | I | ko:K01961 |
| AIL04507.1 | - | hypothetical protein DR75_563 | -0.05 | 0.15 | -0.14 | NC | NC | NC | 1 | - | - |
| AIL05423.1 | rbgA | ribosome biogenesis GTP-binding protein YlqF | -0.50 | -0.42 | -0.37 | NC | NC | NC | 1 | S | ko:K14540 |
| AIL03453.1 | yvgN | aldo/keto reductase family protein | 0.74 | 0.38 | 0.11 | NC | NC | NC | 1 | S | - |
| AIL05604.1 | rplI | ribosomal protein L9 | 0.80 | 0.23 | -0.08 | NC | NC | NC | 1 | J | ko:K02939 |
| AIL04698.1 | - | lipid kinase, YegS//BmrU family protein | 0.40 | 0.19 | 0.15 | NC | NC | NC | 1 | I | - |
| AIL05145.1 | yrrN | peptidase U32 family protein | -0.38 | -0.35 | -0.37 | NC | NC | NC | 1 | O | ko:K08303 |
| AIL04749.1 | tsaD | tRNA threonylcarbamoyl adenosine modification protein YgjD | -0.18 | -0.19 | -0.23 | NC | NC | NC | 1 | O | ko:K01409 |
| AIL03266.1 | rpsH | 30S ribosomal protein S8 | -0.25 | -0.27 | -0.37 | NC | NC | NC | 1 | J | ko:K02994 |
| AIL03912.1 | fabF | beta-ketoacyl-acyl-carrier-protein synthase II | -0.39 | 0.05 | 0.20 | NC | NC | NC | 1 | I | ko:K09458 |
| AIL04075.1 | nrnA | DHH family protein | -0.18 | 0.19 | 0.06 | NC | NC | NC | 1 | S | ko:K06881 |
| AIL05082.1 | maa | chorismate mutase | 0.07 | -0.20 | 0.00 | NC | NC | NC | 1 | E | ko:K00661 |
| AIL05920.1 | WQ51_01275 | EDD, DegV family domain protein | 0.21 | -0.01 | 0.11 | NC | NC | NC | 1 | S | - |
| AIL05653.1 | folD | tetrahydrofolate dehydrogenase/cyclohydrolase, catalytic domain protein | -0.01 | -0.06 | -0.23 | NC | NC | NC | 1 | F | ko:K01491 |
| AIL05105.1 | rfbA | glucose-1-phosphate thymidylyltransferase | -0.35 | -0.14 | -0.19 | NC | NC | NC | 1 | H | ko:K00973 |
| AIL05226.1 | fba | fructose-1,6-bisphosphate aldolase, class II | -0.64 | -0.10 | -0.16 | NC | NC | NC | 1 | G | ko:K01624 |
| AIL03700.1 | upp | uracil phosphoribosyltransferase | -0.18 | -0.16 | -0.15 | NC | NC | NC | 1 | F | ko:K00761 |
| AIL05302.1 | xpt | xanthine phosphoribosyltransferase | -0.44 | -0.05 | -0.12 | NC | NC | NC | 1 | F | ko:K03816 |
| AIL03344.1 | radA | DNA repair protein RadA | 0.47 | 0.02 | -0.02 | NC | NC | NC | 1 | O | ko:K04485 |
| AIL04633.1 | panE2 | 2-dehydropantoate 2-reductase family protein | 0.60 | 0.33 | 0.20 | NC | NC | NC | 1 | H | ko:K00077 |
| AIL05027.1 | hpk31 | his Kinase A domain protein | 0.88 | 0.21 | -0.42 | NC | NC | NC | 1 | T | - |
| AIL04712.1 | - | glycosyl transferase 2 family protein | -0.07 | 0.14 | -0.11 | NC | NC | NC | 1 | M | - |
| AIL03872.1 | trpS | tryptophan--tRNA ligase | -0.09 | 0.74 | 0.04 | NC | NC | NC | 1 | J | ko:K01867 |
| AIL05185.1 | yvoA_2 | UTRA domain protein | -0.60 | -0.09 | 0.00 | NC | NC | NC | 1 | K | ko:K03710 |
| AIL05100.1 | vanR | hypothetical protein DR75_1998 | 0.29 | -0.21 | -0.09 | NC | NC | NC | 1 | K | - |
| AIL03202.1 | lanT | ABC transporter family protein (plasmid) | -0.22 | 0.17 | -0.34 | NC | NC | NC | 1 | V | ko:K06148 |
| AIL03957.1 | - | UTRA domain protein | 0.46 | 0.10 | 0.15 | NC | NC | NC | 1 | K | - |
| AIL03554.1 | yqfL | kinase/pyrophosphorylase family protein | -0.48 | -0.33 | -0.97 | NC | NC | NC | 1 | S | ko:K09773 |
| AIL04119.1 | cmk | cytidylate kinase | 0.47 | 0.08 | 0.05 | NC | NC | NC | 1 | F | ko:K00945 |
| AIL03391.1 | - | hypothetical protein DR75_1893 | 0.74 | 0.34 | 0.34 | NC | NC | NC | 1 | - | - |
| AIL03847.1 | purR | pur operon repressor PurR | -0.56 | -0.11 | -0.18 | NC | NC | NC | 1 | F | ko:K09685 |
| AIL04806.1 | lutB | iron-sulfur cluster-binding protein | -0.46 | -0.19 | -0.22 | NC | NC | NC | 1 | C | ko:K18929 |
| AIL04436.1 | dinG | exonuclease, DNA polymerase III, epsilon subunit family domain protein | -0.28 | 0.65 | -0.14 | NC | NC | NC | 1 | L | ko:K03722 |
| AIL05651.1 | - | restriction endonuclease family protein | -0.96 | -0.23 | -0.35 | NC | NC | NC | 1 | L | ko:K07448 |
| AIL05405.1 | lon | PDZ domain protein | 0.67 | 0.29 | 0.52 | NC | NC | NC | 1 | T | ko:K07177 |
| AIL04624.1 | - | acetyltransferase family protein | 0.01 | -0.08 | 0.22 | NC | NC | NC | 1 | S | - |
| AIL03933.1 | rho | transcription termination factor Rho | -0.01 | 0.19 | 0.12 | NC | NC | NC | 1 | K | ko:K03628 |
| AIL05161.1 | opuAA | glycine betaine/L-proline transport ATP binding subunit | -0.02 | 0.12 | -0.10 | NC | NC | NC | 1 | E | ko:K02000 |
| AIL03487.1 | yaaT | hypothetical protein DR75_1454 | -0.57 | -0.32 | -0.09 | NC | NC | NC | 1 | S | - |
| AIL03736.1 | recG | ATP-dependent DNA helicase RecG | 0.64 | 0.14 | -0.29 | NC | NC | NC | 1 | L | ko:K03655 |
| AIL03673.1 | dltD | D-alanyl-lipoteichoic acid biosynthesis protein DltD | -0.34 | -0.80 | -0.53 | NC | NC | NC | 1 | M | ko:K03740 |
| AIL04289.1 | - | hypothetical protein DR75_1724 | 0.06 | -0.24 | 0.17 | NC | NC | NC | 1 | QT | ko:K09684 |
| AIL05060.1 | ltaS | type I phosphodiesterase / nucleotide pyrophosphatase family protein | 0.51 | 0.72 | 0.48 | NC | NC | NC | 1 | M | ko:K19005 |
| AIL03259.1 | yhaN | AAA domain protein | 0.64 | 0.79 | 0.52 | NC | NC | NC | 1 | L | - |
| AIL05152.1 | rrp1 | response regulator | -0.27 | -0.07 | -0.21 | NC | NC | NC | 1 | K | ko:K02483 |
| AIL03657.1 | XK27_10405 | bacterial membrane YfhO family protein | 0.15 | -0.17 | 0.52 | NC | NC | NC | 1 | S | - |
| AIL03331.1 | glxK | glycerate kinase family protein | -0.50 | -0.37 | -0.14 | NC | NC | NC | 1 | G | ko:K00865 |
| AIL03672.1 | cca | poly A polymerase head domain protein | -0.42 | -0.25 | -0.37 | NC | NC | NC | 1 | J | ko:K00974 |
| AIL04169.1 | - | hypothetical protein DR75_1511 | 0.25 | -0.47 | -0.29 | NC | NC | NC | 1 | S | - |
| AIL05486.1 | ymdB | ymdB-like family protein | -0.08 | 0.02 | -0.08 | NC | NC | NC | 1 | S | ko:K02029 |
| AIL03252.1 | rplJ | ribosomal L10 family protein | -0.23 | -0.19 | -0.31 | NC | NC | NC | 1 | J | ko:K02864 |
| AIL05589.1 | deoD | purine nucleoside phosphorylase | 0.56 | 0.30 | 0.26 | NC | NC | NC | 1 | F | ko:K03784 |
| AIL04071.1 | soj | sporulation initiation inhibitor protein soj | -0.03 | -0.11 | 0.05 | NC | NC | NC | 1 | D | ko:K03496 |
| AIL04208.1 | drrA | hypothetical protein DR75_1923 | 0.34 | 0.30 | 0.07 | NC | NC | NC | 1 | V | ko:K01990 |
| AIL05245.1 | cryZ | zinc-binding dehydrogenase family protein | 0.76 | 0.56 | 0.42 | NC | NC | NC | 1 | C | - |
| AIL04324.1 | deoC | deoxyribose-phosphate aldolase | 0.36 | 0.36 | 0.19 | NC | NC | NC | 1 | F | ko:K01619 |
| AIL05562.1 | ybbR | ybbR-like family protein | 0.03 | -0.12 | -0.23 | NC | NC | NC | 1 | S | - |
| AIL03660.1 | ybhE | hypothetical protein DR75_539 | -0.01 | 0.66 | 0.49 | NC | NC | NC | 1 | S | ko:K09963 |
| AIL04163.1 | rpsM | 30S ribosomal protein S13 | -0.30 | -0.20 | -0.78 | NC | NC | NC | 1 | J | ko:K02952 |
| AIL03473.1 | ispE | 4-(cytidine 5'-diphospho)-2-C-methyl-D-erythritol kinase | 0.14 | -0.26 | -0.26 | NC | NC | NC | 1 | F | ko:K00919 |
| AIL04911.1 | rplF | ribosomal protein L6 | 0.01 | 0.08 | 0.03 | NC | NC | NC | 1 | J | ko:K02933 |
| AIL04256.1 | rsmG | 16S rRNA (guanine(527)-N(7))-methyltransferase GidB | -0.02 | -0.26 | 0.12 | NC | NC | NC | 1 | J | ko:K03501 |
| AIL04903.1 | ymfH | peptidase M16 inactive domain protein | 0.44 | 0.33 | 0.11 | NC | NC | NC | 1 | S | - |
| AIL03839.1 | queA | tRNA ribosyltransferase-isomerase | 0.24 | 0.01 | -0.02 | NC | NC | NC | 1 | J | ko:K07568 |
| AIL03848.1 | murQ | N-acetylmuramic acid 6-phosphate etherase | -0.30 | 0.25 | 0.41 | NC | NC | NC | 1 | G | ko:K07106 |
| AIL04974.1 | lepB | signal peptidase I | 0.04 | -0.31 | -0.04 | NC | NC | NC | 1 | U | ko:K03100 |
| AIL05247.1 | ydhF | aldo/keto reductase family protein | 0.24 | 0.35 | 0.26 | NC | NC | NC | 1 | S | - |
| AIL03840.1 | coaBC | phosphopantothenoylcysteine decarboxylase | 0.46 | -0.23 | -0.21 | NC | NC | NC | 1 | H | ko:K01598 |
| AIL05189.1 | - | HAD hydrolase, IA, variant 1 family protein | -0.21 | 0.10 | -0.10 | NC | NC | NC | 1 | S | - |
| AIL05530.1 | - | hypothetical protein DR75_1059 | 0.79 | 0.45 | 0.27 | NC | NC | NC | 1 | S | - |
| AIL05362.1 | lytR | transcriptional regulator lytR | -0.43 | -0.18 | -0.21 | NC | NC | NC | 1 | K | - |
| AIL03582.1 | ruvB | Holliday junction DNA helicase RuvB | 0.10 | -0.38 | -0.39 | NC | NC | NC | 1 | L | ko:K03551 |
| AIL03200.1 | - | cobQ/CobB/MinD/ParA nucleotide binding domain protein (plasmid) | -0.30 | -0.18 | -0.03 | NC | NC | NC | 1 | D | - |
| AIL04981.1 | trmD | tRNA (guanine(37)-N(1))-methyltransferase | -0.68 | -0.54 | -0.07 | NC | NC | NC | 1 | J | ko:K00554 |
| AIL05903.1 | - | reactive intermediate/imine deaminase family protein | 0.45 | 0.71 | 0.80 | NC | NC | NC | 1 | J | - |
| AIL05023.1 | - | hypothetical protein DR75_2412 | 0.39 | -0.04 | -0.14 | NC | NC | NC | 1 | S | - |
| AIL05128.1 | frr | ribosome recycling factor | -0.11 | 0.17 | -0.33 | NC | NC | NC | 1 | J | ko:K02838 |
| AIL05248.1 | - | hypothetical protein DR75_2009 | 0.42 | -0.08 | -0.27 | NC | NC | NC | 1 | S | - |
| AIL04741.1 | rluD | pseudouridine synthase, RluA family protein | -0.22 | -0.34 | -0.28 | NC | NC | NC | 1 | J | ko:K06180 |
| AIL05210.1 | cadA | copper-translocating P-type ATPase | -0.58 | -0.10 | -0.21 | NC | NC | NC | 1 | P | ko:K01534 |
| AIL05754.1 | - | hypothetical protein DR75_1491 | 0.37 | 0.01 | 0.05 | NC | NC | NC | 1 | S | - |
| AIL03592.1 | - | sugar-specific transcriptional regulator TrmB family protein | -0.75 | -0.67 | -0.87 | NC | NC | NC | 1 | K | - |
| AIL04957.1 | bglX | hypothetical protein DR75_302 | 0.50 | 0.60 | 0.30 | NC | NC | NC | 1 | G | ko:K01207 |
| AIL05685.1 | map | methionine aminopeptidase, type I | 0.28 | 0.27 | 0.42 | NC | NC | NC | 1 | E | ko:K01265 |
| AIL04943.1 | brpA | transcriptional regulator lytR | -0.27 | -0.43 | -0.27 | NC | NC | NC | 1 | K | - |
| AIL05620.1 | ysdC | putative aminopeptidase ysdC | 0.17 | 0.14 | 0.19 | NC | NC | NC | 1 | G | - |
| AIL05724.1 | serA | D-isomer specific 2-hydroxyacid dehydrogenase, NAD binding domain protein | 0.60 | 0.15 | 0.30 | NC | NC | NC | 1 | EH | ko:K00058 |
| AIL03157.1 | - | fic/DOC family protein (plasmid) | 0.17 | 0.13 | 0.10 | NC | NC | NC | 1 | D | - |
| AIL04269.1 | fadD | AMP-binding enzyme family protein | -0.57 | -0.53 | -0.49 | NC | NC | NC | 1 | IQ | ko:K01895 |
| AIL03279.1 | trxB | thioredoxin-disulfide reductase | 0.22 | 0.07 | -0.18 | NC | NC | NC | 1 | C | ko:K00384 |
| AIL04465.1 | rimM | 16S rRNA processing protein RimM | 0.14 | -0.02 | 0.15 | NC | NC | NC | 1 | J | ko:K02860 |
| AIL03314.1 | def | peptide deformylase | 0.54 | 0.14 | 0.21 | NC | NC | NC | 1 | J | ko:K01462 |
| AIL05283.1 | psaA3 | periplasmic solute binding family protein | -0.34 | -0.22 | -0.02 | NC | NC | NC | 1 | P | ko:K09815 |
| AIL04672.1 | yeaZ | tRNA threonylcarbamoyl adenosine modification protein YeaZ | 0.02 | 0.22 | 0.01 | NC | NC | NC | 1 | O | ko:K01409 |
| AIL04249.1 | nrdF | ribonucleotide reductase, small chain family protein | 0.86 | 0.09 | 0.01 | NC | NC | NC | 1 | F | ko:K00526 |
| AIL05053.1 | sdaAA | L-serine dehydratase, iron-sulfur-dependent, alpha subunit | 0.04 | 0.29 | 0.05 | NC | NC | NC | 1 | E | ko:K01752 |
| AIL05406.1 | carA | carbamoyl-phosphate synthase, small subunit | -0.93 | 0.51 | 0.34 | NC | NC | NC | 1 | F | ko:K01956 |
| AIL05039.1 | sufC | FeS assembly ATPase SufC | -0.04 | 0.08 | 0.43 | NC | NC | NC | 1 | O | ko:K09013 |
| AIL05026.1 | pyrH | UMP kinase | -0.38 | -0.04 | -0.06 | NC | NC | NC | 1 | F | ko:K09903 |
| AIL05427.1 | - | phosphate binding family protein | -0.58 | -0.33 | -0.40 | NC | NC | NC | 1 | P | ko:K02040 |
| AIL04264.1 | dhrS4 | short chain dehydrogenase family protein | 0.69 | 0.32 | 0.13 | NC | NC | NC | 1 | IQ | ko:K00059 |
| AIL04262.1 | phoP | alkaline phosphatase synthesis transcriptional regulatory protein phoP | 0.13 | -0.26 | -0.11 | NC | NC | NC | 1 | K | ko:K07658 |
| AIL04523.1 | rlmN | 23S rRNA methyltransferase | -0.77 | -0.26 | -0.08 | NC | NC | NC | 1 | J | ko:K06941 |
| AIL04088.1 | lemA | lemA protein | -0.64 | -0.59 | -0.49 | NC | NC | NC | 1 | S | ko:K03744 |
| AIL05075.1 | pta | phosphate acetyltransferase | -0.46 | 0.01 | -0.13 | NC | NC | NC | 1 | C | ko:K00625 |
| AIL04320.1 | ksgA | dimethyladenosine transferase | -0.24 | -0.06 | -0.27 | NC | NC | NC | 1 | J | ko:K02528 |
| AIL03676.1 | - | kinase/pyrophosphorylase family protein | -0.21 | -0.12 | -0.05 | NC | NC | NC | 1 | F | ko:K09773 |
| AIL03834.1 | yitU | HAD hydrolase, IIB family protein | -0.27 | 0.41 | 0.32 | NC | NC | NC | 1 | S | ko:K21064 |
| AIL03474.1 | ysjB | oxidoreductase, NAD-binding Rossmann fold family protein | 0.07 | -0.01 | -0.07 | NC | NC | NC | 1 | S | ko:K03810 |
| AIL04792.1 | - | hlyD secretion family protein | -0.14 | 0.02 | 0.26 | NC | NC | NC | 1 | M | ko:K02005 |
| AIL03945.1 | - | glycosyl transferase 2 family protein | -0.19 | -0.36 | -0.04 | NC | NC | NC | 1 | M | - |
| AIL03852.1 | recD2 | viral (Super1) RNA helicase family protein | -0.30 | -0.50 | -0.26 | NC | NC | NC | 1 | L | ko:K03581 |
| AIL05216.1 | ansB | asparaginase family protein | 0.54 | -0.05 | 0.04 | NC | NC | NC | 1 | EJ | ko:K01424 |
| AIL05202.1 | - | putative yycH protein | -0.17 | -0.71 | -0.60 | NC | NC | NC | 1 | S | - |
| AIL04884.1 | - | L,D-transpeptidase catalytic domain protein | -0.02 | 0.02 | -0.61 | NC | NC | NC | 1 | S | - |
| AIL04798.1 | rbsR | periplasmic binding s and sugar binding domain of LacI family protein | -0.18 | -0.09 | -0.35 | NC | NC | NC | 1 | K | ko:K02529 |
| AIL05846.1 | - | PTS system sorbose subIIB component family protein | 0.65 | 0.06 | 0.07 | NC | NC | NC | 1 | G | ko:K02794 |
| AIL03282.1 | ybcH | dienelactone hydrolase family protein | -0.54 | -0.14 | -0.22 | NC | NC | NC | 1 | S | ko:K06889 |
| AIL03953.1 | - | ftsX-like permease family protein | 0.34 | 0.70 | -0.17 | NC | NC | NC | 1 | V | ko:K02004 |
| AIL05882.1 | hprK | HPr(Ser) kinase/phosphatase | -0.03 | 0.23 | 0.02 | NC | NC | NC | 1 | F | ko:K06023 |
| AIL04622.1 | lysR | bacterial regulatory helix-turn-helix, lysR family protein | 0.57 | 0.17 | 0.18 | NC | NC | NC | 1 | K | - |
| AIL04301.1 | lysA | diaminopimelate decarboxylase | -0.62 | -0.25 | -0.28 | NC | NC | NC | 1 | E | ko:K01586 |
| AIL04298.1 | yfiB1 | ABC transporter family protein | -0.57 | 0.06 | -0.27 | NC | NC | NC | 1 | V | ko:K06147 |
| AIL04697.1 | rplW | 50S ribosomal protein L23 | -0.13 | -0.35 | -0.38 | NC | NC | NC | 1 | J | ko:K02892 |
| AIL05613.1 | bfmBAA | 2-oxoisovalerate dehydrogenase subunit alpha | -0.19 | 0.27 | 0.48 | NC | NC | NC | 1 | C | ko:K00166 |
| AIL05276.1 | ywlG | hypothetical protein DR75_928 | -0.15 | -0.22 | -0.36 | NC | NC | NC | 1 | S | - |
| AIL04866.1 | rplO | ribosomal protein L15 | -0.07 | 0.15 | -0.02 | NC | NC | NC | 1 | J | ko:K02876 |
| AIL03610.1 | ribF | riboflavin biosynthesis protein RibF | 0.03 | -0.06 | 0.24 | NC | NC | NC | 1 | H | ko:K11753 |
| AIL05680.1 | yqhL | rhodanese-like domain protein | -0.37 | -0.62 | -0.54 | NC | NC | NC | 1 | P | - |
| AIL04151.1 | - | quinone oxidoreductase, YhdH/YhfP family protein | 0.09 | 0.54 | 0.47 | NC | NC | NC | 1 | C | ko:K00001 |
| AIL05261.1 | hom | oxidoreductase, NAD-binding Rossmann fold family protein | 0.26 | -0.25 | -0.25 | NC | NC | NC | 1 | E | ko:K00003 |
| AIL03757.1 | ilvE | branched-chain amino acid aminotransferase | -0.06 | 0.06 | -0.03 | NC | NC | NC | 1 | E | ko:K00826 |
| AIL05376.1 | ysdB | ABC transporter family protein | -0.56 | -0.41 | 0.09 | NC | NC | NC | 1 | S | ko:K01990 |
| AIL05911.1 | hpt | hypoxanthine phosphoribosyltransferase | -0.14 | -0.07 | -0.07 | NC | NC | NC | 1 | F | ko:K00760 |
| AIL04829.1 | fpg | formamidopyrimidine-DNA glycosylase | 0.36 | -0.14 | -0.02 | NC | NC | NC | 1 | L | ko:K10563 |
| AIL04975.1 | mntA | manganese ABC transporter substrate-binding lipoprotein | 0.06 | -0.13 | -0.22 | NC | NC | NC | 1 | P | ko:K19971 |
| AIL04114.1 | rplS | ribosomal protein L19 | -0.24 | 0.01 | -0.31 | NC | NC | NC | 1 | J | ko:K02884 |
| AIL05905.1 | pgm7 | histidine phosphatase super family protein | 0.10 | 0.74 | 0.84 | NC | NC | NC | 1 | G | - |
| AIL04979.1 | nadE | NAD+ synthetase | 0.47 | 0.05 | -0.07 | NC | NC | NC | 1 | H | ko:K01916 |
| AIL04218.1 | accA | acetyl-CoA carboxylase, carboxyl transferase, alpha subunit | -0.49 | -0.10 | -0.07 | NC | NC | NC | 1 | I | ko:K01962 |
| AIL03990.1 | rpsF | ribosomal protein S6 | -0.12 | -0.01 | -0.25 | NC | NC | NC | 1 | J | ko:K02990 |
| AIL04120.1 | oppC | binding--dependent transport system inner membrane component family protein | -0.42 | -0.13 | -0.24 | NC | NC | NC | 1 | EP | ko:K15582 |
| AIL03352.1 | birA | biotin--[acetyl-CoA-carboxylase] ligase | 0.02 | 0.22 | -0.01 | NC | NC | NC | 1 | K | ko:K03524 |
| AIL05328.1 | ktrA | trkA-N domain protein | 0.22 | 0.12 | 0.15 | NC | NC | NC | 1 | P | ko:K03499 |
| AIL05197.1 | XK27_08360 | EDD, DegV family domain protein | -0.34 | -0.26 | -0.13 | NC | NC | NC | 1 | S | - |
| AIL03802.1 | pyrC | dihydroorotase | 0.00 | 0.66 | -0.40 | NC | NC | NC | 1 | F | ko:K01465 |
| AIL04398.1 | fruR | hypothetical protein DR75_2676 | -0.08 | 0.26 | 0.52 | NC | NC | NC | 1 | K | ko:K03436 |
| AIL04813.1 | - | amidinotransferase family protein | 0.13 | 0.11 | 0.28 | NC | NC | NC | 1 | E | - |
| AIL04710.1 | plsC | acyltransferase family protein | 0.14 | -0.06 | -0.06 | NC | NC | NC | 1 | I | ko:K00655 |
| AIL03550.1 | - | NUDIX domain protein | -0.04 | -0.25 | -0.42 | NC | NC | NC | 1 | F | ko:K01515 |
| AIL05213.1 | iscS2 | beta-eliminating lyase family protein | -0.56 | -0.34 | -0.42 | NC | NC | NC | 1 | E | ko:K04487 |
| AIL03825.1 | nagA | N-acetylglucosamine-6-phosphate deacetylase | 0.04 | 0.19 | 0.05 | NC | NC | NC | 1 | G | ko:K01443 |
| AIL03741.1 | rplM | ribosomal protein L13 | -0.21 | -0.17 | -0.25 | NC | NC | NC | 1 | J | ko:K02871 |
| AIL05408.1 | suhB | inositol monophosphatase family protein | 0.28 | 0.04 | -0.02 | NC | NC | NC | 1 | G | ko:K01092 |
| AIL03737.1 | menD | 2-succinyl-5-enolpyruvyl-6-hydroxy-3-cyclohexene-1-carboxylic-acid synthase | 0.69 | -0.22 | 0.39 | NC | NC | NC | 1 | H | ko:K02551 |
| AIL03446.1 | glnQ | ABC transporter family protein | -0.48 | -0.11 | -0.33 | NC | NC | NC | 1 | E | ko:K17076 |
| AIL04660.1 | ecfA1 | ABC transporter family protein | -0.18 | -0.30 | -0.34 | NC | NC | NC | 1 | P | ko:K16786 |
| AIL03443.1 | yodB | hxlR-like helix-turn-helix family protein | 0.39 | -0.18 | -0.06 | NC | NC | NC | 1 | K | - |
| AIL05820.1 | dapH | 2,3,4,5-tetrahydropyridine-2,6-dicarboxylate N-acetyltransferase | -0.12 | -0.25 | -0.25 | NC | NC | NC | 1 | E | ko:K00674 |
| AIL03714.1 | - | metallopeptidase M24 family protein | -0.08 | -0.05 | 0.17 | NC | NC | NC | 1 | E | - |
| AIL03544.1 | vicK | sensory box protein | -0.02 | 0.01 | -0.51 | NC | NC | NC | 1 | T | ko:K07652 |
| AIL04235.1 | guaC | guanosine monophosphate reductase | -0.09 | -0.43 | -0.37 | NC | NC | NC | 1 | F | ko:K00364 |
| AIL04463.1 | tuaA | exopolysaccharide biosynthesis polyprenyl glycosylphosphotransferase family protein | -0.37 | 0.00 | -0.15 | NC | NC | NC | 1 | M | - |
| AIL03106.1 | - | peptidase S41 family protein (plasmid) | 0.34 | -0.03 | 0.21 | NC | NC | NC | 1 | - | - |
| AIL03406.1 | dgs | glycosyl transferases group 1 family protein | 0.22 | 0.21 | 0.06 | NC | NC | NC | 1 | M | ko:K13677 |
| AIL04063.1 | exoA | exodeoxyribonuclease III | 0.04 | 0.06 | -0.03 | NC | NC | NC | 1 | L | ko:K01142 |
| AIL05113.1 | fni | isopentenyl-diphosphate delta-isomerase, type 2 | -0.18 | -0.32 | -0.10 | NC | NC | NC | 1 | C | ko:K01823 |
| AIL04693.1 | - | hypothetical protein DR75_1978 | 0.11 | -0.07 | -0.01 | NC | NC | NC | 1 | KT | - |
| AIL04362.1 | ywbD | S-adenosylmethionine-dependent methyltransferase family protein | 0.23 | -0.39 | -0.57 | NC | NC | NC | 1 | J | ko:K06969 |
| AIL03803.1 | metE | cobalamin-independent synthase, Catalytic domain protein | -0.71 | -0.43 | -0.10 | NC | NC | NC | 1 | E | ko:K00549 |
| AIL03988.1 | hemN | radical SAM superfamily protein | -0.28 | -0.19 | -0.39 | NC | NC | NC | 1 | H | - |
| AIL03160.1 | - | phoH-like family protein (plasmid) | 0.37 | 0.53 | 0.32 | NC | NC | NC | 1 | L | - |
| AIL03536.1 | yunD | hypothetical protein DR75_251 | -0.16 | -0.22 | -0.78 | NC | NC | NC | 1 | F | ko:K01081 |
| AIL03829.1 | tpiA | triose-phosphate isomerase | 0.12 | 0.11 | -0.16 | NC | NC | NC | 1 | G | ko:K01803 |
| AIL05191.1 | hepT | polyprenyl synthetase family protein | -0.02 | 0.04 | 0.07 | NC | NC | NC | 1 | H | ko:K00805 |
| AIL05488.1 | pth | peptidyl-tRNA hydrolase | 0.69 | 0.03 | 0.02 | NC | NC | NC | 1 | J | ko:K01056 |
| AIL04745.1 | yheA | hypothetical protein DR75_2631 | -0.39 | -0.08 | -0.79 | NC | NC | NC | 1 | S | - |
| AIL04393.1 | rfbC | dTDP-4-dehydrorhamnose 3,5-epimerase | -0.19 | -0.12 | -0.16 | NC | NC | NC | 1 | G | ko:K01790 |
| AIL03679.1 | tagD | glycerol-3-phosphate cytidylyltransferase | -0.23 | -0.22 | -0.06 | NC | NC | NC | 1 | IM | ko:K00968 |
| AIL04057.1 | vraR | bacterial regulatory s, luxR family protein | 0.23 | 0.08 | -0.09 | NC | NC | NC | 1 | K | ko:K07694 |
| AIL05919.1 | ispA | farnesyl diphosphate synthase | -0.48 | -0.25 | -0.18 | NC | NC | NC | 1 | H | ko:K13789 |
| AIL03946.1 | yumC | ferredoxin--NADP reductase 2 | -0.21 | -0.18 | -0.20 | NC | NC | NC | 1 | C | ko:K21567 |
| AIL05165.1 | mvaS | hydroxymethylglutaryl-CoA synthase | -0.07 | -0.15 | -0.33 | NC | NC | NC | 1 | I | ko:K01641 |
| AIL05887.1 | uspA | universal stress family protein | 0.52 | 0.37 | 0.58 | NC | NC | NC | 1 | T | - |
| AIL05319.1 | fabZ | beta-hydroxyacyl-(acyl-carrier-protein) dehydratase FabZ | -0.28 | 0.33 | 0.06 | NC | NC | NC | 1 | I | ko:K02372 |
| AIL03309.1 | - | HTH domain protein | 0.53 | -0.28 | 0.95 | NC | NC | NC | 1 | S | - |
| AIL03491.1 | secY | preprotein translocase, SecY subunit | 0.03 | 0.03 | -0.27 | NC | NC | NC | 1 | U | ko:K03076 |
| AIL03855.1 | pfkB | 1-phosphofructokinase | -0.11 | 0.26 | 0.24 | NC | NC | NC | 1 | H | ko:K00882 |
| AIL04788.1 | - | cyclophilin type peptidyl-prolyl cis-trans isomerase/CLD family protein | 0.51 | -0.08 | -0.18 | NC | NC | NC | 1 | M | ko:K03767 |
| AIL03809.1 | coaB | phosphopantothenate--cysteine ligase | 0.23 | 0.00 | 0.20 | NC | NC | NC | 1 | H | ko:K21977 |
| AIL05168.1 | gpmB | histidine phosphatase super family protein | 0.35 | 0.04 | -0.08 | NC | NC | NC | 1 | G | ko:K15640 |
| AIL03683.1 | cspR | tRNA (cytidine(34)-2'-O)-methyltransferase | -0.16 | -0.42 | -0.21 | NC | NC | NC | 1 | J | ko:K03216 |
| AIL05209.1 | pyrD | dihydroorotate dehydrogenase | -0.09 | 0.32 | 0.04 | NC | NC | NC | 1 | F | ko:K00226 |
| AIL04110.1 | - | glycosyl transferase 2 family protein | 0.44 | -0.17 | -0.18 | NC | NC | NC | 1 | M | ko:K20534 |
| AIL03442.1 | metQ | NLPA lipofamily protein | 0.55 | 0.46 | 0.65 | NC | NC | NC | 1 | M | ko:K02072 |
| AIL04765.1 | iscS | aminotransferase class-V family protein | -0.01 | -0.13 | -0.29 | NC | NC | NC | 1 | E | ko:K04487 |
| AIL05649.1 | atpH | ATP synthase F1, delta subunit | -0.56 | -0.68 | -0.73 | NC | NC | NC | 1 | C | ko:K02113 |
| AIL04818.1 | nudF | NUDIX domain protein | 0.15 | 0.18 | 0.43 | NC | NC | NC | 1 | L | ko:K01515 |
| AIL04414.1 | coaE | dephospho-CoA kinase | 0.46 | -0.21 | -0.16 | NC | NC | NC | 1 | F | ko:K00859 |
| AIL05295.1 | rsmD | RNA methyltransferase, RsmD family | -0.10 | 0.09 | -0.17 | NC | NC | NC | 1 | L | ko:K08316 |
| AIL03906.1 | est | esterase D | 0.22 | 0.00 | -0.11 | NC | NC | NC | 1 | S | ko:K03928 |
| AIL03871.1 | - | ABC transporter family protein | 0.15 | 0.03 | -0.50 | NC | NC | NC | 1 | V | ko:K11635 |
| AIL05745.1 | holB | DNA polymerase III, delta' subunit | -0.22 | 0.26 | 0.52 | NC | NC | NC | 1 | L | ko:K02341 |
| AIL05539.1 | thrB | homoserine kinase | 0.40 | 0.05 | -0.08 | NC | NC | NC | 1 | F | ko:K00872 |
| AIL04807.1 | sirR | feoA domain protein | 0.25 | -0.03 | -0.14 | NC | NC | NC | 1 | K | ko:K03709 |
| AIL03244.1 | - | glycosyl transferase 2 family protein | 0.08 | 0.10 | 0.00 | NC | NC | NC | 1 | S | ko:K07011 |
| AIL04328.1 | - | hypothetical protein DR75_314 | -0.57 | -0.43 | -0.45 | NC | NC | NC | 1 | - | - |
| AIL04799.1 | scrR | helix-turn-helix family protein | 0.06 | -0.06 | -0.32 | NC | NC | NC | 1 | K | ko:K02529 |
| AIL03725.1 | apt | adenine phosphoribosyltransferase | -0.17 | -0.03 | 0.00 | NC | NC | NC | 1 | F | ko:K00759 |
| AIL03187.1 | ssb | single-stranded DNA-binding family protein (plasmid) | -0.16 | -0.28 | -0.53 | NC | NC | NC | 1 | L | ko:K03111 |
| AIL04105.1 | yrrK | hypothetical protein DR75_268 | 0.76 | 0.55 | 0.21 | NC | NC | NC | 1 | L | ko:K07447 |
| AIL05167.1 | - | lipid kinase, YegS//BmrU family protein | 0.60 | -0.04 | 0.55 | NC | NC | NC | 1 | I | - |
| AIL03445.1 | yfnB | putative HAD-hydrolase yfnB | 0.30 | 0.05 | -0.05 | NC | NC | NC | 1 | S | ko:K01560 |
| AIL03634.1 | - | HAD hydrolase, IIB family protein | -0.60 | -0.20 | -0.22 | NC | NC | NC | 1 | S | - |
| AIL04529.1 | XK27_08850 | aminoacyl-tRNA editing domain protein | 0.11 | -0.57 | -0.41 | NC | NC | NC | 1 | S | ko:K19055 |
| AIL03410.1 | - | sensory box protein | -0.63 | -0.32 | -0.32 | NC | NC | NC | 1 | S | - |
| AIL03497.1 | - | periplasmic binding family protein | -0.77 | -0.31 | -0.37 | NC | NC | NC | 1 | P | ko:K02016 |
| AIL05278.1 | cydC | thiol reductant ABC exporter, CydD subunit | -0.49 | -0.13 | -0.34 | NC | NC | NC | 1 | CO | ko:K16013 |
| AIL05410.1 | dagK | lipid kinase, YegS//BmrU family protein | -0.23 | 0.04 | -0.04 | NC | NC | NC | 1 | I | ko:K07029 |
| AIL04074.1 | yqeY | gatB domain protein | -0.05 | -0.34 | -0.33 | NC | NC | NC | 1 | S | ko:K09117 |
| AIL05111.1 | panB | 3-methyl-2-oxobutanoate hydroxymethyltransferase | -0.63 | -0.12 | -0.12 | NC | NC | NC | 1 | H | ko:K00606 |
| AIL04828.1 | - | peptidase propeptide and YPEB domain protein | 0.03 | 0.01 | -0.11 | NC | NC | NC | 1 | S | - |
| AIL05944.1 | - | hypothetical protein DR75_1473 | -0.60 | -0.92 | -0.80 | NC | NC | NC | 1 | G | ko:K00965 |
| AIL03758.1 | potD3 | bacterial extracellular solute-binding family protein | -0.53 | -0.04 | 0.38 | NC | NC | NC | 1 | E | ko:K02055 |
| AIL03611.1 | - | putative lipoprotein | -0.35 | 0.00 | 0.11 | NC | NC | NC | 1 | - | - |
| AIL05180.1 | copB | copper-translocating P-type ATPase | 0.42 | 0.36 | -0.04 | NC | NC | NC | 1 | P | ko:K01533 |
| AIL05545.1 | ycsE | HAD hydrolase, IIB family protein | 0.34 | 0.11 | 0.15 | NC | NC | NC | 1 | S | - |
| AIL04565.1 | proB | glutamate 5-kinase | -0.30 | -0.37 | -0.63 | NC | NC | NC | 1 | F | ko:K00931 |
| AIL03980.1 | malR | helix-turn-helix family protein | -0.21 | -0.08 | -0.25 | NC | NC | NC | 1 | K | ko:K02529 |
| AIL05221.1 | mvaD | diphosphomevalonate decarboxylase | -0.26 | -0.15 | -0.50 | NC | NC | NC | 1 | I | ko:K01597 |
| AIL03502.1 | - | recombinase family protein | 0.46 | 0.49 | -0.78 | NC | NC | NC | 1 | L | - |
| AIL03942.1 | truB | tRNA pseudouridine(55) synthase | -0.20 | -0.20 | -0.40 | NC | NC | NC | 1 | J | ko:K03177 |
| AIL05066.1 | yitK | hypothetical protein DR75_236 | 0.49 | 0.06 | -0.04 | NC | NC | NC | 1 | S | ko:K09767 |
| AIL04548.1 | mntB | ABC transporter family protein | -0.30 | 0.23 | -0.25 | NC | NC | NC | 1 | P | ko:K02074 |
| AIL05669.1 | ytbE | hypothetical protein DR75_2579 | 0.46 | 0.22 | 0.07 | NC | NC | NC | 1 | S | - |
| AIL05607.1 | - | ROK family protein | -0.09 | 0.97 | 0.63 | NC | NC | NC | 1 | GK | ko:K00847 |
| AIL03962.1 | - | ABC transporter family protein | -0.14 | 0.82 | -0.07 | NC | NC | NC | 1 | P | ko:K02071 |
| AIL05828.1 | - | helix-turn-helix domain protein | 0.90 | -0.24 | 0.13 | NC | NC | NC | 1 | L | - |
| AIL04783.1 | oppD | hypothetical protein DR75_1822 | -0.07 | -0.18 | 0.26 | NC | NC | NC | 1 | P | ko:K02031 |
| AIL04522.1 | ntpE | putative v-type ATPase, subunit E | -0.07 | 0.16 | -0.22 | NC | NC | NC | 1 | C | ko:K02121 |
| AIL03982.1 | - | SIS domain protein | -0.85 | -0.53 | -0.48 | NC | NC | NC | 1 | G | ko:K19510 |
| AIL04185.1 | - | hypothetical protein DR75_1501 | -0.11 | -0.36 | -0.21 | NC | NC | NC | 1 | M | - |
| AIL05240.1 | yusI | putative arsenate reductase | 0.07 | 0.14 | -0.04 | NC | NC | NC | 1 | P | ko:K00537 |
| AIL04486.1 | - | type I restriction modification DNA specificity domain protein | -0.82 | -0.10 | -0.68 | NC | NC | NC | 1 | V | ko:K01154 |
| AIL05618.1 | murQ | N-acetylmuramic acid 6-phosphate etherase | -0.13 | 0.31 | 0.51 | NC | NC | NC | 1 | G | ko:K07106 |
| AIL03858.1 | norG_2 | bacterial regulatory s, gntR family protein | 0.18 | 0.83 | -0.21 | NC | NC | NC | 1 | K | - |
| AIL03985.1 | yfiC | ABC transporter family protein | 0.21 | 0.29 | 0.03 | NC | NC | NC | 1 | V | ko:K06147 |
| AIL03688.1 | aatB | polar amino acid ABC uptake transporter substrate binding protein | 0.04 | -0.55 | 0.60 | NC | NC | NC | 1 | ET | ko:K02030 |
| AIL03297.1 | - | hypothetical protein DR75_900 | 0.20 | 0.07 | -0.16 | NC | NC | NC | 1 | - | ko:K02029 |
| AIL03924.1 | ssb | single-stranded DNA-binding family protein | 0.03 | -0.49 | -0.15 | NC | NC | NC | 1 | L | ko:K03111 |
| AIL03641.1 | trmH | RNA 2'-O ribose methyltransferase substrate binding family protein | -0.19 | 0.20 | -0.16 | NC | NC | NC | 1 | J | ko:K03218 |
| AIL05124.1 | ppiB | cyclophilin type peptidyl-prolyl cis-trans isomerase/CLD family protein | 0.09 | 0.20 | 0.24 | NC | NC | NC | 1 | O | ko:K03768 |
| AIL04686.1 | XK27_05225 | tetratricopeptide repeat family protein | 0.12 | 0.27 | -0.04 | NC | NC | NC | 1 | S | - |
| AIL04703.1 | dps | DNA protection during starvation protein | 0.15 | -0.42 | 0.12 | NC | NC | NC | 1 | P | ko:K04047 |
| AIL05419.1 | hup | DNA-binding protein HU | -0.17 | -0.16 | -0.58 | NC | NC | NC | 1 | L | ko:K03530 |
| AIL04727.1 | yabR | S1 RNA binding domain protein | -0.12 | 0.07 | -0.08 | NC | NC | NC | 1 | J | ko:K07571 |
| AIL05599.1 | fabH | 3-oxoacyl-[acyl-carrier-protein] synthase 3 | -0.55 | 0.11 | 0.08 | NC | NC | NC | 1 | I | ko:K00648 |
| AIL05655.1 | pdxK | pyridoxine kinase | 0.28 | 0.20 | 0.24 | NC | NC | NC | 1 | H | ko:K00868 |
| AIL05864.1 | rplN | ribosomal protein L14 | -0.27 | -0.31 | -0.29 | NC | NC | NC | 1 | J | ko:K02874 |
| AIL03523.1 | ylmE | hypothetical protein DR75_58 | -0.52 | -0.56 | -0.08 | NC | NC | NC | 1 | S | ko:K06997 |
| AIL03452.1 | gpsA | ketopantoate reductase PanE/ApbA family protein | 0.03 | -0.12 | -0.21 | NC | NC | NC | 1 | I | ko:K00057 |
| AIL04861.1 | ywiB | hypothetical protein DR75_216 | 0.06 | 0.04 | -0.01 | NC | NC | NC | 1 | S | - |
| AIL04857.1 | - | putative septicolysin | 0.23 | -0.12 | -0.68 | NC | NC | NC | 1 | - | - |
| AIL05468.1 | trmB | tRNA (guanine-N(7)-)-methyltransferase | -0.12 | 0.08 | -0.36 | NC | NC | NC | 1 | J | ko:K03439 |
| AIL04820.1 | divIB | cell division FtsQ family protein | -0.57 | -0.25 | -0.26 | NC | NC | NC | 1 | D | ko:K03589 |
| AIL05203.1 | yqeK | HD domain protein | 0.36 | -0.47 | -0.92 | NC | NC | NC | 1 | H | - |
| AIL03995.1 | lacD | tagatose 1,6-diphosphate aldolase | 0.57 | 0.54 | 0.39 | NC | NC | NC | 1 | G | ko:K01635 |
| AIL04329.1 | greA | transcription elongation factor greA | -0.42 | 0.15 | -0.03 | NC | NC | NC | 1 | K | ko:K03624 |
| AIL05657.1 | ppaC | putative manganese-dependent inorganic pyrophosphatase | -0.16 | 0.09 | -0.29 | NC | NC | NC | 1 | C | ko:K15986 |
| AIL03900.1 | mtnN | MTA/SAH nucleosidase | 0.53 | 0.25 | 0.04 | NC | NC | NC | 1 | E | ko:K01243 |
| AIL03764.1 | yrxA | 3H domain protein | 0.30 | 0.05 | 0.23 | NC | NC | NC | 1 | S | ko:K07105 |
| AIL04942.1 | ydaO | amino acid permease family protein | -0.35 | -0.41 | -0.42 | NC | NC | NC | 1 | E | - |
| AIL03417.1 | nanE | thiazole biosynthesis ThiG family protein | 0.04 | 0.34 | 0.08 | NC | NC | NC | 1 | G | ko:K01788 |
| AIL05426.1 | - | SPFH domain / Band 7 family protein | 0.39 | 0.00 | 0.00 | NC | NC | NC | 1 | O | - |
| AIL03533.1 | nagB | glucosamine-6-phosphate deaminase | 0.34 | 0.37 | 0.26 | NC | NC | NC | 1 | G | ko:K02564 |
| AIL04430.1 | cps2D | 3-beta hydroxysteroid dehydrogenase/isomerase family protein | -0.79 | -0.21 | -0.38 | NC | NC | NC | 1 | GM | ko:K01784 |
| AIL05612.1 | yclJ | response regulator | 0.50 | 0.14 | 0.41 | NC | NC | NC | 1 | K | ko:K02483 |
| AIL05518.1 | - | hypothetical protein DR75_1344 | 0.57 | 0.31 | 0.31 | NC | NC | NC | 1 | S | ko:K03595 |
| AIL04931.1 | murI | glutamate racemase | 0.03 | -0.29 | -0.21 | NC | NC | NC | 1 | M | ko:K01776 |
| AIL03320.1 | cinA | hypothetical protein DR75_1888 | 0.51 | 0.30 | 0.06 | NC | NC | NC | 1 | S | ko:K03742 |
| AIL04349.1 | - | hypothetical protein DR75_1625 | 0.79 | 0.04 | 0.36 | NC | NC | NC | 1 | - | - |
| AIL03873.1 | rluA | pseudouridine synthase, RluA family protein | -0.25 | -0.13 | -0.36 | NC | NC | NC | 1 | J | ko:K06177 |
| AIL05876.1 | prmA | ribosomal protein L11 methyltransferase | 0.00 | -0.19 | 0.04 | NC | NC | NC | 1 | J | ko:K02687 |
| AIL03349.1 | tmk | thymidylate kinase | -0.81 | -0.22 | -0.24 | NC | NC | NC | 1 | F | ko:K00943 |
| AIL04723.1 | mleS | malic enzyme, NAD binding domain protein | -0.19 | -0.20 | 0.07 | NC | NC | NC | 1 | C | ko:K00027 |
| AIL05800.1 | ycfI | ABC transporter family protein | -0.09 | -0.07 | -0.35 | NC | NC | NC | 1 | V | ko:K06147 |
| AIL05454.1 | yloC | hypothetical protein DR75_1846 | -0.36 | -0.63 | -0.55 | NC | NC | NC | 1 | S | - |
| AIL05873.1 | ptpA | low molecular weight protein-tyrosine-phosphatase yfkJ | -0.07 | 0.18 | 0.61 | NC | NC | NC | 1 | T | ko:K01104 |
| AIL05285.1 | - | DNA/RNA non-specific endonuclease family protein | -0.10 | 0.03 | 0.20 | NC | NC | NC | 1 | F | ko:K15051 |
| AIL05664.1 | ybaK | aminoacyl-tRNA editing domain protein | 0.56 | 0.24 | 0.00 | NC | NC | NC | 1 | J | ko:K03976 |
| AIL05906.1 | yqeG | HAD hydrolase, IA, variant 1 family protein | -0.34 | -0.87 | -0.99 | NC | NC | NC | 1 | S | ko:K07015 |
| AIL04271.1 | guaD | guanine deaminase | -0.01 | -0.23 | -0.34 | NC | NC | NC | 1 | F | ko:K01487 |
| AIL03949.1 | - | hypothetical protein DR75_1756 | 0.92 | 0.53 | 0.61 | NC | NC | NC | 1 | S | - |
| AIL03411.1 | - | hypothetical protein DR75_2749 | 0.03 | -0.66 | -0.13 | NC | NC | NC | 1 | S | - |
| AIL04572.1 | - | galactose mutarotase related enzyme | 0.07 | 0.53 | 0.16 | NC | NC | NC | 1 | G | - |
| AIL05170.1 | gldA | iron-containing alcohol dehydrogenase family protein | -0.29 | 0.36 | 0.28 | NC | NC | NC | 1 | C | ko:K08317 |
| AIL05321.1 | ntpI | V-type ATPase subunit | -0.38 | -0.17 | -0.05 | NC | NC | NC | 1 | U | ko:K02123 |
| AIL05869.1 | truA | tRNA pseudouridine(38-40) synthase | 0.13 | -0.18 | 0.20 | NC | NC | NC | 1 | J | ko:K06173 |
| AIL03488.1 | vraS | histidine kinase family protein | -0.59 | 0.36 | 0.75 | NC | NC | NC | 1 | T | ko:K07681 |
| AIL04205.1 | yjbM | hypothetical protein DR75_1370 | -0.29 | 0.49 | 0.27 | NC | NC | NC | 1 | S | ko:K07816 |
| AIL04401.1 | - | alpha/beta hydrolase family protein | 0.99 | 0.07 | 0.31 | NC | NC | NC | 1 | S | ko:K06889 |
| AIL03243.1 | cydD | thiol reductant ABC exporter, CydC subunit | -0.09 | -0.35 | -0.41 | NC | NC | NC | 1 | CO | ko:K16012 |
| AIL03493.1 | yitV | phospholipase/Carboxylesterase family protein | 0.49 | 0.04 | -0.17 | NC | NC | NC | 1 | S | ko:K06889 |
| AIL03289.1 | vicX | metallo-beta-lactamase superfamily protein | -0.28 | -0.11 | -0.53 | NC | NC | NC | 1 | S | ko:K00784 |
| AIL03295.1 | ykuT | mechanosensitive ion channel family protein | -0.26 | -0.02 | -0.12 | NC | NC | NC | 1 | M | ko:K16052 |
| AIL04876.1 | tagF1 | Poly(glycerophosphate) glycerophosphotransferase family protein | 0.33 | 0.28 | -0.35 | NC | NC | NC | 1 | M | ko:K09809 |
| AIL05452.1 | clpP | ATP-dependent Clp endopeptidase, proteolytic subunit ClpP | 0.55 | 0.04 | -0.28 | NC | NC | NC | 1 | O | ko:K01358 |
| AIL05441.1 | efp | translation elongation factor P | -0.02 | -0.02 | 0.03 | NC | NC | NC | 1 | J | ko:K02356 |
| AIL05512.1 | dak | deoxynucleoside kinase family protein | 0.53 | -0.03 | -0.19 | NC | NC | NC | 1 | F | - |
| AIL04474.1 | - | iron-containing alcohol dehydrogenase family protein | 0.56 | 0.08 | 0.08 | NC | NC | NC | 1 | C | ko:K08317 |
| AIL03296.1 | hpf | ribosomal subunit interface protein | 0.62 | 0.39 | 0.31 | NC | NC | NC | 1 | J | ko:K05808 |
| AIL03787.1 | rpsJ | ribosomal protein S10 | -0.61 | -0.47 | -0.60 | NC | NC | NC | 1 | J | ko:K02946 |
| AIL03312.1 | engB | ribosome biogenesis GTP-binding protein YsxC | -0.11 | -0.20 | -0.12 | NC | NC | NC | 1 | D | ko:K03978 |
| AIL04850.1 | rplQ | ribosomal protein L17 | -0.30 | -0.19 | -0.94 | NC | NC | NC | 1 | J | ko:K02879 |
| AIL03362.1 | - | hypothetical protein DR75_2725 | 0.42 | 0.05 | 0.08 | NC | NC | NC | 1 | - | - |
| AIL03135.1 | - | arsenical resistance operon trans-acting repressor ArsD (plasmid) | 0.84 | -0.17 | -0.21 | NC | NC | NC | 1 | S | - |
| AIL05131.1 | - | C4-dicarboxylate anaerobic carrier family protein | 0.95 | 0.99 | 0.84 | NC | NC | NC | 1 | S | - |
| AIL05807.1 | XK27_00835 | Cof-like hydrolase family protein | -0.38 | -0.08 | 0.08 | NC | NC | NC | 1 | S | - |
| AIL04552.1 | - | hypothetical protein DR75_2003 | -0.58 | -0.35 | -0.41 | NC | NC | NC | 1 | S | - |
| AIL04044.1 | - | hypothetical protein DR75_1100 | 0.13 | 0.31 | 0.28 | NC | NC | NC | 1 | S | - |
| AIL04036.1 | - | beta-phosphoglucomutase | 0.03 | 0.19 | -0.03 | NC | NC | NC | 1 | S | ko:K01838 |
| AIL04323.1 | - | CBS domain protein | 0.37 | -0.01 | -0.15 | NC | NC | NC | 1 | S | - |
| AIL05439.1 | pyrD | dihydroorotate dehydrogenase B, catalytic subunit | 0.58 | 0.54 | 0.28 | NC | NC | NC | 1 | F | ko:K00226 |
| AIL03389.1 | nadK | ATP-NAD kinase family protein | 0.42 | 0.87 | -0.08 | NC | NC | NC | 1 | H | ko:K00858 |
| AIL05815.1 | - | ABC transporter family protein | 0.03 | 0.03 | -0.19 | NC | NC | NC | 1 | V | ko:K02003 |
| AIL04439.1 | nusG | transcription termination/antitermination factor NusG | -0.08 | 0.12 | 0.10 | NC | NC | NC | 1 | K | ko:K02601 |
| AIL04532.1 | ymfM | helix-turn-helix domain protein | -0.47 | -0.28 | -0.38 | NC | NC | NC | 1 | S | ko:K15539 |
| AIL03532.1 | fabG | 3-oxoacyl-[acyl-carrier-protein] reductase | -0.53 | 0.04 | -0.17 | NC | NC | NC | 1 | IQ | ko:K00059 |
| AIL04178.1 | ypmB | hypothetical protein DR75_1103 | -0.33 | -0.31 | -0.16 | NC | NC | NC | 1 | S | - |
| AIL04058.1 | dhaK | dihydroxyacetone kinase, DhaK subunit | 0.77 | 0.93 | 0.94 | NC | NC | NC | 1 | G | ko:K05878 |
| AIL03522.1 | - | acetyltransferase domain protein | -0.09 | -0.12 | -0.23 | NC | NC | NC | 1 | K | ko:K06977 |
| AIL05862.1 | busR | trkA-C domain protein | 0.65 | -0.12 | -0.05 | NC | NC | NC | 1 | K | - |
| AIL04242.1 | grpE | grpE family protein | -0.21 | -0.33 | -0.57 | NC | NC | NC | 1 | O | ko:K03687 |
| AIL05047.1 | fat | acyl-ACP thioesterase family protein | 0.14 | 0.10 | 0.00 | NC | NC | NC | 1 | I | ko:K01071 |
| AIL03401.1 | - | bacterial regulatory s, tetR family protein | 0.65 | 0.28 | 0.43 | NC | NC | NC | 1 | K | - |
| AIL05688.1 | - | ROK family protein | -0.08 | 0.51 | 0.24 | NC | NC | NC | 1 | GK | - |
| AIL05338.1 | mscL | large conductance mechanosensitive channel protein | 0.49 | -0.05 | -0.10 | NC | NC | NC | 1 | M | ko:K03282 |
| AIL03682.1 | - | PTS system sorbose subIIB component family protein | 0.48 | 0.10 | 0.45 | NC | NC | NC | 1 | G | ko:K02794 |
| AIL04919.1 | - | PTS system sorbose subIIB component family protein | 0.34 | -0.15 | 0.00 | NC | NC | NC | 1 | G | ko:K02794 |
| AIL05519.1 | tatD | hydrolase, TatD family protein | 0.18 | 0.16 | -0.06 | NC | NC | NC | 1 | L | ko:K03424 |
| AIL04070.1 | rrmA | S-adenosyl-L-methionine-dependent methyltransferase family protein | 0.45 | 0.33 | 0.27 | NC | NC | NC | 1 | Q | ko:K00563 |
| AIL03909.1 | infC | translation initiation factor IF-3 | -0.62 | -0.37 | -0.62 | NC | NC | NC | 1 | J | ko:K02520 |
| AIL05012.1 | glnQ | ABC transporter family protein | -0.02 | -0.21 | -0.31 | NC | NC | NC | 1 | E | ko:K02028 |
| AIL05330.1 | XK27_11280 | hypothetical protein DR75_1753 | 0.85 | 0.94 | 0.20 | NC | NC | NC | 1 | S | - |
| AIL03763.1 | lysC | aspartate kinase domain protein | 0.25 | -0.27 | -0.16 | NC | NC | NC | 1 | E | ko:K00928 |
| AIL05930.1 | - | SIS domain protein | 0.08 | -0.05 | -0.37 | NC | NC | NC | 1 | K | - |
| AIL03831.1 | recR | recombination protein RecR | 0.40 | -0.08 | 0.02 | NC | NC | NC | 1 | L | ko:K06187 |
| AIL04983.1 | - | short chain dehydrogenase family protein | -0.14 | 0.30 | 0.44 | NC | NC | NC | 1 | IQ | ko:K00046 |
| AIL04742.1 | mvk | mevalonate kinase | -0.36 | 0.10 | -0.59 | NC | NC | NC | 1 | I | ko:K00869 |
| AIL03844.1 | ytlR | lipid kinase, YegS//BmrU family protein | 0.30 | 0.08 | -0.19 | NC | NC | NC | 1 | I | - |
| AIL05841.1 | ykhA | thioesterase superfamily protein | -0.51 | -0.30 | -0.15 | NC | NC | NC | 1 | I | ko:K01073 |
| AIL05580.1 | pcp | pyroglutamyl-peptidase I | -0.23 | -0.24 | -0.33 | NC | NC | NC | 1 | O | ko:K01304 |
| AIL05534.1 | rbsD | rbsD / FucU transport family protein | 0.06 | -0.14 | -0.17 | NC | NC | NC | 1 | G | ko:K06726 |
| AIL05924.1 | - | alpha/beta hydrolase fold family protein | -0.08 | -0.06 | -0.12 | NC | NC | NC | 1 | I | - |
| AIL04219.1 | dnaB | replication initiation and membrane attachment family protein | -0.16 | 0.53 | 0.75 | NC | NC | NC | 1 | L | ko:K03346 |
| AIL03475.1 | citC | [citrate (pro-3S)-lyase] ligase | 0.84 | 0.46 | 0.25 | NC | NC | NC | 1 | H | ko:K01910 |
| AIL04891.1 | yleF | SIS domain protein | 0.45 | 0.48 | -0.01 | NC | NC | NC | 1 | K | - |
| AIL03420.1 | ydgI | nitroreductase | -0.14 | -0.31 | -0.10 | NC | NC | NC | 1 | C | - |
| AIL03627.1 | nagD | putative phosphatases involved in N-acetyl-glucosamine catabolism | 0.39 | -0.03 | 0.04 | NC | NC | NC | 1 | G | ko:K01101 |
| AIL03693.1 | metN | methionine import ATP-binding protein MetN 2 | 0.61 | 0.85 | 0.72 | NC | NC | NC | 1 | P | ko:K02071 |
| AIL04410.1 | glnQ | ABC transporter family protein | -0.42 | 0.19 | 0.14 | NC | NC | NC | 1 | E | ko:K02028 |
| AIL03444.1 | dacA | disA bacterial checkpoint controller nucleotide-binding family protein | -0.05 | 0.22 | 0.31 | NC | NC | NC | 1 | S | ko:K18672 |
| AIL03819.1 | - | glycosyl hydrolases 25 family protein | 0.32 | -0.03 | 0.17 | NC | NC | NC | 1 | M | - |
| AIL03713.1 | XK27_10035 | ABC transporter family protein | 0.08 | -0.19 | -0.09 | NC | NC | NC | 1 | V | ko:K06147 |
| AIL05442.1 | natB | ABC-2 transporter family protein | 0.28 | -0.36 | 0.06 | NC | NC | NC | 1 | CP | ko:K01992 |
| AIL03910.1 | XK27_04120 | amino acid metabolism family protein | 0.32 | 0.25 | 0.32 | NC | NC | NC | 1 | S | - |
| AIL03576.1 | scpB | segregation and condensation protein B | 0.07 | 0.15 | -0.11 | NC | NC | NC | 1 | D | ko:K06024 |
| AIL04567.1 | rex | oxidoreductase, NAD-binding Rossmann fold family protein | -0.50 | -0.75 | -0.90 | NC | NC | NC | 1 | K | ko:K01926 |
| AIL03808.1 | - | helix-turn-helix domain protein | 0.00 | 0.39 | -0.21 | NC | NC | NC | 1 | KT | ko:K07720 |
| AIL04302.1 | tilS | tRNA(Ile)-lysidine synthetase | -0.50 | 0.59 | -0.70 | NC | NC | NC | 1 | J | ko:K04075 |
| AIL04814.1 | dus | TIM-barrel, nifR3 family protein | -0.50 | -0.07 | -0.10 | NC | NC | NC | 1 | J | ko:K05540 |
| AIL03207.1 | - | putative cylI (plasmid) | -0.40 | -0.23 | -0.18 | NC | NC | NC | 1 | S | - |
| AIL03751.1 | - | sortase family protein | 0.25 | 0.22 | -0.82 | NC | NC | NC | 1 | M | ko:K07284 |
| AIL05453.1 | - | hypothetical protein DR75_2046 | 0.67 | -0.05 | 0.32 | NC | NC | NC | 1 | K | - |
| AIL05883.1 | - | hypothetical protein DR75_1519 | -0.34 | -0.48 | -0.52 | NC | NC | NC | 1 | - | - |
| AIL04201.1 | kup | potassium uptake protein | -0.62 | 0.10 | -0.13 | NC | NC | NC | 1 | P | ko:K03549 |
| AIL03264.1 | - | bacterial PH domain protein | -0.27 | -0.37 | -0.37 | NC | NC | NC | 1 | S | - |
| AIL04774.1 | pgmB | beta-phosphoglucomutase | -0.31 | -0.08 | -0.09 | NC | NC | NC | 1 | S | ko:K01838 |
| AIL03689.1 | rnhB | ribonuclease HII family protein | -0.73 | -0.27 | -0.26 | NC | NC | NC | 1 | L | ko:K03470 |
| AIL05781.1 | - | SIS domain protein | -0.32 | 0.08 | -0.37 | NC | NC | NC | 1 | M | ko:K19504 |
| AIL05940.1 | yfmL | hypothetical protein DR75_69 | -0.16 | 0.55 | -0.35 | NC | NC | NC | 1 | L | - |
| AIL04402.1 | - | helix-turn-helix family protein | 0.24 | -0.02 | -0.58 | NC | NC | NC | 1 | F | ko:K00852 |
| AIL04455.1 | treP | PTS system, trehalose-specific IIBC component | -0.01 | 0.63 | 0.32 | NC | NC | NC | 1 | G | ko:K02817 |
| AIL05459.1 | rpsS | ribosomal protein S19 | -0.38 | 0.00 | -0.53 | NC | NC | NC | 1 | J | ko:K02965 |
| AIL03617.1 | ykuJ | hypothetical protein DR75_1581 | -0.76 | -0.10 | -0.56 | NC | NC | NC | 1 | S | - |
| AIL04080.1 | sufS | cysteine desulfurase, SufS family protein | -0.49 | 0.32 | 0.03 | NC | NC | NC | 1 | E | ko:K11717 |
| AIL04081.1 | gloA | glyoxalase/Bleomycin resistance /Dioxygenase superfamily protein | 1.00 | 0.47 | 0.41 | NC | NC | NC | 1 | E | ko:K01759 |
| AIL04540.1 | - | hypothetical protein DR75_1674 | 0.13 | -0.31 | -0.07 | NC | NC | NC | 1 | - | - |
| AIL03329.1 | folP | dihydropteroate synthase | 0.75 | 0.07 | 0.18 | NC | NC | NC | 1 | H | ko:K00796 |
| AIL05225.1 | rplP | ribosomal protein L16 | -0.01 | -0.04 | -0.12 | NC | NC | NC | 1 | J | ko:K02878 |
| AIL03392.1 | XK27_02070 | nitroreductase family protein | -0.57 | -0.80 | -0.58 | NC | NC | NC | 1 | S | ko:K07078 |
| AIL04605.1 | rpsL | ribosomal protein S12 | 0.13 | -0.02 | -0.30 | NC | NC | NC | 1 | J | ko:K02950 |
| AIL04312.1 | WQ51_03320 | hypothetical protein DR75_2696 | 0.59 | 0.38 | 0.26 | NC | NC | NC | 1 | S | - |
| AIL05900.1 | rplU | ribosomal protein L21 | -0.09 | 0.04 | -0.21 | NC | NC | NC | 1 | J | ko:K02888 |
| AIL05714.1 | folA | dihydrofolate reductase | -0.09 | 0.15 | 0.06 | NC | NC | NC | 1 | H | ko:K00287 |
| AIL04221.1 | kduI | 4-deoxy-L-threo-5-hexosulose-uronate ketol-isomerase 1 | -0.71 | -0.09 | 0.27 | NC | NC | NC | 1 | G | ko:K01815 |
| AIL04159.1 | - | hypothetical protein DR75_2118 | 0.86 | 0.00 | 0.21 | NC | NC | NC | 1 | - | - |
| AIL04937.1 | adcC | ABC transporter family protein | 0.75 | 0.98 | -0.31 | NC | NC | NC | 1 | P | ko:K02074 |
| AIL05436.1 | mgsA | methylglyoxal synthase | 0.68 | 0.20 | 0.14 | NC | NC | NC | 1 | G | ko:K01734 |
| AIL05461.1 | rsmH | 16S rRNA (cytosine(1402)-N(4))-methyltransferase | -0.62 | -0.46 | -0.54 | NC | NC | NC | 1 | J | ko:K03438 |
| AIL04184.1 | perR | peroxide operon regulator | -0.01 | -0.52 | -0.88 | NC | NC | NC | 1 | P | ko:K09825 |
| AIL03402.1 | - | hypothetical protein DR75_693 | 0.37 | 0.34 | 0.41 | NC | NC | NC | 1 | S | - |
| AIL05808.1 | yhfI | metallo-beta-lactamase superfamily protein | 0.23 | 0.22 | 0.24 | NC | NC | NC | 1 | S | - |
| AIL05929.1 | - | putative lipoprotein | -0.99 | -0.41 | -0.29 | NC | NC | NC | 1 | - | - |
| AIL04900.1 | - | oxidoreductase, NAD-binding Rossmann fold family protein | -0.97 | -0.76 | -0.96 | NC | NC | NC | 1 | S | - |
| AIL04946.1 | yaaQ | hypothetical protein DR75_1456 | 0.95 | 0.52 | 0.72 | NC | NC | NC | 1 | S | - |
| AIL04048.1 | atpC | ATP synthase F1, epsilon subunit | -0.29 | -0.49 | -0.27 | NC | NC | NC | 1 | C | ko:K02114 |
| AIL04815.1 | yneT | coA binding domain protein | 0.80 | 0.62 | 0.24 | NC | NC | NC | 1 | S | ko:K06929 |
| AIL04397.1 | dapA | dihydrodipicolinate synthase | 0.02 | 0.08 | 0.19 | NC | NC | NC | 1 | E | ko:K01714 |
| AIL05206.1 | rplR | ribosomal protein L18 | -0.53 | -0.36 | -0.37 | NC | NC | NC | 1 | J | ko:K02881 |
| AIL03609.1 | rplK | ribosomal protein L11 | -0.21 | -0.03 | -0.47 | NC | NC | NC | 1 | J | ko:K02867 |
| AIL05579.1 | fur | ferric uptake regulation protein | -0.68 | -0.53 | -0.63 | NC | NC | NC | 1 | P | ko:K03711 |
| AIL04558.1 | budA | alpha-acetolactate decarboxylase | -0.49 | 0.79 | 0.45 | NC | NC | NC | 1 | H | ko:K01575 |
| AIL05231.1 | ytpR | putative tRNA binding domain protein | -0.08 | -0.50 | -0.32 | NC | NC | NC | 1 | J | ko:K06878 |
| AIL04570.1 | rpoZ | DNA-directed RNA polymerase, omega subunit | -0.07 | -0.12 | -0.28 | NC | NC | NC | 1 | K | ko:K03060 |
| AIL05661.1 | - | type I restriction modification DNA specificity domain protein | -0.54 | -0.24 | -0.39 | NC | NC | NC | 1 | V | ko:K01154 |
| AIL04344.1 | ndk | nucleoside diphosphate kinase B | 0.73 | 0.34 | 0.33 | NC | NC | NC | 1 | F | ko:K00940 |
| AIL03899.1 | - | GDSL-like Lipase/Acylhydrolase family protein | 0.98 | 0.33 | 0.50 | NC | NC | NC | 1 | E | - |
| AIL03798.1 | rplV | ribosomal protein L22 | -0.12 | 0.16 | -0.06 | NC | NC | NC | 1 | J | ko:K02890 |
| AIL05041.1 | dapF | diaminopimelate epimerase | -0.24 | -0.03 | 0.14 | NC | NC | NC | 1 | E | ko:K01778 |
| AIL03959.1 | ctc | ribosomal protein L25, Ctc-form | 0.92 | 0.27 | 0.43 | NC | NC | NC | 1 | J | ko:K02897 |
| AIL03639.1 | - | bacterial extracellular solute-binding s, 5 Middle family protein | -0.45 | 0.18 | 0.38 | NC | NC | NC | 1 | E | ko:K15580 |
| AIL04628.1 | trpS | tryptophan--tRNA ligase | -0.29 | -0.09 | -0.11 | NC | NC | NC | 1 | J | ko:K01867 |
| AIL04953.1 | XK27_03960 | hypothetical protein DR75_937 | -0.10 | -0.29 | -0.40 | NC | NC | NC | 1 | S | - |
| AIL05782.1 | rsmC | tRNA (Uracil-5-)-methyltransferase family protein | 0.10 | -0.13 | -0.02 | NC | NC | NC | 1 | J | ko:K00564 |
| AIL04632.1 | sbcD | exonuclease SbcCD, D subunit | -0.91 | -0.67 | -0.02 | NC | NC | NC | 1 | L | ko:K03547 |
| AIL03174.1 | - | hypothetical protein DR75_2906 (plasmid) | 0.09 | -0.28 | -0.54 | NC | NC | NC | 1 | - | - |
| AIL05290.1 | - | ABC transporter family protein | -0.85 | -0.55 | -0.35 | NC | NC | NC | 1 | V | ko:K02003 |
| AIL04079.1 | ldhB | L-lactate dehydrogenase | 0.31 | -0.31 | -0.04 | NC | NC | NC | 1 | C | ko:K00016 |
| AIL03922.1 | - | cell division ZapA family protein | -0.43 | -0.38 | -0.18 | NC | NC | NC | 1 | D | ko:K09888 |
| AIL03276.1 | ytgP | polysaccharide biosynthesis family protein | -0.09 | -0.81 | 0.37 | NC | NC | NC | 1 | S | ko:K03328 |
| AIL04471.1 | proC | pyrroline-5-carboxylate reductase | 0.63 | 0.61 | 0.54 | NC | NC | NC | 1 | E | ko:K00286 |
| AIL05007.1 | ysnB | phosphodiesterase, family protein | 0.93 | 0.89 | 0.61 | NC | NC | NC | 1 | S | ko:K07095 |
| AIL03596.1 | rplD | 50S ribosomal protein L4 | -0.45 | -0.21 | -0.30 | NC | NC | NC | 1 | J | ko:K02926 |
| AIL05621.1 | whiA | hypothetical protein DR75_2721 | -0.85 | -0.01 | 0.06 | NC | NC | NC | 1 | K | ko:K09762 |
| AIL03181.1 | - | hypothetical protein DR75_2890 (plasmid) | 0.41 | 0.75 | 0.80 | NC | NC | NC | 1 | - | - |
| AIL03651.1 | gntK | hypothetical protein DR75_1944 | -0.42 | 0.16 | -0.17 | NC | NC | NC | 1 | G | ko:K00851 |
| AIL03484.1 | ypmS | hypothetical protein DR75_683 | 0.08 | -0.40 | -0.31 | NC | NC | NC | 1 | S | - |
| AIL05207.1 | - | hypothetical protein DR75_1226 | 0.30 | -0.03 | 0.28 | NC | NC | NC | 1 | S | - |
| AIL05492.1 | - | beta-lactamase family protein | 0.12 | -0.22 | -0.08 | NC | NC | NC | 1 | V | - |
| AIL03347.1 | - | 2-dehydro-3-deoxyphosphogluconate aldolase | 0.44 | 0.74 | 0.41 | NC | NC | NC | 1 | S | ko:K17463 |
| AIL04016.1 | - | 2-dehydro-3-deoxyphosphogluconate aldolase/4-hydroxy-2-oxoglutarate aldolase family protein | 0.80 | 0.08 | 0.07 | NC | NC | NC | 1 | G | ko:K01625 |
| AIL03458.1 | peb1A | bacterial extracellular solute-binding s, 3 family protein | 0.61 | -0.05 | 0.09 | NC | NC | NC | 1 | ET | ko:K10039 |
| AIL04426.1 | pheA | prephenate dehydratase family protein | -0.85 | -0.64 | -0.93 | NC | NC | NC | 1 | E | ko:K04518 |
| AIL04039.1 | - | periplasmic binding s and sugar binding domain of LacI family protein | 0.61 | 0.09 | 0.18 | NC | NC | NC | 1 | K | ko:K02529 |
| AIL04537.1 | tcaA | hypothetical protein DR75_546 | 0.05 | 0.64 | -0.70 | NC | NC | NC | 1 | S | ko:K21463 |
| AIL04684.1 | lytT | response regulator | 0.50 | 0.17 | 0.10 | NC | NC | NC | 1 | K | ko:K07705 |
| AIL04509.1 | - | ATP-dependent DNA helicase RecQ | 0.99 | -0.06 | -0.28 | NC | NC | NC | 1 | L | ko:K03654 |
| AIL05803.1 | truA | tRNA pseudouridine(38-40) synthase | -0.24 | -0.21 | -0.08 | NC | NC | NC | 1 | J | ko:K06173 |
| AIL03335.1 | ccpN | CBS domain protein | -0.08 | 0.13 | -0.10 | NC | NC | NC | 1 | K | - |
| AIL05205.1 | rpoN | RNA polymerase sigma-54 factor | -0.29 | -0.34 | -0.29 | NC | NC | NC | 1 | K | ko:K03092 |
| AIL04012.1 | spoU | RNA 2'-O ribose methyltransferase substrate binding family protein | 0.33 | 0.37 | 0.00 | NC | NC | NC | 1 | J | ko:K03437 |
| AIL05868.1 | ylmH | S4 domain protein | 0.50 | 0.26 | 0.13 | NC | NC | NC | 1 | S | - |
| AIL04825.1 | yqeM | methyltransferase domain protein | -0.71 | -0.10 | -0.02 | NC | NC | NC | 1 | Q | - |
| AIL03963.1 | - | transcriptional regulator, GntR family | 0.35 | -0.06 | 0.14 | NC | NC | NC | 1 | K | - |
| AIL05656.1 | ywlC | tRNA threonylcarbamoyl adenosine modification protein, Sua5/YciO/YrdC/YwlC family | -0.17 | 0.16 | -0.27 | NC | NC | NC | 1 | J | ko:K07566 |
| AIL04559.1 | yxlJ | DNA-3-methyladenine glycosylase family protein | -0.81 | -0.84 | -0.32 | NC | NC | NC | 1 | L | ko:K03652 |
| AIL04027.1 | - | beta-eliminating lyase family protein | 0.01 | 0.20 | -0.17 | NC | NC | NC | 1 | - | - |
| AIL04052.1 | mprF | hypothetical protein DR75_87 | -0.19 | 0.86 | 0.52 | NC | NC | NC | 1 | S | ko:K14205 |
| AIL05289.1 | pi112 | hypothetical protein DR75_1661 | 0.41 | -0.06 | 0.02 | NC | NC | NC | 1 | L | - |
| AIL04321.1 | - | endonuclease/exonuclease/phosphatase family protein | -0.02 | -0.02 | -0.67 | NC | NC | NC | 1 | S | - |
| AIL03388.1 | msrB | peptide methionine sulfoxide reductase MsrB | 0.79 | 0.12 | -0.11 | NC | NC | NC | 1 | O | ko:K07305 |
| AIL04757.1 | - | hypothetical protein DR75_1510 | 0.80 | -0.27 | 0.28 | NC | NC | NC | 1 | - | - |
| AIL04162.1 | dapB | dihydrodipicolinate reductase | -0.66 | -0.16 | -0.12 | NC | NC | NC | 1 | E | ko:K00215 |
| AIL05457.1 | rpmE2 | ribosomal protein L31 | -0.70 | 0.45 | 0.06 | NC | NC | NC | 1 | J | ko:K02909 |
| AIL03278.1 | yeeN | DNA-binding regulatory, YebC/PmpR family protein | -0.30 | -0.68 | -0.55 | NC | NC | NC | 1 | K | - |
| AIL05337.1 | yacL | PIN domain protein | 0.20 | -0.26 | -0.20 | NC | NC | NC | 1 | S | - |
| AIL04539.1 | ygaC | hypothetical protein DR75_2648 | 0.43 | 0.34 | 0.27 | NC | NC | NC | 1 | J | ko:K07586 |
| AIL03563.1 | nrdI | nrdI Flavodoxin like family protein | 0.11 | -0.05 | -0.13 | NC | NC | NC | 1 | F | ko:K03647 |
| AIL05663.1 | XK27_09445 | hypothetical protein DR75_2664 | 0.63 | 0.36 | 0.40 | NC | NC | NC | 1 | S | - |
| AIL05280.1 | - | hypothetical protein DR75_789 | 0.94 | -0.07 | 0.03 | NC | NC | NC | 1 | S | - |
| AIL03215.1 | panD | aspartate 1-decarboxylase | -0.44 | -0.35 | -0.38 | NC | NC | NC | 1 | H | ko:K01579 |
| AIL05301.1 | srlB | PTS system glucitol/sorbitol-specific IIA component family protein | 0.11 | 0.45 | 0.43 | NC | NC | NC | 1 | G | ko:K02781 |
| AIL04786.1 | - | hypothetical protein DR75_1451 | -0.33 | -0.01 | 0.23 | NC | NC | NC | 1 | S | ko:K02068 |
| AIL03274.1 | hlyX | transporter associated domain protein | -0.51 | -0.36 | -0.35 | NC | NC | NC | 1 | S | ko:K03699 |
| AIL05450.1 | def1 | peptide deformylase | -0.23 | 0.41 | 0.21 | NC | NC | NC | 1 | J | ko:K01462 |
| AIL05540.1 | - | universal stress family protein | 0.67 | 0.51 | 0.46 | NC | NC | NC | 1 | T | - |
| AIL04803.1 | purK | D-ala D-ala ligase family protein | -0.21 | -0.13 | -0.14 | NC | NC | NC | 1 | F | ko:K01589 |
| AIL04882.1 | rplT | ribosomal protein L20 | -0.33 | -0.70 | -0.78 | NC | NC | NC | 1 | J | ko:K02887 |
| AIL04746.1 | atpF | ATP synthase F0, B subunit | -0.36 | -0.24 | -0.21 | NC | NC | NC | 1 | C | ko:K02109 |
| AIL03849.1 | - | NADPH-dependent nitro/flavin reductase | 0.50 | -0.06 | 0.18 | NC | NC | NC | 1 | C | - |
| AIL03664.1 | - | histidine phosphatase super family protein | 0.35 | 0.11 | -0.17 | NC | NC | NC | 1 | G | ko:K15634 |
| AIL03656.1 | srtA | sortase family protein | 0.68 | 0.19 | 0.13 | NC | NC | NC | 1 | M | ko:K07284 |
| AIL03846.1 | - | universal stress family protein | -0.03 | -0.49 | -0.34 | NC | NC | NC | 1 | T | - |
| AIL03258.1 | ycaM | inner membrane transporter ycaM | -0.27 | -0.47 | -0.52 | NC | NC | NC | 1 | E | - |
| AIL03515.1 | yrzL | hypothetical protein DR75_267 | 0.43 | 0.20 | -0.12 | NC | NC | NC | 1 | S | - |
| AIL05601.1 | nanE | putative N-acetylmannosamine-6-phosphate 2-epimerase | -0.42 | 0.12 | 0.32 | NC | NC | NC | 1 | G | ko:K01788 |
| AIL03773.1 | yeaO | hypothetical protein DR75_1477 | -0.03 | 0.09 | -0.25 | NC | NC | NC | 1 | S | - |
| AIL03441.1 | - | ankyrin repeat family domain protein | 0.88 | 0.02 | 0.12 | NC | NC | NC | 1 | S | ko:K06867 |
| AIL04365.1 | hit | HIT domain protein | 0.29 | 0.60 | 0.43 | NC | NC | NC | 1 | FG | ko:K02503 |
| AIL05779.1 | - | zinc-binding dehydrogenase family protein | -0.55 | -0.01 | -0.06 | NC | NC | NC | 1 | C | ko:K00001 |
| AIL04644.1 | - | 3-demethylubiquinone-9 3-methyltransferase family protein | 0.30 | -0.32 | 0.33 | NC | NC | NC | 1 | S | ko:K04750 |
| AIL05622.1 | ylbN | hypothetical protein DR75_106 | 0.11 | -0.22 | -0.19 | NC | NC | NC | 1 | S | ko:K07040 |
| AIL04259.1 | ylxR | hypothetical protein DR75_334 | 0.04 | 0.15 | 0.07 | NC | NC | NC | 1 | K | ko:K07742 |
| AIL05839.1 | - | peptidase propeptide and YPEB domain protein | -0.14 | -0.44 | -0.34 | NC | NC | NC | 1 | - | - |
| AIL05036.1 | yvqF | hypothetical protein DR75_1608 | 0.21 | -0.20 | -0.12 | NC | NC | NC | 1 | S | ko:K11622 |
| AIL05136.1 | lexA | repressor LexA | -0.57 | 0.07 | 0.18 | NC | NC | NC | 1 | K | ko:K01356 |
| AIL03366.1 | paiA | protease synthase and sporulation negative regulatory protein PAI 1 | 0.03 | -0.16 | 0.34 | NC | NC | NC | 1 | K | ko:K22441 |
| AIL04084.1 | rlmH | rRNA large subunit m3Psi methyltransferase RlmH | -0.13 | -0.89 | -0.77 | NC | NC | NC | 1 | J | ko:K00783 |
| AIL04155.1 | rbfA | ribosome-binding factor A | 0.36 | 0.03 | -0.25 | NC | NC | NC | 1 | J | ko:K02834 |
| AIL04440.1 | dtpT | amino acid/peptide transporter family protein | -0.33 | -0.35 | -0.26 | NC | NC | NC | 1 | U | ko:K03305 |
| AIL03694.1 | thiM | hydroxyethylthiazole kinase | 0.55 | 0.86 | 0.64 | NC | NC | NC | 1 | H | ko:K00878 |
| AIL03684.1 | - | tyrosine phosphatase family protein | -0.73 | -0.30 | -0.16 | NC | NC | NC | 1 | T | ko:K01104 |
| AIL03768.1 | - | hypothetical protein DR75_1648 | 0.35 | 0.08 | -0.21 | NC | NC | NC | 1 | - | - |
| AIL05019.1 | manO | hypothetical protein DR75_2072 | 0.59 | 0.65 | 0.38 | NC | NC | NC | 1 | S | - |
| AIL04062.1 | - | linear amide C-N hydrolase, choloylglycine hydrolase family protein | 0.54 | 0.76 | 0.63 | NC | NC | NC | 1 | M | ko:K01442 |
| AIL05314.1 | - | putative lipoprotein | -0.18 | -0.71 | -0.30 | NC | NC | NC | 1 | - | - |
| AIL04026.1 | - | hypothetical protein DR75_1500 | 0.24 | -0.79 | -0.91 | NC | NC | NC | 1 | S | - |
| AIL05816.1 | folE | GTP cyclohydrolase I | 0.46 | 0.85 | 0.82 | NC | NC | NC | 1 | F | ko:K00950 |
| AIL03449.1 | XK27_00220 | alpha/beta hydrolase family protein | 0.62 | 0.36 | 0.25 | NC | NC | NC | 1 | S | ko:K06999 |
| AIL03767.1 | tagH | lysM domain protein | 0.06 | -0.61 | -0.87 | NC | NC | NC | 1 | GM | ko:K09693 |
| AIL05702.1 | - | glucosyl transferase GtrII family protein | -0.59 | 0.13 | 0.11 | NC | NC | NC | 1 | S | - |
| AIL04406.1 | - | hypothetical protein DR75_1386 | 0.09 | -0.19 | 0.01 | NC | NC | NC | 1 | - | - |
| AIL03952.1 | - | ABC transporter family protein | 0.81 | -0.47 | -0.83 | NC | NC | NC | 1 | V | ko:K06147 |
| AIL04311.1 | XK27_07850 | CBS domain protein | 0.41 | 0.99 | -0.74 | NC | NC | NC | 1 | S | - |
| AIL03998.1 | xerC | tyrosine recombinase XerC | -0.06 | 0.03 | -0.27 | NC | NC | NC | 1 | D | ko:K03733 |
| AIL04954.1 | XK27_10395 | hypothetical protein DR75_1811 | 0.58 | 0.45 | 0.60 | NC | NC | NC | 1 | S | - |
| AIL04191.1 | yunF | hypothetical protein DR75_2358 | 0.34 | 0.06 | 0.34 | NC | NC | NC | 1 | S | - |
| AIL05159.1 | recF | DNA replication and repair RecF family protein | -0.70 | 0.76 | 0.55 | NC | NC | NC | 1 | L | ko:K03629 |
| AIL03785.1 | dnaD | DnaD domain protein | -0.43 | -0.52 | -0.48 | NC | NC | NC | 1 | L | ko:K02086 |
| AIL04457.1 | - | alpha/beta hydrolase fold family protein | -0.01 | -0.52 | -0.45 | NC | NC | NC | 1 | I | - |
| AIL05250.1 | mdlB | ABC transporter family protein | 0.19 | 0.43 | -0.78 | NC | NC | NC | 1 | V | ko:K06147 |
| AIL05401.1 | rliB | periplasmic binding s and sugar binding domain of LacI family protein | -0.03 | -0.04 | 0.55 | NC | NC | NC | 1 | K | - |
| AIL05181.1 | M1-1000 | response regulator | 0.15 | 0.33 | -0.15 | NC | NC | NC | 1 | K | ko:K02475 |
| AIL03569.1 | nt5e | HAD hydrolase, IA, variant 1 family protein | 0.16 | -0.11 | -0.36 | NC | NC | NC | 1 | S | ko:K01091 |
| AIL03303.1 | feoA | feoA domain protein | -0.34 | 0.41 | -0.02 | NC | NC | NC | 1 | P | ko:K04758 |
| AIL05267.1 | yleB | hypothetical protein DR75_1183 | -0.29 | 0.54 | -0.22 | NC | NC | NC | 1 | S | ko:K07106 |
| AIL03323.1 | lytR | cell envelope-related function transcriptional attenuator common domain protein | 0.17 | -0.11 | 0.24 | NC | NC | NC | 1 | IK | - |
| AIL05735.1 | - | HAD hydrolase, IIB family protein | -0.23 | -0.05 | 0.05 | NC | NC | NC | 1 | S | - |
| AIL03777.1 | - | hypothetical protein DR75_2657 | -0.41 | 0.10 | -0.05 | NC | NC | NC | 1 | - | - |
| AIL04664.1 | - | ftsX-like permease family protein | 0.58 | -0.52 | 0.17 | NC | NC | NC | 1 | V | ko:K11636 |
| AIL03196.1 | - | putative lipoprotein (plasmid) | 0.06 | -0.53 | -0.10 | NC | NC | NC | 1 | CO | - |
| AIL03742.1 | - | merR regulatory family protein | 0.31 | 0.60 | 0.25 | NC | NC | NC | 1 | K | - |
| AIL03775.1 | - | sulfatase family protein | -0.39 | 0.05 | -0.70 | NC | NC | NC | 1 | M | - |
| AIL05758.1 | - | peptidase T | -0.80 | -0.44 | -0.89 | NC | NC | NC | 1 | E | ko:K01258 |
| AIL04947.1 | nrdR | transcriptional regulator NrdR | 0.13 | -0.30 | -0.08 | NC | NC | NC | 1 | K | ko:K07738 |
| AIL04095.1 | punA | purine nucleoside phosphorylase I, inosine and guanosine-specific | 0.19 | 0.30 | 0.15 | NC | NC | NC | 1 | F | ko:K00772 |
| AIL03437.1 | argR1 | hypothetical protein DR75_2141 | 0.74 | -0.26 | -0.05 | NC | NC | NC | 1 | K | ko:K03402 |
| AIL04789.1 | dut | dUTPase family protein | -0.01 | 0.21 | 0.19 | NC | NC | NC | 1 | F | ko:K01520 |
| AIL04009.1 | fabZ | beta-hydroxyacyl-(acyl-carrier-protein) dehydratase FabZ | -0.66 | -0.24 | -0.42 | NC | NC | NC | 1 | I | ko:K02372 |
| AIL04230.1 | tpx | putative thiol peroxidase | 0.86 | 0.65 | 0.54 | NC | NC | NC | 1 | O | ko:K11065 |
| AIL05750.1 | sepF | cell division protein sepF | -0.62 | -0.35 | -0.54 | NC | NC | NC | 1 | D | ko:K09772 |
| AIL04812.1 | rpsQ | 30S ribosomal protein S17 | -0.47 | -0.46 | -0.63 | NC | NC | NC | 1 | J | ko:K02961 |
| AIL04309.1 | rplX | ribosomal protein L24 | 0.06 | 0.20 | -0.16 | NC | NC | NC | 1 | J | ko:K02895 |
| AIL04544.1 | - | hypothetical protein DR75_1400 | -0.60 | -0.41 | -0.14 | NC | NC | NC | 1 | - | - |
| AIL05910.1 | cobQ | cobB/CobQ-like glutamine amidotransferase domain protein | 0.01 | -0.15 | 0.02 | NC | NC | NC | 1 | S | ko:K07009 |
| AIL03926.1 | cutC | cutC family protein | -0.27 | -0.49 | -0.28 | NC | NC | NC | 1 | P | ko:K06201 |
| AIL04621.1 | copZ | heavy-metal-associated domain protein | 0.88 | 0.41 | 0.15 | NC | NC | NC | 1 | P | - |
| AIL04220.1 | ytzB | hypothetical protein DR75_1765 | 0.53 | 0.23 | 0.10 | NC | NC | NC | 1 | S | - |
| AIL05232.1 | - | dihydrofolate reductase | 0.37 | 0.24 | 0.15 | NC | NC | NC | 1 | H | - |
| AIL03130.1 | repA | putative prgW (plasmid) | -0.74 | -0.68 | -0.60 | NC | NC | NC | 1 | S | - |
| AIL05000.1 | ltrC | hypothetical protein DR75_2588 | 0.01 | 0.49 | 0.19 | NC | NC | NC | 1 | I | - |
| AIL04910.1 | XK27_08845 | ABC transporter family protein | -0.30 | -0.59 | -0.37 | NC | NC | NC | 1 | S | ko:K05833 |
| AIL05004.1 | ctsR | transcriptional regulator CtsR | 0.34 | -0.16 | -0.20 | NC | NC | NC | 1 | K | ko:K03708 |
| AIL03482.1 | - | hypothetical protein DR75_210 | -0.14 | 0.26 | -0.03 | NC | NC | NC | 1 | S | ko:K07010 |
| AIL03841.1 | - | PTS system mannose/fructose/sorbose IID component family protein | 0.12 | -0.51 | -0.61 | NC | NC | NC | 1 | G | ko:K02796 |
| AIL04408.1 | phnA | phnA Zinc-Ribbon family protein | -0.92 | -0.49 | -0.52 | NC | NC | NC | 1 | P | ko:K06193 |
| AIL04898.1 | yabA | hypothetical protein DR75_1453 | 0.34 | -0.28 | -0.38 | NC | NC | NC | 1 | L | - |
| AIL05633.1 | - | acetyltransferase family protein | -0.58 | -0.39 | -0.13 | NC | NC | NC | 1 | J | - |
| AIL03975.1 | - | bacterial transcriptional regulator family protein | 0.04 | -0.14 | -0.50 | NC | NC | NC | 1 | K | ko:K19333 |
| AIL05352.1 | yfdH | glycosyl transferase 2 family protein | -0.44 | -0.06 | 0.01 | NC | NC | NC | 1 | M | ko:K12999 |
| AIL03490.1 | ptbA | PTS system, glucose subfamily, IIA component domain protein | 0.86 | 0.51 | 0.49 | NC | NC | NC | 1 | G | ko:K02755 |
| AIL05614.1 | panC | pantoate--beta-alanine ligase | 0.13 | -0.20 | -0.81 | NC | NC | NC | 1 | H | ko:K01918 |
| AIL04519.1 | - | amino acid permease family protein | -0.19 | -0.80 | -0.25 | NC | NC | NC | 1 | E | ko:K20265 |
| AIL04711.1 | - | hypothetical protein DR75_2613 | 0.52 | 0.71 | 0.65 | NC | NC | NC | 1 | - | - |
| AIL03506.1 | - | glycine cleavage H-family protein | -0.35 | 0.91 | 0.42 | NC | NC | NC | 1 | E | ko:K02437 |
| AIL05032.1 | nth | endonuclease III | -0.55 | -0.29 | -0.68 | NC | NC | NC | 1 | L | ko:K10773 |
| AIL04091.1 | groS | 10 kDa chaperonin | 0.16 | -0.06 | -0.30 | NC | NC | NC | 1 | O | ko:K04078 |
| AIL03275.1 | nnrD | yjeF-related protein | 0.24 | 0.03 | 0.00 | NC | NC | NC | 1 | H | ko:K17758 |
| AIL03606.1 | ung | uracil-DNA glycosylase | -0.29 | -0.04 | 0.00 | NC | NC | NC | 1 | L | ko:K03648 |
| AIL04577.1 | arnC | glycosyl transferase 2 family protein | -0.45 | -0.47 | -0.10 | NC | NC | NC | 1 | M | ko:K00786 |
| AIL03615.1 | - | glycosyl Hydrolase Family 88 family protein | -0.14 | 0.86 | 0.32 | NC | NC | NC | 1 | S | ko:K18581 |
| AIL04214.1 | - | UTRA domain protein | -0.43 | -0.08 | -0.25 | NC | NC | NC | 1 | K | ko:K03710 |
| AIL04747.1 | - | hypothetical protein DR75_1089 | 0.35 | -0.17 | -0.31 | NC | NC | NC | 1 | F | - |
| AIL03397.1 | - | helix-turn-helix family protein | 0.50 | -0.04 | -0.07 | NC | NC | NC | 1 | K | - |
| AIL04761.1 | xerD | tyrosine recombinase XerD | 0.45 | 0.07 | -0.04 | NC | NC | NC | 1 | D | ko:K04763 |
| AIL04924.1 | - | acetyltransferase family protein | 0.37 | 0.06 | 0.03 | NC | NC | NC | 1 | K | ko:K03827 |
| AIL03562.1 | agaS | SIS domain protein | -0.43 | -0.23 | -0.25 | NC | NC | NC | 1 | M | ko:K02082 |
| AIL05132.1 | ypjC | hypothetical protein DR75_558 | -0.51 | -0.22 | -0.35 | NC | NC | NC | 1 | S | - |
| AIL04858.1 | oatA | acyltransferase family protein | -0.20 | -0.04 | 0.17 | NC | NC | NC | 1 | I | - |
| AIL04778.1 | rimP | hypothetical protein DR75_332 | -0.39 | -0.36 | -0.14 | NC | NC | NC | 1 | S | ko:K09748 |
| AIL05819.1 | - | helix-turn-helix family protein | 0.13 | 0.14 | -0.08 | NC | NC | NC | 1 | K | - |
| AIL03981.1 | - | crp-like helix-turn-helix domain protein | -0.56 | -0.07 | -0.17 | NC | NC | NC | 1 | K | - |
| AIL05071.1 | - | phosphoglycerate mutase 1 family protein | -0.31 | -0.79 | -0.30 | NC | NC | NC | 1 | G | ko:K01834 |
| AIL05474.1 | - | bacterial regulatory s, tetR family protein | -0.45 | -0.59 | -0.06 | NC | NC | NC | 1 | K | - |
| AIL03115.1 | - | addiction module toxin, RelE/StbE family protein (plasmid) | 0.53 | -0.31 | -0.27 | NC | NC | NC | 1 | S | ko:K19157 |
| AIL03310.1 | - | helix-turn-helix family protein | -0.19 | 0.28 | -0.05 | NC | NC | NC | 1 | K | - |
| AIL03153.1 | - | hypothetical protein DR75_2888 (plasmid) | -0.49 | -0.02 | -0.07 | NC | NC | NC | 1 | KLT | ko:K00703 |
| AIL04755.1 | rsmI | ribosomal RNA small subunit methyltransferase I | 0.06 | -0.48 | -0.47 | NC | NC | NC | 1 | H | ko:K07056 |
| AIL04736.1 | ecsA | ABC transporter family protein | -0.69 | 0.16 | 0.14 | NC | NC | NC | 1 | V | ko:K01990 |
| AIL05241.1 | xylR | ROK family protein | -0.44 | -0.28 | -0.01 | NC | NC | NC | 1 | GK | - |
| AIL03501.1 | citX | holo-ACP synthase CitX | 0.71 | -0.02 | -0.58 | NC | NC | NC | 1 | HI | ko:K05964 |
| AIL05344.1 | ydcN1 | helix-turn-helix family protein | -0.14 | -0.11 | -0.03 | NC | NC | NC | 1 | K | - |
| AIL04795.1 | argR2 | arginine repressor | -0.24 | -0.45 | -0.75 | NC | NC | NC | 1 | K | ko:K03402 |
| AIL03535.1 | - | peptidase M20/M25/M40 family protein | 0.77 | -0.86 | -0.26 | NC | NC | NC | 1 | E | - |
| AIL04525.1 | - | acetyltransferase family protein | 0.07 | 0.42 | -0.35 | NC | NC | NC | 1 | J | ko:K03790 |
| AIL05234.1 | ydiB | tRNA threonylcarbamoyl adenosine modification protein YjeE | -0.04 | 0.08 | 0.09 | NC | NC | NC | 1 | S | ko:K06925 |
| AIL05127.1 | hflX | GTP-binding protein HflX | 0.38 | -0.19 | -0.32 | NC | NC | NC | 1 | S | ko:K03665 |
| AIL03828.1 | mtlR | deoR-like helix-turn-helix domain protein | -0.24 | -0.20 | 0.29 | NC | NC | NC | 1 | K | ko:K03483 |
| AIL04400.1 | - | phenazine biosynthesis, PhzF family protein | -0.40 | 0.53 | -0.72 | NC | NC | NC | 1 | S | - |
| AIL04293.1 | purR1 | bacterial regulatory s, lacI family protein | -0.46 | 0.39 | -0.06 | NC | NC | NC | 1 | K | ko:K02529 |
| AIL04730.1 | yhaO | calcineurin-like phosphoesterase family protein | 0.02 | -0.35 | 0.01 | NC | NC | NC | 1 | L | ko:K03547 |
| AIL05367.1 | - | glycosyl transferase 2 family protein | -0.37 | -0.33 | -0.04 | NC | NC | NC | 1 | S | - |
| AIL03616.1 | - | hypothetical protein DR75_2147 | -0.36 | -0.43 | 0.10 | NC | NC | NC | 1 | S | - |
| AIL03732.1 | rimI | ribosomal-protein-alanine acetyltransferase | 0.32 | 0.11 | -0.30 | NC | NC | NC | 1 | K | ko:K03789 |
| AIL03123.1 | - | lactococcin-G-processing and transport ATP-binding protein LagD (plasmid) | 0.01 | -0.42 | -0.18 | NC | NC | NC | 1 | V | ko:K20344 |
| AIL05722.1 | yurR | FAD dependent oxidoreductase family protein | -0.09 | -0.13 | -0.01 | NC | NC | NC | 1 | E | ko:K00285 |
| AIL04212.1 | ydaM | glycosyl transferase 2 family protein | -0.25 | 0.24 | 0.05 | NC | NC | NC | 1 | M | - |
| AIL03414.1 | - | helix-turn-helix family protein | 0.74 | 0.35 | 0.31 | NC | NC | NC | 1 | K | - |
| AIL05418.1 | trxA | thioredoxin | -0.18 | 0.66 | 0.40 | NC | NC | NC | 1 | O | ko:K03671 |
| AIL04050.1 | yloU | asp23 family protein | 0.13 | -0.14 | 0.10 | NC | NC | NC | 1 | S | - |
| AIL04966.1 | acyP | acylphosphatase | 0.25 | 0.16 | 0.48 | NC | NC | NC | 1 | C | ko:K01512 |
| AIL05411.1 | rpmB | ribosomal protein L28 | -0.32 | -0.33 | -0.25 | NC | NC | NC | 1 | J | ko:K02902 |
| AIL05824.1 | citD | citrate lyase acyl carrier protein | 0.95 | 0.92 | 0.66 | NC | NC | NC | 1 | C | ko:K01646 |
| AIL03593.1 | - | universal stress family protein | -0.88 | 0.07 | -0.33 | NC | NC | NC | 1 | T | - |
| AIL03668.1 | tdk | thymidine kinase family protein | -0.14 | 0.03 | -0.09 | NC | NC | NC | 1 | F | ko:K00857 |
| AIL04322.1 | - | acetyltransferase family protein | 0.06 | 0.00 | -0.02 | NC | NC | NC | 1 | S | ko:K06975 |
| AIL05583.1 | yneR | putative yneR | 0.55 | 0.38 | 0.19 | NC | NC | NC | 1 | S | - |
| AIL04468.1 | yneF | hypothetical protein DR75_732 | 0.16 | -0.43 | -0.26 | NC | NC | NC | 1 | S | ko:K09976 |
| AIL05586.1 | XK27_02735 | marR family protein | -0.13 | -0.63 | -0.84 | NC | NC | NC | 1 | K | - |
| AIL04138.1 | stp | serine/threonine phosphatase stp | 0.36 | 0.33 | 0.61 | NC | NC | NC | 1 | T | ko:K20074 |
| AIL03219.1 | pstA | phosphate ABC transporter, permease protein PstA | -0.55 | -0.41 | -0.35 | NC | NC | NC | 1 | P | ko:K02038 |
| AIL04278.1 | gatC | aspartyl/glutamyl-tRNA(Asn/Gln) amidotransferase, C subunit | -0.08 | 0.34 | 0.01 | NC | NC | NC | 1 | J | ko:K02435 |
| AIL03262.1 | nusB | transcription antitermination factor NusB | 0.13 | 0.09 | 0.05 | NC | NC | NC | 1 | K | ko:K03625 |
| AIL05753.1 | - | hypothetical protein DR75_1559 | 0.27 | -0.30 | -0.27 | NC | NC | NC | 1 | - | - |
| AIL05148.1 | - | hypothetical protein DR75_1040 | -0.02 | 0.02 | 0.17 | NC | NC | NC | 1 | - | - |
| AIL03761.1 | yjqA | bacterial PH domain protein | 0.11 | 0.88 | 0.64 | NC | NC | NC | 1 | S | - |
| AIL05493.1 | iunH3 | inosine-uridine preferring nucleoside hydrolase family protein | -0.64 | -0.41 | 0.40 | NC | NC | NC | 1 | F | ko:K01239 |
| AIL03546.1 | rsmE | RNA methyltransferase, RsmE family protein | -0.72 | -0.42 | -0.31 | NC | NC | NC | 1 | J | ko:K09761 |
| AIL04744.1 | cobB | sir2 family protein | 0.65 | 0.99 | 0.48 | NC | NC | NC | 1 | K | ko:K12410 |
| AIL03272.1 | rpsI | ribosomal S9/S16 family protein | -0.28 | 0.14 | -0.20 | NC | NC | NC | 1 | J | ko:K02996 |
| AIL04715.1 | mrnC | ribonuclease III domain protein | 0.05 | -0.27 | -0.47 | NC | NC | NC | 1 | J | ko:K11145 |
| AIL03415.1 | cah | eukaryotic-type carbonic anhydrase family protein | 0.55 | 0.53 | 0.14 | NC | NC | NC | 1 | P | ko:K01674 |
| AIL05476.1 | ybjQ | heavy-metal-binding family protein | 0.34 | 0.38 | 0.49 | NC | NC | NC | 1 | S | - |
| AIL03923.1 | - | peptidase M48 family protein | -0.25 | 0.11 | -0.03 | NC | NC | NC | 1 | O | - |
| AIL05585.1 | - | hypothetical protein DR75_388 | -0.02 | 0.30 | 0.30 | NC | NC | NC | 1 | S | - |
| AIL05219.1 | - | ABC transporter family protein | 0.87 | 0.31 | 0.22 | NC | NC | NC | 1 | V | ko:K06147 |
| AIL05272.1 | uppS | di-trans,poly-cis-decaprenylcistransferase | -0.65 | -0.15 | -0.06 | NC | NC | NC | 1 | I | ko:K00806 |
| AIL03976.1 | nrdI | nrdI protein | 0.78 | 0.09 | 0.09 | NC | NC | NC | 1 | F | ko:K03647 |
| AIL03650.1 | ftsX | ftsX-like permease family protein | 0.08 | -0.03 | -0.23 | NC | NC | NC | 1 | D | ko:K09811 |
| AIL03136.1 | - | Hg(II)-responsive transcriptional regulator (plasmid) | 0.86 | -0.08 | 0.27 | NC | NC | NC | 1 | K | - |
| AIL03897.1 | - | hypothetical protein DR75_1590 | -0.21 | -0.45 | -0.32 | NC | NC | NC | 1 | - | - |
| AIL04785.1 | yhaH | ytxH-like family protein | -0.24 | 0.20 | 0.31 | NC | NC | NC | 1 | D | - |
| AIL04469.1 | - | glyoxalase-like domain protein | 0.16 | 0.19 | 0.00 | NC | NC | NC | 1 | S | - |
| AIL05694.1 | yitS | EDD, DegV family domain protein | 0.87 | 0.12 | 0.22 | NC | NC | NC | 1 | S | - |
| AIL05481.1 | yitT | hypothetical protein DR75_237 | -0.13 | -0.40 | -0.03 | NC | NC | NC | 1 | S | - |
| AIL04146.1 | yebC | DNA-binding regulatory, YebC/PmpR family protein | -0.03 | 0.16 | 0.07 | NC | NC | NC | 1 | K | - |
| AIL04454.1 | glnR | merR regulatory family protein | -0.58 | -0.14 | -0.36 | NC | NC | NC | 1 | K | ko:K03713 |
| AIL04912.1 | XK27_08630 | hypothetical protein DR75_2540 | -0.07 | -0.36 | -0.62 | NC | NC | NC | 1 | T | ko:K07166 |
| AIL04342.1 | - | yycH family protein | 0.01 | -0.20 | -0.42 | NC | NC | NC | 1 | S | - |
| AIL04190.1 | rbsR | helix-turn-helix family protein | 0.01 | 0.55 | -0.02 | NC | NC | NC | 1 | K | ko:K02529 |
| AIL03102.1 | - | replication initiator A family protein (plasmid) | -0.31 | 0.18 | -0.14 | NC | NC | NC | 1 | - | - |
| AIL05736.1 | mraZ | mraZ family protein | -0.24 | -0.12 | -0.16 | NC | NC | NC | 1 | K | ko:K03925 |
| AIL05236.1 | pstS | phosphate binding family protein | 0.06 | -0.79 | -0.53 | NC | NC | NC | 1 | P | ko:K02040 |
| AIL05282.1 | - | glycosyl transferase 2 family protein | -0.27 | -0.53 | -0.27 | NC | NC | NC | 1 | S | - |
| AIL04370.1 | - | hypothetical protein DR75_1627 | 0.64 | -0.31 | -0.29 | NC | NC | NC | 1 | - | - |
| AIL04970.1 | XK27_05220 | hypothetical protein DR75_1308 | 0.70 | 0.33 | 0.43 | NC | NC | NC | 1 | S | - |
| AIL03495.1 | - | hypothetical protein DR75_1096 | -0.42 | -0.03 | 0.69 | NC | NC | NC | 1 | G | - |
| AIL05257.1 | - | putative thioesterase/dihydrolipoamide acyltransferase | -0.49 | -0.65 | -0.10 | NC | NC | NC | 1 | S | - |
| AIL05201.1 | - | helix-turn-helix family protein | -0.79 | -0.35 | -0.32 | NC | NC | NC | 1 | K | ko:K20342 |
| AIL03740.1 | copY | copper transport repressor, CopY/TcrY family | -0.16 | 0.02 | 0.21 | NC | NC | NC | 1 | K | ko:K02171 |
| AIL05380.1 | hslV | ATP-dependent protease HslVU, peptidase subunit | -0.39 | 0.06 | 0.10 | NC | NC | NC | 1 | O | ko:K01419 |
| AIL03987.1 | - | nucleotidyltransferase domain protein | 0.84 | 0.49 | 0.68 | NC | NC | NC | 1 | S | ko:K07075 |
| AIL05432.1 | - | hypothetical protein DR75_2796 | -0.17 | 0.16 | -0.42 | NC | NC | NC | 1 | - | - |
| AIL03390.1 | yclP | ABC transporter family protein | 0.42 | -0.34 | -0.51 | NC | NC | NC | 1 | P | ko:K02013 |
| AIL03559.1 | - | ABC transporter family protein | -0.20 | 0.13 | 0.12 | NC | NC | NC | 1 | V | ko:K02003 |
| AIL04013.1 | folK | 2-amino-4-hydroxy-6-hydroxymethyldihydropteridine diphosphokinase | -0.56 | -0.94 | -0.40 | NC | NC | NC | 1 | H | ko:K00950 |
| AIL05349.1 | - | hypothetical protein DR75_1512 | -0.40 | -0.92 | 0.64 | NC | NC | NC | 1 | S | - |
| AIL03793.1 | - | response regulator | -0.23 | 0.01 | -0.29 | NC | NC | NC | 1 | K | - |
| AIL03832.1 | yjbO | pseudouridine synthase, RluA family protein | -0.50 | -0.29 | -0.40 | NC | NC | NC | 1 | J | ko:K06177 |
| AIL04502.1 | XK27_10430 | NADH(P)-binding family protein | 0.34 | 0.10 | 0.22 | NC | NC | NC | 1 | S | ko:K07118 |
| AIL04623.1 | yktB | hypothetical protein DR75_1800 | -0.61 | -0.17 | -0.22 | NC | NC | NC | 1 | S | - |
| AIL04073.1 | recX | recX family protein | -0.78 | -0.26 | 0.02 | NC | NC | NC | 1 | S | ko:K03565 |
| AIL04266.1 | - | lipase family protein | -0.24 | -0.18 | -0.08 | NC | NC | NC | 1 | I | - |
| AIL05907.1 | - | hypothetical protein DR75_1058 | 0.16 | -0.41 | 0.08 | NC | NC | NC | 1 | S | - |
| AIL05787.1 | chpA | pemK-like family protein | 0.22 | -0.15 | -0.14 | NC | NC | NC | 1 | T | ko:K07171 |
| AIL04583.1 | - | putative membrane protein | 0.12 | 0.12 | 0.09 | NC | NC | NC | 1 | - | - |
| AIL03769.1 | zur | zinc-specific metallo-regulatory protein | -0.24 | 0.75 | -0.04 | NC | NC | NC | 1 | P | ko:K02076 |
| AIL04610.1 | - | signal peptidase I | 0.18 | -0.17 | 0.03 | NC | NC | NC | 1 | U | ko:K03100 |
| AIL05895.1 | - | PTS system, glucose subfamily, IIA component domain protein | -0.66 | 0.17 | -0.06 | NC | NC | NC | 1 | G | ko:K02777 |
| AIL05469.1 | - | glucosyl transferase GtrII family protein | -0.76 | -0.51 | -0.66 | NC | NC | NC | 1 | S | - |
| AIL03403.1 | potA11 | ABC transporter family protein | -0.43 | 0.75 | -0.86 | NC | NC | NC | 1 | E | ko:K02010 |
| AIL03739.1 | - | bacterial regulatory helix-turn-helix, lysR family protein | 0.34 | 0.16 | 0.23 | NC | NC | NC | 1 | K | - |
| AIL05892.1 | mdlA | ABC transporter family protein | -0.43 | -0.04 | -0.17 | NC | NC | NC | 1 | V | ko:K06148 |
| AIL03762.1 | - | hypothetical protein DR75_2390 | -0.38 | -0.04 | -0.39 | NC | NC | NC | 1 | S | - |
| AIL04480.1 | - | hypothetical protein DR75_555 | 0.12 | -0.09 | -0.24 | NC | NC | NC | 1 | E | - |
| AIL05372.1 | add | adenosine deaminase | 0.39 | 0.59 | 0.94 | NC | NC | NC | 1 | F | ko:K01488 |
| AIL05110.1 | - | bacterial regulatory s, tetR family protein | 0.16 | 0.12 | -0.64 | NC | NC | NC | 1 | K | - |
| AIL05857.1 | - | hypothetical protein DR75_2329 | 0.35 | -0.02 | -0.02 | NC | NC | NC | 1 | S | - |
| AIL04547.1 | rpmD | ribosomal protein L30 | -0.05 | 0.09 | 0.49 | NC | NC | NC | 1 | J | ko:K02907 |
| AIL05463.1 | - | thioredoxin family protein | 0.80 | -0.38 | -0.64 | NC | NC | NC | 1 | O | - |
| AIL05850.1 | copZ | heavy-metal-associated domain protein | 0.39 | 0.12 | 0.32 | NC | NC | NC | 1 | P | - |
| AIL05770.1 | rplL | ribosomal protein L7/L12 | -0.35 | -0.56 | -0.80 | NC | NC | NC | 1 | J | ko:K02935 |
| AIL04350.1 | rpsK | 30S ribosomal protein S11 | -0.29 | -0.21 | -0.37 | NC | NC | NC | 1 | J | ko:K02948 |
| AIL05549.1 | - | arginine repressor | -0.10 | 0.04 | -0.34 | NC | NC | NC | 1 | K | ko:K03402 |
| AIL03735.1 | WQ51_02665 | hypothetical protein DR75_1475 | 0.69 | -0.18 | 0.42 | NC | NC | NC | 1 | S | - |
| AIL05393.1 | rpmC | ribosomal protein L29 | -0.35 | -0.22 | -0.38 | NC | NC | NC | 1 | J | ko:K02904 |
| AIL04936.1 | ylaN | hypothetical protein DR75_1205 | 0.10 | -0.70 | -0.94 | NC | NC | NC | 1 | S | - |
| AIL04450.1 | ytxG | hypothetical protein DR75_743 | 0.15 | 0.36 | 0.38 | NC | NC | NC | 1 | S | - |
| AIL03427.1 | dmpI | 4-oxalocrotonate tautomerase enzyme family protein | -0.75 | 0.12 | 0.16 | NC | NC | NC | 1 | G | ko:K01821 |
| AIL03516.1 | ntpG | ATP synthase (F/14-kDa) subunit | 0.10 | -0.52 | -0.25 | NC | NC | NC | 1 | C | ko:K02122 |
| AIL04505.1 | - | SIS domain protein | 0.35 | 0.21 | 0.24 | NC | NC | NC | 1 | K | - |
| AIL04893.1 | - | putative lysine decarboxylase family protein | -0.21 | -0.11 | 0.24 | NC | NC | NC | 1 | S | ko:K06966 |
| AIL05867.1 | pgm6 | histidine phosphatase super family protein | -0.49 | 0.33 | -0.95 | NC | NC | NC | 1 | G | - |
| AIL05297.1 | yqgX | hypothetical protein DR75_1178 | 0.29 | 0.58 | 0.24 | NC | NC | NC | 1 | S | ko:K01069 |
| AIL05823.1 | rsgA | ribosome small subunit-dependent GTPase A | -0.45 | 0.11 | -0.34 | NC | NC | NC | 1 | S | ko:K06949 |
| AIL04310.1 | vex3 | ftsX-like permease family protein | -0.36 | -0.08 | 0.57 | NC | NC | NC | 1 | V | ko:K02004 |
| AIL03409.1 | rpsP | ribosomal protein S16 | -0.01 | 0.03 | 0.06 | NC | NC | NC | 1 | J | ko:K02959 |
| AIL04590.1 | yueF | hypothetical protein DR75_2650 | 0.43 | -0.24 | -0.17 | NC | NC | NC | 1 | S | - |
| AIL04399.1 | - | hypothetical protein DR75_1513 | 0.60 | 0.15 | -0.03 | NC | NC | NC | 1 | - | - |
| AIL05791.1 | yaaK | DNA-binding protein, YbaB/EbfC family | 0.21 | 0.19 | -0.09 | NC | NC | NC | 1 | S | ko:K09747 |
| AIL03287.1 | queT | queT transporter family protein | 0.20 | -0.37 | -0.21 | NC | NC | NC | 1 | S | - |
| AIL05631.1 | ybbF | phosphotransferase system, EIIB family protein | -0.22 | -0.10 | -0.13 | NC | NC | NC | 1 | G | ko:K02808 |
| AIL04556.1 | ylxQ | ribosomal L7Ae/L30e/S12e/Gadd45 family protein | -0.03 | -0.38 | -0.63 | NC | NC | NC | 1 | J | - |
| AIL05194.1 | xseB | exodeoxyribonuclease VII, small subunit | -0.70 | 0.16 | 0.46 | NC | NC | NC | 1 | L | ko:K03602 |
| AIL03623.1 | ispF | 2-C-methyl-D-erythritol 2,4-cyclodiphosphate synthase | 0.80 | -0.65 | -0.17 | NC | NC | NC | 1 | F | ko:K01770 |
| AIL03681.1 | accB | acetyl-CoA carboxylase, biotin carboxyl carrier protein | -0.39 | -0.01 | -0.13 | NC | NC | NC | 1 | I | ko:K02160 |
| AIL05230.1 | - | AAA domain protein | -0.32 | -0.51 | -0.37 | NC | NC | NC | 1 | E | - |
| AIL04117.1 | WQ51_04310 | asp23 family protein | 0.21 | 0.17 | 0.07 | NC | NC | NC | 1 | S | ko:K10947 |
| AIL03888.1 | rpsO | ribosomal protein S15 | -0.31 | -0.37 | -0.45 | NC | NC | NC | 1 | J | ko:K02956 |
| AIL03305.1 | - | hypothetical protein DR75_1624 | -0.14 | -0.72 | -0.40 | NC | NC | NC | 1 | - | - |
| AIL05536.1 | binR | hypothetical protein DR75_2488 | 0.09 | 0.65 | 0.42 | NC | NC | NC | 1 | L | - |
| AIL05684.1 | nrdG | anaerobic ribonucleoside-triphosphate reductase activating protein | 0.59 | -0.58 | -0.61 | NC | NC | NC | 1 | O | ko:K04068 |
| AIL04147.1 | mutY | A/G-specific adenine glycosylase | -0.09 | -0.22 | 0.29 | NC | NC | NC | 1 | L | ko:K03575 |
| AIL04945.1 | yhfA | beta-phosphoglucomutase, putative | -0.06 | 0.18 | 0.00 | NC | NC | NC | 1 | S | - |
| AIL04245.1 | - | PTS system, Lactose/Cellobiose specific IIA subunit | 0.25 | 0.13 | -0.15 | NC | NC | NC | 1 | G | ko:K02759 |
| AIL05425.1 | yebE | hypothetical protein DR75_612 | 0.41 | 0.01 | -0.23 | NC | NC | NC | 1 | S | - |
| AIL04385.1 | yaaA | S4 domain protein | 0.59 | 0.32 | 0.15 | NC | NC | NC | 1 | S | ko:K14761 |
| AIL03931.1 | - | O-Antigen ligase family protein | -0.44 | -0.33 | -0.24 | NC | NC | NC | 1 | M | - |
| AIL03113.1 | - | putative replication-associated protein (plasmid) | 0.12 | -0.29 | -0.34 | NC | NC | NC | 1 | - | - |
| AIL05286.1 | yrrM | cephalosporin hydroxylase family protein | 0.09 | 0.46 | -0.32 | NC | NC | NC | 1 | S | ko:K00588 |
| AIL04473.1 | yjcF | acetyltransferase domain protein | 0.13 | -0.02 | 0.21 | NC | NC | NC | 1 | S | - |
| AIL03294.1 | - | antibiotic biosynthesis monooxygenase family protein | -0.33 | 0.18 | -0.16 | NC | NC | NC | 1 | S | - |
| AIL05789.1 | ylbG | hypothetical protein DR75_1200 | -0.19 | 0.11 | -0.03 | NC | NC | NC | 1 | S | - |
| AIL05079.1 | tenA | thiaminase II | 0.72 | 0.49 | 0.74 | NC | NC | NC | 1 | K | ko:K03707 |
| AIL03653.1 | yidC | membrane insertase, YidC/Oxa1 family domain protein | -0.25 | 0.06 | -0.16 | NC | NC | NC | 1 | U | ko:K03217 |
| AIL03703.1 | - | putative gGDEF domain protein | -0.04 | 0.16 | 0.01 | NC | NC | NC | 1 | T | - |
| AIL03878.1 | - | putative membrane protein | 0.51 | -0.51 | -0.44 | NC | NC | NC | 1 | - | - |
| AIL05035.1 | rnpA | ribonuclease P protein component | 0.44 | -0.15 | -0.18 | NC | NC | NC | 1 | J | ko:K03536 |
| AIL04609.1 | - | fibronectin-binding family protein | -0.66 | 0.75 | -0.25 | NC | NC | NC | 1 | S | - |
| AIL04100.1 | yviA | hypothetical protein DR75_2662 | 0.53 | 0.40 | 0.01 | NC | NC | NC | 1 | S | - |
| AIL05713.1 | fabG | NAD(P)H binding domain of trans-2-enoyl-CoA reductase family protein | -0.46 | -0.88 | -0.44 | NC | NC | NC | 1 | IQ | ko:K00059 |
| AIL05535.1 | - | hypothetical protein DR75_316 | 0.48 | 0.20 | 0.13 | NC | NC | NC | 1 | S | ko:K01989 |
| AIL03902.1 | - | hypothetical protein DR75_2375 | -0.40 | 0.19 | -0.37 | NC | NC | NC | 1 | QT | ko:K09684 |
| AIL05363.1 | yufQ | branched-chain amino acid transport system / permease component family protein | 0.19 | 0.42 | -0.11 | NC | NC | NC | 1 | U | ko:K02057 |
| AIL05831.1 | - | ham1 family protein | 0.11 | -0.09 | 0.00 | NC | NC | NC | 1 | F | ko:K02428 |
| AIL03905.1 | glnQ | ABC transporter family protein | -0.54 | -0.12 | -0.25 | NC | NC | NC | 1 | E | ko:K02028 |
| AIL05143.1 | yvrA | ABC transporter family protein | -0.23 | 0.21 | 0.38 | NC | NC | NC | 1 | HP | ko:K02013 |
| AIL05375.1 | - | ftsX-like permease family protein | 0.13 | -0.15 | -0.19 | NC | NC | NC | 1 | V | ko:K02004 |
| AIL03359.1 | - | mga helix-turn-helix domain protein | 0.27 | 0.53 | -0.36 | NC | NC | NC | 1 | K | - |
| AIL05067.1 | dtd | D-tyrosyl-tRNA(Tyr) deacylase | 0.57 | 0.12 | 0.51 | NC | NC | NC | 1 | J | ko:K07560 |
| AIL05126.1 | ylbF | hypothetical protein DR75_1201 | 0.21 | -0.14 | 0.08 | NC | NC | NC | 1 | S | - |
| AIL04445.1 | - | HAD hydrolase, IA, variant 1 family protein | -0.31 | -0.59 | -0.24 | NC | NC | NC | 1 | S | - |
| AIL05223.1 | - | voltage gated chloride channel family protein | 0.50 | 0.87 | 1.00 | NC | NC | NC | 1 | P | - |
| AIL05467.1 | - | bacterial regulatory, arsR family protein | 0.33 | -0.19 | 0.06 | NC | NC | NC | 1 | K | - |
| AIL05107.1 | - | hypothetical protein DR75_1731 | -0.19 | 0.59 | -0.13 | NC | NC | NC | 1 | - | - |
| AIL05399.1 | ynzC | hypothetical protein DR75_583 | -0.21 | -0.29 | -0.41 | NC | NC | NC | 1 | S | - |
| AIL03269.1 | cdd | cytidine deaminase | 0.41 | 0.35 | 0.23 | NC | NC | NC | 1 | F | ko:K01489 |
| AIL03633.1 | - | osmC-like family protein | 0.64 | 0.91 | 0.53 | NC | NC | NC | 1 | O | - |
| AIL04982.1 | - | PTS system, Lactose/Cellobiose specific IIB subunit | 0.44 | -0.02 | 0.25 | NC | NC | NC | 1 | - | - |
| AIL04251.1 | ywkD | hypothetical protein DR75_1061 | 0.61 | 0.66 | 0.47 | NC | NC | NC | 1 | E | ko:K08234 |
| AIL05325.1 | - | phage major tail, phi13 family protein | 0.30 | -0.01 | -0.10 | NC | NC | NC | 1 | N | - |
| AIL04346.1 | - | hypothetical protein DR75_66 | 0.90 | 0.38 | 0.55 | NC | NC | NC | 1 | S | - |
| AIL05786.1 | metI | binding--dependent transport system inner membrane component family protein | 0.57 | 0.76 | 0.47 | NC | NC | NC | 1 | U | ko:K02069 |
| AIL05364.1 | aldR | reactive intermediate/imine deaminase family protein | 0.56 | -0.33 | -0.35 | NC | NC | NC | 1 | J | ko:K09022 |
| AIL04356.1 | - | NUDIX domain protein | 0.07 | 0.21 | 0.12 | NC | NC | NC | 1 | L | ko:K03574 |
| AIL03674.1 | pspC | pspC domain protein | 0.93 | 0.56 | 0.91 | NC | NC | NC | 1 | KT | ko:K03973 |
| AIL03571.1 | phaJ | maoC like domain protein | -0.36 | 0.63 | 0.53 | NC | NC | NC | 1 | I | - |
| AIL03811.1 | ytkL | metallo-beta-lactamase superfamily protein | 0.31 | 0.42 | 0.49 | NC | NC | NC | 1 | S | - |
| AIL03699.1 | - | NADPH-dependent FMN reductase family protein | 0.85 | -0.35 | -0.53 | NC | NC | NC | 1 | S | - |
| AIL03799.1 | msrA | peptide-methionine (S)-S-oxide reductase | -0.39 | 0.31 | 0.03 | NC | NC | NC | 1 | O | ko:K07304 |
| AIL05254.1 | estA | alpha/beta hydrolase fold family protein | 0.34 | -0.09 | 0.06 | NC | NC | NC | 1 | S | ko:K03930 |
| AIL05402.1 | mglC | branched-chain amino acid transport system / permease component family protein | 0.18 | 0.45 | 0.19 | NC | NC | NC | 1 | U | ko:K02057 |
| AIL03663.1 | - | putative membrane protein | -0.02 | 0.27 | -0.34 | NC | NC | NC | 1 | - | - |
| AIL04294.1 | ytoA | bacterial transferase hexapeptide family protein | 0.46 | -0.25 | -0.06 | NC | NC | NC | 1 | S | - |
| AIL04447.1 | ykuP | flavodoxin | -0.60 | -0.36 | -0.15 | NC | NC | NC | 1 | C | ko:K03839 |
| AIL04862.1 | - | hypothetical protein DR75_2666 | 0.18 | 0.42 | 0.29 | NC | NC | NC | 1 | - | - |
| AIL05054.1 | - | hypothetical protein DR75_1202 | -0.35 | -0.10 | -0.21 | NC | NC | NC | 1 | S | - |
| AIL04776.1 | ykzG | hypothetical protein DR75_1617 | -0.22 | 0.43 | 0.47 | NC | NC | NC | 1 | S | - |
| AIL05388.1 | menH | 2-succinyl-6-hydroxy-2,4-cyclohexadiene-1-carboxylate synthase | 1.00 | -0.64 | -0.62 | NC | NC | NC | 1 | I | ko:K08680 |
| AIL04384.1 | - | putative prophage Lp2 protein 7 | 0.23 | 0.15 | 0.02 | NC | NC | NC | 1 | - | - |
| AIL05359.1 | ybeY | putative rRNA maturation factor YbeY | -0.09 | 0.09 | -0.18 | NC | NC | NC | 1 | S | ko:K07042 |
| AIL04135.1 | - | hypothetical protein DR75_2133 | 0.08 | 0.37 | 0.46 | NC | NC | NC | 1 | - | - |
| AIL05784.1 | ytcD | hxlR-like helix-turn-helix family protein | -0.52 | -0.57 | -0.77 | NC | NC | NC | 1 | K | - |
| AIL03505.1 | - | hypothetical protein DR75_1808 | -0.08 | -0.52 | -0.20 | NC | NC | NC | 1 | S | - |
| AIL03418.1 | - | formate/nitrite transporter family protein | -0.10 | -0.60 | -0.51 | NC | NC | NC | 1 | P | ko:K02598 |
| AIL03565.1 | - | hypothetical protein DR75_150 | 0.31 | -0.22 | -0.17 | NC | NC | NC | 1 | - | - |
| AIL04154.1 | yutD | hypothetical protein DR75_252 | 0.32 | -0.31 | 0.31 | NC | NC | NC | 1 | S | - |
| AIL05253.1 | rodA | cell cycle family protein | 0.21 | -0.40 | -0.62 | NC | NC | NC | 1 | D | ko:K05837 |
| AIL04140.1 | rpmG | ribosomal protein L33 | -0.68 | -0.15 | -0.49 | NC | NC | NC | 1 | J | ko:K02913 |
| AIL04561.1 | lytS | 5TMR of 5TMR-LYT family protein | 0.12 | 0.41 | 0.13 | NC | NC | NC | 1 | T | ko:K07704 |
| AIL03324.1 | yojA | citrate transporter family protein | 0.28 | -0.16 | 0.21 | NC | NC | NC | 1 | EG | ko:K03299 |
| AIL05103.1 | gmk2 | guanylate kinase family protein | 0.65 | -0.77 | -0.03 | NC | NC | NC | 1 | F | ko:K00942 |
| AIL04743.1 | - | hypothetical protein DR75_1484 | 0.06 | 0.28 | 0.47 | NC | NC | NC | 1 | S | - |
| AIL05921.1 | cat | cation diffusion facilitator transporter family protein | 0.12 | -0.03 | -0.36 | NC | NC | NC | 1 | P | - |
| AIL04007.1 | - | bacterial regulatory s, tetR family protein | -0.54 | -0.97 | 0.30 | NC | NC | NC | 1 | K | - |
| AIL05391.1 | - | HAD hydrolase, IIB family protein | -0.34 | 0.86 | 0.46 | NC | NC | NC | 1 | S | - |
| AIL04097.1 | XK27_01785 | hypothetical protein DR75_927 | 0.40 | 0.31 | -0.47 | NC | NC | NC | 1 | S | - |
| AIL03766.1 | ydjH | hypothetical protein DR75_1227 | 0.70 | 0.35 | 0.33 | NC | NC | NC | 1 | S | ko:K06872 |
| AIL04160.1 | pyrP | uracil-xanthine permease family protein | -0.46 | -0.69 | -0.39 | NC | NC | NC | 1 | F | ko:K02824 |
| AIL04216.1 | pncA | isochorismatase family protein | 0.92 | 0.31 | 0.32 | NC | NC | NC | 1 | Q | ko:K16788 |
| AIL04333.1 | - | bacteriophage HK97-gp10, tail-component family protein | 0.27 | -0.53 | -0.25 | NC | NC | NC | 1 | S | - |
| AIL03292.1 | - | hypothetical protein DR75_2452 | -0.38 | 0.05 | -0.34 | NC | NC | NC | 1 | S | - |
| AIL05011.1 | - | mepB family protein | 0.10 | -0.33 | -0.50 | NC | NC | NC | 1 | S | - |
| AIL04604.1 | - | Cof-like hydrolase family protein | -0.52 | -0.06 | -0.39 | NC | NC | NC | 1 | S | - |
| AIL05560.1 | - | bacterial regulatory s, tetR family protein | -0.26 | -0.80 | 0.11 | NC | NC | NC | 1 | K | - |
| AIL04418.1 | oppC | binding--dependent transport system inner membrane component family protein | -0.44 | -0.69 | -0.62 | NC | NC | NC | 1 | EP | ko:K02034 |
| AIL03521.1 | yjhE | hypothetical protein DR75_415 | 0.94 | 0.26 | -0.08 | NC | NC | NC | 1 | S | - |
| AIL05059.1 | comEB | ComE operon protein 2 | 0.47 | 0.72 | -0.30 | NC | NC | NC | 1 | F | ko:K01493 |
| AIL04354.1 | rpsN | 30S ribosomal protein S14 type Z | -0.48 | -0.39 | -0.18 | NC | NC | NC | 1 | J | ko:K02954 |
| AIL03440.1 | vex2 | ABC transporter family protein | 0.09 | 0.19 | -0.07 | NC | NC | NC | 1 | V | ko:K02003 |
| AIL05834.1 | citG | triphosphoribosyl-dephospho-CoA synthase CitG | 0.50 | 0.18 | 0.56 | NC | NC | NC | 1 | H | ko:K05966 |
| AIL04139.1 | ruvA | Holliday junction DNA helicase RuvA | -0.02 | -0.12 | -0.17 | NC | NC | NC | 1 | L | ko:K03550 |
| AIL04962.1 | pgsA | CDP-diacylglycerol--glycerol-3-phosphate 3-phosphatidyltransferase | 0.58 | 0.12 | -0.01 | NC | NC | NC | 1 | I | ko:K00995 |
| AIL04369.1 | XK27_04830 | hypothetical protein DR75_2750 | 0.58 | 0.55 | 0.25 | NC | NC | NC | 1 | S | - |
| AIL05925.1 | - | hypothetical protein DR75_1438 | -0.32 | -0.54 | 0.10 | NC | NC | NC | 1 | S | - |
| AIL05193.1 | - | phosphoribosyl-ATP pyrophosphohydrolase family protein | 0.12 | 0.02 | -0.22 | NC | NC | NC | 1 | S | ko:K16785 |
| AIL05863.1 | menC | o-succinylbenzoate synthase | -0.13 | 0.07 | 0.54 | NC | NC | NC | 1 | H | ko:K02549 |
| AIL03918.1 | - | acetyltransferase family protein | 0.50 | 0.84 | 0.81 | NC | NC | NC | 1 | S | ko:K03824 |
| AIL04357.1 | sipC | signal peptidase I | 0.37 | -0.38 | -0.08 | NC | NC | NC | 1 | U | ko:K03100 |
| AIL05617.1 | yidA | SIS domain protein | 0.45 | 0.02 | 0.16 | NC | NC | NC | 1 | K | - |
| AIL03421.1 | - | binding--dependent transport system inner membrane component family protein | -0.23 | 0.54 | 0.21 | NC | NC | NC | 1 | U | ko:K02025 |
| AIL04029.1 | oppB | binding--dependent transport system inner membrane component family protein | 0.74 | 0.11 | -0.15 | NC | NC | NC | 1 | EP | ko:K02033 |
| AIL05268.1 | yybA | marR family protein | 0.48 | 0.30 | 0.04 | NC | NC | NC | 1 | K | - |
| AIL04701.1 | cydA | bacterial Cytochrome Ubiquinol Oxidase family protein | -0.82 | -0.01 | -0.26 | NC | NC | NC | 1 | C | ko:K00425 |
| AIL05822.1 | - | cytidine and deoxycytidylate deaminase zinc-binding region family protein | 0.20 | 0.64 | 0.21 | NC | NC | NC | 1 | F | - |
| AIL03645.1 | - | acetyltransferase family protein | -0.60 | 0.05 | -0.02 | NC | NC | NC | 1 | K | - |
| AIL05354.1 | cps2D | 3-beta hydroxysteroid dehydrogenase/isomerase family protein | -0.85 | -0.31 | -0.03 | NC | NC | NC | 1 | GM | ko:K01784 |
| AIL04066.1 | - | putative membrane protein | -0.31 | 0.46 | -0.29 | NC | NC | NC | 1 | - | - |
| AIL03118.1 | - | bacterial regulatory, arsR family protein (plasmid) | 0.17 | -0.26 | -0.69 | NC | NC | NC | 1 | K | ko:K03892 |
| AIL03311.1 | napA | transporter, monovalent cation:proton antiporter-2 family protein | 0.28 | 0.22 | 0.07 | NC | NC | NC | 1 | P | - |
| AIL03917.1 | ebsA | putative ebsA protein | 0.77 | 0.29 | 0.05 | NC | NC | NC | 1 | S | - |
| AIL04707.1 | yuaF | putative yuaF like protein | 0.26 | 0.71 | 0.59 | NC | NC | NC | 1 | OU | - |
| AIL03709.1 | - | alpha/beta hydrolase fold family protein | -0.61 | -0.34 | -0.52 | NC | NC | NC | 1 | I | - |
| AIL04959.1 | - | sigma-54 interaction domain protein | 0.13 | 0.08 | -0.20 | NC | NC | NC | 1 | K | - |
| AIL03805.1 | - | hypothetical protein DR75_613 | -0.03 | -0.25 | -0.18 | NC | NC | NC | 1 | S | - |
| AIL03566.1 | fnq20 | FAD-NAD(P)-binding family protein | 0.12 | 0.17 | 0.92 | NC | NC | NC | 1 | S | - |
| AIL03779.1 | rpoE | putative DNA-directed RNA polymerase subunit delta | -0.27 | -0.83 | -0.55 | NC | NC | NC | 1 | K | ko:K03048 |
| AIL03254.1 | yabO | S4 domain protein | 0.72 | -0.18 | -0.25 | NC | NC | NC | 1 | J | - |
| AIL05173.1 | gpsB | DivIVA domain protein | 0.08 | 0.02 | 0.01 | NC | NC | NC | 1 | D | - |
| AIL03195.1 | - | hypothetical protein DR75_2891 (plasmid) | 0.00 | 0.19 | 0.27 | NC | NC | NC | 1 | - | - |
| AIL04965.1 | yajC | preprotein translocase, YajC subunit | -0.06 | -0.61 | -0.29 | NC | NC | NC | 1 | U | ko:K03210 |
| AIL05718.1 | infA | translation initiation factor IF-1 | -0.28 | 0.35 | 0.27 | NC | NC | NC | 1 | J | ko:K02518 |
| AIL05133.1 | - | hypothetical protein DR75_1666 | 0.89 | -0.73 | -0.52 | NC | NC | NC | 1 | - | - |
| AIL05387.1 | rpsT | ribosomal protein S20 | -0.17 | -0.64 | 0.22 | NC | NC | NC | 1 | J | ko:K02968 |
| AIL03730.1 | ywnA | putative HTH-type transcriptional regulator ywnA | 0.87 | -0.53 | -1.00 | NC | NC | NC | 1 | K | - |
| AIL05861.1 | yugI | S1 RNA binding domain protein | -0.15 | -0.15 | -0.56 | NC | NC | NC | 1 | J | ko:K07570 |
| AIL04553.1 | ptsH | phosphocarrier protein HPr | -0.57 | 0.78 | 0.35 | NC | NC | NC | 1 | G | ko:K11189 |
| AIL04996.1 | yhbY | CRS1 / YhbY domain protein | -0.01 | -0.59 | -0.60 | NC | NC | NC | 1 | J | ko:K07574 |
| AIL03655.1 | - | hypothetical protein DR75_1664 | 0.21 | -0.64 | -0.15 | NC | NC | NC | 1 | - | - |
| AIL03715.1 | glxI | glyoxalase-like domain protein | 0.59 | 0.00 | 0.15 | NC | NC | NC | 1 | E | ko:K01759 |
| AIL03538.1 | secG | preprotein translocase, SecG subunit | -0.22 | -0.41 | -0.95 | NC | NC | NC | 1 | U | ko:K03075 |
| AIL04515.1 | sdaAB | L-serine dehydratase, iron-sulfur-dependent, beta subunit | -0.80 | -0.91 | -0.06 | NC | NC | NC | 1 | E | ko:K01752 |
| AIL05335.1 | - | hypothetical protein DR75_379 | -0.24 | -0.08 | -0.25 | NC | NC | NC | 1 | - | - |
| AIL05312.1 | - | hypothetical protein DR75_2764 | 0.75 | 0.26 | 0.15 | NC | NC | NC | 1 | K | - |
| AIL04662.1 | ctpA | hypothetical protein DR75_680 | 0.63 | 0.22 | 0.85 | NC | NC | NC | 1 | M | ko:K03797 |
| AIL03111.1 | lgt | prolipoprotein diacylglyceryl transferase (plasmid) | -0.36 | -0.41 | 0.22 | NC | NC | NC | 1 | M | ko:K13292 |
| AIL03573.1 | - | phage major tail protein, TP901-1 family | 0.63 | -0.22 | 0.67 | NC | NC | NC | 1 | S | - |
| AIL05037.1 | ecfT | cobalt transport family protein | -0.41 | -0.60 | -0.64 | NC | NC | NC | 1 | U | ko:K16785 |
| AIL03267.1 | yrvD | hypothetical protein DR75_690 | -0.18 | -0.29 | -0.21 | NC | NC | NC | 1 | S | - |
| AIL04431.1 | - | putative membrane protein | 0.71 | 0.50 | -0.51 | NC | NC | NC | 1 | - | - |
| AIL03498.1 | acmA | mannosyl-glycoendo-beta-N-acetylglucosaminidase family protein | -0.85 | -0.35 | 0.04 | NC | NC | NC | 1 | NU | ko:K01185 |
| AIL04332.1 | yitW | hypothetical protein DR75_513 | -0.83 | -0.58 | -0.52 | NC | NC | NC | 1 | S | - |
| AIL05593.1 | pdtaR | ANTAR domain protein | -0.37 | -0.54 | -0.28 | NC | NC | NC | 1 | K | ko:K22010 |
| AIL03812.1 | recU | recombination protein U | -0.60 | -0.23 | 0.13 | NC | NC | NC | 1 | L | ko:K03700 |
| AIL04756.1 | mta | merR regulatory family protein | -0.45 | -0.58 | 0.05 | NC | NC | NC | 1 | K | - |
| AIL05058.1 | ytsP | putative gAF domain protein | 0.40 | 0.48 | 0.62 | NC | NC | NC | 1 | T | ko:K08968 |
| AIL04533.1 | - | hypothetical protein DR75_899 | 0.06 | 0.07 | 0.16 | NC | NC | NC | 1 | - | - |
| AIL04041.1 | mgtE | magnesium transporter | 0.15 | -0.43 | -0.49 | NC | NC | NC | 1 | P | ko:K06213 |
| AIL03666.1 | oppB | binding--dependent transport system inner membrane component family protein | -0.94 | -0.48 | -0.72 | NC | NC | NC | 1 | EP | ko:K15581 |
| AIL03994.1 | - | hypothetical protein DR75_905 | 0.04 | 0.68 | 0.42 | NC | NC | NC | 1 | S | - |
| AIL05345.1 | - | hypothetical protein DR75_491 | -0.34 | -0.30 | -0.40 | NC | NC | NC | 1 | - | - |
| AIL04203.1 | XK27_08840 | branched-chain amino acid transport system / permease component family protein | 0.38 | 0.48 | -0.09 | NC | NC | NC | 1 | U | ko:K05832 |
| AIL04330.1 | - | helix-turn-helix family protein | 0.13 | -0.51 | -0.44 | NC | NC | NC | 1 | K | ko:K01356 |
| AIL05760.1 | - | hypothetical protein DR75_1847 | -0.58 | -0.41 | -0.70 | NC | NC | NC | 1 | S | - |
| AIL04494.1 | atpE | ATP synthase F0, C subunit | -0.38 | -0.05 | -0.08 | NC | NC | NC | 1 | C | ko:K02110 |
| AIL03235.1 | rdrB | hypothetical protein DR75_2604 | 0.15 | 0.08 | -0.78 | NC | NC | NC | 1 | K | ko:K02444 |
| AIL05630.1 | yozE | hypothetical protein DR75_681 | 0.28 | 0.91 | -0.71 | NC | NC | NC | 1 | S | - |
| AIL05505.1 | ypjD | hypothetical protein DR75_559 | -0.03 | -0.52 | -0.45 | NC | NC | NC | 1 | S | - |
| AIL04239.1 | - | hypothetical protein DR75_2373 | -0.23 | -0.24 | -0.30 | NC | NC | NC | 1 | - | - |
| AIL03950.1 | pvaA | lysozyme-like family protein | 0.06 | -0.87 | -0.90 | NC | NC | NC | 1 | M | - |
| AIL04782.1 | - | hypothetical protein DR75_6 | -0.30 | 0.28 | -0.22 | NC | NC | NC | 1 | - | - |
| AIL05761.1 | - | hypothetical protein DR75_882 | -0.45 | 0.34 | 0.72 | NC | NC | NC | 1 | - | - |
| AIL03180.1 | - | putative uvrC (plasmid) | -0.01 | -0.45 | -0.03 | NC | NC | NC | 1 | - | - |
| AIL04768.1 | - | hypothetical protein DR75_1652 | 0.23 | -0.08 | -0.41 | NC | NC | NC | 1 | S | - |
| AIL04089.1 | lrp | bacterial regulatory, Fis family protein | -0.04 | 0.55 | 0.28 | NC | NC | NC | 1 | QT | - |
| AIL04148.1 | - | response regulator | 0.92 | -0.99 | -0.82 | NC | NC | NC | 1 | K | ko:K18349 |
| AIL05837.1 | - | hypothetical protein DR75_537 | 0.82 | -0.06 | -0.32 | NC | NC | NC | 1 | S | - |
| AIL03701.1 | - | putative membrane protein | 0.38 | 0.01 | 0.25 | NC | NC | NC | 1 | - | - |
| AIL03463.1 | - | hypothetical protein DR75_382 | -0.25 | -0.44 | 0.80 | NC | NC | NC | 1 | - | - |
| AIL05076.1 | ybbM | hypothetical protein DR75_1450 | -0.76 | 0.02 | -0.14 | NC | NC | NC | 1 | S | ko:K02069 |
| AIL03271.1 | ribU | hypothetical protein DR75_545 | -0.97 | -0.60 | -0.91 | NC | NC | NC | 1 | U | - |
| AIL04864.1 | rpl | SIS domain protein | -0.05 | 0.03 | 0.37 | NC | NC | NC | 1 | K | - |
| AIL03508.1 | - | lysM domain protein | -0.63 | -0.16 | -0.28 | NC | NC | NC | 1 | M | - |
| AIL03369.1 | - | cation diffusion facilitator transporter family protein | -0.83 | -0.76 | -0.39 | NC | NC | NC | 1 | U | - |
| AIL05447.1 | - | hypothetical protein DR75_2591 | 0.94 | 0.94 | 0.17 | NC | NC | NC | 1 | S | - |
| AIL03746.1 | - | hypothetical protein DR75_1892 | 0.35 | -0.08 | -0.16 | NC | NC | NC | 1 | S | - |
| AIL04822.1 | - | hypothetical protein DR75_301 | -0.88 | 0.08 | -0.51 | NC | NC | NC | 1 | L | - |
| AIL03293.1 | - | general stress protein A | -0.10 | -0.64 | -0.04 | NC | NC | NC | 1 | M | - |
| AIL03690.1 | kdgK | pfkB carbohydrate kinase family protein | 0.34 | -0.34 | 0.89 | NC | NC | NC | 1 | G | ko:K00874 |
| AIL04719.1 | - | PTS system mannose/fructose/sorbose IID component family protein | 0.05 | -0.04 | 0.94 | NC | NC | NC | 1 | G | ko:K19509 |
| AIL05389.1 | - | xylose isomerase-like TIM barrel family protein | 0.48 | -0.40 | -0.03 | NC | NC | NC | 1 | G | - |
| AIL04102.1 | rpmJ | ribosomal protein L36 | 0.08 | 0.36 | 0.04 | NC | NC | NC | 1 | J | ko:K02919 |
| AIL05513.1 | rpmG | ribosomal protein L33 | -0.17 | 0.29 | 0.10 | NC | NC | NC | 1 | J | ko:K02913 |
| AIL05355.1 | rpe | ribulose-phosphate 3-epimerase | -0.28 | -0.20 | -0.20 | NC | NC | NC | 1 | G | ko:K01783 |
| AIL03315.1 | lspA | signal peptidase II | 0.20 | -0.13 | 0.08 | NC | NC | NC | 1 | MU | ko:K03101 |
| AIL05138.1 | glpF | MIP channel s family protein | 0.33 | 0.84 | 0.33 | NC | NC | NC | 1 | U | ko:K02440 |
| AIL05242.1 | - | iron-sulfur cluster biosynthesis family protein | 0.57 | 0.19 | 0.01 | NC | NC | NC | 1 | S | - |
| AIL03431.1 | - | hypothetical protein DR75_163 | 0.00 | -0.30 | -0.19 | NC | NC | NC | 1 | S | - |
| AIL03625.1 | oadG | hlyD secretion family protein | 0.90 | 0.17 | 0.83 | NC | NC | NC | 1 | I | - |
| AIL03380.1 | ccl | queT transporter family protein | -0.20 | -0.93 | -0.86 | NC | NC | NC | 1 | S | - |
| AIL03101.1 | - | hypothetical protein DR75_2991 (plasmid) | 0.86 | 0.16 | -0.16 | NC | NC | NC | 1 | - | - |
| AIL04313.1 | acpP | phosphopantetheine attachment site family protein | -0.85 | -0.14 | -0.81 | NC | NC | NC | 1 | IQ | ko:K02078 |
| AIL04696.1 | ykcA | glyoxalase/Bleomycin resistance /Dioxygenase superfamily protein | -0.07 | 0.52 | 0.28 | NC | NC | NC | 1 | E | - |
| AIL04374.1 | rpmG | ribosomal protein L33 | -0.33 | -0.06 | 0.16 | NC | NC | NC | 1 | J | ko:K02913 |
| AIL04051.1 | yebE | hypothetical protein DR75_1557 | -0.17 | 0.00 | -0.47 | NC | NC | NC | 1 | S | - |
| AIL04726.1 | - | putative membrane protein | 0.26 | -0.75 | 0.64 | NC | NC | NC | 1 | - | - |
| AIL05365.1 | - | hypothetical protein DR75_391 | 0.34 | 0.23 | 0.08 | NC | NC | NC | 1 | - | - |
| AIL03104.1 | - | hypothetical protein DR75_2992 (plasmid) | 0.66 | 0.24 | 0.21 | NC | NC | NC | 1 | - | - |
| AIL04484.1 | - | pTS family porter | -0.77 | -0.20 | 0.16 | NC | NC | NC | 1 | - | - |
| AIL05420.1 | - | hypothetical protein DR75_991 | -0.29 | -0.29 | -0.11 | NC | NC | NC | 1 | S | ko:K16924 |
| AIL04923.1 | lipL | biotin/lipoate A/B ligase family protein | 0.20 | 0.33 | 0.32 | NC | NC | NC | 1 | H | ko:K16869 |
| AIL05101.1 | - | hypothetical protein DR75_73 | 0.88 | 0.80 | 0.72 | NC | NC | NC | 1 | - | - |
| AIL03126.1 | - | putative replication control protein PrgN (plasmid) | 0.22 | 0.20 | 0.18 | NC | NC | NC | 1 | - | - |
| AIL03989.1 | mntH | metal ion transporter, metal ion family protein | 0.33 | -0.20 | 0.39 | NC | NC | NC | 1 | U | ko:K03322 |
| AIL04790.1 | rpmG | ribosomal protein L33 | -0.57 | -0.85 | -0.06 | NC | NC | NC | 1 | J | ko:K02913 |
| AIL04005.1 | - | hypothetical protein DR75_1526 | 0.68 | -0.49 | -0.07 | NC | NC | NC | 1 | S | - |
| AIL04506.1 | ywrF | flavin reductase like domain protein | 0.36 | 0.02 | 0.03 | NC | NC | NC | 1 | S | - |
| AIL04920.1 | - | PTS system, mannose/fructose/sorbose, IIB component family protein | 0.36 | -0.31 | -0.11 | NC | NC | NC | 1 | G | ko:K02745 |
| AIL03291.1 | - | PTS system fructose IIA component family protein | -0.47 | -0.14 | -0.43 | NC | NC | NC | 1 | G | ko:K02793 |
| AIL05777.1 | ntpK | V-type sodium ATPase subunit K | -0.16 | 0.23 | 0.00 | NC | NC | NC | 1 | U | ko:K02124 |
| AIL04267.1 | - | hypothetical protein DR75_47 | 0.62 | 0.65 | 0.25 | NC | NC | NC | 1 | S | - |
| AIL05650.1 | - | helix-turn-helix family protein | 0.62 | -0.42 | 0.36 | NC | NC | NC | 1 | K | - |
| AIL04360.1 | sacT | CAT RNA binding domain protein | -0.68 | 0.04 | 0.10 | NC | NC | NC | 1 | K | ko:K03488 |
| AIL03125.1 | - | ASCH domain protein (plasmid) | 0.79 | 0.79 | -0.50 | NC | NC | NC | 1 | S | - |
| AIL04908.1 | aroD | 3-dehydroquinate dehydratase | 0.25 | 0.45 | 0.06 | NC | NC | NC | 1 | E | ko:K03785 |
| AIL03107.1 | arsR | bacterial regulatory, arsR family protein (plasmid) | -0.01 | -0.19 | -0.11 | NC | NC | NC | 1 | K | ko:K03892 |
| AIL04206.1 | rpsU | ribosomal protein S21 | 0.31 | 0.19 | -0.19 | NC | NC | NC | 1 | J | ko:K02970 |
| AIL04617.1 | atpB | ATP synthase F0, A subunit | -0.55 | -0.28 | -0.48 | NC | NC | NC | 1 | C | ko:K02108 |
| AIL05412.1 | cdsA | cytidylyltransferase family protein | -0.31 | -0.22 | 0.48 | NC | NC | NC | 1 | S | ko:K00981 |
| AIL03529.1 | cysE | serine O-acetyltransferase | -0.38 | -0.63 | 0.09 | NC | NC | NC | 1 | E | ko:K00640 |
| AIL03436.1 | ulaA | PTS system sugar-specific permease component family protein | 0.47 | -0.48 | -0.72 | NC | NC | NC | 1 | S | ko:K03475 |
| AIL05801.1 | rimI | acetyltransferase family protein | -0.97 | -0.02 | -0.06 | NC | NC | NC | 1 | K | ko:K03789 |
| AIL03459.1 | oadB | sodium ion-translocating decarboxylase, beta subunit | 0.21 | 0.55 | 0.69 | NC | NC | NC | 1 | C | ko:K01572 |
| AIL04944.1 | - | helix-turn-helix domain protein | 0.43 | -0.24 | -0.16 | NC | NC | NC | 1 | K | - |
| AIL04740.1 | rhaD | rhamnulose-1-phosphate aldolase | 0.23 | 0.57 | 0.33 | NC | NC | NC | 1 | G | ko:K01629 |
| AIL04802.1 | gdh | glucose 1-dehydrogenase | 0.27 | -0.36 | 0.06 | NC | NC | NC | 1 | IQ | ko:K00034 |
| AIL04667.1 | - | mazG nucleotide pyrophosphohydrolase domain protein | -0.22 | -0.29 | -0.53 | NC | NC | NC | 1 | S | - |
| AIL03864.1 | ykuP | flavodoxin | -0.39 | 0.71 | 0.33 | NC | NC | NC | 1 | C | ko:K03839 |
| AIL03148.1 | - | hypothetical protein DR75_2897 (plasmid) | -0.38 | -0.92 | -0.59 | NC | NC | NC | 1 | L | - |
| AIL05062.1 | - | bacteriocin-protection, YdeI/OmpD-Associated family protein | -0.94 | -0.34 | 0.14 | NC | NC | NC | 1 | S | - |
| AIL03553.1 | - | hypothetical protein DR75_985 | -0.24 | -0.76 | -0.35 | NC | NC | NC | 1 | S | - |
| AIL03599.1 | - | LPXTG cell wall anchor domain protein | 0.34 | 0.71 | -0.20 | NC | NC | NC | 1 | S | - |
| AIL05532.1 | - | wxL domain surface cell wall-binding family protein | -0.67 | -0.58 | -0.54 | NC | NC | NC | 1 | S | - |
| AIL05840.1 | - | uvrB/uvrC motif family protein | 0.36 | 0.05 | 0.10 | NC | NC | NC | 1 | L | ko:K03703 |
| AIL05051.1 | recO | DNA repair protein RecO | 0.87 | 0.81 | 0.26 | NC | NC | NC | 1 | L | ko:K03584 |
| AIL04305.1 | - | hypothetical protein DR75_480 | 0.13 | 0.78 | 0.46 | NC | NC | NC | 1 | - | - |
| AIL04496.1 | ytqA | radical SAM superfamily protein | -0.51 | -0.45 | -0.48 | NC | NC | NC | 1 | S | ko:K07139 |
| AIL03783.1 | - | YGGT family protein | -0.33 | -0.26 | 0.00 | NC | NC | NC | 1 | S | ko:K02221 |

**Table. S6**. Proteins consistently upregulated throughout the time course of copper NP treatment in *E. faecalis* ATCC 29212.

| **Protein ID** | **Gene name** | **Protein description** | **Fold Ratio** | | | **Expression Pattern (EP)** | | | **EP group** | **COG** | **KEGG number** |
| --- | --- | --- | --- | --- | --- | --- | --- | --- | --- | --- | --- |
|  |  |  | **10 min** | **30 min** | **60 min** | **10 min** | **30 min** | **60 min** |  |  |  |
| AIL03545.1 | - | hypothetical protein DR75_2114 | 1.85 | 1.88 | 1.87 | U | U | U | 2 | M | ko:K03931 |
| AIL04916.1 | ypcG | hypothetical protein DR75_1068 | 1.34 | 2.14 | 1.46 | U | U | U | 2 | G | ko:K17318 |
| AIL03250.1 | malE | bacterial extracellular solute-binding family protein | 1.79 | 2.13 | 1.81 | U | U | U | 2 | G | ko:K15770 |
| AIL04449.1 | priA | primosomal protein N' | 1.24 | 1.17 | 1.11 | U | U | U | 2 | L | ko:K04066 |
| AIL04453.1 | mapA | hypothetical protein DR75_10 | 1.66 | 1.99 | 1.60 | U | U | U | 2 | G | ko:K00691 |
| AIL04487.1 | nplT | hypothetical protein DR75_422 | 1.20 | 1.73 | 1.40 | U | U | U | 2 | G | ko:K01208 |
| AIL05719.1 | galE | UDP-glucose 4-epimerase GalE | 1.75 | 1.56 | 1.36 | U | U | U | 2 | M | ko:K01784 |
| AIL03601.1 | - | dihydroorotate dehydrogenase family protein | 1.79 | 1.90 | 1.47 | U | U | U | 2 | G | ko:K01625 |
| AIL04409.1 | yclK | HAMP domain protein | 3.63 | 3.55 | 3.39 | U | U | U | 2 | T | - |
| AIL05596.1 | sodA | superoxide dismutase Mn | 1.60 | 1.13 | 1.25 | U | U | U | 2 | C | ko:K04564 |
| AIL03816.1 | yvyE | hypothetical protein DR75_767 | 3.72 | 3.56 | 3.30 | U | U | U | 2 | S | ko:K01271 |
| AIL04915.1 | - | hypothetical protein DR75_2586 | 1.82 | 1.42 | 1.12 | U | U | U | 2 | - | - |
| AIL05043.1 | arbF3 | PTS system, beta-glucoside-specific IIABC component family protein | 2.30 | 2.18 | 1.87 | U | U | U | 2 | G | ko:K02755 |
| AIL04157.1 | ptsG | PTS system, glucose subfamily, IIA component domain protein | 1.55 | 2.11 | 1.83 | U | U | U | 2 | G | ko:K02777 |
| AIL05083.1 | hipO | amidohydrolase family protein | 3.14 | 3.08 | 3.09 | U | U | U | 2 | E | ko:K05823 |
| AIL04292.1 | sgcB | PTS system, Lactose/Cellobiose specific IIB subunit | 1.11 | 2.14 | 1.14 | U | U | U | 2 | G | ko:K02774 |
| AIL04303.1 | - | hypothetical protein DR75_1667 | 4.16 | 1.41 | 1.76 | U | U | U | 2 | - | - |
| AIL05384.1 | dinB | hypothetical protein DR75_1449 | 2.84 | 1.90 | 1.04 | U | U | U | 2 | L | ko:K02346 |
| AIL05561.1 | pucD | [2Fe-2S] binding domain protein | 1.30 | 1.66 | 1.11 | U | U | U | 2 | C | ko:K00087 |
| AIL04620.1 | lacC | tagatose-6-phosphate kinase | 3.18 | 1.25 | 1.41 | U | U | U | 2 | F | ko:K00917 |
| AIL03542.1 | - | PGAP1-like family protein | 2.01 | 1.02 | 1.26 | U | U | U | 2 | S | - |
| AIL04578.1 | opuCD | binding--dependent transport system inner membrane component family protein | 2.88 | 2.45 | 2.02 | U | U | U | 2 | E | ko:K05846 |
| AIL05030.1 | - | beta-lactamase family protein | 2.43 | 2.11 | 2.04 | U | U | U | 2 | V | - |
| AIL05679.1 | - | hypothetical protein DR75_1257 | 2.61 | 2.80 | 2.36 | U | U | U | 2 | - | - |
| AIL05081.1 | - | wxL domain surface cell wall-binding family protein | 2.39 | 2.05 | 1.14 | U | U | U | 2 | S | - |
| AIL03178.1 | - | ABC transporter family protein (plasmid) | 2.47 | 1.46 | 1.80 | U | U | U | 2 | V | ko:K06148 |
| AIL03997.1 | - | hypothetical protein DR75_156 | 1.99 | 3.31 | 3.47 | U | U | U | 2 | - | - |

**Table. S7**. Proteins consistently downregulated throughout the time course of copper NP treatment in *E. faecalis* ATCC 29212.

| **Protein ID** | **Gene name** | **Protein description** | **Fold Ratio** | | | **Expression Pattern (EP)** | | | **EP group** | **COG** | **KEGG number** |
| --- | --- | --- | --- | --- | --- | --- | --- | --- | --- | --- | --- |
|  |  |  | **10 min** | **30 min** | **60 min** | **10 min** | **30 min** | **60 min** |  |  |  |
| AIL03183.1 | - | subtilase family protein (plasmid) | -1.00 | -1.21 | -1.31 | D | D | D | 3 | O | ko:K20486 |
| AIL05732.1 | tyrA | ACT domain protein | -1.49 | -1.01 | -1.02 | D | D | D | 3 | E | ko:K04517 |
| AIL03927.1 | cad | FMN-binding domain protein | -2.45 | -2.25 | -1.52 | D | D | D | 3 | S | ko:K20379 |
| AIL04870.1 | pflA | pyruvate formate-lyase 1-activating enzyme | -1.47 | -1.88 | -1.12 | D | D | D | 3 | C | ko:K04069 |
| AIL05009.1 | miaA | tRNA dimethylallyltransferase | -1.94 | -1.27 | -1.39 | D | D | D | 3 | F | ko:K00791 |
| AIL04948.1 | lplA | lipoyltransferase and lipoate-ligase family protein | -1.66 | -1.43 | -1.88 | D | D | D | 3 | H | ko:K03800 |
| AIL05693.1 | - | hypothetical protein DR75_1813 | -1.46 | -1.26 | -1.32 | D | D | D | 3 | S | ko:K09705 |
| AIL03408.1 | - | helix-turn-helix family protein | -2.55 | -3.60 | -1.91 | D | D | D | 3 | K | - |
| AIL03824.1 | - | hypothetical protein DR75_2783 | -1.38 | -1.07 | -1.26 | D | D | D | 3 | S | ko:K09768 |
| AIL04103.1 | ndoA | mRNA interferase EndoA | -1.49 | -1.56 | -1.10 | D | D | D | 3 | L | ko:K07171 |
| AIL04087.1 | hgdC | putative CoA-substrate-specific enzyme activase domain protein | -1.72 | -3.01 | -2.80 | D | D | D | 3 | I | - |
| AIL05315.1 | apc3 | hydantoinase/oxoprolinase family protein | -1.15 | -2.23 | -2.49 | D | D | D | 3 | EQ | - |
| AIL03936.1 | - | carbamate kinase | -3.60 | -3.77 | -2.02 | D | D | D | 3 | E | ko:K00926 |
| AIL04479.1 | psiE | phosphate-starvation-inducible E family protein | -3.85 | -1.77 | -2.85 | D | D | D | 3 | S | ko:K13256 |
| AIL03727.1 | copY | copper transport repressor, CopY/TcrY family | -2.60 | -1.69 | -13.29 | D | D | D | 3 | K | ko:K02171 |

**Table. S8**. Proteins identified from *P. mirabilis* ATCC 7002 following copper NP treatment.

| **Protein ID** | **Gene name** | **Protein description** | **Fold Ratio** | | **Expression Pattern (EP)** | | **EP group** | **COG ID** | **KEGG number** |
| --- | --- | --- | --- | --- | --- | --- | --- | --- | --- |
| **10 min** | **30 min** | **10 min** | **30 min** |
| KGA90317.1 | clpB | ATP-dependent chaperone protein ClpB | -0.25 | -0.38 | NC | NC | 1 | O | ko:K03695 |
| KGA91925.1 | rpoC | DNA-directed RNA polymerase, beta' subunit | 1.34 | -0.05 | U | NC | 5 | K | ko:K03046 |
| KGA91942.1 | rpoB | DNA-directed RNA polymerase, beta subunit | 0.90 | -0.64 | NC | NC | 1 | K | ko:K03043 |
| KGA90602.1 | yfhM | MG2 domain protein | 0.04 | 0.54 | NC | NC | 1 | S | ko:K06894 |
| KGA91321.1 | aceE | pyruvate dehydrogenase (acetyl-transferring), homodimeric type | 0.22 | -0.57 | NC | NC | 1 | C | ko:K00163 |
| KGA91736.1 | ileS | isoleucine--tRNA ligase | -0.45 | -1.06 | NC | D | 6 | J | ko:K01870 |
| KGA91537.1 | dnaK | chaperone protein DnaK | -0.05 | 0.17 | NC | NC | 1 | O | ko:K04043 |
| KGA90885.1 | purL | phosphoribosylformylglycinamidine synthase | 1.41 | -0.46 | U | NC | 5 | F | ko:K01952 |
| KGA91131.1 | oppA | periplasmic oligopeptide-binding protein | 0.03 | 0.15 | NC | NC | 1 | E | ko:K15580 |
| KGA91422.1 | acnB | aconitate hydratase 2 | -0.10 | -0.14 | NC | NC | 1 | C | ko:K01682 |
| KGA90082.1 | pflB | formate acetyltransferase | 1.79 | -0.12 | U | NC | 5 | C | ko:K00656 |
| KGA90141.1 | - | oxoglutarate dehydrogenase (succinyl-transferring), E1 component | 1.35 | -0.58 | U | NC | 5 | CG | - |
| KGA90657.1 | adhE | aldehyde-alcohol dehydrogenase | 0.08 | -1.02 | NC | D | 6 | C | ko:K04072 |
| KGA90518.1 | htpG | histidine kinase-, DNA gyrase B-, and HSP90-like ATPase family protein | -0.22 | 0.05 | NC | NC | 1 | O | ko:K04079 |
| KGA91670.1 | lon | ATP-dependent protease La | -0.27 | -0.11 | NC | NC | 1 | O | ko:K01338 |
| KGA90994.1 | secA | preprotein translocase, SecA subunit | -0.03 | -0.33 | NC | NC | 1 | U | ko:K03070 |
| KGA91000.1 | putA | bifunctional protein putA | 1.03 | 0.05 | U | NC | 5 | C | ko:K13821 |
| KGA92309.1 | ytfN | hypothetical protein DR94_2739 | -0.22 | -0.05 | NC | NC | 1 | S | ko:K09800 |
| KGA90346.1 | alaS | alanine--tRNA ligase | -0.52 | -0.50 | NC | NC | 1 | J | ko:K01872 |
| KGA91937.1 | fusA | translation elongation factor G | 1.35 | -0.35 | U | NC | 5 | J | ko:K02355 |
| KGA89005.1 | bamA | outer membrane assembly complex, YaeT protein | 0.99 | 0.22 | NC | NC | 1 | M | ko:K07277 |
| KGA92495.1 | gyrB | DNA gyrase, B subunit | 0.21 | -0.19 | NC | NC | 1 | L | ko:K02470 |
| KGA91927.1 | - | glycyl-tRNA synthetase beta subunit | -0.33 | 0.07 | NC | NC | 1 | J | - |
| KGA89012.1 | - | autotransporter beta-domain protein | 0.18 | 0.29 | NC | NC | 1 | O | ko:K12685 |
| KGA89123.1 | groL | chaperonin GroL | 0.15 | 0.18 | NC | NC | 1 | O | ko:K04077 |
| KGA88943.1 | ptrA | protease 3 | 0.34 | 0.02 | NC | NC | 1 | O | ko:K01407 |
| KGA89319.1 | ydgA | hypothetical protein DR94_1528 | -0.07 | 0.33 | NC | NC | 1 | S | - |
| KGA92312.1 | infB | translation initiation factor IF-2 | 0.74 | -0.05 | NC | NC | 1 | J | ko:K02519 |
| KGA91586.1 | tig | trigger factor | 0.13 | 0.21 | NC | NC | 1 | D | ko:K03545 |
| KGA89613.1 | rne | ribonuclease, Rne/Rng family domain protein | -0.20 | -0.19 | NC | NC | 1 | J | ko:K08300 |
| KGA90476.1 | narG | nitrate reductase, alpha subunit | 0.83 | 0.04 | NC | NC | 1 | C | ko:K00370 |
| KGA90506.1 | pta | phosphate acetyltransferase | 2.17 | -0.18 | U | NC | 5 | C | ko:K13788 |
| KGA89775.1 | cirA | tonB dependent receptor family protein | -0.10 | 0.29 | NC | NC | 1 | P | ko:K16089 |
| KGA89669.1 | arnA | bifunctional polymyxin resistance protein ArnA | 1.68 | -0.09 | U | NC | 5 | I | ko:K01784 |
| KGA90212.1 | mukB | hypothetical protein DR94_2335 | -0.20 | -0.20 | NC | NC | 1 | D | ko:K03632 |
| KGA91588.1 | carB | carbamoyl-phosphate synthase large chain | 0.52 | -0.46 | NC | NC | 1 | F | ko:K01955 |
| KGA91093.1 | ptsI | phosphoenolpyruvate-protein phosphotransferase | -0.26 | -0.42 | NC | NC | 1 | G | ko:K08483 |
| KGA90265.1 | leuS | leucine--tRNA ligase | 1.04 | -0.36 | U | NC | 5 | J | ko:K01869 |
| KGA92176.1 | valS | valine--tRNA ligase | 0.37 | -0.44 | NC | NC | 1 | J | ko:K01873 |
| KGA90924.1 | - | catalase | -0.23 | 0.09 | NC | NC | 1 | P | ko:K03781 |
| KGA91813.1 | ppiD | peptidyl-prolyl cis-trans isomerase D | -0.36 | 0.13 | NC | NC | 1 | O | ko:K03770 |
| KGA90729.1 | mutS | DNA mismatch repair protein MutS | 0.24 | -0.04 | NC | NC | 1 | L | ko:K03555 |
| KGA89664.1 | aspS | aspartate--tRNA ligase | 0.84 | -0.64 | NC | NC | 1 | J | ko:K01876 |
| KGA90725.1 | gyrA | DNA gyrase, A subunit | 1.21 | -0.25 | U | NC | 5 | L | ko:K02469 |
| KGA91510.1 | cpdB | 2',3'-cyclic-nucleotide 2'-phosphodiesterase | -0.20 | 0.14 | NC | NC | 1 | F | ko:K01119 |
| KGA90696.1 | guaA | GMP synthase glutamine-hydrolyzing | 2.08 | 0.04 | U | NC | 5 | F | ko:K01951 |
| KGA90754.1 | fliC | flagellin 1 | -1.14 | -1.34 | D | D | 3 | N | ko:K02406 |
| KGA91167.1 | ppsA | phosphoenolpyruvate synthase | -0.02 | -0.05 | NC | NC | 1 | H | ko:K01007 |
| KGA89912.1 | pepN | aminopeptidase N | 0.44 | -0.74 | NC | NC | 1 | E | ko:K01256 |
| KGA91050.1 | - | tonB dependent receptor family protein | -0.08 | 0.32 | NC | NC | 1 | S | - |
| KGA91021.1 | nuoG | NADH dehydrogenase (quinone), G subunit | 0.04 | -0.10 | NC | NC | 1 | C | ko:K00336 |
| KGA92456.1 | ppc | phosphoenolpyruvate carboxylase family protein | 0.37 | 0.22 | NC | NC | 1 | H | ko:K01595 |
| KGA92343.1 | hslU | ATP-dependent protease HslVU, ATPase subunit | -0.52 | -0.03 | NC | NC | 1 | O | ko:K03667 |
| KGA92282.1 | purA | adenylosuccinate synthase | 0.18 | -0.26 | NC | NC | 1 | F | ko:K01939 |
| KGA91532.1 | ftsY | signal recognition particle-docking protein FtsY | -0.41 | 0.09 | NC | NC | 1 | D | ko:K03110 |
| KGA91940.1 | yhgF | S1 RNA binding domain protein | -0.22 | -0.22 | NC | NC | 1 | K | ko:K06959 |
| KGA92295.1 | rnr | ribonuclease R | 0.17 | -0.13 | NC | NC | 1 | J | ko:K12573 |
| KGA89719.1 | thrS | threonine--tRNA ligase | -1.06 | -1.29 | D | D | 3 | J | ko:K01868 |
| KGA91165.1 | nuoC | NADH-quinone oxidoreductase subunit C/D | 0.54 | -0.54 | NC | NC | 1 | C | ko:K13378 |
| KGA91488.1 | clpX | ATP-dependent Clp protease, ATP-binding subunit ClpX | -0.54 | -0.01 | NC | NC | 1 | O | ko:K03544 |
| KGA89550.1 | acnA | aconitate hydratase 1 | 1.63 | 0.18 | U | NC | 5 | C | ko:K01681 |
| KGA92272.1 | ftsH | ATP-dependent zinc metalloprotease FtsH | -0.55 | -0.04 | NC | NC | 1 | D | ko:K03798 |
| KGA90254.1 | rpsA | ribosomal protein S1 | 1.56 | -0.08 | U | NC | 5 | J | ko:K02945 |
| KGA91894.1 | pckA | phosphoenolpyruvate carboxykinase | 1.43 | 1.76 | U | U | 2 | F | ko:K01610 |
| KGA92245.1 | nusA | transcription elongation protein nusA | -0.56 | -0.20 | NC | NC | 1 | K | ko:K02600 |
| KGA92069.1 | - | oligopeptidase A | 0.27 | -0.51 | NC | NC | 1 | O | - |
| KGA89186.1 | uvrA | excinuclease ABC subunit A | -0.17 | -0.07 | NC | NC | 1 | L | ko:K03701 |
| KGA90738.1 | pykF | pyruvate kinase | 0.89 | -0.70 | NC | NC | 1 | G | ko:K00873 |
| KGA90833.1 | - | periplasmic oligopeptide-binding protein | -0.14 | 0.28 | NC | NC | 1 | T | - |
| KGA90938.1 | ushA | protein UshA | -0.09 | 0.28 | NC | NC | 1 | F | ko:K11751 |
| KGA90460.1 | frdA | fumarate reductase, flavoprotein subunit | 2.18 | -0.15 | U | NC | 5 | C | ko:K00244 |
| KGA88939.1 | - | proline--tRNA ligase | -0.45 | -0.17 | NC | NC | 1 | J | - |
| KGA92107.1 | rho | transcription termination factor Rho | -0.39 | -0.18 | NC | NC | 1 | K | ko:K03628 |
| KGA89953.1 | maeA | NAD-dependent malic enzyme | -0.66 | -0.26 | NC | NC | 1 | C | ko:K00027 |
| KGA91508.1 | - | transketolase | 2.05 | -0.12 | U | NC | 5 | G | - |
| KGA91502.1 | - | hypothetical protein DR94_1786 | -0.17 | 0.27 | NC | NC | 1 | F | ko:K01081 |
| KGA89336.1 | pheT | phenylalanine--tRNA ligase, beta subunit | 1.00 | -0.53 | NC | NC | 1 | J | ko:K01890 |
| KGA90023.1 | metG | methionyl-tRNA synthetase | -0.08 | -0.10 | NC | NC | 1 | J | ko:K01874 |
| KGA89806.1 | ompA | outer membrane protein A | 0.43 | 0.13 | NC | NC | 1 | M | ko:K03286 |
| KGA91935.1 | polA | DNA polymerase I | 0.35 | -0.40 | NC | NC | 1 | L | ko:K02335 |
| KGA92467.1 | atpD | ATP synthase F1, beta subunit | -0.31 | 0.07 | NC | NC | 1 | F | ko:K02112 |
| KGA91818.1 | degQ | peptidase Do family protein | -0.03 | 0.17 | NC | NC | 1 | M | ko:K04771 |
| KGA88903.1 | deoB | phosphopentomutase | -0.13 | 0.08 | NC | NC | 1 | F | ko:K01839 |
| KGA91192.1 | gcvP | glycine dehydrogenase | 0.82 | 1.53 | NC | U | 7 | E | ko:K00281 |
| KGA92356.1 | glpK | glycerol kinase | 1.10 | -0.18 | U | NC | 5 | F | ko:K00864 |
| KGA92346.1 | - | formate dehydrogenase, alpha subunit | 0.45 | -0.05 | NC | NC | 1 | S | - |
| KGA91796.1 | yjjK | heme ABC exporter, ATP-binding protein CcmA | -0.52 | -0.03 | NC | NC | 1 | S | ko:K06020 |
| KGA91447.1 | - | phosphopyruvate hydratase | 0.23 | 0.17 | NC | NC | 1 | G | - |
| KGA90060.1 | glnS | glutamine--tRNA ligase | 0.23 | 0.21 | NC | NC | 1 | J | ko:K01886 |
| KGA91092.1 | aldB | aldehyde dehydrogenase family protein | -0.07 | -0.26 | NC | NC | 1 | C | - |
| KGA91819.1 | pyrG | CTP synthase | 0.69 | -0.69 | NC | NC | 1 | F | ko:K01937 |
| KGA91977.1 | dppA | bacterial extracellular solute-binding s, 5 Middle family protein | -0.32 | 0.34 | NC | NC | 1 | E | ko:K02035 |
| KGA92361.1 | atpA | ATP synthase F1, alpha subunit | 1.38 | -0.14 | U | NC | 5 | F | ko:K02111 |
| KGA91186.1 | lysS | lysine--tRNA ligase | 0.52 | -0.45 | NC | NC | 1 | J | ko:K04567 |
| KGA92502.1 | ugd | UDP-glucose 6-dehydrogenase | 1.10 | -0.26 | U | NC | 5 | C | ko:K00012 |
| KGA89410.1 | topA | DNA topoisomerase I | 0.21 | -0.38 | NC | NC | 1 | L | ko:K03168 |
| KGA89926.1 | serS | serine--tRNA ligase | -0.19 | 0.06 | NC | NC | 1 | J | ko:K01875 |
| KGA90690.1 | - | glyceraldehyde-3-phosphate dehydrogenase, type I | 1.58 | -0.01 | U | NC | 5 | F | ko:K00134 |
| KGA90094.1 | dacC | D-alanyl-D-alanine carboxypeptidase dacC | -0.14 | 0.21 | NC | NC | 1 | M | ko:K07258 |
| KGA91445.1 | relA | GTP pyrophosphokinase | -0.06 | 0.20 | NC | NC | 1 | KT | ko:K00951 |
| KGA91203.1 | pepB | peptidase B | -0.46 | -0.37 | NC | NC | 1 | E | ko:K07751 |
| KGA90160.1 | pgm | phosphoglucomutase, alpha-D-glucose phosphate-specific | -0.30 | -0.45 | NC | NC | 1 | G | ko:K01835 |
| KGA90771.1 | - | serine hydroxymethyltransferase | -0.11 | 0.09 | NC | NC | 1 | E | - |
| KGA91397.1 | nrdA | ribonucleoside-diphosphate reductase, alpha subunit | 0.65 | 0.80 | NC | NC | 1 | F | ko:K00525 |
| KGA89881.1 | fumC | fumarate hydratase, class II | -0.28 | -0.14 | NC | NC | 1 | C | ko:K01679 |
| KGA91777.1 | pgk | phosphoglycerate kinase | 0.15 | 0.06 | NC | NC | 1 | F | ko:K00927 |
| KGA89231.1 | rpoA | DNA-directed RNA polymerase, alpha subunit | -0.15 | -0.23 | NC | NC | 1 | K | ko:K03040 |
| KGA91082.1 | gltX | glutamate--tRNA ligase | -0.23 | -0.23 | NC | NC | 1 | J | ko:K01885 |
| KGA89213.1 | pgi | glucose-6-phosphate isomerase | 1.34 | -0.19 | U | NC | 5 | F | ko:K01810 |
| KGA89747.1 | asnS | asparagine--tRNA ligase | 0.58 | -0.52 | NC | NC | 1 | J | ko:K01893 |
| KGA91383.1 | - | phosphoribosylaminoimidazolesuccinocarboxamide synthase | 0.20 | -0.03 | NC | NC | 1 | F | - |
| KGA90191.1 | sucC | succinyl-CoA ligase [ADP-forming] subunit beta | 0.15 | -0.51 | NC | NC | 1 | F | ko:K01903 |
| KGA90087.1 | gnd | 6-phosphogluconate dehydrogenase | 0.67 | -0.59 | NC | NC | 1 | H | ko:K00033 |
| KGA89026.1 | lptD | LPS-assembly protein lptD | 0.94 | -0.33 | NC | NC | 1 | M | ko:K04744 |
| KGA92002.1 | tuf | translation elongation factor Tu | -0.99 | -0.65 | NC | NC | 1 | J | ko:K02358 |
| KGA89261.1 | tuf | translation elongation factor Tu | -0.14 | -0.47 | NC | NC | 1 | J | ko:K02358 |
| KGA91955.1 | purH | phosphoribosylaminoimidazolecarboxamide formyltransferase/IMP cyclohydrolase | 1.76 | -0.09 | U | NC | 5 | F | ko:K00602 |
| KGA92310.1 | - | putative uroporphyrinogen-III C-methyltransferase | 0.04 | 0.34 | NC | NC | 1 | S | - |
| KGA92300.1 | pyrB | aspartate carbamoyltransferase | -0.88 | -0.35 | NC | NC | 1 | F | ko:K00609 |
| KGA90570.1 | lpdA | dihydrolipoyl dehydrogenase | 0.83 | 0.05 | NC | NC | 1 | C | ko:K00382 |
| KGA89574.1 | icd | isocitrate dehydrogenase, NADP-dependent | 0.57 | -0.60 | NC | NC | 1 | C | ko:K00031 |
| KGA91259.1 | maeB | NADP-dependent malic enzyme | 1.61 | 0.10 | U | NC | 5 | C | ko:K00027 |
| KGA89732.1 | argS | arginine--tRNA ligase | 0.59 | -0.03 | NC | NC | 1 | J | ko:K01887 |
| KGA90340.1 | pepD | aminoacyl-histidine dipeptidase | -0.22 | 0.09 | NC | NC | 1 | E | ko:K01270 |
| KGA90790.1 | aceF | dihydrolipoyllysine-residue acetyltransferase | -0.38 | -0.22 | NC | NC | 1 | C | ko:K00627 |
| KGA89426.1 | zwf | glucose-6-phosphate dehydrogenase | -0.38 | -0.60 | NC | NC | 1 | G | ko:K00036 |
| KGA89864.1 | prc | tail-specific protease | 0.10 | -0.33 | NC | NC | 1 | M | ko:K03797 |
| KGA91938.1 | fliY_2 | bacterial extracellular solute-binding s, 3 family protein | -0.16 | 0.25 | NC | NC | 1 | ET | ko:K02030 |
| KGA89709.1 | ompF | gram-negative porin family protein | 0.56 | 0.11 | NC | NC | 1 | M | ko:K09476 |
| KGA91281.1 | cysK | cysteine synthase A | -0.06 | -0.07 | NC | NC | 1 | E | ko:K01738 |
| KGA90869.1 | pepP | xaa-Pro aminopeptidase | -0.69 | -0.06 | NC | NC | 1 | E | ko:K01262 |
| KGA89974.1 | sucB | dihydrolipoyllysine-residue succinyltransferase, E2 component of oxoglutarate dehydrogenase complex | -0.01 | 0.15 | NC | NC | 1 | C | ko:K00658 |
| KGA91648.1 | dnaX | DNA polymerase III, subunit gamma and tau | -0.50 | -0.47 | NC | NC | 1 | H | ko:K02343 |
| KGA92057.1 | fdsA | formate dehydrogenase, alpha subunit | -1.27 | 0.39 | D | NC | 4 | C | ko:K00123 |
| KGA90440.1 | narH | nitrate reductase, beta subunit | -0.49 | 0.26 | NC | NC | 1 | C | ko:K00371 |
| KGA90442.1 | glpQ | glycerophosphoryl diester phosphodiesterase | 0.08 | 0.26 | NC | NC | 1 | C | ko:K01126 |
| KGA90995.1 | copA | copper-translocating P-type ATPase | 0.03 | 0.17 | NC | NC | 1 | P | ko:K17686 |
| KGA92202.1 | hflC | HflC protein | -0.50 | 0.02 | NC | NC | 1 | O | ko:K04087 |
| KGA89889.1 | cbpA | curved DNA-binding protein | 0.16 | 0.25 | NC | NC | 1 | O | ko:K05516 |
| KGA89673.1 | tyrS | tyrosine--tRNA ligase | -0.20 | 0.34 | NC | NC | 1 | J | ko:K01866 |
| KGA90563.1 | cheD | methyl-accepting chemotaxis protein I | -0.04 | 0.28 | NC | NC | 1 | NT | ko:K03406 |
| KGA92145.1 | pepA | cytosol aminopeptidase | -0.20 | -0.57 | NC | NC | 1 | E | ko:K01255 |
| KGA89939.1 | sdhA | succinate dehydrogenase, flavoprotein subunit | 1.53 | 0.04 | U | NC | 5 | C | ko:K00239 |
| KGA91555.1 | tal | transaldolase | 0.17 | -0.20 | NC | NC | 1 | F | ko:K00616 |
| KGA91897.1 | - | glutamine synthetase, type I | -0.73 | 0.26 | NC | NC | 1 | E | - |
| KGA92498.1 | poxB | pyruvate dehydrogenase ubiquinone | 1.13 | -0.31 | U | NC | 5 | EH | ko:K00156 |
| KGA91253.1 | ftsZ | cell division protein FtsZ | -0.48 | -0.15 | NC | NC | 1 | D | ko:K03531 |
| KGA88878.1 | rpoD | RNA polymerase sigma factor rpoD | -0.27 | -0.14 | NC | NC | 1 | K | ko:K03086 |
| KGA91920.1 | - | FAD binding domain protein | 0.86 | -0.17 | NC | NC | 1 | E | - |
| KGA88904.1 | tsf | translation elongation factor Ts | -0.28 | -0.16 | NC | NC | 1 | J | ko:K02357 |
| KGA91159.1 | hmuR | TonB-dependent hemoglobin/transferrin/lactoferrin receptor family protein | 1.73 | 0.13 | U | NC | 5 | P | ko:K16087 |
| KGA92189.1 | mdh | malate dehydrogenase, NAD-dependent | 0.26 | 0.23 | NC | NC | 1 | C | ko:K00024 |
| KGA88959.1 | deoA | thymidine phosphorylase | -0.18 | -0.27 | NC | NC | 1 | F | ko:K00758 |
| KGA89129.1 | tyrB | aromatic-amino-acid aminotransferase | -0.76 | -0.01 | NC | NC | 1 | H | ko:K00832 |
| KGA90500.1 | guaB | inosine-5'-monophosphate dehydrogenase | -0.44 | -0.36 | NC | NC | 1 | F | ko:K00088 |
| KGA89047.1 | metQ | D-methionine-binding lipoprotein metQ | -0.04 | 0.22 | NC | NC | 1 | M | ko:K02072 |
| KGA91476.1 | - | threonine synthase | -0.29 | 0.11 | NC | NC | 1 | E | - |
| KGA89162.1 | aspA | aspartate ammonia-lyase | 0.36 | -0.39 | NC | NC | 1 | E | ko:K01744 |
| KGA90301.1 | ffh | signal recognition particle protein | -0.55 | 0.26 | NC | NC | 1 | U | ko:K03106 |
| KGA91328.1 | bamC | lipoprotein 34 | -0.03 | 0.51 | NC | NC | 1 | M | ko:K07287 |
| KGA92288.1 | pnp | polyribonucleotide nucleotidyltransferase | 1.69 | -0.17 | U | NC | 5 | J | ko:K00962 |
| KGA92081.1 | - | protein yhjJ | -0.27 | 0.53 | NC | NC | 1 | O | - |
| KGA90662.1 | nuoF | NADH oxidoreductase (quinone), F subunit | 0.25 | -0.12 | NC | NC | 1 | C | ko:K00335 |
| KGA88973.1 | hldE | bifunctional protein RfaE, domain I | -0.22 | 0.09 | NC | NC | 1 | F | ko:K03272 |
| KGA92387.1 | glmS | glutamine-fructose-6-phosphate transaminase | 1.60 | 0.02 | U | NC | 5 | H | ko:K00820 |
| KGA88925.1 | rapA | RNA polymerase-associated protein rapA | -0.12 | -0.65 | NC | NC | 1 | K | ko:K03580 |
| KGA91645.1 | thrA | bifunctional aspartokinase/homoserine dehydrogenase 1 | 0.35 | 0.51 | NC | NC | 1 | E | ko:K12524 |
| KGA92493.1 | metL | bifunctional aspartokinase/homoserine dehydrogenase 2 | -1.59 | 0.32 | D | NC | 4 | E | ko:K12524 |
| KGA91497.1 | dnaJ | chaperone protein DnaJ | -0.59 | -0.07 | NC | NC | 1 | O | ko:K03686 |
| KGA91576.1 | - | ytxH-like family protein | -0.19 | 0.47 | NC | NC | 1 | - | - |
| KGA92174.1 | glmM | phosphoglucosamine mutase | 0.86 | -0.25 | NC | NC | 1 | G | ko:K03431 |
| KGA89462.1 | - | peptidase C69 family protein | 0.05 | 0.03 | NC | NC | 1 | M | - |
| KGA90236.1 | gltI | bacterial extracellular solute-binding s, 3 family protein | 0.26 | 0.31 | NC | NC | 1 | ET | ko:K10001 |
| KGA91352.1 | gadB | glutamate decarboxylase | -0.47 | -0.83 | NC | NC | 1 | E | ko:K01580 |
| KGA92362.1 | atpG | ATP synthase F1, gamma subunit | -0.51 | 0.00 | NC | NC | 1 | C | ko:K02115 |
| KGA91948.1 | glpD | FAD dependent oxidoreductase family protein | 0.38 | -0.32 | NC | NC | 1 | C | ko:K00111 |
| KGA89142.1 | aceA | isocitrate lyase | 0.10 | -0.34 | NC | NC | 1 | C | ko:K01637 |
| KGA89692.1 | pykA | pyruvate kinase | 0.31 | 0.31 | NC | NC | 1 | G | ko:K00873 |
| KGA91876.1 | typA | GTP-binding protein TypA/BipA | 2.51 | -0.10 | U | NC | 5 | T | ko:K06207 |
| KGA89055.1 | hyfG | hydrogenase-4 component G | 2.03 | 0.05 | U | NC | 5 | C | ko:K12142 |
| KGA92088.1 | acsA | acetate--CoA ligase | 1.32 | 0.45 | U | NC | 5 | I | ko:K01895 |
| KGA90879.1 | - | hypothetical protein DR94_229 | 1.01 | 0.05 | U | NC | 5 | O | - |
| KGA91103.1 | ligA | DNA ligase, NAD-dependent | 0.49 | 0.06 | NC | NC | 1 | L | ko:K01972 |
| KGA89866.1 | pepT | peptidase T | -0.17 | -0.36 | NC | NC | 1 | E | ko:K01258 |
| KGA89971.1 | gltA | citrate (Si)-synthase | 2.12 | -0.23 | U | NC | 5 | H | ko:K01647 |
| KGA90147.1 | serC | phosphoserine transaminase | -0.50 | -0.03 | NC | NC | 1 | H | ko:K00831 |
| KGA88975.1 | YPO2511 | hypothetical protein DR94_3049 | 1.11 | 0.31 | U | NC | 5 | O | - |
| KGA88879.1 | - | putative lysine decarboxylase family protein | -0.07 | -0.90 | NC | NC | 1 | S | - |
| KGA91206.1 | - | malate dehydrogenase | 1.08 | 0.48 | U | NC | 5 | S | - |
| KGA88891.1 | - | PPIC-type PPIASE domain protein | -0.26 | 0.30 | NC | NC | 1 | O | - |
| KGA91205.1 | - | cysteine--tRNA ligase | -0.23 | -0.23 | NC | NC | 1 | J | - |
| KGA91100.1 | hcp | hydroxylamine reductase | -1.47 | 0.41 | D | NC | 4 | C | ko:K05601 |
| KGA89734.1 | hrpA | ATP-dependent helicase HrpA | -1.44 | 0.17 | D | NC | 4 | L | ko:K03578 |
| KGA90463.1 | glpA | glycerol-3-phosphate dehydrogenase, anaerobic, A subunit | 0.37 | -0.08 | NC | NC | 1 | C | ko:K00111 |
| KGA91007.1 | GALT | galactose-1-phosphate uridylyltransferase | -0.49 | -0.57 | NC | NC | 1 | C | ko:K00965 |
| KGA89548.1 | dld | D-lactate dehydrogenase | 0.61 | 1.06 | NC | U | 7 | C | ko:K03777 |
| KGA88923.1 | - | hypothetical protein DR94_3027 | 1.83 | 0.15 | U | NC | 5 | H | - |
[truncated: 319,440 more chars]
